# Amino Acids and Ribose: Drivers of Protein and RNA Fermentation by Ingested Bacteria of a Primitive Gut Ecosystem

**DOI:** 10.1128/AEM.01297-19

**Published:** 2019-09-17

**Authors:** Lydia Zeibich, Maraike Staege, Oliver Schmidt, Harold L. Drake

**Affiliations:** a Department of Ecological Microbiology, University of Bayreuth, Bayreuth, Germany; University of Illinois at Urbana-Champaign

**Keywords:** amino acid fermentation, anaerobes, gut ecosystem, invertebrate microbiology

## Abstract

Earthworms are among the most primitive animals and are of fundamental importance to the turnover of organic matter in the terrestrial biosphere. These invertebrates ingest materials that are colonized by microbes, some of which are subject to disruption by the crop/gizzard or other lytic events during gut passage. Protein and RNA are dominant polymers of disrupted microbial cells, and these biopolymers facilitate robust fermentations by surviving ingested bacteria. To further resolve these fermentations, amino acids and ribose (as fermentable constituents of protein and RNA, respectively) were evaluated as potential drivers of fermentation in gut content of the model earthworm Lumbricus terrestris (taxa were examined with 16S rRNA-based analyses). Of eight amino acids tested, glutamate, aspartate, and threonine were most stimulatory and yielded dissimilar fermentations facilitated by contrasting taxa (e.g., glutamate stimulated the *Fusobacteriaceae* and yielded H_2_ and formate, whereas aspartate stimulated the *Aeromonadaceae* and yielded succinate and propionate). A marginal Stickland fermentation was associated with the *Peptostreptococcaceae* and *Lachnospiraceae*. Ribose fermentation yielded a complex product profile facilitated primarily by the *Aeromonadaceae*. The transient nature of succinate was linked to its decarboxylation to propionate and the *Fusobacteriaceae*, whereas the transient nature of formate was linked to formate-hydrogen lyase activity and the *Peptostreptococcaceae*. These findings reinforce the likelihood that (i) the animal host and hosted fermentative bacteria compete for the constituents of protein and RNA in the alimentary canal and (ii) diverse gut fermenters engaged in the fermentation of these constituents produce products that can be utilized by earthworms.

**IMPORTANCE** Animal health is linked to gut ecosystems whose primary function is normally the digestion of dietary matter. Earthworms are representative of one of the oldest known animal lineages and, despite their primitive nature, have unique environmental impact by virtue of their dietary consumption of their habitat, i.e., soil-associated matter. A resident gut community is a hallmark of many gut ecosystems of evolutionarily more advanced animals, but the alimentary canal of earthworms is dominated by ingested transient soil microbes. Protein and RNA are (i) the primary organic components of microbial cells that are subject to lysis during gut passage and (ii) fermentable dietary substrates in the alimentary canal. This study examined the gut-associated fermentation of constituents of these biopolymers to determine how their fermentation is integrated to the microbiological dynamics of the gut and might contribute to earthworm-linked transformations of organic matter in the terrestrial biosphere.

## Abstract (precis)

Animal health is linked to gut ecosystems whose primary function is normally the digestion of dietary matter. Earthworms are representative of one of the oldest known animal lineages and, despite their primitive nature, have unique environmental impact by virtue of their dietary consumption of their habitat, i.e., soil-associated matter. A resident gut community is a hallmark of many gut ecosystems of evolutionarily more advanced animals, but the alimentary canal of earthworms is dominated by ingested transient soil microbes. Protein and RNA are (i) the primary organic components of microbial cells that are subject to lysis during gut passage and (ii) fermentable dietary substrates in the alimentary canal. This study examined the gut-associated fermentation of constituents of these biopolymers to determine how their fermentation is integrated to the microbiological dynamics of the gut and might contribute to earthworm-linked transformations of organic matter in the terrestrial biosphere.

## INTRODUCTION

Fossil evidence suggests that ancestors of earthworms (annelids) and other worm-like animals existed 0.5-to-1.1 billion years ago (1, 2), making these invertebrates evolutionarily among the oldest known animal lineages (e.g., winged insects, termites, and ruminants date back approximately 300, 150, and 50 million years ago, respectively [3–5]). Earthworms are mostly unseen in the environment, but these primitive subsurface animals can reach enormous densities and have profound impact on the cycling of matter in the terrestrial biosphere, an impact linked to the ingestion of soil, plant material, and associated microorganisms (6–8). Although earthworms have positive effects on soil fertility and are of value for vermicomposting environmental wastes (9–14), their invasiveness may have negative consequences (15, 16).

The evolution and ecological impact of earthworms are linked in part to the utility of their anoxic alimentary canal in which fermentation is the main microbial process and can yield up to approximately 30 mM fatty acids in the aqueous phase of the mid-gut (17–19). In contrast to more highly evolved gut ecosystems in which complex resident microbial communities occur (20–23), fermenters in the gut of earthworms appear to be dominated by ingested transients that pass through the alimentary canal in 1 day or less (24). Ingested plant material and microorganisms are subject to disruption by the abrasive action of the crop/gizzard, resulting in the release of diverse nutrients, including biopolymers (25, 26). Bacteriophages are abundant in ingested soil, and bacteria in the gut could also be disrupted by phage-facilitated lysis (27–30). Furthermore, the lysis of bacteria by earthworm-derived lysozyme (31, 32) and the bacterial lysis of fungi (33, 34) could also contribute to the disruption of microbial cells in the gut.

Microbes can contain more than 50% protein and 20% RNA on a dry weight basis (35–39). Assuming these amounts apply to microbial cytoplasm with an 80% water content, cytoplasm has approximately 1 M polymeric amino acids and 100 mM polymeric ribose (these estimates assume the average amino acid has a molecular weight of 100 and ribose constitutes 40% of RNA). Thus, a viable microbe in the immediate vicinity of the cytoplasm of a ruptured cell would experience an extraordinarily high availability of protein- and RNA-based nutrients that could trigger physiological responses. Indeed, the capacity of gut-associated bacteria to rapidly ferment these biopolymers contributes to the anaerobic microbial potentials of the alimentary canal (24, 40).

In addition to protein from ruptured microbes, the glycoprotein-rich mucus that is excreted into the alimentary canal (41, 42) and ingested plant biomass (e.g., plant shoots and debris [43, 44]) are other sources of protein for fermentative gut microbes. Independent of its origin, the amount of protein in the alimentary canal decreases sharply from anterior to posterior, and the amount of ammonium in the gut increases inversely (45). Furthermore, the amounts of ammonium in the gut and earthworm casts are very high compared to the negligible amounts in preingested soil (7, 46). These trends suggest that (i) gut fermentation of protein leads to the enrichment of ammonium in the gut via the deamination of amino acids and (ii) cast-linked enhancement of amino acid-derived ammonium in soil might impact soil nitrification and plant growth.

The fermentation of protein is dependent on diverse proteases that yield fermentable amino acids (47). There is a substantial amount of information on (i) gut fermentation of protein in higher animals (48–53) and (ii) catabolic processes by which amino acids can be fermented, including the Stickland reaction in which one amino acid serves as an electron donor and another amino acid serves as an electron acceptor (54, 55). The occurrence of up to nearly 2 mM amino acids in the aqueous phase of gut content (7, 19) is consistent with the occurrence of proteases in the alimentary canal and casts of earthworms (42, 56). The strong enhancement of gut content fermentation by protein (40) and the availability of amino acids in the gut (7, 19) corroborate the likelihood that amino acids are subject to fermentation during gut passage. However, the amino acid-specific response of a given fermentative taxon in the gut is unresolved.

To our knowledge, published information on the fermentation of RNA is scant (40). The fermentation of RNA is dependent on its initial degradation by hydrolytic or phosphorolytic RNases that yield monophosphorylated or diphosphorylated nucleotides, respectively, which can be further degraded and yield ribose, purines, and pyrimidines (57). In this regard, ribose is likely the primary fermentable component of RNA (40); however, the taxa and associated activities responsible for ribose fermentation are not resolved. The phosphoketolase pathway and the pentose phosphate cycle are processes by which pentoses such as ribose can be fermented to diverse products indicative of those found in the alimentary canal (54, 58, 59). These catabolic processes are in contrast to those utilized to ferment amino acids (54, 60, 61), suggesting that, in a complex community, different taxa might be engaged in these contrasting processes.

The microbes involved in the fermentation of amino acids and ribose in the gut theoretically compete with the earthworm for these substrates. Likewise, as with other animals, the fatty acids produced by these fermenters are subject to utilization by earthworms (18, 19, 62, 63). Thus, these gut fermenters are likely both competitive and beneficial relative to the earthworm host and, as such, important to the overall microbiological dynamics of the alimentary canal. However, as noted above, the microbiological processes and associated taxa responsible for these fermentations are not known. These collective considerations and the fact that protein and RNA stimulate dissimilar taxa (40) prompted us to postulate that amino acids and ribose are subject to fermentation by contrasting gut taxa. Utilizing the model earthworm Lumbricus terrestris, this postulate was examined by resolving the fermentative response of gut content when challenged with amino acids or ribose and by utilizing 16S rRNA-based analyses to determine which taxa were associated with these fermentations.

## RESULTS

### Amino acid-based fermentation in gut content of *L. terrestris*.

Eight representative amino acids that are known to be fermentable (54, 60, 61) (i.e., alanine, aspartate, glutamate, glycine, leucine, threonine, tyrosine, and valine) were evaluated in a preliminary study for their capacity to stimulate fermentation in anoxic gut content microcosms. Only glutamate, aspartate, and threonine yielded a strong enhancement of fermentation (see Fig. S1 and Table S1 in the supplemental material). Casamino Acids (a mixture of common amino acids) also stimulated fermentation. These preliminary findings suggested that stimulation of fermentation was restricted to specific, rather than all, amino acids. Glutamate, aspartate, threonine, and Casamino Acids were selected for more detailed studies, and the potential for Stickland fermentation was assessed with glycine and either alanine or valine.

**FIG 1:**
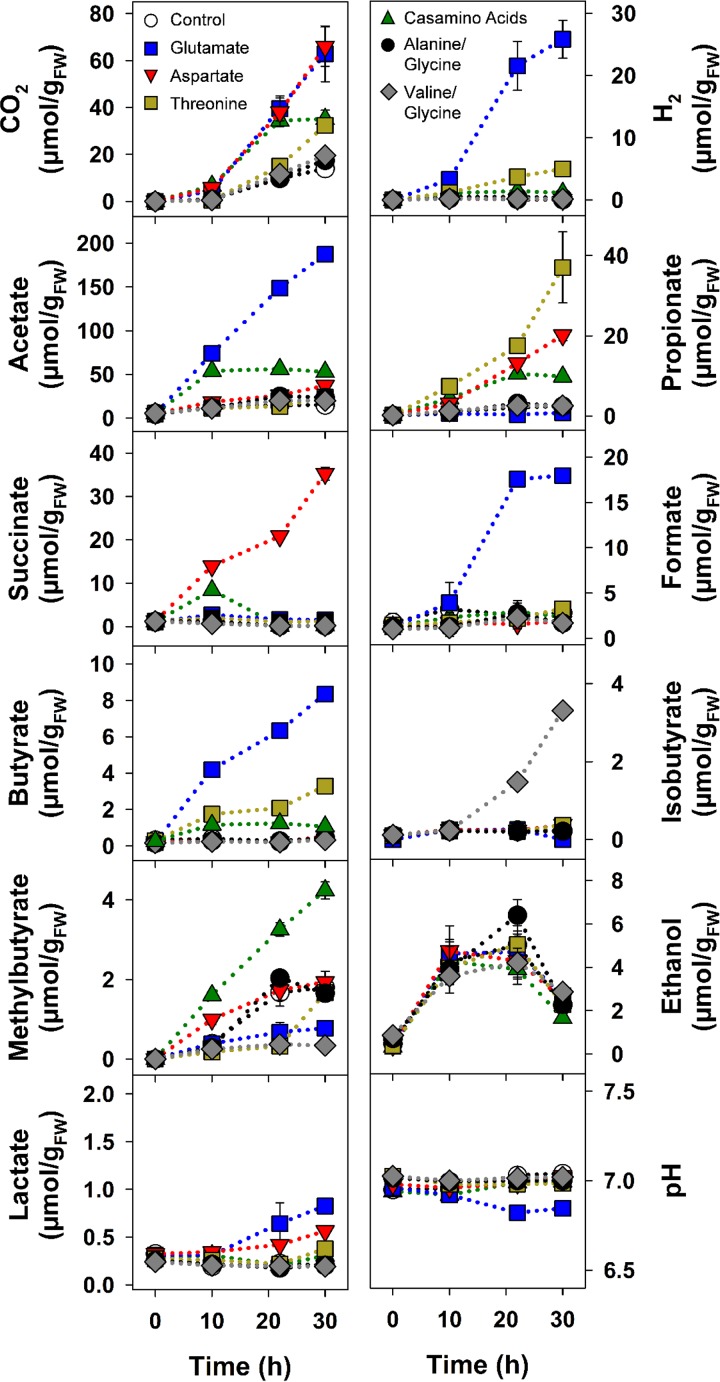
Effects of amino acids on the fermentation product profiles of anoxic microcosms of *L. terrestris* gut contents. Initial concentrations approximated 10 mM for Casamino Acids, glutamate, aspartate, threonine, and glycine and 5 mM for alanine and valine; the control lacked supplement. Values are the arithmetic averages from three replicate analyses, and error bars indicate the standard deviations. Some standard deviations are smaller than the size of the symbol and therefore not apparent. FW, fresh weight.

**FIG 2:**
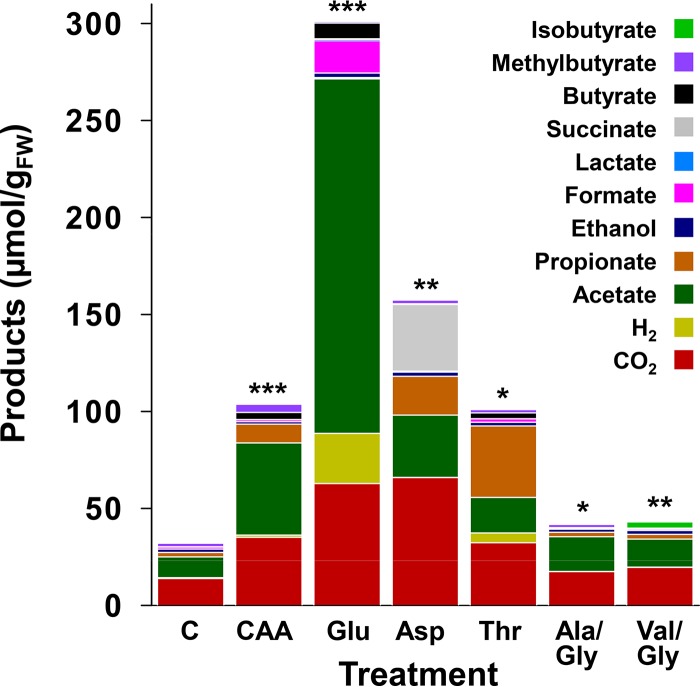
Collective amounts of fermentation products in amino acid treatments. Values are the averages from triplicate analyses shown in Fig. 1 and represent the net amounts of products at the end of the 30 h of incubation. The asterisks indicate significant differences between the collective amounts of products formed in control and amino acid treatments. *, *P ≤ *0.05; **, *P ≤ *0.01; ***, *P ≤ *0.001 by *t* test with unequal variances (see Table S2 in the supplemental material for *P* values, mean values, and variances); C, unsupplemented control; CAA, Casamino Acids; Glu, glutamate; Asp, aspartate; Thr, threonine; Ala, alanine; Gly, glycine; Val, valine; FW, fresh weight.

The glutamate treatment produced the strongest response, yielding diverse products without an apparent delay (Fig. 1), illustrating how much a single amino acid can stimulate gut fermenters. Several pathways can be utilized for glutamate fermentation, and glutamate fermenters can produce acetate, CO_2_, H_2_, formate, and butyrate (64, 65), products that increased significantly in the glutamate treatment (Fig. 1; see also Table S2). The theoretical recoveries of supplemental glutamate-derived carbon and reducing equivalents in the detected products approximated 90% and 92%, respectively (Table 1). These findings and the formation of nearly the same amount of ammonium to that of the supplemented glutamate (see Table S3) indicated that most of the glutamate was consumed. Aspartate treatments yielded large amounts of propionate and succinate, and threonine treatments yielded propionate as the dominant significant product (Fig. 1 and 2; Table S2). Propionate is also the dominant product of threonine fermentation by human colon microbiota (49). The comparative amounts of detected ammonium at the end of the incubation (Table S3) and the theoretical recoveries of carbon and reductant (Table 1) indicated that the amount of supplemental amino acid was adequate for the detected products and that glutamate was more effectively fermented than aspartate and threonine. The enrichment of ammonium in the amino acid treatments (Table S3) suggests that the high *in situ* amounts of ammonium in the alimentary canal and cast of earthworms (7, 46) might at least be partially derived from the deamination and fermentation of amino acids during gut passage.

**TABLE 1:** Estimated recoveries of carbon and reducing equivalents (i.e., electrons) in amino acid treatments^*a*^

Product	Recovery (%)^*b*^											
	Glutamate		Aspartate		Threonine		Alanine/glycine		Valine/glycine		Casamino Acids	
	Carbon	RE^*c*^	Carbon	RE	Carbon	RE	Carbon	RE	Carbon	RE	Carbon	RE
CO_2_	10	NA^*d*^	13	NA	4.6	NA	1.0	NA	1.3	NA	4.0	NA
H_2_	NA	2.8	NA	—^*e*^	NA	0.6	NA	—	NA	—	NA	0.1
Acetate	69	76	11	14	3.8	3.8	4.1	5.5	1.7	1.5	14	13
Ethanol	0.2	0.4	0.2	0.4	0.1	0.1	—	—	0.1	0.1	—	—
Lactate	0.4	0.4	0.3	0.4	0.2	0.2	0.1	0.1	0.1	0.0	0.1	0.1
Succinate	1.1	1.0	35	41	0.8	0.7	—	—	—	—	—	—
Formate	3.1	1.7	—	—	0.2	0.1	—	—	—	—	—	—
Butyrate	6.4	8.8	0.2	0.3	2.9	3.6	0.2	0.3	—	—	2.7	3.1
Propionate	—	—	13	21	26	30	0.0	0.1	0.1	0.1	4.2	4.7
Isobutyrate	—	—	0.1	0.2	0.1	0.2	—	—	2.7	3.1	0.1	0.1
Methylbutyrate	—	—	0.2	0.3	—	—	—	—	—	—	2.3	2.9
												
Total	90	92	73	77	39	39	5.4	6.0	5.9	5.0	28	25

a See Fig. 1 for product profiles. Net amounts of products formed in the unsupplemented control were subtracted from those of supplemented treatments.

b Recoveries are based on the amount of substrate provided. Values are based on the arithmetic average from three replicate analyses.

c RE, reducing equivalents.

d NA, not applicable.

e —, no net increase of the product during the incubation in supplemented treatments relative to that in the control treatment.

The co-amino acid treatments (alanine plus glycine or valine plus glycine) displayed a weak stimulation of fermentation; however, the collective amounts of products formed in these treatments were significantly higher than that of the control treatment (Fig. 2; Table S2). Furthermore, isobutyrate accumulated in valine/glycine treatments but was only detected at trace levels in all other amino acid and control treatments (Fig. 1). Nonetheless, theoretical carbon and electron recoveries were low in the co-amino acid treatments (Table 1), suggesting that gut content microbes had a minimal potential for a Stickland fermentation in a time frame indicative of gut passage.

Casamino Acids yielded acetate, CO_2_, propionate, and methylbutyrate as the main fermentation products (Fig. 1 and 2; Table S2). Methylbutyrate was not as abundant in any other treatment. Methylbutyrate also appeared to accumulate in the marginal fermentation observed in the leucine treatment in the preliminary study (Table S1), suggesting that the fermentation of leucine may have been at least partially responsible for the production of methylbutyrate in the Casamino Acids treatment. The theoretical recoveries of Casamino Acids-derived carbon and reducing equivalents in the detected products approximated 28% and 25%, respectively (Table 1). This result and the apparent inability of certain amino acids to greatly enhance fermentation (Fig. S1 and Table S1) suggested that the collective fermenters of gut content were not capable of fermenting all amino acids equally, a trend consistent with certain amino acids being less easily fermented by the microbial community of the human colon (49). Nonetheless, the enhanced formation of fermentation products in certain treatments (Fig. 1 and 2) indicated that gut fermenters were poised to respond to specific amino acids in a time frame indicative of gut passage.

### Fermentative bacterial families stimulated by amino acids.

**FIG 3:**
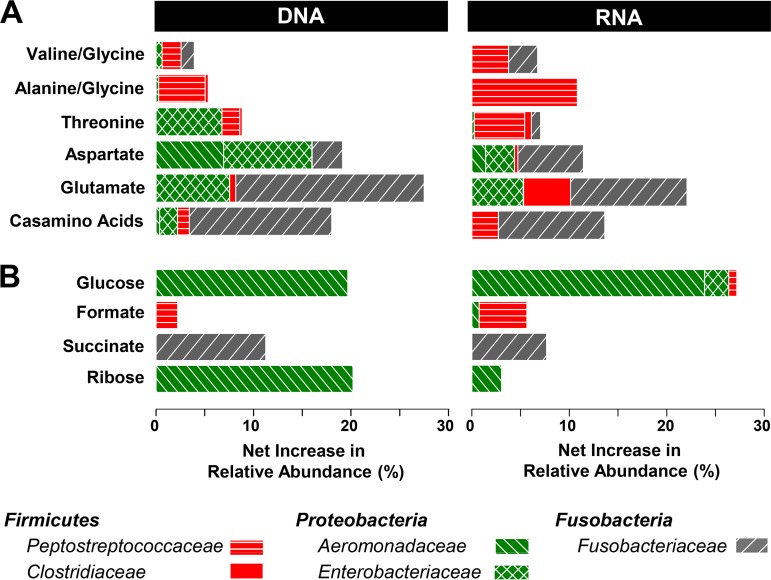
Net increases in 16S rRNA gene (DNA) and 16S rRNA (RNA) relative sequence abundances of bacterial families stimulated by supplemental amino acids (A), ribose, succinate, formate, and glucose (B) in *L. terrestris* gut content microcosms. The graphs are limited to families that displayed a net increase in relative sequence abundance of ≥4% in at least one treatment; the families are color coded to the respective phyla. Net increases of relative abundances were calculated as follows (8): (i) the calculation is based either on mean relative abundances when samples from the three replicates were analyzed separately (i.e., all RNA and DNA samples of control treatments and RNA samples at 30 h of supplemented treatments) or on single relative abundances when samples of the three replicates were pooled for sequence analyses (i.e., DNA samples at 0 h and 30 h and RNA samples at 0 h of supplemented treatments); (ii) mean or single relative abundances at the beginning of incubation were subtracted from those at the end of the 30 h of incubation for control and supplemented treatments; (iii) the resulting time-corrected relative abundances of control treatments were subtracted from those of supplemented treatments (negative time-corrected relative abundances of control treatments were ignored).

A total of 9,169,869 bacterial 16S rRNA and 16S rRNA gene sequences were obtained from the amino acid treatments, yielding 32 phyla (including candidate phyla), and rarefaction analyses indicated that the most abundant taxa were targeted (see Fig. S2). Based on net increases in relative sequence abundances, (i) the *Fusobacteriaceae* were mostly stimulated by glutamate, aspartate, and Casamino Acids, (ii) the *Aeromonadaceae* displayed only an apparent net increase in relative abundance in the aspartate treatment, (iii) the net relative abundance of the *Peptostreptococcaceae* increased mainly in Casamino Acids, threonine, alanine/glycine, and valine/glycine treatments, (iv) the *Clostridiaceae* responded most positively to glutamate, and (v) the *Enterobacteriaceae* responded most positively to glutamate and aspartate (Fig. 3A; see also Table S4). Statistical analysis indicated that the *Lachnospiraceae* were only associated with the marginal Stickland fermentations (Table S4). Consistent with the strong stimulation of the *Enterobacteriaceae*, *Clostridiaceae*, and *Fusobacteriaceae* in glutamate treatments (Fig. 3A; Table S4), the number of detected phylotypes, the number of expected phylotypes (Chao1), and Shannon indices of glutamate treatments were lower than those of unsupplemented controls (see Table S5).

The apparent shift in community members during the incubation was confirmed by nonmetric multidimensional scaling (NMDS) analysis of the detected phylotypes (97% sequence similarity) (see Fig. S3A and B). Shifts were more pronounced for amino acid treatments than in the unsupplemented control. The similarity of the bacterial community of different treatments at the beginning of incubation (see Fig. S4A) and in the triplicate analyses at the end of the incubation (Fig. S4B) illustrate the reproducibility of the phylogenic analyses and is reflected in the groupings of the NMDS analysis (Fig. S3A and B).

### Fermentation of ribose and effects of transient intermediates.

**FIG 4:**
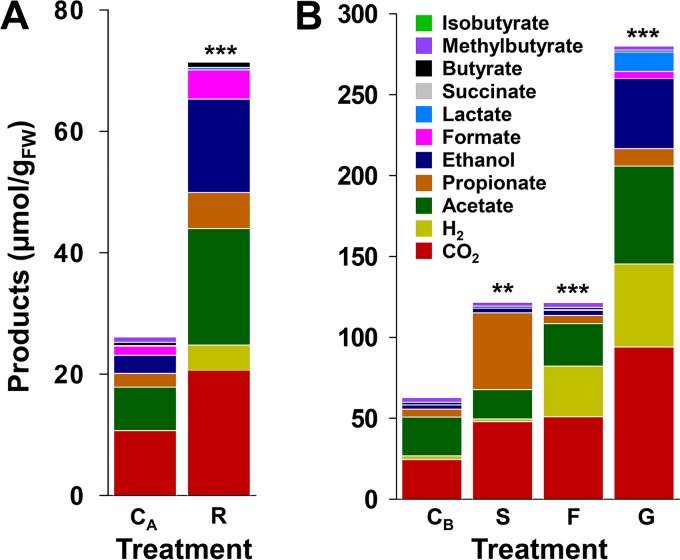
Collective amounts of fermentation products in ribose (A) and transient intermediate (B) treatments. Values are the averages from triplicate analyses shown in Table S6 in the supplemental material (ribose) and Fig. 5 (transient intermediates) and represent the net amounts of products at the end of the 30 h of incubation. The asterisks indicate significant differences between the collective amount of products formed in unsupplemented control and supplemented treatments. **, *P ≤ *0.01; ***, *P ≤ *0.001 by *t* test with unequal variances (see Table S7 for *P* values, mean values, and variances); C_A_ and C_B_, unsupplemented controls; R, ribose; S, succinate; F, formate; G, glucose; FW, fresh weight.

Ribose significantly enhanced the collective formation of fermentation products (Fig. 4A), and 82% and 87% of ribose-derived carbon and reducing equivalents, respectively, were recovered (Table 2). Propionate and H_2_ were significant products in the ribose treatment (Fig. 4A; see also Tables S6 and S7) and were detected in certain amino acid treatments (Fig. 2). The production of propionate and H_2_ can be coincident with the transient formation of succinate and formate, respectively, during gut content fermentation of protein, RNA, and cell lysate rich in protein and RNA (24, 40). These observations are indicative of the conversion of succinate to propionate via a decarboxylation pathway (66) and the consumption of formate by formate-hydrogen lyase (67, 68). However, these transformations of succinate and formate have not been demonstrated and were therefore evaluated.

**TABLE 2:** Estimated recoveries of carbon and reducing equivalents (i.e., electrons) in ribose, succinate, formate, and glucose treatments^*a*^

Product	Recovery (%)^*b*^							
	Ribose		Succinate		Formate		Glucose	
	Carbon	RE^*c*^	Carbon	RE	Carbon	RE	Carbon	RE
CO_2_	10	NA^*d*^	16	NA	52	NA	24	NA
H_2_	NA	2.1	NA	—^*e*^	NA	58	NA	8.4
Acetate	25	25	—	—	9	18	25	25
Ethanol	26	40	0.4	0.7	2.3	6.8	28	42
Succinate	2.5	2.2	—	—	—	—	—	—
Lactate	1.0	1.0	—	—	4.6	9.2	13	13
Formate	3.4	1.7	—	—	—	—	1.5	0.7
Propionate	11	13	86	114	0.8	2.0	6.1	7.2
Isobutyrate	—	—	—	—	0.7	1.7	—	—
Methylbutyrate	—	—	—	—	0.7	1.9	—	—
								
Total	82	87	102	115	71	98	98	96

a See Fig. 4 and Fig. 5 for product profiles. Net amounts of products formed in the unsupplemented control were subtracted from those of supplemented treatments.

b Recoveries are based on the amount of substrate consumed. Values are based on the arithmetic average from three replicate analyses.

c RE, reducing equivalents.

d NA, not applicable.

e —, no net increase of the product during the incubation in supplemented treatments relative to that in the control treatment.

Supplemental succinate and formate were subject to consumption (Fig. 5) and significantly enhanced the collective fermentation product profile (Fig. 4B; Table S7). The consumption of succinate was concomitant with the production of increased amounts of propionate and CO_2_, and the consumption of formate was concomitant with the production of increased amounts of H_2_ and CO_2_ (Fig. 5), product profiles consistent with the aforementioned transformations of succinate and formate. Furthermore, the control treatment also displayed a transient occurrence of succinate and concomitant accumulation of propionate. Likewise, the transient production of succinate and formate was concomitant with the formation of propionate and H_2_, respectively, during the fermentation of glucose, a potentially mucus-derived saccharide found in the alimentary canal (18, 19) (Fig. 5). These collective findings demonstrated that the secondary utilization of succinate and formate can contribute to the production of propionate and H_2_, respectively, during gut content fermentation.

**FIG 5:**
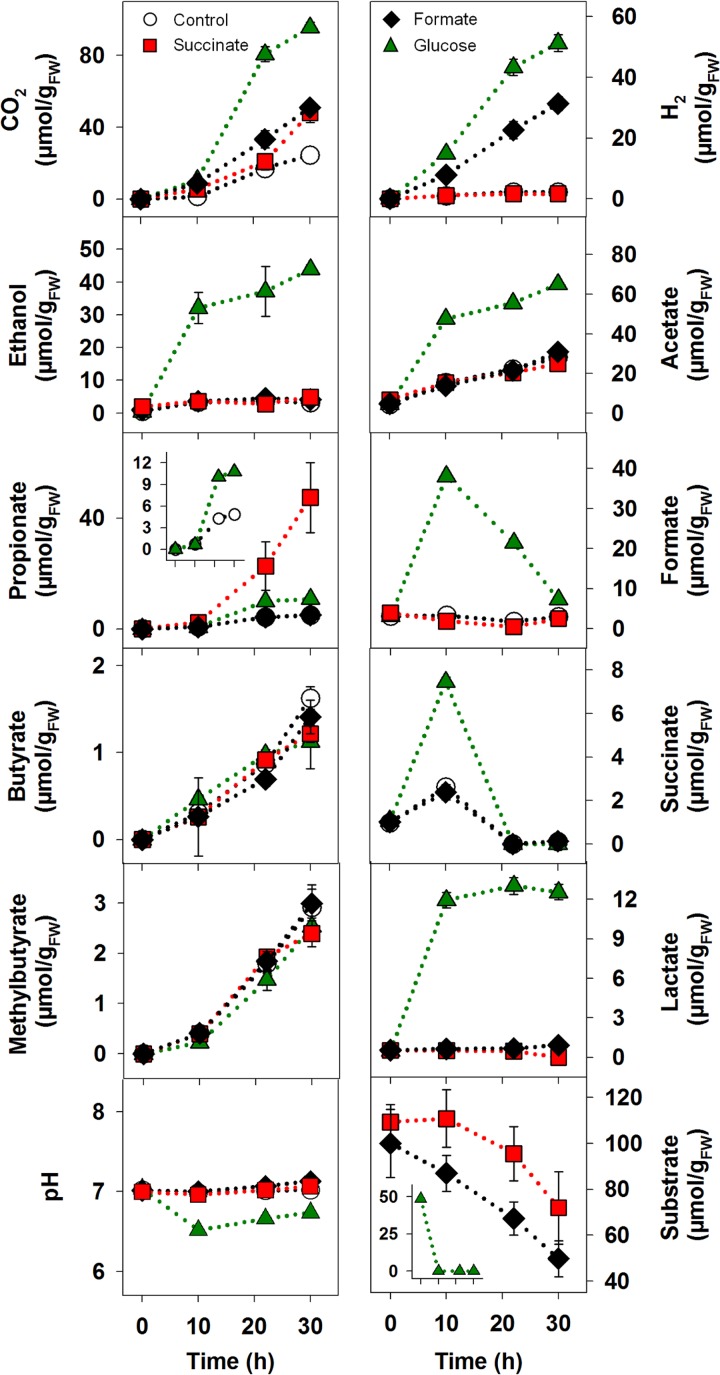
Effects of succinate, formate, and glucose on the fermentation product profiles of anoxic microcosms of *L. terrestris* gut contents. Initial concentrations approximated 10 mM for succinate and formate and 5 mM for glucose; the control lacked supplement. Values are the arithmetic averages from three replicate analyses, and error bars indicate the standard deviations. Some standard deviations are smaller than the size of the symbol and therefore not apparent. FW, fresh weight.

### Fermentative bacterial families stimulated by ribose and transient intermediates.

A total of 606,090 bacterial 16S rRNA and 16S rRNA gene sequences were obtained from the saccharide and transient intermediate treatments, yielding 25 phyla (including candidate phyla), and the rarefaction analyses indicated that the most abundant taxa were targeted (see Fig. S5 and S6). A net increase in the relative abundance of *Aeromonadaceae*-affiliated sequences in the ribose treatment indicated that ribose stimulated members of this family (Fig. 3B; see Fig. S7 and S8 and Table S8). The *Fusobacteriaceae* were mostly stimulated by succinate, whereas formate treatments yielded a net increase in the relative abundance of *Peptostreptococcaceae*-affiliated sequences (Fig. 3B). At the end of incubation, the relative abundances of 16S rRNA sequences affiliated to these families were significantly greater in supplemented treatments than in the unsupplemented control (Table S8). Consistent with the strong physiological response to glucose (Fig. 4B) and stimulation of the *Aeromonadaceae* in that treatment (Fig. 3B), the number of detected phylotypes, the number of expected phylotypes (Chao1), and Shannon indices of the glucose treatment were lower than those of unsupplemented control (see Table S9 and Fig. S6B). These findings indicated that shifts in community members occurred during the incubation, and NMDS analysis of all detected phylotypes (97% sequence similarity) confirmed that the microbial communities changed during the incubation in all treatments (see Fig. S3C to F).

## DISCUSSION

L. terrestris is a model anecic earthworm, feeding on diverse material and associated microorganisms that are subject to disruption during gut passage (6, 25, 26, 69, 70). In this regard, disrupted ingested biomass and gut mucus constitute sources of protein and RNA in the alimentary canal (35–39, 41, 43, 44, 71), and the responsiveness of gut fermenters to amino acids and ribose as model protein- and RNA-derived fermentable substrates, respectively (Fig. 2 and 4), is consistent with the availability of these biopolymers and the products of their hydrolysis in the gut.

### Responsive fermentative phylotypes.

**FIG 6:**
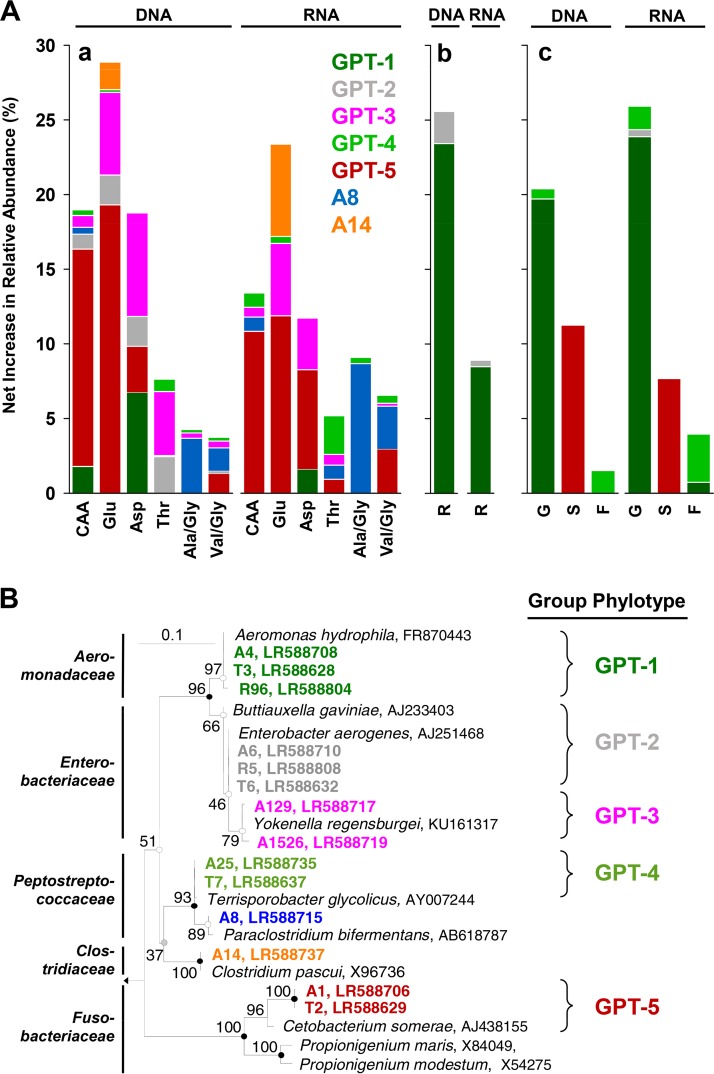
16S rRNA-based overview of the net increase of relative abundances of the main stimulated group phylotypes and phylogenetic tree (dendrogram) of these stimulated group phylotypes. (A) Each group phylotype (GPT) consists of identical or nearly identical phylotypes based on a ≥97% sequence similarity. Phylotypes are based on a sequence similarity cutoff of 97% and were considered stimulated when a phylotype in at least one of the supplemented treatments displayed a ≥2% net increase in relative abundance. Net increases of relative abundances were calculated as follows (8): (i) the calculation is based either on mean relative abundances when samples from the three replicates were analyzed separately (i.e., all RNA and DNA samples of control treatments and RNA samples at 30 h of supplemented treatments) or on single relative abundances when samples of the three replicates were pooled for sequence analyses (i.e., DNA samples at 0 h and 30 h and RNA samples at 0 h of supplemented treatments); (ii) mean or single relative abundances at the beginning of incubation were subtracted from those at the end of incubation for control and supplemented treatments; (iii) the resulting time-corrected relative abundances of control treatments were subtracted from those of supplemented treatments (negative time-corrected relative abundances of control treatments were ignored). CAA, Casamino Acids; Glu, glutamate; Asp, aspartate; Thr, threonine; Ala, alanine; Gly, glycine; Val, valine; S, succinate; F, formate; G, glucose. (B) The phylogenetic tree was calculated using the neighbor-joining, maximum parsimony, and maximum likelihood methods. Solid circles, congruent nodes in three trees; empty circles, congruent nodes in maximum parsimony and maximum likelihood trees; gray circles, congruent nodes in maximum parsimony and neighbor-joining trees. Branch length and bootstrap values (1,000 resamplings) are from the maximum parsimony tree. The bar indicates 0.1 changes per nucleotide. Thermotoga maritima (AE000512) was used as an outgroup. Accession numbers are shown at the end of each branch. Phylotype descriptors: A, phylotypes derived from amino acid experiment (Fig. 1); R, phylotypes derived from ribose experiment (Fig. 4A); T, phylotypes derived from transient experiment (Fig. 4B).

A previous study demonstrated that *Firmicutes*- and *Fusobacteria*-affiliated obligate anaerobes were responsive during the fermentation of protein and that the fermentation of RNA was linked to responsive *Proteobacteria*-affiliated facultative aerobes (40). In the present study, numerous responsive phylotypes, including five group phylotypes (GPT), were also affiliated to these families (Fig. 6; see also Table S10 in the supplemental material) (note, a group phylotype consists of identical or nearly identical phylotypes based on sequence similarity [8]). The relatively short read lengths generated by Illumina sequencing can compromise the taxonomic assignment of sequences at the species level (72, 73), and Illumina phylotype assessments should be qualified accordingly. In addition, the efficiency of a primer-dependent detection of a phylotype is influenced by the quantity of the target sequence. In this regard, the number of genomic 16S rRNA genes is variable (74) but very low compared to the high number of cellular ribosomes (and thus the number of 16S rRNA molecules), which can exceed 10^4^ per cell (75), suggesting that microbial cells might be more detectable with a 16S rRNA-based analysis. Within the constraints of these considerations, the fermentative activities of the detected phylotypes were relatively consistent with the phenotypic properties of the most closely related described species (Table 3).

**TABLE 3:** Description of main stimulated phylotypes and group phylotypes as shown in Fig. 6^*a*^

GPT^*b*^	PT^*c*^	Description
GPT-1	A4, T3, R96	Group phylotype GPT-1 (99% to 100% identity to Aeromonas hydrophila) was significantly stimulated by ribose and aspartate (Fig. 6). The facultative aerobe *A. hydrophila* ferments pentoses to acetate, ethanol, lactate, succinate, formate, CO_2_, and H_2_ (100–104). Consistent with its response to ribose, this fermentative phylotype was shown previously to respond to RNA and RNA-rich cell lysate (40). Although *A. hydrophila* is not known to ferment aspartate, it and closely related Aeromonas media harbor (i) aspartate ammonia lyase that transforms aspartate into the electron acceptor fumarate which reductively forms succinate (105, 106) and (ii) aspartate carbamoyltransferase that is utilized in the synthesis of pyrimidine precursors (107). Group phylotype GPT-1 was also responsive to glucose, a finding consistent with its responsiveness to diverse polymeric and nonpolymeric saccharides (8, 108).
GPT-2	A6, R5, T6	Based on 16S rRNA gene sequences, the *Enterobacteriaceae*-affiliated group phylotype GPT-2 (99% to 100% identity to the facultative aerobes Buttiauxella gaviniae and Enterobacter aerogenes) displayed a broad response in glutamate, aspartate, threonine, Casamino Acids, ribose, and formate treatments (Fig. 6). *B. gaviniae* produces fatty acids and gases when fermenting sugars such as ribose, and several *Buttiauxella*-associated species can utilize amino acids, including glutamate, aspartate, and threonine as sole carbon and energy sources (109). The *Buttiauxella*- and *Enterobacter*-affiliated phylotypes were also stimulated in gut contents supplemented with RNA or cell lysate (40).
GPT-3	A129, A1526	Sequences of the *Yokenella*-affiliated group phylotype GPT-3 (97% to 99% identity to the facultative aerobe Yokenella regensburgei) displayed an apparent net increase in relative abundance in glutamate, aspartate, and threonine treatments. We are unaware of information on the ability of *Y. regensburgei* to ferment amino acids, but its occurrence in human wounds and infection is suggestive of its potential ability to use amino acids (110, 111).
GPT-4	A25, T7	The group phylotype GPT-4 (99% to 100% identity to Terrisporobacter glycolicus) was stimulated in threonine and formate treatments (Fig. 6). This is consistent with (i) the ability of *T. glycolicus* to convert threonine to propionate (112) and (ii) the potential for this acetogen to from acetate from formate (113). Acetogen-affiliated phylotypes also responded positively in cell lysate treatments that produced large amounts of transient formate (40). Acetogens are capable of diverse dissimilatory processes, including fermentation (114, 115); thus, the stimulation of a potential acetogen is not strictly dependent on acetogenesis.
GPT-5	A1, T2	The *Fusobacteriaceae* were represented by group phylotype GPT-5 (96% identity to Cetobacterium somerae), which was responsive in the glutamate, aspartate, valine/glycine, and Casamino Acids treatments (Fig. 6), findings consistent with this group phylotype being strongly stimulated by protein (40). Although a 96% sequence identity is relatively low for species-level classification, *C. somerae* occurs in gastrointestinal systems and ferments amino acids and peptides to acetate, propionate, and butyrate, products detected in the aforementioned amino acid treatments (116, 117). Group phylotype GPT-5 was more distantly related to species of the strictly anaerobic genus *Propionigenium* that are able to utilize succinate for growth and produce propionate (66, 118), properties consistent with the product profile of the succinate treatment (Fig. 4) in which this group phylotype was also responsive (Fig. 6).
	A8	*Peptostreptococcaceae*-affiliated phylotype A8 (99% identity to the amino acid fermenter Paraclostridium bifermentans) responded in the co-amino acid treatments (Fig. 6), which was indicative of Stickland fermentation (60). In this regard, *P. bifermentans* isolated from the human gut can be cultivated on co-amino acids such as the alanine/glycine treatment utilized in the present study (53), which is consistent with phylotype A8 facilitating Stickland fermentation. Phylotype A8 was also weakly responsive in the Casamino Acids treatment (Fig. 6), and *P. bifermentans*-affiliated phylotypes are also strongly stimulated by protein and cell lysate (40), activities consistent with the ability of *P. bifermentans* to ferment numerous amino acids (53).
	A14	Glutamate-stimulated phylotype A14 (Fig. 6) was closely related to Clostridium pascui (100% identity), a proteolytic spore-forming anaerobe that ferments glutamate (119). This phylotype is also stimulated by protein-rich cell lysate (40), reinforcing the likelihood that this phylotype can ferment certain amino acids.

a See Table S10 in the supplemental material for statistical analyses of the phylotypes.

b GPT, group phylotype.

c PT, stimulated phylotype.

Because responsive gut fermenters are dominated by ingested transients (24), the potential for a fermentative response is dependent on the occurrence of a given fermentative phylotype in preingested soil. For example, the *Fusobacteriaceae* in gut content is responsive in some cases but in other cases is essentially nondetectable, thus reflecting the variable detectability of this family in the soil on which an earthworm is maintained (8, 24, 40). In the current study, the *Fusobacteriaceae* were represented by GPT-5 which was responsive to amino acids (Fig. 6), a finding consistent with (i) the stimulation of this phylotype during the fermentation of protein (40) and (ii) its most closely related species being capable of fermenting amino acids (Table 3).

The analysis of the phenotypic properties of a responsive phylotype was based on its apparent ability to fermentatively dissimilate an amino acid (Table 3). However, amino acids could have also stimulated phylotypes via assimilatory processes. Furthermore, the taxonomic assessments were restricted to bacteria (Fig. 6 and Table 3), but soil also contains fermentative fungi (76–78) that may have contributed to fermentation. Indeed, the capacity of fungi to produce diverse hydrolases (79, 80) suggests that ingested fungi contribute to microbial processes in the alimentary canal.

### Conclusions and perspectives.

**FIG 7:**
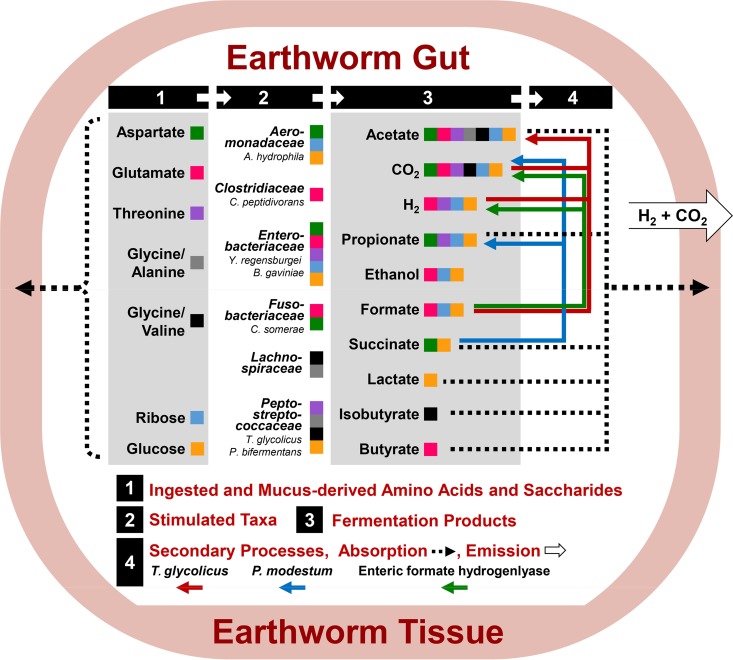
Hypothetical model of fermentative transformations of amino acids and saccharides in the gut of *L. terrestris*. The model depicts events that are interfaced to (i) the *in situ* hydrolysis of dietary protein, dietary RNA, and glycoprotein-rich mucus, and (ii) the earthworm’s utilization of biopolymer constituents and fermentation-derived products.

The present study indicated that (i) the fermentation of certain amino acids was associated with fermentative subsets of contrasting gut-associated *Firmicutes*-, *Proteobacteria*-, and *Fusobacteria*-affiliated taxa, (ii) ribose stimulated fermentative *Proteobacteria*-affiliated taxa, and (iii) the transient products succinate and formate were subject to secondary processes associated with *Firmicutes*- and *Fusobacteria*-affiliated taxa (Fig. 7). The experimental protocol was designed to detect the responsiveness of fermentative taxa to a supplemental substrate, and the strong enhancement of a given phylotype is not proposed to occur *in situ*. However, as a proof of principle, the findings conceptualized in the model (Fig. 7) illustrate that fermentative bacteria in the alimentary canal of *L. terrestris* are poised to respond to specific products of the hydrolysis of protein and RNA, verifying that these biopolymers are fermentable in the gut (40).

Several of the common amino acids that occur in the gut (7) were not evaluated but would be subject to fermentation *in situ*. For example, isoleucine can represent approximately 10% of the amino acids in the earthworm gut (7) and can be fermented to methylbutyrate (60), a potential corroborated by the formation of methylbutyrate in the Casamino Acids treatment (Fig. 1). Within the constraints of these considerations and in support of the interactions depicted in the model (Fig. 7), the phylotype-affiliated taxa facilitate fermentations indicative of those detected in the different treatments (Table 3), and the diverse products formed in response to amino acids, ribose, and transient intermediates are consistent with those found in the alimentary canal (7, 18). To simplify the taxonomic analyses, only the most abundant highly responsive phylotypes were evaluated, but less abundant or slower responding phylotypes likely contributed to the fermentations and might be of relevance *in situ*.

It is assumed that gut bacteria and the animal host compete for amino acids (Fig. 7); however, the degree to which that occurs is unknown. In the human colon, bacterial fermentation of amino acids goes unchallenged because the colon cannot take up amino acids (49). Fermentation-derived fatty acids in the alimentary canal can be either dissimilated or assimilated by earthworms (Fig. 7) (18, 19, 62, 63). Animal-microbe fermentative interactions have been extensively characterized in more advanced biopolymer-degrading gut ecosystems that compartmentalize highly diverse host-associated syntrophic species (e.g., termites and ruminants [23, 81]). The more primitive earthworm gut illustrates the competitive and beneficial interactions that can occur between the animal host and the transiently hosted fermenter (Fig. 7). In this regard, although the alimentary canal of the earthworm might be considered relatively simple, the microbial properties of the matter that passes through it are not. Soil is one of the most complex microbial habitats, with a gram (dry weight) of ingested soil having up to approximately 10^10^ microbial cells that have enormous phylogenic diversity (82). Furthermore, the cultivable number of microbes capable of anaerobic growth in soil can range from 10^7^ to 10^9^ per gram dry weight soil (83, 84), illustrating the large potential of ingested material to facilitate anaerobic processes in the anoxic gut.

Glycoprotein-rich gut mucus (41, 42) provides fermentable amino acids and saccharides for ingested microbes (Fig. 7). While it is advantageous for earthworms to utilize mucus-derived fermentation products (Fig. 7), such recycling of worm-derived organic carbon cannot explain how earthworms perpetuate. Ultimately, ingested nutrients including biopolymers must be utilized. In this regard, microbe- and plant-derived organic carbon rapidly stimulate fermentation by gut bacteria (8, 40), and dietary polymers that are more easily hydrolyzed are likely primary sources of fermentable organic carbon. For example, structural polysaccharides that are difficult to hydrolyze (e.g., cellulose and chitin) are not readily utilized for gut content fermentation, whereas protein and nonstructural polysaccharides (e.g., starch and glycogen) are easily hydrolyzed and rapidly fermented by the gut community (8, 40). Thus, the fermentative capacity of the gut community to convert ingested biomass to products that can be utilized by the earthworm can contribute to the sustenance of the animal host.

The fermentative transformation of protein and RNA in the anoxic gut of earthworms is clearly not unique to these invertebrates; these biopolymers are subject to fermentative degradation in all O_2_-limited environments. However, the ecosystem- and microbiome-level consequences of the fermentative transformations of these biopolymers have not been as intensively investigated as those of plant biopolymers such as cellulose (85, 86). At the global level, the capacity of prokaryotes to synthesize protein- and RNA-rich biomass may be similar to the capacity of plants to synthesize biomass rich in polysaccharides, with the productivity of both autotrophic and heterotrophic microbes being important (87–92). These considerations illustrate the enormous global capacity of microbes to synthesize protein and RNA. In this regard, the evolution of life is believed to have started approximately 4 billion years ago under anoxic conditions, and the existence of plants that ultimately became major producers of polysaccharides is thought to have occurred approximately 1 billion years ago (93). As such, and on the assumption that protein and RNA were dominant polymers of primordial microbial cells, it seems likely that microbe-derived protein and RNA were early drivers of fermentation and other redox processes when the planet was O_2_ free and obligate anaerobes dominated. The biological potential to fermentatively profit from these microbial biopolymers is exemplified in the primitive gut ecosystem of earthworms (Fig. 7), and resolving the fermentative transformations of these biopolymers in diverse anoxic environments would increase our understanding of how they contribute to the anaerobic turnover of organic carbon in today’s biosphere.

## MATERIALS AND METHODS

### Earthworms and soil.

*L. terrestris* specimens from Fischerkönig Angelgeräte (Neustadt/Orla, Germany) were purchased from Fisherman’s World (Bayreuth, Germany) and maintained in loamy soil supplemented on the top with turf (which contained soil, roots, grass, and leaves) for approximately ten days prior to use. Soil and turf were collected from the meadow Trafo Wiese in Bayreuth.

### Stock solutions.

Stock solutions of Casamino Acids (Difco Laboratories, Detroit, MI), alanine (Merck, Darmstadt, Germany), aspartate (Merck), glutamate (Merck), glycine (Sigma-Aldrich, Taufkirchen, Germany), leucine (AppliChem, Darmstadt, Germany), threonine (Merck), tyrosine (Merck), valine (Merck), ribose (Sigma-Aldrich), formate (Sigma-Aldrich), succinate (Sigma-Aldrich), and glucose (AppliChem) were prepared with anoxic sodium phosphate buffer (36 mM, pH 7 [pH was adjusted with NaOH]). Solutions were filter sterilized (0.22-μm pore size, cellulose-acetate membrane) into sterile anoxic 100-ml serum vials that were crimp sealed with sterile butyl-rubber stoppers (Glasgerätebau Ochs Laborfachhandel e.K., Bovenden, Germany [product number 102049]); the vials were then flushed 10 min with sterile argon (100%).

### Anoxic gut content microcosms.

Gut contents were extracted and pooled in an O_2_-free chamber (100% N_2_ [Mecaplex, Grenchen, Switzerland]) as described previously (40). Each microcosm constituted a 10-ml slurry consisting of 1 g fresh weight gut content, sodium phosphate buffer (36 mM, pH 7), and stock solution in a 27-ml sterile glass crimp-seal tube. Gut content and buffer were added to sterile tubes in an O_2_-free chamber; the tubes were then closed with sterile butyl-rubber stoppers, crimp sealed, removed from the chamber, and flushed 10 min with sterile N_2_. Stock solutions were added using sterile N_2_-flushed syringes, yielding a total volume of 10 ml. Tubes were pressurized to 60 kPa with sterile N_2_. Control treatments lacked supplement. As in previous studies (8, 24, 40), incubation was in the dark at room temperature (21 to 24°C) for 30 h, a time that will likely capture potential activities that could occur during gut passage that can be up to 24 h (46, 94, 95). Sampling of gas and liquid phases was with sterile syringes.

### Chemical and statistical analyses.

Parameters for gas chromatography, high-performance liquid chromatography (HPLC), and pH measurements were as described previously (40). For the HPLC analyses, a 50-μl injection volume was used with a 1200 Series HPLC instrument (Agilent Technologies, Wilmington, DE, USA). Amounts of H_2_ and CO_2_ in the gas and liquid phases were calculated from the ideal gas law and standard solubility tables (96); for CO_2_, amounts of bicarbonate (calculated from dissolved CO_2_, pH, and the dissociation constant) were taken into consideration. For converting amounts of a product from micromoles per gram fresh weight (as used throughout the presentation) to millimolar or micromoles per gram dry weight, values were multiplied by 0.1 (e.g., 100 μmol per g fresh weight equals 10 mM) or divided by 0.45 (e.g., 100 μmol per g fresh weight equals 222 μmol per g dry weight), respectively.

Ammonium was measured with a modified published protocol (97) utilizing 96-well multitest plates (neoLab, Heidelberg, Germany). Per well, a 100-μl sample was mixed with 50 μl of 2% sodium phenolate (Merck), 25 μl of 0.005% sodium nitroprusside (Merck), and 25 μl of sodium hypochlorite solution. The sodium hypochlorite solution consisted of 25 ml sodium hypochlorite containing 12% Cl (Roth, Karlsruhe, Germany) and 1.125 g NaOH (Roth) that was then adjusted to 250 ml with deionized water. After a 30-min incubation in the dark at 30°C, absorbance at 630 nM was measured with a μQuant spectrophotometer (BioTek Instruments GmbH, Bad Friedrichshall, Germany).

Theoretical recoveries of carbon and reducing equivalents (i.e., electrons) were calculated as described previously (40). Calculations of recoveries of reducing equivalents were based on 4.2 electrons per carbon atom for Casamino Acids, 4.8 electrons per carbon atom for valine, 3.6 electrons per carbon atom for glutamate, 3.0 electrons per carbon atom for aspartate and glycine, 4.0 electrons per carbon atom for threonine, alanine, ribose, and glucose, 3.5 electrons per carbon atom for succinate, and 2.0 electrons per carbon atom for formate.

The unequal variance *t* test for calculating *P* values of fermentation products was used as described previously (24). Linear discriminant analysis effect size (LEfSe) (98) was used to (i) evaluate the significance (Kruskal-Wallis test) of taxa responding to the different treatments and (ii) rank significant taxa according to the effect sizes using linear discriminant analysis (LDA) (24). Nonmetric multidimensional scaling (NMDS) based on the Bray-Curtis dissimilarity matrices was conducted with the software Past 3 (99) to evaluate dissimilarities of the microbial communities in different treatments.

### Molecular analyses.

Extraction of nucleic acids and synthesis of cDNA were as described previously (40). PCR amplification, Illumina MiSeq sequencing, and amplicon-metagenomics data processing performed by Microsynth AG (Balgach, Switzerland) were as described previously (24). Rarefaction curves were calculated with aRarefact (http://www.uga.edu/strata/software/). Phylogenetic trees were calculated with ARB (120) using representative sequences of the most abundant operational taxonomic units (OTUs; phylotypes) and closely affiliated reference sequences.

### Sequence abundances.

The relative abundances of all sequences, including less abundant sequences not highlighted in Results, are provided in Table S11 (amino acids), Table S12 (ribose), and Table S13 (transient intermediates) in the supplemental material.

### Accession number(s).

Sequences were deposited at the European Nucleotide Archive (ENA) under study numbers PRJEB32428, PRJEB32430, and PRJEB32429 for the amino acid, ribose, and transient intermediate experiments, respectively. Representative sequences of phylotypes with ≥0.1% relative abundance were deposited under the accession numbers LR588706 to LR588802, LR588803 to LR588886, and LR588628 to LR588705 for the amino acid, ribose, and transient intermediate experiments, respectively.

## Supplementary Material

Supplemental file 1
