# Supplementary material for: Amino Acids and Ribose: Drivers of Protein and RNA Fermentation by Ingested Bacteria of a Primitive Gut Ecosystem
Source: Appl Environ Microbiol. 2019 Sep 17;85(19):e01297-19. doi: 10.1128/AEM.01297-19 (PMC6752017; doi:10.1128/AEM.01297-19)
Supplement: Supplemental file 1 [file AEM.01297-19-s0001.pdf]

Supplemental Material

Amino Acids and Ribose: Drivers of Protein and RNA Fermentation by Ingested Bacteria of a Primitive Gut Ecosystem

Lydia Zeibich, Maraike Staege, Oliver Schmidt, and Harold L. Drake*

Department of Ecological Microbiology, University of Bayreuth, 95440 Bayreuth, Germany

*Corresponding author: Harold L. Drake, Department of Ecological Microbiology, University of Bayreuth, 95440 Bayreuth, Germany. Tel: (+49) (0)921-555640. Fax: (+49) (0)921-555793. E-Mail: HLD@Uni-Bayreuth.De

List of Supplemental Figures and Tables

Figures

Fig. S1. Collective amounts of fermentation products in amino acid treatments of the preliminary study.

Fig. S2. Rarefaction analyses of bacterial 16S rRNA genes (A) and 16S rRNA (B) sequences obtained from gut content microcosms supplemented with amino acids.

Fig. S3. Two-dimensional non-metric multidimensional scaling (NMDS) plot of the microbial community composition in amino acid (A, B), ribose (C, D), and succinate, formate, and glucose (E, F) treatments.

Fig. S4. 16S rRNA and 16S rRNA gene analyses of control and amino acid treatments.

Fig. S5 . Rarefaction analyses of bacterial 16S rRNA and 16S rRNA gene sequences obtained from gut content microcosms supplemented with ribose.

Fig. S6. Rarefaction analyses of bacterial 16S rRNA genes (A) and 16S rRNA (B) sequences obtained from content microcosms supplemented with succinate, formate, and glucose.

Fig. S7. 16S rRNA and 16S rRNA gene analyses of control and ribose treatments.

Fig. S8. 16S rRNA (RNA) and 16S rRNA gene (DNA) analyses of control, succinate, formate, and glucose treatments.

Tables

Table S1. Effect of amino acids on the fermentation product profiles of anoxic microcosms of *L. terrestris* gut contents.

Table S2. *P* values of fermentation products in amino acid treatments.

Table S3. Production of ammonium in amino acid treatments.

Table S4. Statistical analyses of stimulated families in amino acid treatments.

Table S5. Alpha diversity of the microbial community in control and amino acid treatments.

Table S6. Effect of ribose on the fermentation product profiles of anoxic microcosms of *L. terrestris* gut contents.

Table S7. *P* values of fermentation products in ribose, succinate, formate, and glucose treatments.

Table S8. Statistical analyses of stimulated families in ribose, glucose and transient intermediate treatments.

Table S9 Alpha diversity of the microbial community in control, ribose, succinate, formate and glucose treatments

Table S10. Statistical analyses of main stimulated phylotypes displayed in Fig. 6.

Table S11. Summary of all detected families in control and amino acid treatments based on 16S rRNA gene (A) and 16S rRNA (B) analysis.

Table S12. Summary of all detected families in control and ribose treatments based on 16S rRNA gene (A) and 16S rRNA (B) analysis

Table S13. Summary of all detected families in control, glucose, formate, and succinate treatments based on 16S rRNA gene (A) and 16S rRNA (B) analysis.

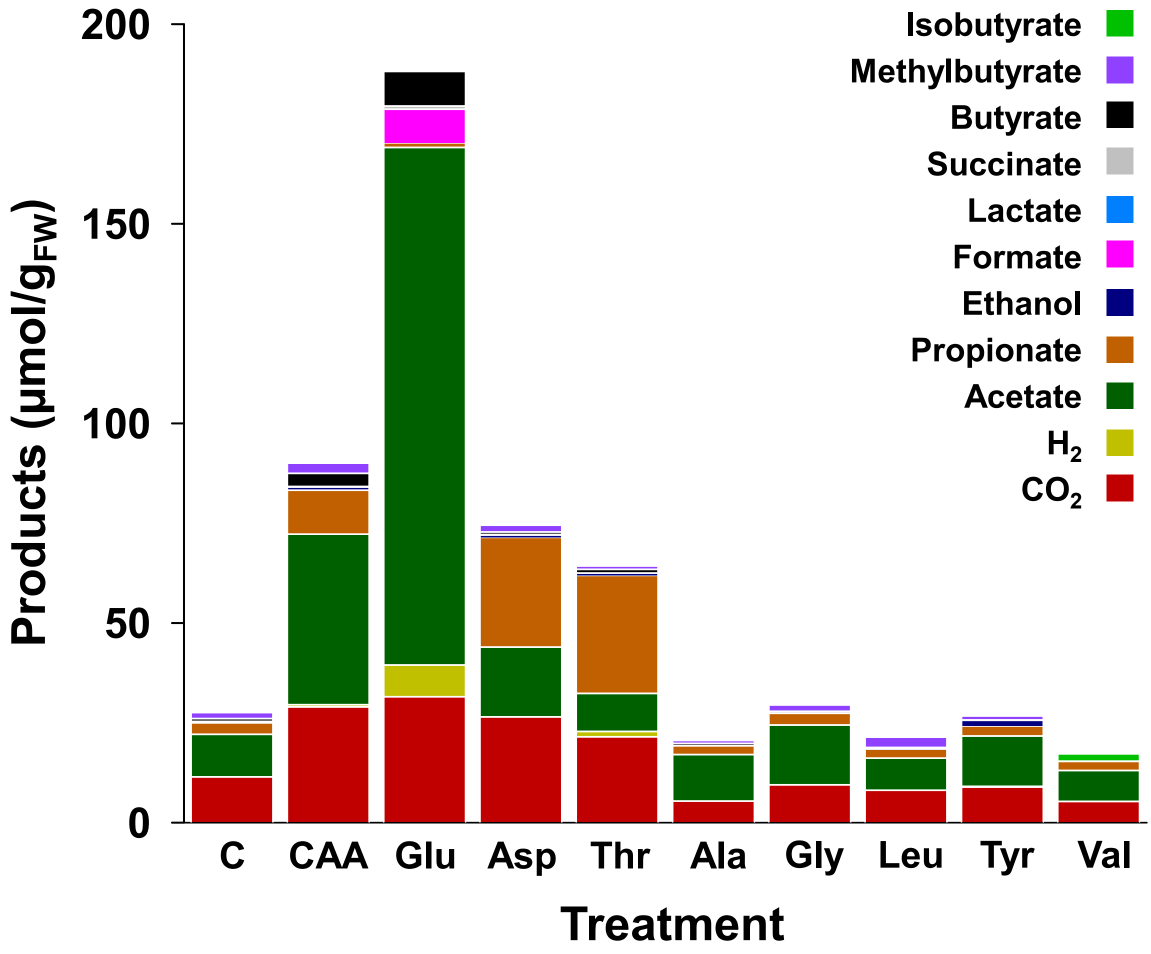

Fig. S1. Collective amounts of fermentation products in amino acid treatments of the preliminary study. Initial amino acid concentrations approximated 10 mM; the control lacked supplement. Values are the average of duplicate analyses shown in Table S1 and represent the net amounts of products at the end of the 30 h incubation. Abbreviations: C, unsupplemented control; CAA, casamino acids; Glu, glutamate; Asp, aspartate; Thr, threonine; Ala, alanine; Gly, glycine; Leu, Leucine; Tyr, Tyrosine; Val, valine; FW, fresh weight.

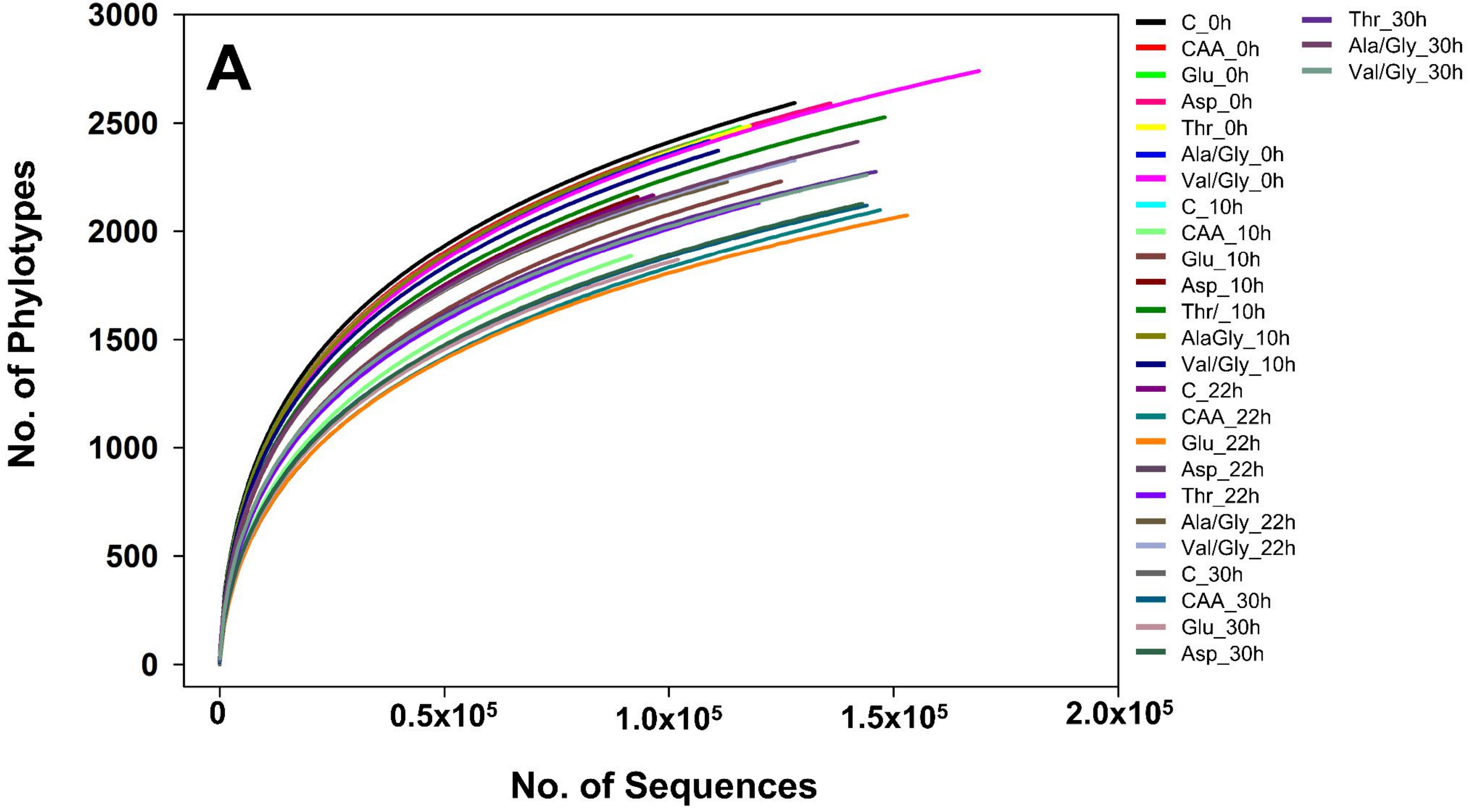
A
3000
2500
2000
1500
1000
500
0
No. of Phylotypes
0
0.5x10^5
1.0x10^5
1.5x10^5
2.0x10^5
No. of Sequences
C_0h
CAA_0h
Glu_0h
Asp_0h
Thr_0h
Ala/Gly_0h
Val/Gly_0h
C_10h
CAA_10h
Glu_10h
Asp_10h
Thr/_10h
AlaGly_10h
Val/Gly_10h
C_22h
CAA_22h
Glu_22h
Asp_22h
Thr_22h
Ala/Gly_22h
Val/Gly_22h
C_30h
CAA_30h
Glu_30h
Asp_30h
Thr_30h
Ala/Gly_30h
Val/Gly_30h

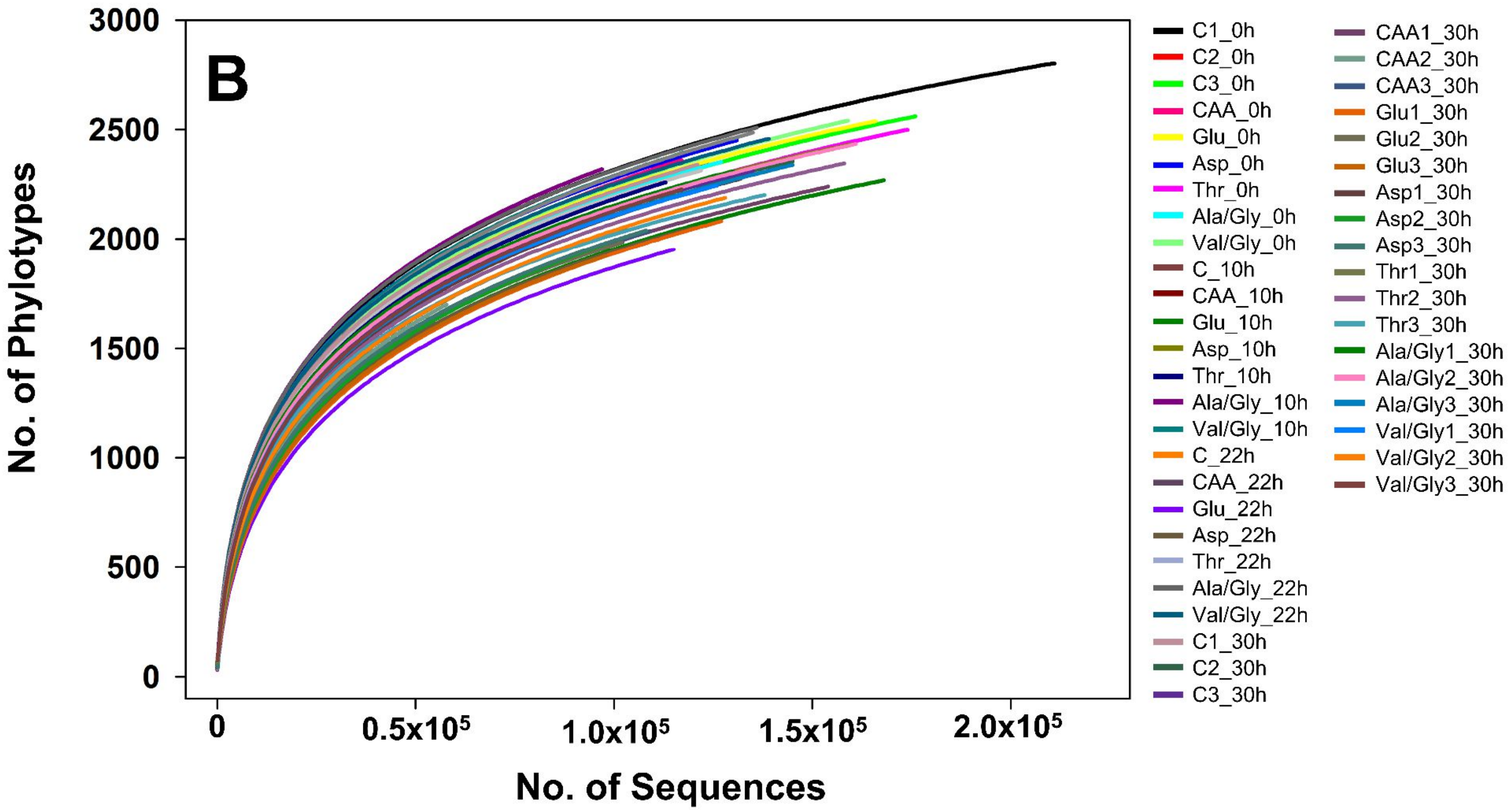

Fig. S2. Rarefaction analyses of bacterial 16S rRNA genes (A) and 16S rRNA (B) sequences obtained from gut content microcosms supplemented with amino acids. Phylotypes were based on a 97% sequence similarity cutoff. Samples of the three replicates of the 16S rRNA control treatment at 0 h, and all 16S rRNA treatments at 30 h were analyzed separately. Samples of the three replicates were pooled for each of the other treatments at 0 h, 10 h, 22 h, or 30 h. Abbreviations: 0h, 10h, 22h, and 30h indicate the time of sampling in hours; C, unsupplemented control; CAA, casamino acids; Glu, glutamate; Asp, aspartate; Thr, threonine; Ala, alanine; Gly, glycine; Val, valine. Identification numbers (e.g., C1) indicate the respective replicates.

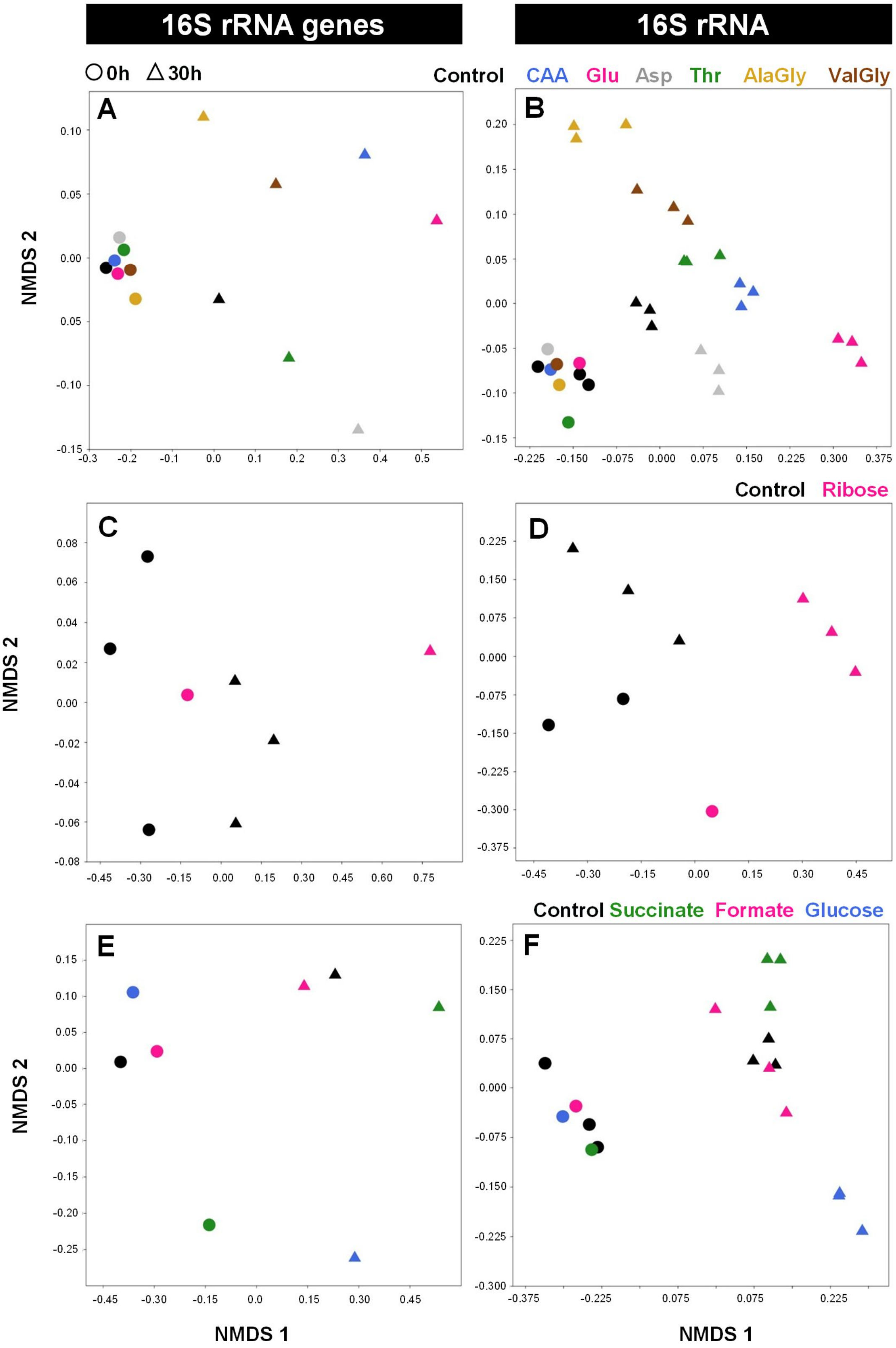

Fig. S3. Two-dimensional non-metric multidimensional scaling (NMDS) plot of the microbial community composition in amino acid (A, B), ribose (C, D), and succinate, formate, and glucose (E, F) treatments. Distance matrices (Bray-Curtis) are based on the relative abundances of all detected phylotypes in the different treatments (Table S11-S13). The origin of the sequence data in this figure is based on the sequence data illustrated in the rarefaction analyses (Figures S2, S5, and S6), and, as outlined in the legends of those figures, the replicated RNA analyses are based on each replicate treatment being sequenced. Proximity of symbols represent the degree of similarity between the different treatments. Abbreviations: C, unsupplemented control; CAA, casamino acids; Glu, glutamate; Asp, aspartate; Thr, threonine; Ala, alanine; Gly, glycine; Val, valine.

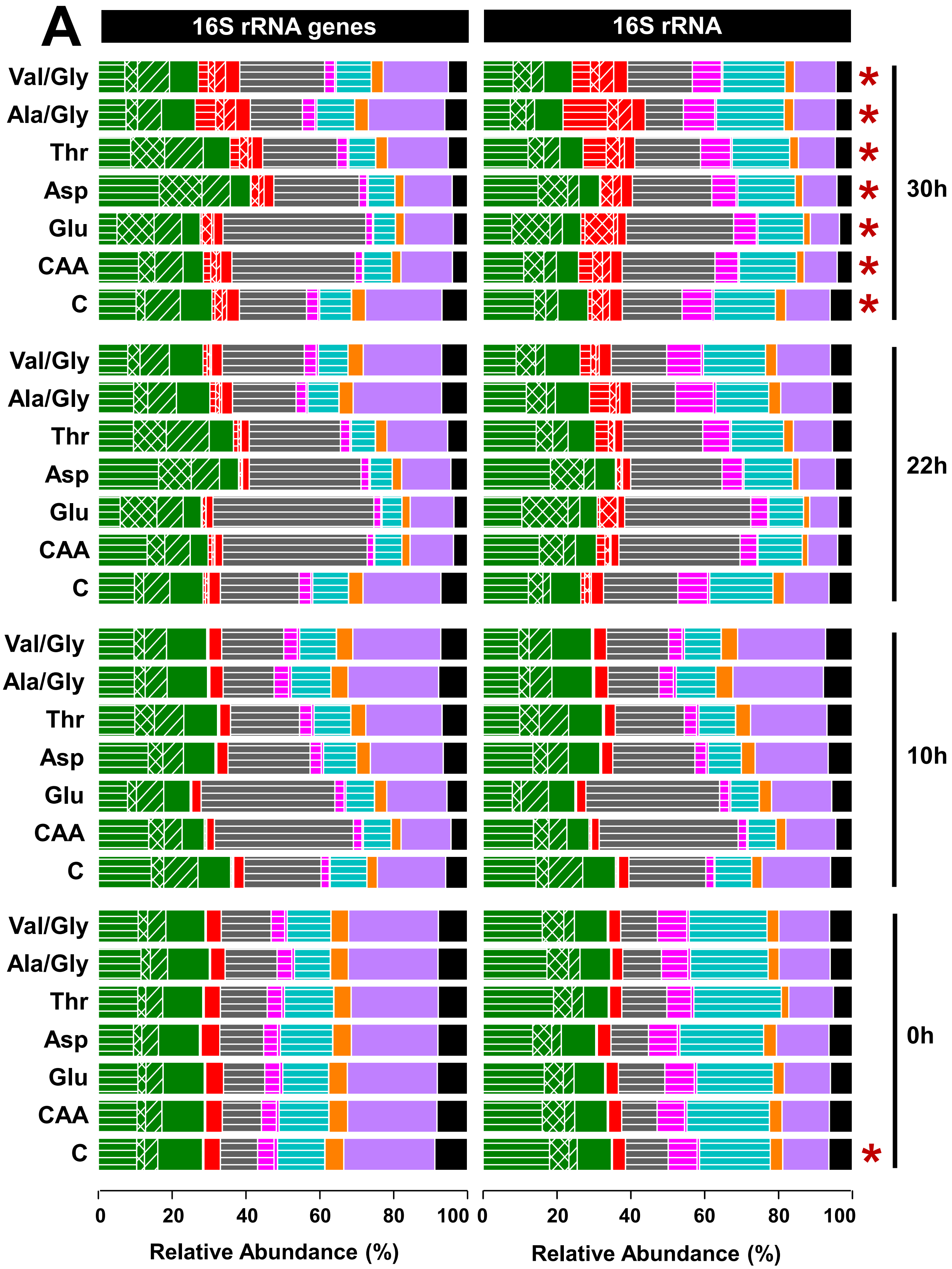
A
16S rRNA genes
16S rRNA
Val/Gly
Ala/Gly
Thr
Asp
Glu
CAA
C
30h
22h
10h
0h
0
20
40
60
80
100
Relative Abundance (%)
Relative Abundance (%)

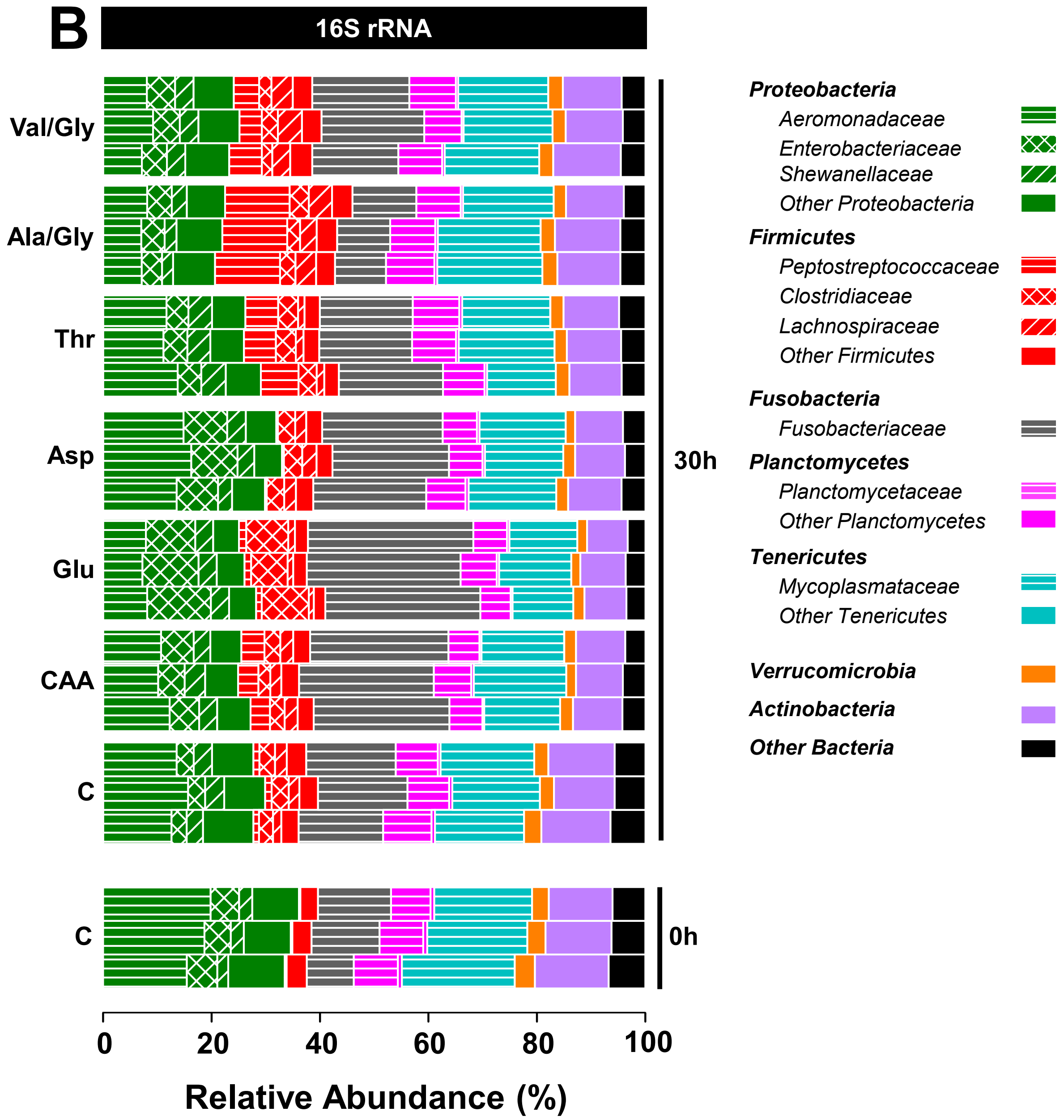

Fig. S4. 16S rRNA gene and 16S rRNA analyses of control and amino acid treatments. The most abundant families (i.e., families with ≥ 4% relative abundance in at least one sampling period) are displayed in the color of the respective phylum. Process data are shown in Fig. 1, and information on all detected taxa is provided in Table S11. Abbreviations: C, unsupplemented control; CAA, casamino acids; Glu, glutamate; Asp, aspartate; Thr, threonine; Ala, alanine; Gly, glycine; Val, valine. Panel A: Single bars without asterisk indicate that 16S rRNA gene or 16S rRNA samples of the three replicates were pooled for the sequence analysis. Asterisk indicates analysis was performed individually for the three replicates (see grouped bars Panel B).

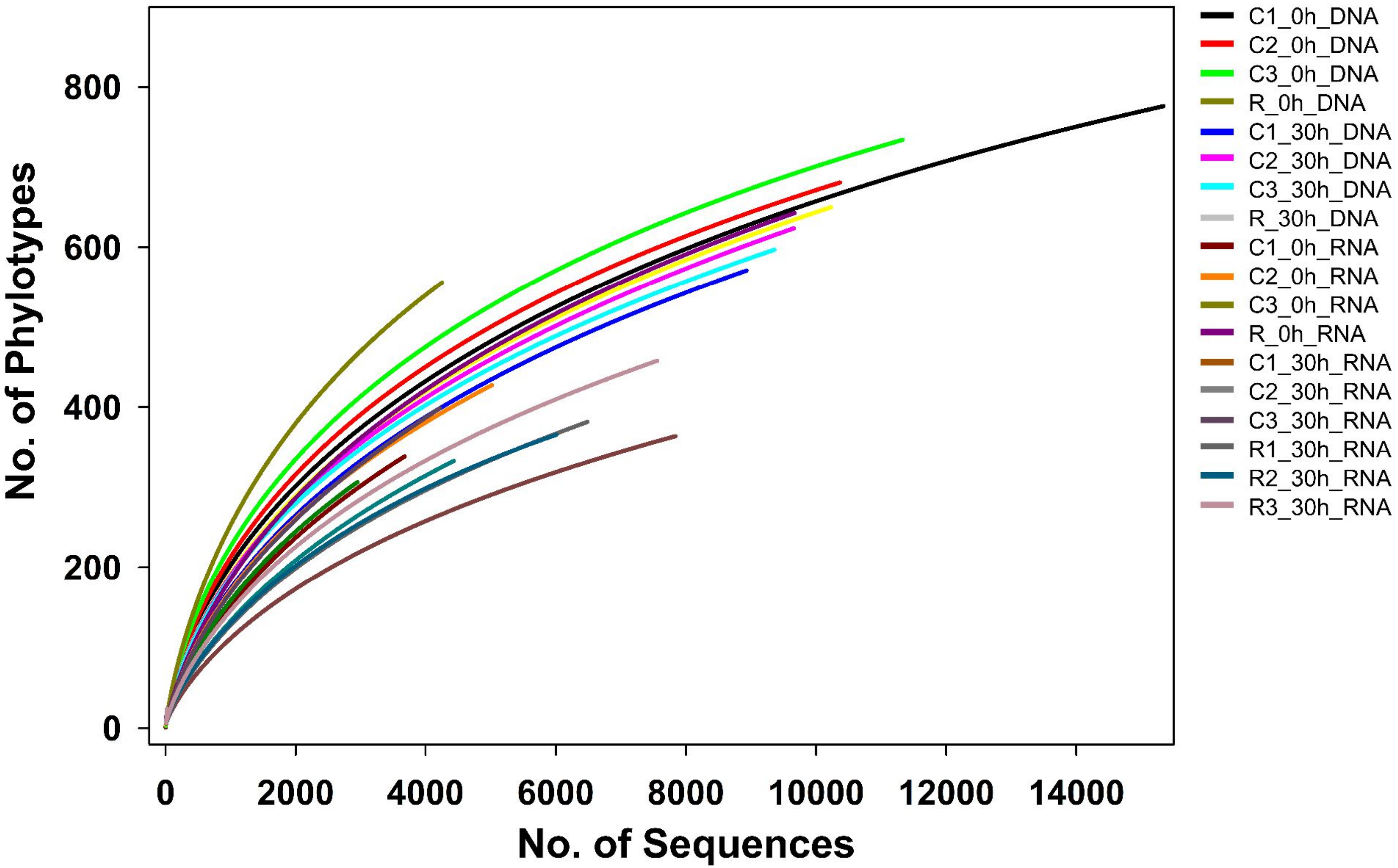

Fig. S5. Rarefaction analyses of bacterial 16S rRNA and 16S rRNA gene sequences obtained from gut content microcosms supplemented with ribose. Phylotypes were based on a 97% sequence similarity cutoff. Samples of the three replicates of the 16S rRNA gene control treatment at 0 h and 30 h, 16S rRNA control treatment at 0 h, and all 16S rRNA treatments at 30 h were analyzed separately. Samples of the three replicates were pooled for each of the other treatments at 0 h or 30 h. Abbreviations: 0h and 30h indicate the time of sampling in hours; DNA, 16S rRNA genes; RNA, 16S rRNA; C, unsupplemented control; R, ribose. Identification numbers (e.g., C1) indicate the respective replicates.

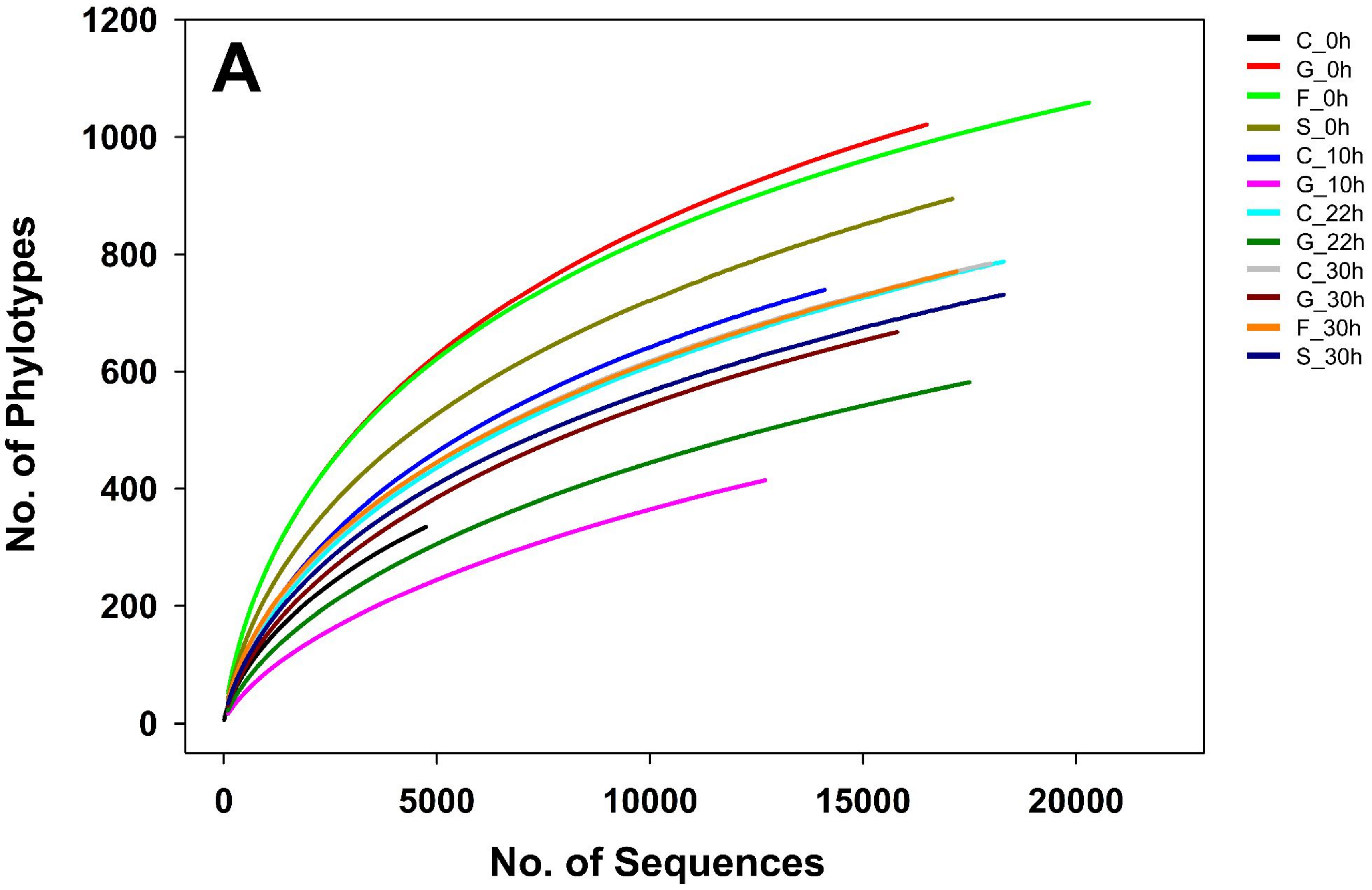
A
1200
1000
800
600
400
200
0
No. of Phylotypes
0
5000
10000
15000
20000
No. of Sequences
C_0h
G_0h
F_0h
S_0h
C_10h
G_10h
C_22h
G_22h
C_30h
G_30h
F_30h
S_30h

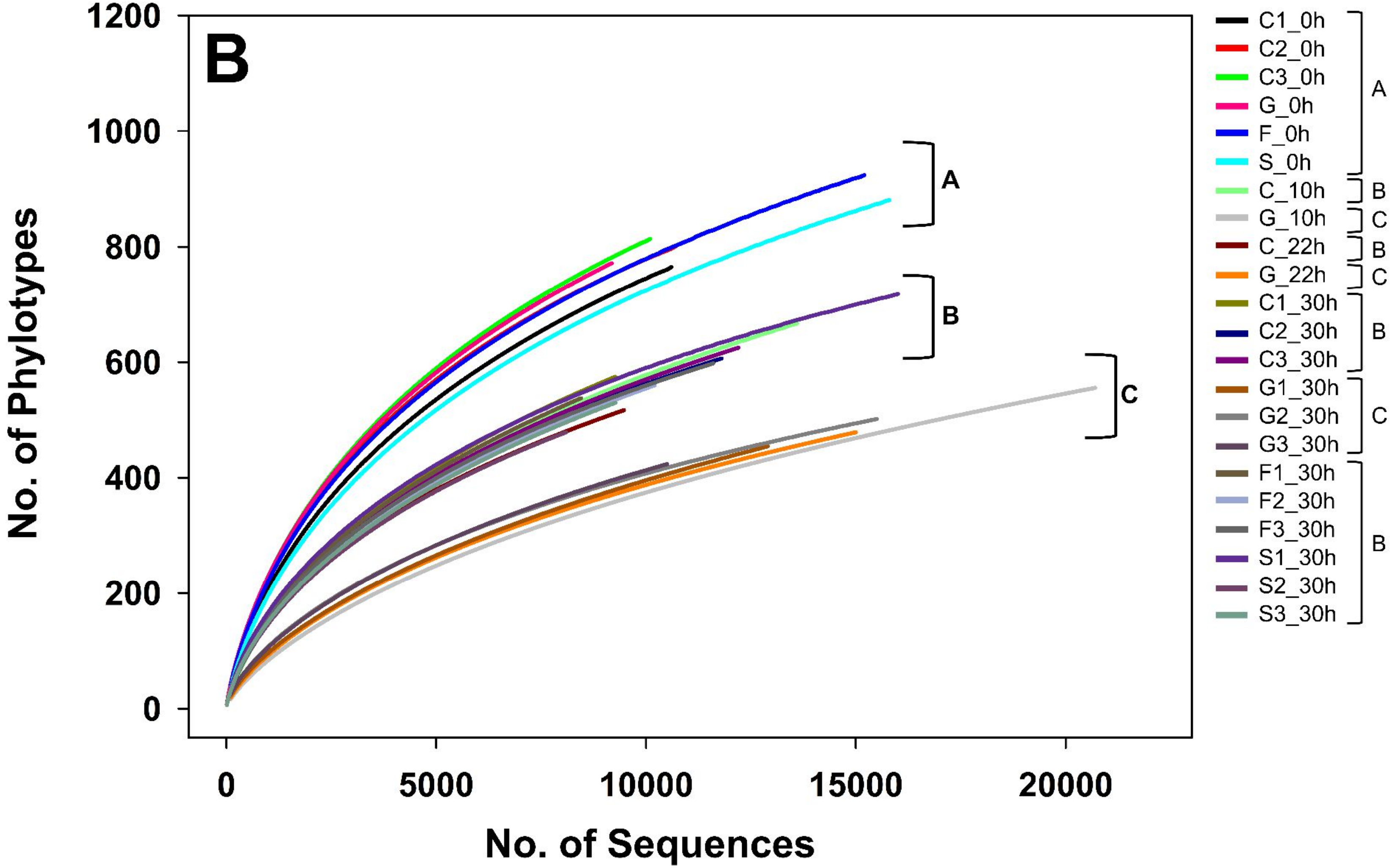

Fig. S6. Rarefaction analyses of bacterial 16S rRNA genes (A) and 16S rRNA (B) sequences obtained from content microcosms supplemented with succinate, formate, and glucose. Phylotypes were based on a 97% sequence similarity cutoff. Samples of the three replicates of the 16S rRNA control treatment at 0 h, and all 16S rRNA treatments at 30 h were analyzed separately. Samples of the three replicates were pooled for each of the other treatments at 0 h, 10 h, 22h, or 30 h. Abbreviations: 0h, 10h, 22h, and 30h indicate the time of sampling in hours; C, unsupplemented control; S, succinate; F, formate; G, glucose. Identification numbers (e.g., C1) indicate the respective replicates.

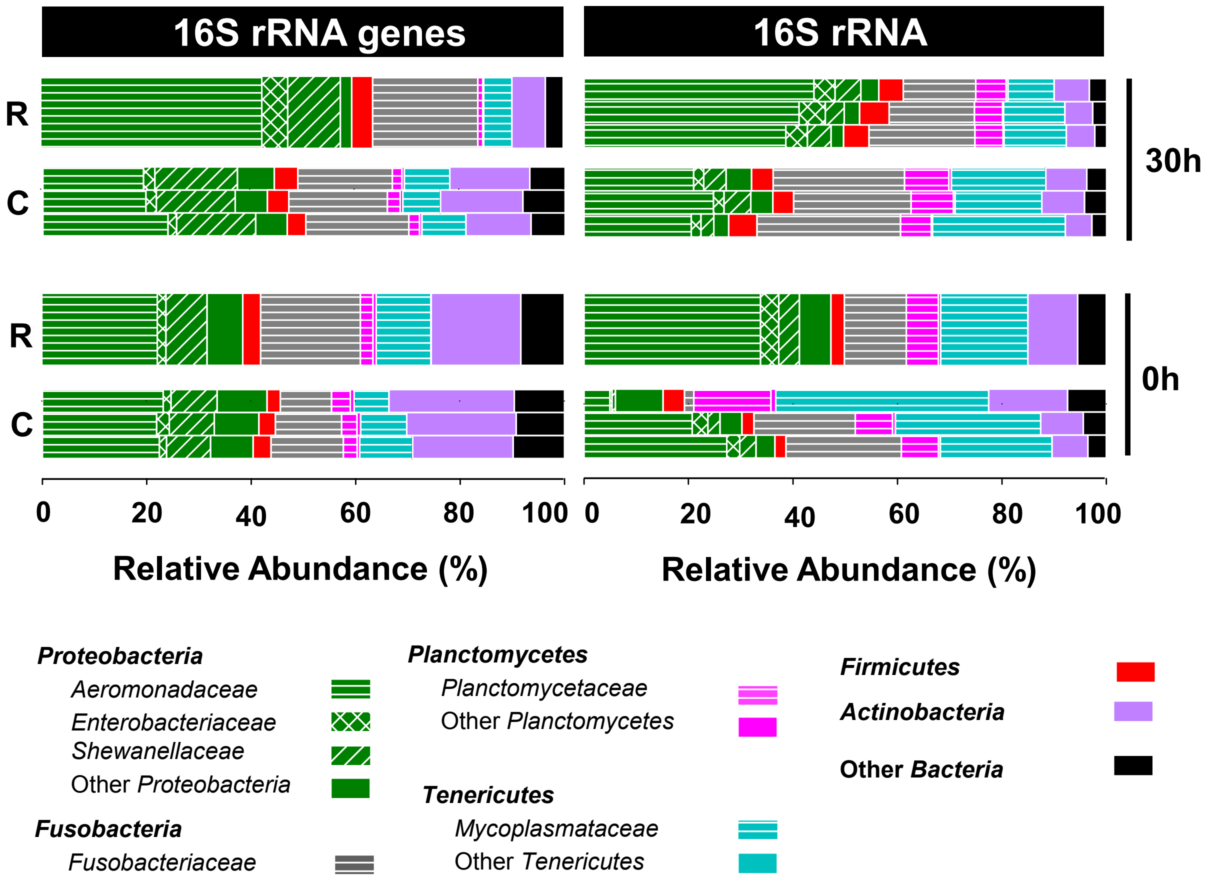

Fig. S7. 16S rRNA and 16S rRNA gene analyses of control and ribose treatments. The most abundant families (i.e., families with ≥ 4% relative abundance in at least one sampling period) are displayed in the color of the respective phylum. Process data are shown Fig. 4A and Table S6, and information on all detected taxa is provided in Table S12. Abbreviations: C, unsupplemented control; R, ribose. Single bars indicate that 16S rRNA or 16S rRNA gene samples of the three replicates were pooled for the sequence analysis and grouped bars indicate that the sequence analysis was performed individually for the three replicates.

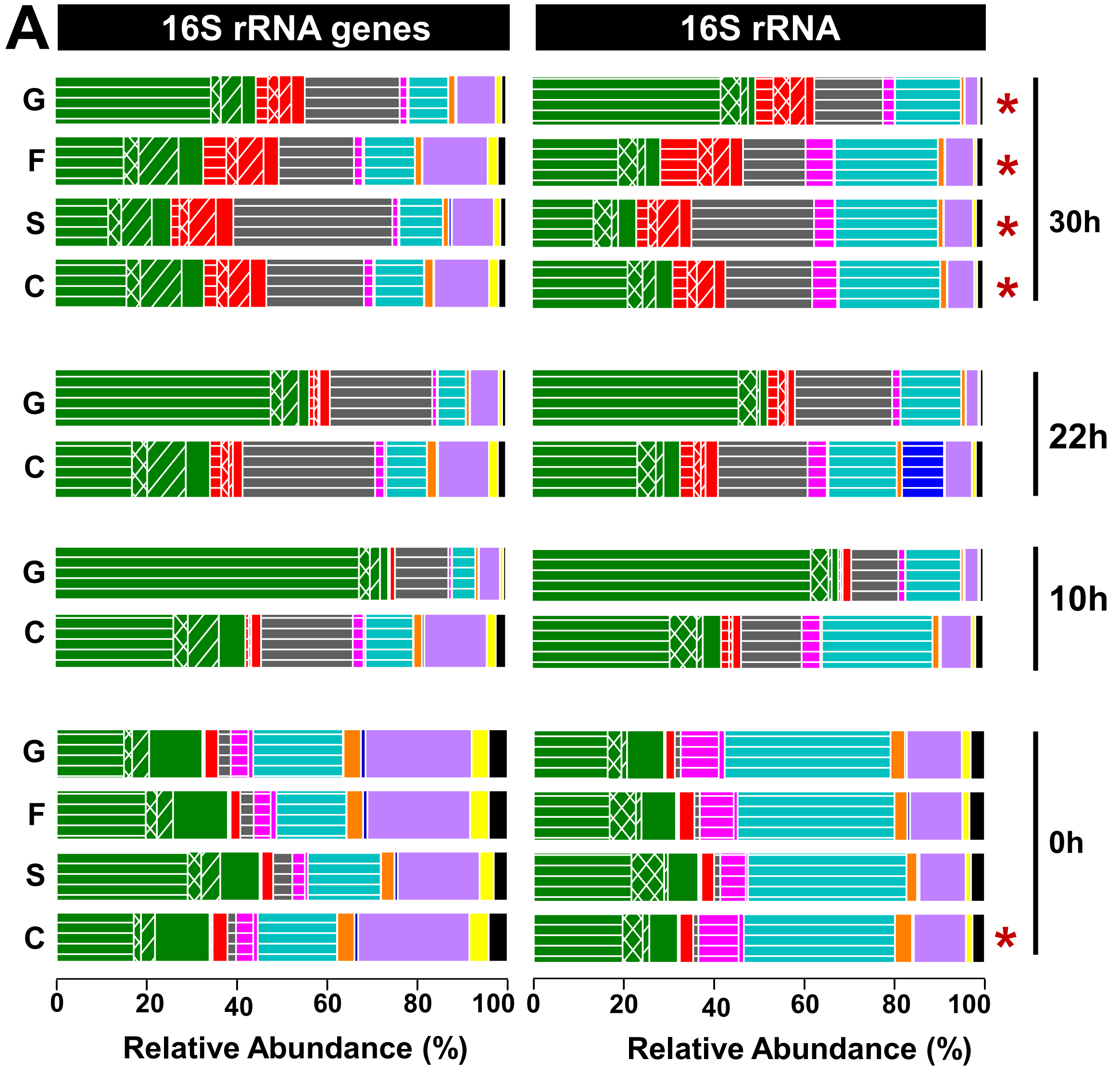
A
16S rRNA genes
16S rRNA
G
F
S
C
30h
22h
10h
0h
*
0
20
40
60
80
100
Relative Abundance (%)
Relative Abundance (%)

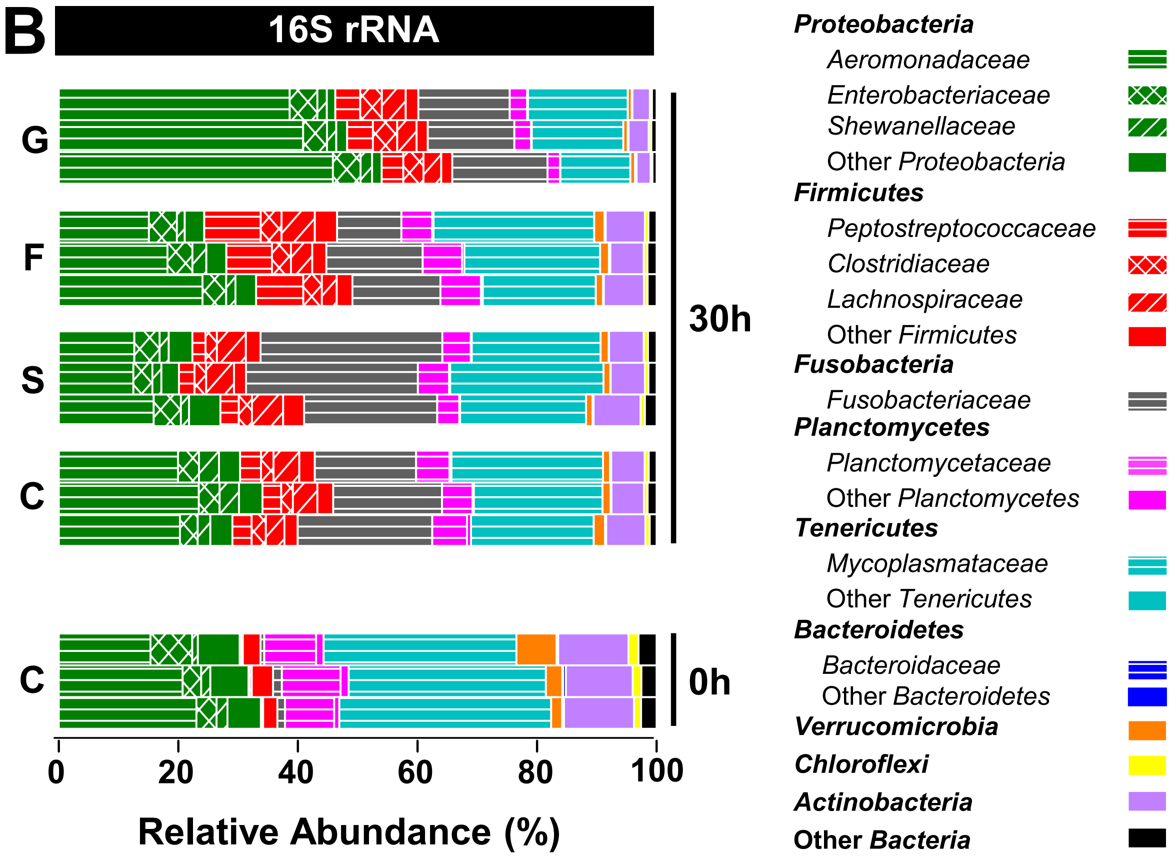

Fig. S8. 16S rRNA gene and 16S rRNA analyses of control, succinate, formate, and glucose treatments. The most abundant families (i.e., families with ≥ 4% relative abundance in at least one sampling period) are displayed in the color of the respective phylum. Process data are shown in Fig. 5, and information on all detected taxa is provided in Table S13. Abbreviations: C, unsupplemented control; S, succinate; F, formate; G, glucose. Panel A: Single bars without asterisk indicate that 16S rRNA gene or 16S rRNA samples of the three replicates were pooled for the sequence analysis. Asterisk indicates analysis was performed individually for the three replicates (see grouped bars Panel B).

Table S1. Effect of amino acids on the fermentation product profiles of anoxic microcosms of *L. terrestris* gut contents.

Treatment	Time (h)	Products (µmol/g_{FW})											pH
		CO_2	H_2	Acetate	Succinate	Prop-ionate	Formate	Lactate	Ethanol	Butyrate	Iso-butyrate	Methyl-butyrate	
**Control**	0	0.0 / 0.0	0.0 / 0.0	6.1 / 6.6	1.4 / 1.5	0.5 / 0.6	2.4 / 2.4	0.8 / 0.8	1.2 / 1.1	0.7 / 0.6	0.0 / 0.1	0.0 / 0.0	7.0 / 7.0
	30	13 / 9.9	0.0 / 0.0	17 / 17	0.0 / 0.0	3.5 / 3.4	0.6 / 0.6	0.5 / 0.5	1.5 / 1.5	1.4 / 1.3	0.1 / 0.1	1.4 / 1.7	7.1 / 7.1
**Casamino Acids**	0	0.0 / 0.0	0.0 / 0.0	9.3 / 9.3	2.0 / 1.9	0.0 / 0.0	2.9 / 3.1	1.2 / 1.2	0.7 / 0.7	0.6 / 0.6	0.1 / 0.1	0.0 / 0.0	7.0 / 7.0
	30	28 / 30	0.5 / 0.6	52 / 52	1.3 / 1.1	11 / 11	0.4 / 0.3	1.3 / 1.4	1.5 / 1.5	3.8 / 3.9	0.3 / 0.3	2.6 / 2.5	7.0 / 7.0
**Alanine**	0	0.0 / 0.0	0.0 / 0.0	5.7 / 6.0	1.3 / 1.3	0.6 / 0.6	2.5 / 2.7	1.0 / 0.8	1.0 / 1.1	0.7 / 0.7	0.0 / 0.1	0.0 / 0.0	7.1 / 7.1
	30	5.4 / 5.4	0.0 / 0.0	18 / 17	0.4 / 0.5	3.0 / 2.6	1.1 / 0.5	0.6 / 0.5	1.0 / 1.0	1.3 / 1.3	0.1 / 0.1	0.7 / 0.8	7.1 / 7.1
**Aspartate**	0	0.0 / 0.0	0.0 / 0.0	5.4 / 5.7	1.3 / 1.4	0.5 / 0.5	2.5 / 4.6	0.9 / 0.9	1.1 / 1.0	0.6 / 0.6	0.1 / 0.0	0.0 / 0.0	6.8 / 6.8
	30	25 / 28	0.0 / 0.0	22 / 24	1.5 / 1.4	28 / 28	0.2 / 0.2	0.9 / 0.9	1.7 / 1.6	1.2 / 1.3	0.3 / 0.3	1.7 / 1.7	6.9 / 6.9
**Glutamate**	0	0.0 / 0.0	0.0 / 0.0	5.5 / 5.3	1.2 / 1.1	0.5 / 0.5	2.6 / 2.5	0.9 / 0.9	0.8 / 0.8	0.6 / 0.7	0.1 / 0.1	0.0 / 0.0	6.7 / 6.7
	30	25 / 38	5.0 / 11	130 / 140	1.9 / 2.0	1.6 / 1.7	11 / 11	0.7 / 0.5	1.8 / 1.5	9.0 / 9.6	0.0 / 0.0	0.3 / 0.4	6.6 / 6.6
**Glycine**	0	0.0 / 0.0	0.0 / 0.0	5.7 / 5.3	1.2 / 1.2	0.5 / 0.5	2.3 / 2.3	0.9 / 0.9	0.9 / 0.9	0.6 / 0.6	0.0 / 0.1	0.0 / 0.0	6.7 / 6.7
	30	7.9 / 11	0.0 / 0.0	21 / 20	0.2 / 0.2	3.4 / 3.4	0.8 / 0.2	0.5 / 0.5	0.9 / 1.1	1.1 / 1.0	0.1 / 0.1	1.6 / 1.6	6.7 / 6.7
**Leucine**	0	0.0 / 0.0	0.0 / 0.0	5.8 / 8.1	1.3 / 1.9	0.5 / 0.6	1.7 / 2.4	0.8 / 1.1	0.9 / 1.3	0.6 / 0.9	0.1 / 0.1	0.0 / 0.0	7.1 / 7.0
	30	7.6 / 8.6	0.0 / 0.0	15 / 15	0.2 / 0.2	2.8 / 2.8	0.8 / 0.9	0.5 / 0.5	1.3 / 1.2	1.0 / 1.0	0.1 / 0.1	2.6 / 2.6	7.1 / 7.1
**Threonine**	0	0.0 / 0.0	0.0 / 0.0	8.0 / 9.0	1.8 / 2.0	0.8 / 1.1	3.0 / 3.0	1.0 / 1.0	0.9 / 0.9	0.9 / 1.0	0.1 / 0.0	0.0 / 0.0	7.0 / 7.1
	30	26 / 17	1.2 / 1.5	18 / 18	1.1 / 1.3	32 / 29	4.1 / 1.1	1.0 / 1.0	1.5 / 1.5	1.9 / 1.9	0.3 / 0.2	0.9 / 0.8	7.0 / 7.0
**Tyrosine**	0	0.0 / 0.0	0.0 / 0.0	6.5 / 6.3	1.6 / 1.5	0.6 / 0.5	2.6 / 2.7	1.0 / 0.9	1.0 / 1.0	0.9 / 0.9	0.1 / 0.1	0.0 / 0.0	7.1 / 7.1
	30	9.0 / 8.8	0.2 / 0.2	19 / 19	0.0 / 0.0	3.0 / 3.1	0.3 / 0.3	0.5 / 0.5	2.3 / 2.6	0.9 / 1.0	0.1 / 0.0	1.0 / 1.0	7.1 / 7.1
**Valine**	0	0.0 / 0.0	0.0 / 0.0	7.9 / 7.6	1.8 / 1.6	0.6 / 0.6	2.5 / 2.5	0.8 / 0.8	1.3 / 1.0	0.9 / 0.8	0.0 / 0.1	0.0 / 0.0	7.1 / 7.1
	30	7.1 / 3.5	0.0 / 0.0	16 / 15	0.0 / 0.0	2.9 / 2.9	0.3 / 0.3	0.5 / 0.5	0.8 / 0.9	0.9 / 0.8	1.9 / 1.8	0.0 / 0.1	7.1 / 7.1

[a]The amount of amino acids per microcosm approximated 10 mM; control lacked supplement. Amounts of products formed in the duplicates are separated by a slash. FW, fresh weight.

Table S2. *P* values of fermentation products in amino acid treatments.[a]

**Products**				**CO_2**								**H_2**			
**Treatment**	C	CAA	Glu	Asp	Thr	Ala/Gly	Val/Gly		C	CAA	Glu	Asp	Thr	Ala/Gly	Val/Gly
**Mean value[b]**	14	35	63	66	32	17	20		0.4	1.1	26	0.0	0.0	-1.1	-1.0
**Variance**	4.2	14	142	70	6.6	9.0	5.5		0.0	0.0	9.3	0.0	0.2	0.0	0.0
P value		0.003	0.020	0.009	0.001	0.168	0.034			0.023	0.005	0.000	0.264	0.000	0.002
**Products**				**Acetate**								**Succinate**			
**Treatment**	C	CAA	Glu	Asp	Thr	Ala/Gly	Val/Gly		C	CAA	Glu	Asp	Thr	Ala/Gly	Val/Gly
**Mean valueb**	11	48	183	32	18	18	14		-0.9	-0.9	0.5	34	0.0	-1.1	-1.0
**Variance**	0.0	2.4	2.5	1.7	27	1.3	0.4		0.0	0.0	0.0	2.1	0.2	0.0	0.0
P value		0.001	0.000	0.001	0.127	0.009	0.010			0.650	0.004	0.001	0.095	0.002	0.130
**Products**				**Formate**								**Propionate**			
**Treatment**	C	CAA	Glu	Asp	Thr	Ala/Gly	Val/Gly		C	CAA	Glu	Asp	Thr	Ala/Gly	Val/Gly
**Mean value[b]**	1.1	1.1	17	0.7	1.8	0.4	0.7		2.3	9.6	0.6	20	37	2.3	2.4
**Variance**	1.0	0.2	0.3	0.8	0.8	0.2	0.1		0.0	0.1	0.0	1.7	78	0.1	0.0
P value		0.999	0.000	0.640	0.397	0.388	0.596			0.000	0.000	0.002	0.021	0.760	0.186
**Products**				**Butyrate**								**Methylbutyrate**			
**Treatment**	C	CAA	Glu	Asp	Thr	Ala/Gly	Val/Gly		C	CAA	Glu	Asp	Thr	Ala/Gly	Val/Gly
**Mean value[b]**	0.1	3.7	8.2	0.3	3.0	0.3	0.2		1.8	4.2	0.8	1.9	1.7	1.7	0.3
**Variance**	0.0	0.1	0.0	0.0	0.0	0.0	0.0		0.0	0.0	0.0	0.1	0.0	0.0	0.0
P value		0.001	0.000	0.237	0.000	0.234	0.902			0.000	0.000	0.493	0.385	0.165	0.000
**Products**				**Isobutyrate**								**Lactate**			
**Treatment**	C	CAA	Glu	Asp	Thr	Ala/Gly	Val/Gly		C	CAA	Glu	Asp	Thr	Ala/Gly	Val/Gly
**Mean value[b]**	0.1	0.2	0.0	0.3	0.3	0.1	3.2		-0.1	0.0	0.5	0.2	0.1	0.0	0.0
**Variance**	0.0	0.0	0.0	0.0	0.0	0.0	0.0		0.0	0.0	0.0	0.0	0.0	0.0	0.0
P value		0.067	0.057	0.078	0.036	0.549	0.000			0.040	0.000	0.025	0.133	0.052	0.023
**Products**				**Ethanol**								**Total**			
**Treatment**	C	CAA	Glu	Asp	Thr	Ala/Gly	Val/Gly		C	CAA	Glu	Asp	Thr	Ala/Gly	Val/Gly
**Mean value[b]**	1.8	1.3	2.3	2.2	1.8	1.6	2.0		31	103	301	158	101	41	42
**Variance**	0.3	0.0	0.0	0.1	0.0	0.0	0.0		2.9	9.6	219	55	146	13	3.4
P value		0.317	0.243	0.403	0.774	0.627	0.563			0.000	0.001	0.001	0.010	0.027	0.002

[a]*P* values (significant at $P \leq 0.05$) were calculated by *t*-test with unequal variances and are based on the difference between the net amount of products in control (C) and casamino acid (CAA), glutamate (Glu), aspartate (Asp), threonine (Thr), alanine and glycine (Ala/Gly), and valine and glycine (Val/Gly) treatments. To calculate net amounts, amounts of products at the beginning of incubation were subtracted from those at the end of incubation. See Fig. 1 for product profile.

[b]Mean values (n = 3) are in μmol/g_{FW} (FW, fresh weight).

Table S3. Production of ammonium in amino acid treatments.[a]

Treatment	Time (h)	NH_4^+ (mM)
**Control**	0	0.6 ± 0.5
	30	0.0 ± 0.0
**Casamino Acids**	0	0.8 ± 0.3
	30	4.7 ± 0.6
**Glutamate**	0	0.9 ± 0.1
	30	9.5 ± 1.0
**Aspartate**	0	1.0 ± 0.0
	30	7.8 ± 0.7
**Threonine**	0	0.6 ± 0.2
	30	2.7 ± 0.2
**Alanine/Glycine**	0	0.6 ± 0.0
	30	1.3 ± 0.3
**Valine/Glycine**	0	0.4 ± 0.1
	30	1.2 ± 0.3

[a]The amount of amino acids per microcosm approximated 10 mM; control lacked supplement. Values are the arithmetic average of three replicate analyses (± standard deviation). See Fig. 1 for product profile.

Table S4. Statistical analyses of stimulated families in amino acid treatments.[a]

Family	Treatment	Mean	Standard Deviation	Median	LDA Score (log10)[b]
Clostridiaceae	Control	2.9	0.3	2.9	
	Glutamate	7.7	1.0	7.8	4.9(3)
	Threonine	3.6	0.2	3.6	4.5(3)
Enterobacteriaceae	Control	3.0	0.2	3.1	
	Casamino Acids	5.4	0.6	5.4	4.7(2)
	Glutamate	10	1.3	10	5.0(2)
	Aspartate	8.1	0.4	8.1	4.9(2)
	Threonine	4.3	0.2	4.3	4.6(4)
	Alanine/Glycine	4.2	0.4	4.3	4.6(2)
	Valine/Glycine	4.9	0.3	4.9	4.7(2)
Fusobacteriaceae	Control	16	0.5	16	
	Casamino Acids	25	0.3	25	5.4(1)
	Glutamate	29	2.3	29	5.5(1)
	Aspartate	22	0.7	22	5.3(1)
	Threonine	18	1.2	17	5.3(1)
Lachnospiraceae	Control	1.9	0.3	2.0	
	Alanine/Glycine	3.7	0.6	3.8	4.6(3)
	Valine/Glycine	3.9	0.6	3.9	4.6(3)
Peptostreptococcaceae	Control	1.1	0.1	1.1	
	Casamino Acids	3.9	0.3	3.8	4.6(3)
	Threonine	6.3	0.6	6.0	4.8(2)
	Alanine/Glycine	12	0.1	12	5.1(1)
	Valine/Glycine	4.9	1.0	4.7	4.7(1)
Shewanellaceae	Control	3.3	0.3	3.4	
	Threonine	4.4	0.1	4.3	4.6(3)

[a]Families reaching a LDA score ≥ 4.0 were considered. LEfSe analysis, mean value, standard deviation, and median are based on the relative abundance of 16S rRNA sequences of the three replicates per treatment at the end of the incubation.

[b]LDA scores were calculated using LEfSe. Numbers in parentheses display the rank in the LDA analysis (i.e., higher ranking families exhibited a stronger response to supplement compared to lower ranking ones).

Table S5. Alpha diversity of the microbial community in control and amino acid treatments.[a]

(A) 16S rRNA

Sampling Time	0h									10h					
Treatment	C1	C2	C3	CAA	Glu	Asp	Thr	Ala/Gly	Val/Gly	C	CAA	Glu	Asp	Thr	Ala/Gly
No of Sequences	211207	124938	176174	117131	166463	131510	174976	127750	159830	122862	168746	126412	160540	113043	97295
Observed PTs[b] (normalized)[c]	2803 (477)	2345 (476)	2561 (477)	2362 (476)	2540 (477)	2454 (477)	2503 (476)	2356 (478)	2544 (478)	2318 (482)	2271 (480)	2260 (480)	2435 (481)	2260 (481)	2321 (481)
Chao1 (normalized)[c]	3406 (482)	2969 (478)	3309 (480)	3143 (478)	3164 (479)	3232 (479)	3180 (479)	3013 (484)	3302 (480)	2958 (486)	2887 (482)	3121 (482)	3019 (483)	3026 (484)	3012 (482)
Shannon (normalized)[c]	4.6 (4.0)	4.4 (3.9)	4.3 (3.8)	4.4 (3.9)	4.4 (3.9)	4.5 (4.0)	4.1 (3.7)	4.4 (3.9)	4.5 (4.0)	4.3 (3.8)	3.6 (3.2)	3.9 (3.5)	4.2 (3.7)	4.3 (3.8)	4.6 (4.1)

Sampling Time	10h	22h							30h						
Treatment	Val/Gly	C	CAA	Glu	Asp	Thr	Ala/Gly	Val/Gly	C1	C2	C3	CAA1	CAA2	CAA3	Glu1
No of Sequences	120964	135915	94755	115249	120291	140737	136979	139028	121953	145730	135750	154449	57895	109922	127517
Observed PTs[b] (normalized)[c]	2414 (482)	2490 (486)	1862 (483)	1953 (481)	2053 (483)	2333 (484)	2514 (485)	2459 (485)	2345 (485)	2359 (485)	2316 (486)	2241 (484)	1700 (482)	2041 (482)	2084 (484)
Chao1 (normalized)[c]	3167 (486)	3131 (486)	2533 (485)	2632 (485)	2723 (485)	2963 (485)	3179 (486)	3186 (485)	3160 (486)	3011 (486)	2954 (486)	2914 (485)	2362 (484)	2660 (485)	2784 (486)
Shannon (normalized)[c]	4.6 (4.1)	4.4 (3.9)	3.6 (3.2)	3.6 (3.2)	3.8 (3.5)	4.2 (3.8)	4.8 (4.3)	4.7 (4.2)	4.6 (4.1)	4.4 (3.9)	4.4 (4.0)	4.0 (3.7)	4.0 (3.6)	4.0 (3.6)	3.8 (3.4)

Sampling Time	30h													
Treatment	Glu2	Glu3	Asp1	Asp2	Asp3	Thr1	Thr2	Thr3	Ala/Gly1	Ala/Gly2	Ala/Gly3	Val/Gly1	Val/Gly2	Val/Gly2
No of Sequences	102218	98379	132724	98432	108184	103154	158797	138039	130264	161641	145579	126821	128701	117114
Observed PTs[b] (normalized)[c]	1985 (482)	1925 (482)	2280 (483)	1980 (484)	2039 (484)	2048 (484)	2348 (485)	2202 (486)	2312 (486)	2437 (484)	2341 (484)	2248 (484)	2192 (484)	2228 (483)
Chao1 (normalized)[c]	2647 (485)	2524 (485)	2914 (485)	2806 (485)	2690 (485)	2595 (487)	3010 (486)	2808 (487)	2997 (486)	3129 (486)	3080 (486)	2895 (486)	2898 (487)	2948 (484)
Shannon (normalized)[c]	3.9 (3.5)	3.8 (3.4)	4.2 (3.7)	4.0 (3.6)	4.0 (3.6)	4.3 (3.9)	4.4 (3.9)	4.4 (4.0)	4.6 (4.1)	4.6 (4.1)	4.5 (4.1)	4.5 (4.1)	4.3 (3.9)	4.5 (4.0)

(B) 16S rRNA genes

Sampling Time	0h							10h						
Treatment	C	CAA	Glu	Asp	Thr	Ala/Gly	Val/Gly	C	CAA	Glu	Asp	Thr	Ala/Gly	Val/Gly
No of Sequences	128322	108230	116420	136612	118672	109665	169345	6431	91618	125262	93662	148875	93869	111828
Observed PTs[b] (normalized)[c]	2594 (477)	2431 (476)	2485 (476)	2594 (476)	2491 (475)	2422 (475)	2741 (475)	646 -[d]	1887 (465)	2232 (471)	2165 (475)	2531 (476)	2328 (478)	2378 (477)
Chao1 (normalized)[c]	3418 (480)	3114 (479)	3342 (481)	3445 (484)	3290 (477)	3202 (479)	3534 (479)	1084 -[d]	2652 (473)	3011 (476)	3036 (480)	3274 (479)	3156 (484)	3185 (480)
Shannon (normalized)[c]	5.0 (4.5)	4.9 (4.4)	4.9 (4.4)	4.8 (4.3)	4.8 (4.3)	4.8 (4.3)	4.8 (4.3)	4.1 -[d]	3.5 (3.2)	3.8 (3.4)	4.3 (3.9)	4.5 (4.0)	4.8 (4.3)	4.7 (4.2)

Sampling Time	22h							30h						
Treatment	C	CAA	Glu	Asp	Thr	Ala/Gly	Val/Gly	C	CAA	Glu	Asp	Thr	Ala/Gly	Val/Gly
No of Sequences	96516	147534	153193	120118	120781	113657	128504	119569	144816	102791	143408	146505	142761	144847
Observed PTs[b] (normalized)[c]	2167 (480)	2101 (472)	2076 (474)	2000 (475)	2137 (479)	2233 (478)	2330 (480)	2294 (480)	2123 (473)	1874 (470)	2130 (473)	2278 (480)	2417 (480)	2265 (479)
Chao1 (normalized)[c]	2940 (484)	3155 (482)	2897 (483)	2723 (482)	2855 (483)	2949 (481)	3050 (484)	3027 (483)	2870 (481)	2484 (477)	2894 (483)	2916 (483)	3218 (483)	3006 (484)
Shannon (normalized)[c]	4.5 (4.0)	3.3 (3.0)	3.3 (3.0)	3.6 (3.3)	4.1 (3.7)	4.7 (4.2)	4.5 (4.0)	4.5 (4.1)	3.7 (3.4)	3.5 (3.2)	3.9 (3.5)	4.3 (3.9)	4.7 (4.3)	4.3 (3.9)

[a]Samples of the three replicates of the 16S rRNA control treatment at 0 h, and all 16S rRNA treatments at 30 h were analyzed separately. Samples of the three replicates were pooled for each of the other treatments at 0 h, 10 h, 22 h, or 30 h. Abbreviations: C, unsupplemented control; CAA, casamino acids; Glu, glutamate; Asp, aspartate; Thr, threonine; Ala, alanine; Gly, glycine; Val, valine. Identification numbers (e.g., C1) indicate the respective replicates.

[b]Phylotypes were clustered based on a sequence similarity cut-off of 97%.

[c]For comparison of amplicon libraries of different sizes, the data sets were normalized to 50,000 sequences.

[d]-, normalization was not possible because of the low number of sequences in this sample.

Table S6. Effect of ribose on the fermentation product profiles of anoxic microcosms of *L. terrestris* gut contents.[a]

Treatment	Time (h)	Products (µmol/g_{FW})										Ribose	pH
		CO_2	H_2	Acetate	Succinate	Prop-ionate	Methyl-butyrate	Formate	Butyrate	Ethanol	Lactate		
Control	0	0.0 ± 0.0	0.0 ± 0.0	6.4 ± 0.1	1.1 ± 0.0	0.7 ± 0.0	0.0 ± 0.0	2.1 ± 0.1	0.5 ± 0.0	0.0 ± 0.0	0.9 ± 0.0	0.0 ± 0.0	7.0 ± 0.0
	30	11 ± 2.7	0.1 ± 0.3	14 ± 0.5	0.4 ± 0.1	2.9 ± 0.1	0.9 ± 0.1	3.6 ± 0.4	1.1 ± 0.2	3.0 ± 0.8	1.0 ± 0.0	0.0 ± 0.0	7.0 ± 0.0
Ribose	0	0.0 ± 0.0	0.0 ± 0.0	6.4 ± 2.0	1.1 ± 0.3	0.7 ± 0.1	0.0 ± 0.0	2.7 ± 0.7	0.5 ± 0.0	0.9 ± 0.1	0.6 ± 0.1	56 ± 23	7.0 ± 0.0
	30	21 ± 2.5	4.1 ± 0.8	26 ± 1.7	1.0 ± 0.3	6.6 ± 0.4	0.2 ± 0.1	7.5 ± 0.6	1.3 ± 0.1	17 ± 0.8	1.0 ± 0.0	36 ± 2.7	6.9 ± 0.0

[a]The amount of ribose per microcosm approximated 5 mM; controls lacked supplement. FW, fresh weight.

Table S7. *P* values of fermentation products in ribose, succinate, formate, and glucose treatments.[a]

**Products**			**CO_2**							**H_2**		
**Treatment**	C_A	R	C_B	S	F	G	C_A	R	C_B	S	F	G
**Mean value[b]**	11	21	25	48	51	94	0.1	4.1	2.3	1.7	31	51
**Variance**	7.5	6.3	16	29	4.6	1.2	0.1	0.7	0.0	0.0	5.3	7.9
***P* value[c]**		0.010		0.004	0.002	0.001		0.004		0.030	0.002	0.001
**Products**			**Acetate**							**Succinate**		
**Treatment**	C_A	R	C_B	S	F	G	C_A	R	C_B	S	F	G
**Mean value[b]**	7.1	19	24	18	26	61	-0.7	0.1	-0.9	-	-0.9	-1.1
**Variance**	0.3	2.4	4.8	2.1	7.8	2.2	0.0	0.1	0.0	-	0.0	0.0
***P* value[c]**		0.006		0.031	0.328	0.000		0.105		-	0.821	0.075
**Products**			**Formate**							**Propionate**		
**Treatment**	C_A	R	C_B	S	F	G	C_A	R	C_B	S	F	G
**Mean value[b]**	1.5	4.8	0.0	-1.4	-	4.2	2.3	5.9	4.9	47.4	5.0	11
**Variance**	0.3	0.3	0.1	0.3	-	0.1	0.0	0.2	0.2	161	0.2	0.0
***P* value[c]**		0.002		0.040	-	0.000		0.005		0.028	0.727	0.002
**Products**			**Butyrate**							**Methylbutyrate**		
**Treatment**	C_A	R	C_B	S	F	G	C_A	R	C_B	S	F	G
**Mean value[b]**	0.0	0.0	1.6	1.2	1.4	1.1	0.0	0.0	2.9	2.4	3.0	2.5
**Variance**	0.0	0.0	0.0	0.0	0.0	0.1	0.0	0.0	0.2	0.1	0.1	0.0
***P* value[c]**		-		0.013	0.185	0.082		-		0.178	0.822	0.282
**Products**			**Isobutyrate**							**Lactate**		
**Treatment**	C_A	R	C_B	S	F	G	C_A	R	C_B	S	F	G
**Mean value[b]**	0.0	0.0	0.2	0.2	0.3	0.0	0.1	0.4	-0.4	-0.5	0.4	12.1
**Variance**	0.0	0.0	0.0	0.0	0.0	0.0	0.0	0.0	0.0	0.0	0.1	0.4
***P* value[c]**		-		0.525	0.281	0.006		0.004		0.204	0.029	0.001
**Products**			**Ethanol**							**Total**		
**Treatment**	C_A	R	C_B	S	F	G	C_A	R	C_B	S	F	G
**Mean value[b]**	3.0	15	2.6	2.9	3.2	43	25	66	77	231	172	299
**Variance**	0.6	0.7	0.3	0.8	0.5	2.1	8.5	18	103	460	431	197
***P* value[c]**		0.003		0.666	0.332	0.000		0.000		0.002	0.006	0.000

[a]*P* values (significant at $P \leq 0.05$) were calculated by *t*-test with unequal variances and are based on the difference between the net amount of products in control (C_A and C_B), ribose (R), succinate (S), formate (F), or glucose (G) treatments. To calculate net amounts, amounts of products at the beginning of incubation were subtracted from those at the end of incubation. See Fig. 4 for product profiles.

[b]Mean values (n = 3) are in $\mu mol/g_{FW}$ (FW, fresh weight).

Table S8. Statistical analyses of stimulated families in ribose, glucose and transient intermediate treatments.[a]

Family	Treatment	Mean	Standard Deviation	Median	LDA Score (log10)[b]
Aeromonadaceae	$Control_A$	22	2.3	21	
	Ribose	41	2.7	41	5.6[(1)]
	$Control_B$	21	1.9	20	
	Glucose	42	3.7	41	5.6[(1)]
Clostridiaceae	$Control_B$	2.1	0.2	2.1	
	Formate	3.3	0.2	3.2	4.5[(3)]
	Glucose	3.7	0.4	3.6	4.6[(4)]
Enterobacteriaceae	$Control_A$	2.0	0.1	1.9	
	Ribose	4.4	0.5	4.1	4.6[(2)]
	$Control_B$	3.3	3.0	3.5	
	Formate	4.3	0.4	4.2	4.6[(2)]
	Glucose	4.4	0.4	4.6	4.6[(2)]
Lachnospicraeae	$Control_B$	3.8	0.6	4.1	
	Succinate	4.9	0.3	4.8	4.7[(1)]
Peptostreptococcaceae	$Control_B$	3.3	0.2	3.2	
	Formate	8.3	1.0	7.9	5.0[(1)]
	Glucose	4.0	0.4	4.2	4.6[(3)]

[a]Families reaching a LDA score ≥ 4.0 were considered. LEfSe analysis, mean value, standard deviation, and median are based on the relative abundance of 16S rRNA sequences of the three replicates per treatment at 30 h of incubation.

[b]LDA scores were calculated using LEfSe. Numbers in parentheses display the rank in the LDA analysis (i.e., higher ranking families exhibited a stronger response to supplement compared to lower ranking ones).

Table S9. Alpha diversity of the microbial community in control, ribose, succinate, formate and glucose treatments.[a]

(A) 16S rRNA genes

Sampling Time	0h								10h	
Treatment[a]	C_A1	C_A2	C_A3	R	C_B	S	F	G	C	G
No of Sequences	15345	10377	11331	10234	15359	17131	20373	16530	14156	12798
Observed PTs[b] (normalized)[c]	776 (198	681 (199)	734 (203)	650 (188)	965 (235)	896 (221)	1060 (232)	1022 (232)	742 (216)	416 (139)
Chao1 (normalized)[c]	1046 (241	984 (234)	1019 (236)	974 (227)	1315 (253)	1311 (239)	1380 (245)	1337 (247)	1109 (243)	720 (185)
Shannon (normalized)[c]	4.0 (3.5	4.1 (3.6)	4.3 (3.7)	3.8 (3.4)	4.6 (3.9)	3.9 (3.3)	4.5 (3.8)	4.6 (3.8)	3.5 (3.0)	1.7 (1.5)

Sampling Time	22h		30h							
Treatment[a]	C	G	C_A1	C_A2	C_A3	R	C_B	S	F	G
No of Sequences	18327	17591	8937	9669	9364	7845	18096	18342	17229	15892
Observed PTs[b] (normalized)[c]	789 (216)	583 (175)	571 (179)	624 (192)	597 (190)	364 (136)	787 (223)	733 (209)	772 (220)	669 (204)
Chao1 (normalized)[c]	1193 (244)	912 (223)	317 (223)	995 (237)	958 (232)	611 (196)	1105 (255)	1071 (237)	1105 (249)	998 (236)
Shannon (normalized)[c]	3.4 (3.0)	2.3 (2.1)	3.5 (3.1)	3.7 (3.3)	3.7 (3.3)	2.6 (2.4)	3.7 (3.3)	3.3 (2.9)	3.9 (3.3)	3.1 (2.4)

(B) 16S rRNA

Sampling Time	0h										10h		22h		30h		
**Treatment[a]**	**C_A1**	**C_A2**	**C_A3**	**R**	**C_B1**	**C_B2**	**C_B3**	**S**	**F**	**G**	**C**	**G**	**C**	**G**	**C_A1**	**C_A2**	**C_A3**
**No of Sequences**	3684	2958	4255	9677	10695	10734	10158	15874	15238	9185	13621	20734	9473	15076	4433	5019	4226
**Observed PTs[b]**	339	307	556	643	768	802	816	883	925	772	668	556	517	480	333	428	399
**(normalized)[c]**	(144)	(152)	(213)	(188)	(216)	(225)	(227)	(218)	(232)	(224)	(197)	(142)	(202)	(157)	(146)	(176)	(168)
**Chao1**	634	581	866	10110	1178	1185	1242	1325	1332	1163	1074	1019	891	865	634	743	813
**(normalized)[c]**	(175)	(183)	(242)	(234)	(237)	(240)	(246)	(240)	(248)	(240)	(229)	(199)	(228)	(211)	(196)	(217)	(212)
**Shannon**	3.1	3.1	4.0	3.5	3.5	3.8	3.9	3.5	3.8	3.8	3.0	1.9	3.2	2.2	2.9	3.4	3.3
**(normalized)[c]**	(2.7)	(2.9)	(3.4)	(3.1)	(3.0)	(3.2)	(3.3)	(2.9)	(3.2)	(3.2)	(2.7)	(1.7)	(2.9)	(2.0)	(2.6)	(3.0)	(3.0)

Sampling Time	30h														
**Treatment[a]**	**R1**	**R2**	**R3**	**C_B1**	**C_B2**	**C_B3**	**S1**	**S2**	**S3**	**F1**	**F2**	**F3**	**G1**	**G2**	**G3**
**No of Sequences**	6497	6020	7561	9280	11820	12244	16056	8100	9278	8459	10208	11610	12941	15522	10518
**Observed PTs[b]**	382	366	458	575	607	626	719	479	530	538	560	598	455	502	424
**(normalized)[c]**	(149)	(146)	(160)	(202)	(204)	(214)	(220)	(192)	(200)	(208)	(197)	(207)	(151)	(167)	(162)
**Chao1**	675	554	773	986	987	1004	1012	793	913	951	979	897	851	808	744
**(normalized)[c]**	(206)	(198)	(215)	(229)	(242)	(252)	(250)	(224)	(234)	(237)	(223)	(237)	(195)	(210)	(206)
**Shannon**	2.9	3.0	3.1	3.3	3.3	3.3	3.5	3.1	3.2	3.4	3.4	3.5	2.5	2.7	2.7
**(normalized)[c]**	(2.6)	(2.7)	(2.8)	(3.0)	(2.9)	(3.0)	(3.1)	(2.8)	(2.8)	(3.1)	(3.1)	(3.2)	(2.3)	(2.5)	(2.5)

[a]For the ribose experiment: Samples of the three replicates of the 16S rRNA gene control treatment at 0 h and 30 h, 16S rRNA control treatment at 0 h, and all 16S rRNA treatments at 30 h were analyzed separately. Samples of the three replicates were pooled for each of the other treatments at 0 h or 30 h. Abbreviations: C_A, unsupplemented control; R, ribose. For the transient intermediate experiment: Samples of the three replicates of the 16S rRNA control treatment at 0 h, and all 16S rRNA treatments at 30 h were analyzed separately. Samples of the three replicates were pooled for each of the other treatments at 0 h, 10 h, 22h, or 30 h. Abbreviations: C_B, unsupplemented control; S, succinate; F, formate, G, glucose.

[b]Phylotypes were clustered based on a sequence similarity cut-off of 97%.

[c]For comparison of amplicon libraries of different sizes, the transient data sets were normalized to 5,000 and the ribose data set were normalized to 2,500 sequences.

Table S10. Statistical analyses of main stimulated phylotypes displayed in Fig. 6.[a]

Group Phylotype	Phylotype[b]	Treatment	Mean	Standard Deviation	Median	LDA Score (log10)[c]
**GPT-1**	T3	Control	21	1.9	20	
		Glucose	42	3.7	41	$5.8^{(1)}$
	R96	Control	13	0.7	13	
		Ribose	30	1.5	29	$5.5^{(1)}$
**GPT-2**	A6	Control	2.1	0.2	2.2	
		Casamino Acids	3.3	0.3	3.3	$4.5^{(2)}$
		Glutamate	4.3	0.6	4.2	$4.6^{(3)}$
		Aspartate	3.2	0.2	3.1	$4.5^{(2)}$
		Alanine/Glycine	2.9	0.3	3.0	$4.5^{(3)}$
		Valine/Glycine	3.4	0.1	3.4	$4.5^{(2)}$
	T6	Control	2.9	0.2	2.9	
		Glucose	3.7	0.3	3.6	$4.6^{(2)}$
		Formate	3.9	0.4	3.8	$4.6^{(2)}$
	R5	Control	1.6	0.1	1.6	
		Ribose	3.3	0.5	3.1	$4.5^{(2)}$
**GPT-3**	A129	Control	0.4	0.0	0.4	
		Casamino Acids	1.1	0.1	1.0	$4.0^{(5)}$
		Glutamate	3.2	0.3	3.2	$4.5^{(4)}$
		Aspartate	2.4	0.1	2.4	$4.4^{(3)}$
		Threonine	1.0	0.1	1.0	$4.0^{(6)}$
		Alanine/Glycine	0.6	0.1	0.7	$3.8^{(7)}$
		Valine/Glycine	0.7	0.1	0.8	$3.9^{(4)}$
	A1526	Control	0.3	0.0	0.3	
		Glutamate	2.6	0.4	2.6	$4.4^{(5)}$
		Aspartate	2.0	0.1	2.1	$4.3^{(4)}$
		Threonine	0.8	0.0	0.8	$3.9^{(7)}$
		Alanine/Glycine	0.5	0.1	0.5	$3.7^{(10)}$
		Valine/Glycine	0.6	0.1	0.6	$3.8^{(9)}$
**GPT-4**	A25	Control	0.1	0.0	0.2	
		Casamino Acids	1.1	0.3	1.0	$4.0^{(4)}$
		Glutamate				3.8(7)
		Threonine	2.7	0.2	2.6	$4.4^{(3)}$
		Alanine/Glycine	0.6	0.3	0.4	3.8(8)
		Valine/Glycine	0.7	0.2	0.8	3.8(5)
	T7	Control	1.0	0.1	0.9	
		Glucose	2.5	0.5	2.8	$4.4^{(3)}$
		Formate	4.2	0.8	4.2	$4.6^{(1)}$
**GPT-5**	A1	Control	16	0.5	16	
		Casamino Acids	25	0.3	25	$5.4^{(1)}$
		Glutamate	29	1.1	29	$5.5^{(1)}$
		Aspartate	21	0.7	21	$5.3^{(1)}$
		Threonine	18	1.2	17	$5.2^{(1)}$
	A8	Control	0.6	0.0	0.6	
		Casamino Acids	1.6	0.1	1.6	$4.2^{(3)}$
		Threonine	1.6	0.3	1.4	$4.2^{(4)}$
		Alanine/Glycine	9.3	1.0	9.7	$5.0^{(1)}$
		Valine/Glycine	3.5	1.2	3.2	$4.5^{(1)}$
	A14	Control	0.0	0.0	0.0	
		Glutamate	6.2	0.9	6.2	$4.8^{(2)}$

[a]Only phylotypes that were significantly stimulated (based on LEfSe analyses) by a given supplement are shown. The LEfSe analysis, mean value, standard deviation, and median are based on the relative abundance of 16S rRNA sequences of the three replicates per treatment at the end of the incubation.

[b]A, phylotypes in amino acid experiment; T, phylotypes in transient intermediate experiment; R, phylotypes in ribose experiment.

[c]LDA scores were calculated using LEfSe. Numbers in parentheses display the rank in the LDA analysis (i.e., higher ranking phylotypes exhibited a stronger response to supplement compared to lower ranking ones).

Table S11. Summary of all detected families in control and amino acid treatments based on 16S rRNA gene (A) and 16S rRNA (B) analysis.[a]

(A) 16S rRNA genes

Sampling Time:	0 h							10 h						
Treatment:	C	CAA	Glu	Asp	Thr	Ala/Gly	Val/Gly	C	CAA	Glu	Asp	Thr	Ala/Gly	Val/Gly
**Phyla, Class, Family[b]**	Relative Abundance (%)													
Acidobacteria,														
Acidobacteria,														
Acidobacteriaceae (16)	0.1	0.0	0.1	0.1	0.1	0.0	0.1	0.1	0.0	0.0	0.1	0.1	0.0	0.1
Blastocatellia,														
Blastocatellaceae (23)	0.2	0.1	0.1	0.1	0.1	0.1	0.1	0.2	0.1	0.1	0.1	0.1	0.1	0.1
Holophagae,														
Unassigned *Holophagae* (12)	0.1	0.1	0.1	0.1	0.1	0.1	0.1	0.1	0.1	0.1	0.1	0.1	0.1	0.1
Solibacteres,														
Solibacteraceae (34)	0.2	0.2	0.2	0.2	0.2	0.2	0.2	0.1	0.1	0.2	0.1	0.2	0.2	0.2
Subgroup_11 (5)	0.0	0.0	0.0	0.0	0.0	0.0	0.0	0.1	0.0	0.0	0.0	0.0	0.0	0.0
Subgroup_17 (16)	0.1	0.1	0.1	0.1	0.1	0.1	0.1	0.0	0.0	0.1	0.1	0.1	0.1	0.1
Subgroup_22 (16)	0.0	0.0	0.0	0.0	0.0	0.0	0.0	0.0	0.0	0.0	0.0	0.0	0.0	0.0
Subgroup_25 (9)	0.1	0.1	0.1	0.1	0.1	0.1	0.1	0.0	0.0	0.0	0.0	0.0	0.1	0.0
Subgroup_5 (10)	0.1	0.1	0.1	0.1	0.1	0.1	0.1	0.2	0.1	0.1	0.1	0.1	0.1	0.1
Subgroup_6 (113)	2.0	1.7	1.8	1.7	1.7	1.6	1.7	1.0	1.0	1.3	1.4	1.5	1.8	1.6
Unassigned *Acidobacteria* (8)	0.0	0.0	0.0	0.0	0.0	0.0	0.0	0.0	0.0	0.0	0.0	0.0	0.0	0.0
Actinobacteria,														
Acidimicrobiia,														
Acidimicrobiaceae (30)	1.3	1.3	1.3	1.3	1.3	1.4	1.3	0.8	0.7	0.9	1.1	1.2	1.3	1.4
Iamiaceae (12)	0.2	0.2	0.2	0.2	0.2	0.2	0.2	0.1	0.1	0.1	0.1	0.2	0.2	0.2
Unassigned *Acidimicrobiales* (70)	1.9	1.8	1.8	1.7	1.8	1.8	1.7	1.3	1.0	1.3	1.5	1.5	1.8	1.8
Actinobacteria,														
Actinospicaceae (1)	0.0	0.0	0.0	0.0	0.0	0.0	0.0	0.0	0.0	0.0	0.0	0.0	0.0	0.0
Catenulisporaceae (1)	0.0	0.0	0.0	0.0	0.0	0.0	0.0	0.0	0.0	0.0	0.0	0.0	0.0	0.0
Mycobacteriaceae (12)	1.3	1.1	1.1	1.1	1.0	1.2	1.2	0.7	0.7	0.7	0.9	1.0	1.0	1.0
Nocardiaceae (12)	0.1	0.0	0.1	0.1	0.0	0.1	0.1	0.0	0.0	0.0	0.1	0.1	0.1	0.1
Acidothermaceae (10)	0.2	0.3	0.2	0.2	0.2	0.3	0.2	0.2	0.1	0.1	0.1	0.2	0.2	0.2
Cryptosporangiaceae (2)	0.0	0.0	0.0	0.0	0.0	0.0	0.0	0.0	0.0	0.0	0.0	0.0	0.0	0.0
Frankiaceae (5)	0.1	0.1	0.1	0.1	0.1	0.1	0.1	0.1	0.1	0.1	0.1	0.1	0.1	0.1
Geodermatophilaceae (3)	0.0	0.0	0.0	0.0	0.0	0.0	0.0	0.0	0.0	0.0	0.0	0.0	0.0	0.0
Nakamurellaceae (5)	0.2	0.2	0.2	0.2	0.2	0.2	0.2	0.1	0.1	0.1	0.1	0.2	0.2	0.2
Sporichthyaceae (6)	0.1	0.1	0.1	0.1	0.1	0.1	0.1	0.1	0.0	0.1	0.1	0.1	0.1	0.1
Glycomycetaceae (2)	0.0	0.0	0.0	0.0	0.0	0.0	0.0	0.0	0.0	0.0	0.0	0.0	0.0	0.0
Kineosporiaceae (5)	0.0	0.0	0.0	0.0	0.0	0.0	0.0	0.0	0.0	0.0	0.0	0.0	0.0	0.0
Beutenbergiaceae (1)	0.0	0.0	0.0	0.0	0.0	0.0	0.0	0.0	0.0	0.0	0.0	0.0	0.0	0.0
Brevibacteriaceae (1)	0.0	0.0	0.0	0.0	0.0	0.0	0.0	0.0	0.0	0.0	0.0	0.0	0.0	0.0
Cellulomonadaceae (1)	0.1	0.1	0.1	0.1	0.1	0.1	0.1	0.1	0.0	0.1	0.1	0.1	0.1	0.1
Demequinaceae (1)	0.0	0.0	0.0	0.0	0.0	0.0	0.0	0.0	0.0	0.0	0.0	0.0	0.0	0.0
Dermabacteraceae (1)	0.0	0.0	0.0	0.0	0.0	0.0	0.0	0.0	0.0	0.0	0.0	0.0	0.0	0.0
Dermacoccaceae (1)	0.0	0.0	0.0	0.0	0.0	0.0	0.0	0.0	0.0	0.0	0.0	0.0	0.0	0.0
Intrasporangiaceae (4)	0.3	0.3	0.3	0.3	0.3	0.3	0.3	0.2	0.2	0.2	0.3	0.3	0.4	0.4
Microbacteriaceae (15)	0.3	0.3	0.2	0.3	0.3	0.3	0.3	0.3	0.1	0.2	0.2	0.2	0.2	0.2
Micrococcaceae (4)	0.6	0.6	0.6	0.5	0.6	0.5	0.5	0.4	0.3	0.3	0.3	0.4	0.4	0.4
Promicromonosporaceae (1)	0.0	0.0	0.0	0.0	0.0	0.0	0.0	0.0	0.0	0.0	0.0	0.0	0.0	0.0
Sanguibacteraceae (1)	0.0	0.0	0.0	0.0	0.0	0.0	0.0	0.0	0.0	0.0	0.0	0.0	0.0	0.0
Micromonosporaceae (44)	1.1	1.0	1.0	1.0	1.0	1.0	1.0	0.7	0.6	0.7	0.9	0.9	1.1	1.0
Nocardioidaceae (41)	2.2	2.2	2.4	2.3	2.3	2.3	2.3	1.9	1.3	1.6	1.8	2.1	2.4	2.3

Sampling Time:	0 h							10 h						
Treatment:	C	CAA	Glu	Asp	Thr	Ala/Gly	Val/Gly	C	CAA	Glu	Asp	Thr	Ala/Gly	Val/Gly
**Phyla, Class, Family**[b]	Relative Abundance (%)													
Propionibacteriaceae (11)	0.3	0.3	0.3	0.3	0.3	0.3	0.3	0.3	0.2	0.1	0.2	0.2	0.3	0.3
Pseudonocardiaceae (20)	0.6	0.5	0.5	0.5	0.5	0.6	0.5	0.6	0.3	0.4	0.5	0.5	0.7	0.6
Streptomycetaceae (5)	0.7	0.7	0.7	0.7	0.7	0.7	0.7	0.6	0.4	0.5	0.5	0.6	0.7	0.7
Nocardiopsaceae (1)	0.0	0.0	0.0	0.0	0.0	0.0	0.0	0.0	0.0	0.0	0.0	0.0	0.0	0.0
Streptosporangiaceae (4)	0.1	0.1	0.1	0.1	0.1	0.1	0.1	0.1	0.0	0.1	0.1	0.1	0.1	0.1
Thermomonosporaceae (15)	0.1	0.0	0.0	0.1	0.1	0.0	0.1	0.1	0.0	0.0	0.0	0.0	0.0	0.1
Unassigned *Frankiales* (2)	0.0	0.0	0.0	0.0	0.0	0.0	0.0	0.0	0.0	0.0	0.0	0.0	0.0	0.0
Unassigned *Actinobacteria* (2)	0.1	0.1	0.1	0.1	0.1	0.1	0.1	0.0	0.0	0.0	0.1	0.1	0.1	0.1
Coriobacteriia,														
Coriobacteriaceae (1)	0.0	0.0	0.0	0.0	0.0	0.0	0.0	0.0	0.0	0.0	0.0	0.0	0.0	0.0
Rubrobacteria,														
Rubrobacteriaceae (6)	0.1	0.1	0.1	0.1	0.1	0.1	0.1	0.1	0.1	0.1	0.1	0.1	0.1	0.1
Thermoleophilia,														
Gaiellaceae (11)	2.3	2.3	2.3	2.2	2.3	2.4	2.3	2.1	1.2	1.5	1.8	1.9	2.4	2.4
Unassigned *Gaiellales* (72)	4.7	4.7	4.8	4.4	4.5	4.6	4.5	3.1	2.5	2.9	3.8	3.8	4.5	4.4
Conexibacteraceae (3)	0.0	0.0	0.0	0.0	0.0	0.0	0.0	0.1	0.0	0.0	0.0	0.0	0.0	0.0
Parviterribacteraceae (3)	0.0	0.0	0.0	0.0	0.0	0.0	0.0	0.0	0.0	0.0	0.0	0.0	0.0	0.0
Patulibacteraceae (15)	0.0	0.0	0.0	0.0	0.0	0.0	0.0	0.0	0.0	0.0	0.0	0.0	0.1	0.0
Solirubrobacteraceae (15)	0.6	0.6	0.7	0.6	0.6	0.6	0.7	0.7	0.4	0.5	0.6	0.7	0.8	0.8
Unassigned *Solirubrobacterales* (26)	0.7	0.6	0.7	0.7	0.7	0.7	0.7	0.6	0.4	0.6	0.7	0.7	0.8	0.8
Unassigned *Thermoleophilia* (67)	2.4	2.5	2.3	2.4	2.3	2.2	2.6	2.0	1.5	1.8	2.1	2.1	2.4	2.2
Unassigned *Actinobacteria* (34)	2.1	2.0	2.1	1.9	1.9	2.1	2.0	1.1	1.0	1.2	1.5	1.5	1.7	1.7
Armatimonadetes,														
Armatimonadia,														
Unassigned *Armatimonadales* (3)	0.0	0.0	0.0	0.0	0.0	0.0	0.0	0.0	0.0	0.0	0.0	0.0	0.0	0.0
Chthonomonadaceae (2)	0.0	0.0	0.0	0.0	0.0	0.0	0.0	0.0	0.0	0.0	0.0	0.0	0.0	0.0
Unassigned *Chthonomonadales* (2)	0.0	0.0	0.0	0.0	0.0	0.0	0.0	0.0	0.0	0.0	0.0	0.0	0.0	0.0
Unassigned *Armatimonadetes* (21)	0.0	0.0	0.0	0.0	0.0	0.0	0.0	0.0	0.0	0.0	0.0	0.0	0.0	0.0
Bacteroidetes,														
Bacteroidia,														
Prolixibacteraceae (1)	0.0	0.0	0.0	0.0	0.0	0.0	0.0	0.0	0.0	0.0	0.0	0.0	0.0	0.0
Cytophagia (2)	0.0	0.0	0.0	0.0	0.0	0.0	0.0	0.0	0.0	0.0	0.0	0.0	0.0	0.0
Cytophagaceae (33)	0.0	0.0	0.0	0.0	0.0	0.1	0.0	0.1	0.0	0.0	0.0	0.0	0.0	0.0
Flammeovirgaceae (1)	0.0	0.0	0.0	0.0	0.0	0.0	0.0	0.0	0.0	0.0	0.0	0.0	0.0	0.0
Flavobacteriia,														
Flavobacteriaceae (21)	0.6	0.6	0.6	0.6	0.5	0.4	0.5	0.7	0.3	0.4	0.5	0.5	0.2	0.3
Sphingobacteriia,														
Chitinophagaceae (48)	0.1	0.1	0.1	0.2	0.1	0.1	0.1	0.1	0.1	0.1	0.1	0.1	0.1	0.1
Lentimicrobiaceae (1)	0.0	0.0	0.0	0.0	0.0	0.0	0.0	0.0	0.0	0.0	0.0	0.0	0.0	0.0
Saprospiraceae (2)	0.0	0.0	0.0	0.0	0.0	0.0	0.0	0.0	0.0	0.0	0.0	0.0	0.0	0.0
Sphingobacteriaceae (3)	0.0	0.0	0.0	0.0	0.0	0.0	0.0	0.0	0.0	0.0	0.0	0.0	0.0	0.0
Unassigned *Sphingobacteriales* (14)	0.0	0.0	0.0	0.0	0.0	0.0	0.0	0.0	0.0	0.0	0.0	0.0	0.0	0.0
Unassigned *Sphingobacteriia* (10)	0.0	0.0	0.0	0.0	0.0	0.0	0.0	0.0	0.0	0.0	0.0	0.0	0.0	0.0
Unassigned *Bacteroidetes* (3)	0.0	0.0	0.0	0.0	0.0	0.0	0.0	0.0	0.0	0.0	0.0	0.0	0.0	0.0
BJ-169,														
Unassigned *BJ-169* (6)	0.0	0.0	0.0	0.0	0.0	0.0	0.0	0.0	0.0	0.0	0.0	0.0	0.0	0.0
BRC1,														
Unassigned *BCR1* (14)	0.0	0.0	0.0	0.0	0.0	0.0	0.0	0.0	0.0	0.0	0.0	0.0	0.0	0.0
Chlamydiae,														
Chlamydiae,														

Sampling Time:	0 h							10 h						
Treatment:	C	CAA	Glu	Asp	Thr	Ala/Gly	Val/Gly	C	CAA	Glu	Asp	Thr	Ala/Gly	Val/Gly
**Phyla, Class, Family**[b]	Relative Abundance (%)													
Chlamydiaceae (4)	0.0	0.0	0.0	0.0	0.0	0.0	0.0	0.0	0.0	0.0	0.0	0.0	0.0	0.0
Parachlamydiaceae (154)	0.1	0.1	0.1	0.2	0.1	0.1	0.1	0.1	0.0	0.1	0.1	0.1	0.1	0.1
Simkaniaceae (11)	0.0	0.0	0.0	0.0	0.0	0.0	0.0	0.0	0.0	0.0	0.0	0.0	0.0	0.0
Waddliaceae (1)	0.0	0.0	0.0	0.0	0.0	0.0	0.0	0.0	0.0	0.0	0.0	0.0	0.0	0.0
Unassigned *Chlamydiales* (7)	0.0	0.0	0.0	0.0	0.0	0.0	0.0	0.0	0.0	0.0	0.0	0.0	0.0	0.0
Chlorobi,														
Chlorobia,														
Unassigned *Chlorobia* (4)	0.0	0.0	0.0	0.0	0.0	0.0	0.0	0.0	0.0	0.0	0.0	0.0	0.0	0.0
Chloroflexi,														
Anaerolineae,														
Anaerolineaceae (14)	0.0	0.0	0.0	0.0	0.0	0.0	0.0	0.0	0.0	0.0	0.0	0.0	0.0	0.0
Unassigned *Ardenticatenia* (3)	0.0	0.0	0.0	0.0	0.0	0.0	0.0	0.0	0.0	0.0	0.0	0.0	0.0	0.0
Caldilineae,														
Caldilineaceae (15)	0.1	0.1	0.1	0.1	0.1	0.1	0.1	0.1	0.1	0.1	0.1	0.1	0.1	0.1
Chloroflexia,														
Roseiflexaceae (8)	0.2	0.2	0.2	0.2	0.2	0.2	0.2	0.0	0.1	0.1	0.2	0.2	0.2	0.2
Unassigned *Chloroflexia* (4)	0.0	0.0	0.0	0.0	0.0	0.0	0.0	0.0	0.0	0.0	0.0	0.0	0.0	0.0
Ktedonobacteria,														
Ktedonobacterales,														
Ktedonobacteraceae (9)	0.0	0.0	0.0	0.0	0.0	0.0	0.0	0.0	0.0	0.0	0.0	0.0	0.0	0.0
Thermosporotrichaceae (7)	0.0	0.0	0.0	0.0	0.0	0.0	0.0	0.0	0.0	0.0	0.0	0.0	0.0	0.0
Unassigned *Ktedonobacterales* (6)	0.0	0.0	0.0	0.0	0.0	0.0	0.0	0.0	0.0	0.0	0.0	0.0	0.0	0.0
Unassigned *Ktedonobacteria* (18)	0.2	0.1	0.1	0.1	0.1	0.1	0.1	0.1	0.1	0.1	0.1	0.1	0.1	0.1
Thermomicrobia,														
Thermomicrobiaceae (1)	0.0	0.0	0.0	0.0	0.0	0.0	0.0	0.0	0.0	0.0	0.0	0.0	0.0	0.0
Unassigned *Thermomicrobia* (56)	0.5	0.5	0.4	0.4	0.4	0.4	0.4	0.2	0.2	0.3	0.3	0.3	0.4	0.4
Unassigned *Chloroflexi* (111)	2.1	2.1	2.0	2.0	2.1	2.1	2.0	1.5	1.1	1.4	1.7	1.8	2.1	2.0
Cyanobacteria,														
Chloroplast,														
Unassigned *Chloroplast* (34)	0.1	0.0	0.0	0.0	0.0	0.0	0.0	0.0	0.0	0.0	0.0	0.0	0.0	0.0
Cyanobacteria,														
Unassigned *Cyanobacteria* (2)	0.0	0.0	0.0	0.0	0.0	0.0	0.0	0.0	0.0	0.0	0.0	0.0	0.0	0.0
Gastranaerophilales,														
Unassigned *Gastranaerophilales* (1)	0.0	0.0	0.0	0.0	0.0	0.0	0.0	0.0	0.0	0.0	0.0	0.0	0.0	0.0
Obscuribacterales,														
Unassigned *Obscuribacterales* (6)	0.0	0.0	0.0	0.0	0.0	0.0	0.0	0.0	0.0	0.0	0.0	0.0	0.0	0.0
Unassigned *Cyanobacteria* (11)	0.0	0.0	0.0	0.0	0.0	0.0	0.0	0.0	0.0	0.0	0.0	0.0	0.0	0.0
Deferribacteres,														
Unassigned *Deferribacteres* (1)	0.0	0.0	0.0	0.0	0.0	0.0	0.0	0.0	0.0	0.0	0.0	0.0	0.0	0.0
Deinococcus-Thermus,														
Deinococci,														
Unassigned *Deinococci* (4)	0.0	0.0	0.0	0.0	0.0	0.0	0.0	0.0	0.0	0.0	0.0	0.0	0.0	0.0
Elusimicrobia,														
Unassigned *Elusimicrobia,*	0.0	0.0	0.0	0.0	0.0	0.0	0.0	0.0	0.0	0.0	0.0	0.0	0.0	0.0
Euryarchaeota,														
Methanomicrobia,														
Methanosarcinaceae (1)	0.0	0.0	0.0	0.0	0.0	0.0	0.0	0.0	0.0	0.0	0.0	0.0	0.0	0.0
Thermoplasmata,														
Unassinged Thermoplasmatales (1)	0.0	0.0	0.0	0.0	0.0	0.0	0.0	0.0	0.0	0.0	0.0	0.0	0.0	0.0

Sampling Time:	0 h							10 h						
Treatment:	C	CAA	Glu	Asp	Thr	Ala/Gly	Val/Gly	C	CAA	Glu	Asp	Thr	Ala/Gly	Val/Gly
**Phyla, Class, Family[b]**	**Relative Abundance (%)**													
Fibrobacteres,														
Fibrobacteria,														
Fibrobacteraceae (5)	0.0	0.0	0.0	0.0	0.0	0.0	0.0	0.0	0.0	0.0	0.0	0.0	0.0	0.0
Unassigned *Fibrobacterales* (3)	0.0	0.0	0.0	0.0	0.0	0.0	0.0	0.0	0.0	0.0	0.0	0.0	0.0	0.0
Firmicutes,														
Bacilli,														
Alicyclobacillaceae (5)	0.0	0.0	0.0	0.0	0.0	0.0	0.0	0.0	0.0	0.0	0.0	0.0	0.0	0.0
Bacillaceae (38)	0.8	0.8	0.8	0.8	0.7	0.7	0.7	0.5	0.4	0.5	0.5	0.6	0.7	0.6
Paenibacillaceae (88)	0.2	0.2	0.2	0.2	0.2	0.2	0.2	0.2	0.1	0.1	0.2	0.1	0.2	0.2
Pasteuriaceae (6)	0.0	0.0	0.0	0.0	0.0	0.0	0.0	0.0	0.0	0.0	0.0	0.0	0.0	0.0
Planococcaceae (7)	0.1	0.1	0.1	0.1	0.1	0.1	0.1	0.0	0.0	0.1	0.1	0.1	0.1	0.1
Sporolactobacillaceae (1)	0.0	0.0	0.0	0.0	0.0	0.0	0.0	0.0	0.0	0.0	0.0	0.0	0.0	0.0
Staphylococcaceae (1)	0.0	0.0	0.0	0.0	0.0	0.0	0.0	0.0	0.0	0.0	0.0	0.0	0.0	0.0
Thermoactinomycetaceae (18)	0.0	0.0	0.0	0.0	0.0	0.0	0.0	0.0	0.0	0.0	0.0	0.0	0.0	0.0
Streptococcaceae (1)	0.0	0.0	0.0	0.0	0.0	0.0	0.0	0.0	0.0	0.0	0.0	0.0	0.0	0.0
Unassigned *Bacilli* (13)	0.7	0.7	0.7	0.8	0.6	0.7	0.7	0.3	0.2	0.3	0.4	0.3	0.4	0.4
Clostridia,														
Caldicoprobacteraceae (7)	0.0	0.0	0.0	0.0	0.0	0.0	0.0	0.0	0.0	0.0	0.0	0.0	0.0	0.0
Christensenellaceae (7)	0.0	0.0	0.0	0.0	0.0	0.0	0.0	0.0	0.0	0.0	0.0	0.0	0.0	0.0
Clostridiaceae (38) **[A14]**	0.2	0.2	0.2	0.2	0.2	0.2	0.2	0.3	0.2	0.2	0.2	0.2	0.3	0.2
Defluviitaleaceae (4)	0.0	0.0	0.0	0.0	0.0	0.0	0.0	0.0	0.0	0.0	0.0	0.0	0.0	0.0
Eubacteriaceae (4)	0.0	0.0	0.0	0.0	0.0	0.0	0.0	0.0	0.0	0.0	0.0	0.0	0.0	0.0
Gracilibacteraceae (7)	0.0	0.0	0.0	0.0	0.0	0.0	0.0	0.0	0.0	0.0	0.0	0.0	0.0	0.0
Heliobacteriaceae (22)	0.0	0.0	0.0	0.0	0.0	0.0	0.0	0.0	0.0	0.0	0.0	0.0	0.0	0.0
Lachnospiraceae (79)	0.1	0.1	0.1	0.1	0.1	0.1	0.1	0.2	0.0	0.1	0.1	0.1	0.1	0.1
Peptococcaceae (17)	2.5	2.5	2.9	2.9	2.5	2.1	2.1	1.5	1.2	1.4	1.6	1.6	2.0	1.9
Peptostreptococcaceae (11) **[GPT-4], [A8]**	0.1	0.1	0.1	0.1	0.1	0.1	0.1	0.4	0.5	0.2	0.2	0.3	0.3	0.3
Ruminococcaceae (96)	0.1	0.1	0.1	0.1	0.1	0.1	0.1	0.1	0.0	0.1	0.1	0.1	0.1	0.1
Syntrophomonadaceae (10)	0.0	0.0	0.0	0.0	0.0	0.0	0.0	0.0	0.0	0.0	0.0	0.0	0.0	0.0
Thermoanaerobacteraceae (8)	0.0	0.0	0.0	0.0	0.0	0.0	0.0	0.0	0.0	0.0	0.0	0.0	0.0	0.0
Unassigned *Clostridiales* (17)	0.0	0.0	0.0	0.0	0.0	0.0	0.0	0.0	0.0	0.0	0.0	0.0	0.0	0.0
Unassigned *Thermoanaerobacterales* (1)	0.0	0.0	0.0	0.0	0.0	0.0	0.0	0.0	0.0	0.0	0.0	0.0	0.0	0.0
Unassigned *Clostridia* (34)	0.0	0.0	0.0	0.0	0.0	0.0	0.0	0.0	0.0	0.0	0.0	0.0	0.0	0.0
Erysipelotrichia,														
Erysipelotrichaceae (18)	0.0	0.0	0.0	0.0	0.0	0.0	0.0	0.0	0.0	0.0	0.0	0.0	0.0	0.0
Limnochordia,														
Limnochordaceae (23)	0.0	0.0	0.0	0.0	0.0	0.0	0.0	0.0	0.0	0.0	0.0	0.0	0.0	0.0
Unassigned *Limnochordales* (3)	0.0	0.0	0.0	0.0	0.0	0.0	0.0	0.0	0.0	0.0	0.0	0.0	0.0	0.0
Negativicutes,														
Veillonellaceae (20)	0.0	0.0	0.0	0.0	0.0	0.0	0.0	0.0	0.0	0.0	0.0	0.0	0.0	0.0
Unassigned *Selenomonadales* (3)	0.0	0.0	0.0	0.0	0.0	0.0	0.0	0.0	0.0	0.0	0.0	0.0	0.0	0.0
Unassigned *Firmicutes* (1)	0.0	0.0	0.0	0.0	0.0	0.0	0.0	0.0	0.0	0.0	0.0	0.0	0.0	0.0
Fusobacteria,														
Fusobacteriia,														
Fusobacteriaceae (10) **[GPT-5]**	10	11	11	12	13	14	14	21	38	36	22	19	14	17
Leptotrichiaceae (1)	0.0	0.0	0.0	0.0	0.0	0.0	0.0	0.0	0.0	0.0	0.0	0.0	0.0	0.0
Gemmatimonadetes,														
Gemmatimonadetes,														
Gemmatimonadaceae (37)	0.6	0.5	0.5	0.5	0.5	0.5	0.5	0.3	0.3	0.4	0.4	0.4	0.5	0.5
Longimicrobiaceae (1)	0.0	0.0	0.0	0.0	0.0	0.0	0.0	0.0	0.0	0.0	0.0	0.0	0.0	0.0

Sampling Time:	0 h						
Treatment:	C	CAA	Glu	Asp	Thr	Ala/Gly	Val/Gly
Phyla, Class, Family[b]	Relative Abundance (%)						
Unassigned *Gemmatimonadetes* (6)	0.0	0.0	0.0	0.0	0.0	0.0	0.0
Hydrogenedentes,							
Unassigned *Hydrogenedentes* (2)	0.0	0.0	0.0	0.0	0.0	0.0	0.0
Latescibacteria,							
Unassigned *Latescibacteria* (30)	0.0	0.0	0.0	0.0	0.0	0.0	0.0
Nitrospirae,							
Nitrospira,							
Nitrospiraceae (8)	0.2	0.2	0.2	0.2	0.2	0.2	0.2
Unassigned *Nitrospira* (18)	0.7	0.7	0.6	0.6	0.6	0.6	0.6
Parcubacteria,							
Unassigned *Parcubacteria* (1)	0.0	0.0	0.0	0.0	0.0	0.0	0.0
Planctomycetes,							
Phycisphaerae,							
Phycisphaeraceae (38)	0.0	0.0	0.0	0.0	0.0	0.0	0.0
Tepidisphaeraceae (82)	0.8	0.6	0.7	0.6	0.6	0.6	0.6
Unassigned *Phycisphaerales* (4)	0.0	0.0	0.0	0.0	0.0	0.0	0.0
Unassigned *Phycisphaerae* (7)	0.0	0.0	0.0	0.0	0.0	0.0	0.0
Planctomycetacia,							
Planctomycetaceae (733)	4.5	4.0	4.1	3.8	4.0	4.0	3.7
Unassigned *Planctomycetes* (82)	0.1	0.1	0.1	0.1	0.1	0.1	0.1
Proteobacteria,							
Alphaproteobacteria,							
Caulobacteraceae (5)	0.0	0.0	0.0	0.0	0.0	0.0	0.0
Beijerinckiaceae (2)	0.0	0.0	0.0	0.0	0.0	0.0	0.0
Bradyrhizobiaceae (5)	1.2	1.2	1.1	1.1	1.0	1.2	1.0
Hyphomicrobiaceae (8)	0.3	0.4	0.3	0.3	0.3	0.3	0.3
Methylobacteriaceae (4)	0.1	0.2	0.1	0.1	0.1	0.2	0.1
Methylocystaceae (1)	0.0	0.0	0.0	0.0	0.0	0.0	0.0
Phyllobacteriaceae (3)	0.2	0.2	0.2	0.2	0.2	0.2	0.2
Rhizobiaceae (3)	0.0	0.0	0.0	0.0	0.0	0.0	0.0
Rhodobiaceae (2)	1.3	1.2	1.2	1.3	1.3	1.2	1.1
Roseiarcaceae (1)	0.0	0.0	0.0	0.0	0.0	0.0	0.0
Xanthobacteraceae (9)	3.7	3.4	3.4	3.4	3.3	3.2	3.1
Unassigned *Rhizobiales* (19)	0.3	0.3	0.2	0.3	0.3	0.3	0.3
Rhodobacteraceae (6)	0.0	0.0	0.0	0.0	0.0	0.0	0.0
Acetobacteraceae (15)	0.1	0.1	0.1	0.1	0.1	0.1	0.1
Rhodospirillaceae (19)	0.5	0.4	0.4	0.3	0.3	0.4	0.4
Unassigned *Rhodospirillales* (25)	0.6	0.6	0.6	0.5	0.5	0.6	0.5
Anaplasmataceae (1)	0.0	0.0	0.0	0.0	0.0	0.0	0.0
Holosporaceae (2)	0.0	0.0	0.0	0.0	0.0	0.0	0.0
Mitochondria (2)	0.0	0.0	0.0	0.0	0.0	0.0	0.0
Rickettsiaceae (1)	0.0	0.0	0.0	0.0	0.0	0.0	0.0
Unassigned *Rickettsiales* (7)	0.0	0.0	0.0	0.0	0.0	0.0	0.0
Erythrobacteraceae (1)	0.0	0.0	0.0	0.0	0.0	0.0	0.0
Sphingomonadaceae (5)	0.0	0.0	0.0	0.0	0.0	0.0	0.0
Unassigned *Sphingomonadales* (2)	0.0	0.0	0.0	0.0	0.0	0.0	0.0
Unassigned *Alphaproteobacteria* (19)	0.2	0.1	0.1	0.1	0.1	0.1	0.1
Betaproteobacteria,							
Alcaligenaceae (2)	0.1	0.1	0.1	0.0	0.0	0.0	0.1

Sampling Time:	10 h						
Treatment:	C	CAA	Glu	Asp	Thr	Ala/Gly	Val/Gly
Phyla, Class, Family[b]	Relative Abundance (%)						
Unassigned *Gemmatimonadetes* (6)	0.0	0.0	0.0	0.0	0.0	0.0	0.0
Hydrogenedentes,							
Unassigned *Hydrogenedentes* (2)	0.0	0.0	0.0	0.0	0.0	0.0	0.0
Latescibacteria,							
Unassigned *Latescibacteria* (30)	0.0	0.0	0.0	0.0	0.0	0.0	0.0
Nitrospirae,							
Nitrospira,							
Nitrospiraceae (8)	0.1	0.1	0.1	0.1	0.1	0.2	0.2
Unassigned *Nitrospira* (18)	0.5	0.3	0.4	0.4	0.5	0.6	0.5
Parcubacteria,							
Unassigned *Parcubacteria* (1)	0.0	0.0	0.0	0.0	0.0	0.0	0.0
Planctomycetes,							
Phycisphaerae,							
Phycisphaeraceae (38)	0.0	0.0	0.0	0.0	0.0	0.0	0.0
Tepidisphaeraceae (82)	0.2	0.3	0.4	0.5	0.5	0.6	0.5
Unassigned *Phycisphaerales* (4)	0.0	0.0	0.0	0.0	0.0	0.0	0.0
Unassigned *Phycisphaerae* (7)	0.1	0.0	0.0	0.0	0.0	0.0	0.0
Planctomycetacia,							
Planctomycetaceae (733)	2.2	2.3	2.5	3.0	3.3	3.9	3.7
Unassigned *Planctomycetes* (82)	0.0	0.0	0.1	0.1	0.1	0.1	0.1
Proteobacteria,							
Alphaproteobacteria,							
Caulobacteraceae (5)	0.0	0.0	0.0	0.0	0.0	0.0	0.0
Beijerinckiaceae (2)	0.0	0.0	0.0	0.0	0.0	0.0	0.0
Bradyrhizobiaceae (5)	0.7	0.6	0.7	0.9	0.9	1.1	1.2
Hyphomicrobiaceae (8)	0.1	0.2	0.2	0.3	0.2	0.3	0.3
Methylobacteriaceae (4)	0.2	0.1	0.1	0.1	0.1	0.1	0.1
Methylocystaceae (1)	0.0	0.0	0.0	0.0	0.0	0.0	0.0
Phyllobacteriaceae (3)	0.1	0.1	0.1	0.2	0.2	0.2	0.2
Rhizobiaceae (3)	0.0	0.0	0.0	0.0	0.0	0.0	0.0
Rhodobiaceae (2)	0.9	0.6	0.8	1.0	0.9	1.2	1.1
Roseiarcaceae (1)	0.0	0.0	0.0	0.0	0.0	0.0	0.0
Xanthobacteraceae (9)	2.1	1.7	2.0	2.5	2.6	3.2	3.1
Unassigned *Rhizobiales* (19)	0.2	0.1	0.2	0.2	0.2	0.3	0.2
Rhodobacteraceae (6)	0.0	0.0	0.0	0.0	0.0	0.0	0.1
Acetobacteraceae (15)	0.1	0.1	0.1	0.1	0.1	0.1	0.1
Rhodospirillaceae (19)	0.2	0.2	0.2	0.3	0.3	0.3	0.3
Unassigned *Rhodospirillales* (25)	0.5	0.3	0.4	0.5	0.5	0.6	0.6
Anaplasmataceae (1)	0.0	0.0	0.0	0.0	0.0	0.0	0.0
Holosporaceae (2)	0.0	0.0	0.0	0.0	0.0	0.0	0.0
Mitochondria (2)	0.0	0.0	0.0	0.0	0.0	0.0	0.0
Rickettsiaceae (1)	0.0	0.0	0.0	0.0	0.0	0.0	0.0
Unassigned *Rickettsiales* (7)	0.0	0.0	0.0	0.0	0.0	0.0	0.0
Erythrobacteraceae (1)	0.0	0.0	0.0	0.0	0.0	0.0	0.0
Sphingomonadaceae (5)	0.1	0.0	0.0	0.0	0.0	0.0	0.0
Unassigned *Sphingomonadales* (2)	0.0	0.0	0.0	0.0	0.0	0.0	0.0
Unassigned *Alphaproteobacteria* (19)	0.1	0.1	0.1	0.1	0.1	0.1	0.1
Betaproteobacteria,							
Alcaligenaceae (2)	0.0	0.0	0.0	0.0	0.0	0.1	0.1

Sampling Time:	0 h							10 h						
Treatment:	C	CAA	Glu	Asp	Thr	Ala/Gly	Val/Gly	C	CAA	Glu	Asp	Thr	Ala/Gly	Val/Gly
**Phyla, Class, Family[b]**	**Relative Abundance (%)**													
Burkholderiaceae (10)	0.0	0.0	0.0	0.0	0.0	0.0	0.0	1.0	0.0	0.0	0.0	0.0	0.0	0.0
Comamonadaceae (17)	0.1	0.1	0.1	0.1	0.1	0.1	0.1	0.1	0.1	0.1	0.1	0.1	0.1	0.2
Oxalobacteraceae (15)	0.0	0.0	0.0	0.0	0.0	0.0	0.0	0.0	0.0	0.0	0.0	0.0	0.0	0.0
Neisseriaceae (1)	0.0	0.0	0.0	0.0	0.0	0.0	0.0	0.0	0.0	0.0	0.0	0.0	0.0	0.0
Gallionellaceae (3)	0.0	0.0	0.0	0.0	0.0	0.0	0.0	0.0	0.0	0.0	0.0	0.0	0.0	0.0
Nitrosomonadaceae (30)	0.3	0.3	0.3	0.3	0.2	0.3	0.2	0.3	0.2	0.2	0.2	0.2	0.3	0.3
Rhodocyclaceae (9)	0.1	0.1	0.1	0.1	0.1	0.1	0.1	0.1	0.0	0.0	0.1	0.1	0.1	0.1
Unassigned *Betaproteobacteria* (41)	0.3	0.3	0.3	0.3	0.3	0.3	0.3	0.3	0.1	0.2	0.2	0.2	0.3	0.3
Deltaproteobacteria,														
Bacteriovoracaceae (12)	0.0	0.0	0.0	0.0	0.0	0.0	0.0	0.0	0.0	0.0	0.0	0.0	0.0	0.0
Bdellovibrionaceae (54)	0.0	0.0	0.0	0.0	0.0	0.0	0.0	0.0	0.0	0.0	0.0	0.0	0.1	0.0
Desulfarculaceae (1)	0.0	0.0	0.0	0.0	0.0	0.0	0.0	0.0	0.0	0.0	0.0	0.0	0.0	0.0
Desulfobulbaceae (3)	0.0	0.0	0.0	0.0	0.0	0.0	0.0	0.0	0.0	0.0	0.0	0.0	0.0	0.0
Desulfurellaceae (41)	1.0	0.9	0.9	0.9	0.8	0.9	0.9	0.9	0.5	0.6	0.6	0.8	0.8	0.9
Desulfuromonadaceae (2)	0.0	0.0	0.0	0.0	0.0	0.0	0.0	0.0	0.0	0.0	0.0	0.0	0.0	0.0
Geobacteraceae (26)	0.2	0.2	0.2	0.1	0.1	0.2	0.1	0.0	0.1	0.1	0.1	0.1	0.1	0.1
Archangiaceae (20)	0.0	0.0	0.0	0.1	0.1	0.1	0.0	0.1	0.0	0.0	0.0	0.1	0.1	0.0
Haliangiaceae (74)	0.2	0.2	0.2	0.2	0.2	0.2	0.2	0.2	0.1	0.2	0.2	0.2	0.3	0.3
Myxococcaceae (3)	0.0	0.0	0.0	0.0	0.0	0.0	0.0	0.0	0.0	0.0	0.0	0.0	0.0	0.0
Nannocystaceae (6)	0.0	0.0	0.0	0.0	0.0	0.0	0.0	0.0	0.0	0.0	0.0	0.0	0.0	0.0
Phaselicystidaceae (14)	0.1	0.1	0.1	0.1	0.1	0.1	0.1	0.0	0.1	0.1	0.1	0.1	0.1	0.1
Polyangiaceae (48)	0.1	0.1	0.1	0.1	0.1	0.1	0.1	0.1	0.1	0.1	0.1	0.1	0.1	0.1
Sandaracinaceae (38)	0.1	0.1	0.1	0.1	0.1	0.1	0.1	0.1	0.0	0.0	0.1	0.1	0.1	0.1
Vulgatibacteraceae (3)	0.0	0.0	0.0	0.0	0.0	0.0	0.0	0.0	0.0	0.0	0.0	0.0	0.0	0.0
Unassigned *Myxococcales* (177)	0.2	0.2	0.2	0.2	0.2	0.2	0.2	0.1	0.1	0.2	0.2	0.2	0.2	0.2
Oligoflexaceae (29)	0.0	0.0	0.0	0.0	0.0	0.0	0.0	0.0	0.0	0.0	0.0	0.0	0.0	0.0
Unassigned *Oligoflexales* (86)	0.0	0.1	0.0	0.0	0.0	0.0	0.0	0.0	0.0	0.0	0.0	0.0	0.0	0.0
Syntrophaceae (1)	0.0	0.0	0.0	0.0	0.0	0.0	0.0	0.0	0.0	0.0	0.0	0.0	0.0	0.0
Unassigned *Deltaproteobacteria* (19)	0.0	0.0	0.0	0.0	0.0	0.0	0.0	0.0	0.0	0.0	0.0	0.0	0.0	0.0
Gammaproteobacteria,														
Acidiferrobacteraceae (3)	0.0	0.0	0.0	0.0	0.0	0.0	0.0	0.0	0.0	0.0	0.0	0.0	0.0	0.0
Aeromonadaceae (8) **[GPT-1]**	10	11	11	9.5	11	12	11	14	14	7.9	14	10	9.8	9.7
Shewanellaceae (3)	3.6	4.5	4.6	4.5	4.6	4.7	5.0	9.3	4.8	7.4	5.6	7.9	6.0	6.0
Cellvibrionaceae (2)	0.0	0.0	0.0	0.0	0.0	0.0	0.0	0.0	0.0	0.0	0.0	0.0	0.0	0.0
Halieaceae (4)	0.0	0.0	0.0	0.0	0.0	0.0	0.0	0.0	0.0	0.0	0.0	0.0	0.0	0.0
Enterobacteriaceae (12) **[GPT-2], [GPT-3]**	2.1	2.3	2.2	2.2	2.1	2.6	2.5	3.3	4.2	2.4	4.0	5.3	2.9	2.8
Coxiellaceae (35)	0.1	0.1	0.1	0.1	0.1	0.1	0.1	0.0	0.0	0.1	0.1	0.1	0.1	0.1
Legionellaceae (30)	0.0	0.0	0.0	0.0	0.0	0.0	0.0	0.0	0.0	0.0	0.0	0.0	0.0	0.0
Methylococcaceae (3)	0.0	0.0	0.0	0.0	0.0	0.0	0.0	0.0	0.0	0.0	0.0	0.0	0.0	0.0
Oleiphilaceae (1)	0.0	0.0	0.0	0.0	0.0	0.0	0.0	0.0	0.0	0.0	0.0	0.0	0.0	0.0
Moraxellaceae (2)	0.0	0.0	0.0	0.0	0.0	0.0	0.0	0.0	0.0	0.0	0.0	0.0	0.0	0.0
Pseudomonadaceae (4)	0.0	0.0	0.0	0.0	0.0	0.0	0.0	0.0	0.0	0.0	0.0	0.0	0.0	0.0
Unassigned Thiotrichales (1)	0.0	0.0	0.0	0.0	0.0	0.0	0.0	0.0	0.0	0.0	0.0	0.0	0.0	0.0
Xanthomonadaceae (12)	0.0	0.0	0.0	0.0	0.0	0.0	0.0	0.0	0.0	0.0	0.0	0.0	0.0	0.0
Unassigned *Xanthomonadales* (24)	0.3	0.3	0.3	0.3	0.3	0.3	0.3	0.1	0.2	0.2	0.2	0.3	0.3	0.3
Unassigned *Gammaproteobacteria* (30)	0.0	0.0	0.0	0.1	0.0	0.0	0.0	0.0	0.0	0.0	0.0	0.0	0.0	0.0
Unassigned *Proteobacteria* (2)	0.0	0.0	0.0	0.0	0.0	0.0	0.0	0.0	0.0	0.0	0.0	0.0	0.0	0.0
RsaHf231,														
Unassigned *RsaHf231* (1)	0.0	0.0	0.0	0.0	0.0	0.0	0.0	0.0	0.0	0.0	0.0	0.0	0.0	0.0
Saccharibacteria,														
Unassigned *Saccharibacteria* (78)	0.0	0.0	0.0	0.0	0.0	0.0	0.0	0.0	0.0	0.0	0.0	0.0	0.0	0.0

Sampling Time:	0 h							10 h						
Treatment:	C	CAA	Glu	Asp	Thr	Ala/Gly	Val/Gly	C	CAA	Glu	Asp	Thr	Ala/Gly	Val/Gly
**Phyla, Class, Family**[b]	Relative Abundance (%)													
Spirochaetae,														
Spirochaetes,														
Spirochaetaceae (1)	0.0	0.0	0.0	0.0	0.0	0.0	0.0	0.0	0.0	0.0	0.0	0.0	0.0	0.0
Brevinemataceae (1)	0.0	0.0	0.0	0.0	0.0	0.0	0.0	0.0	0.0	0.0	0.0	0.0	0.0	0.0
Synergistetes,														
Synergistia,														
Synergistaceae (1)	0.0	0.0	0.0	0.0	0.0	0.0	0.0	0.0	0.0	0.0	0.0	0.0	0.0	0.0
Tectomicrobia,														
Unassigned *Tectomicrobia* (21)	0.2	0.2	0.2	0.2	0.2	0.2	0.2	0.1	0.1	0.1	0.1	0.2	0.2	0.2
Tenericutes,														
Mollicutes,														
Unassigned *Entomoplasmatales* (3)	0.1	0.0	0.1	0.1	0.1	0.1	0.1	0.0	0.0	0.0	0.0	0.1	0.0	0.1
Haloplasmataceae (7)	0.0	0.0	0.0	0.0	0.0	0.0	0.0	0.0	0.0	0.0	0.0	0.0	0.0	0.0
Mycoplasmataceae (19)	13	13	12	14	13	9.7	12	10	7.5	7.7	8.9	10	11	10
Thaumarchaeota,														
Unassigned *Thaumarchaeota* (9)	0.0	0.0	0.0	0.0	0.0	0.0	0.0	0.0	0.0	0.0	0.0	0.0	0.0	0.0
TM6_Dependentiae,														
Unassigned *TM6_Dependentiae* (40)	0.0	0.0	0.0	0.0	0.0	0.0	0.0	0.0	0.0	0.0	0.0	0.0	0.0	0.0
Verrucomicrobia,														
Unassigned OPB35 soil group (87)	0.3	0.2	0.3	0.2	0.2	0.2	0.2	0.2	0.1	0.1	0.2	0.2	0.2	0.2
Opitutae,														
Opitutaceae (5)	0.0	0.0	0.0	0.0	0.0	0.0	0.0	0.0	0.0	0.0	0.0	0.0	0.0	0.0
Unassigned *Opitutae* (2)	0.0	0.0	0.0	0.0	0.0	0.0	0.0	0.0	0.0	0.0	0.0	0.0	0.0	0.0
Spartobacteria,														
Chthoniobacteraceae (46)	0.1	0.1	0.1	0.1	0.1	0.1	0.1	0.1	0.1	0.1	0.1	0.1	0.1	0.1
Unassigned *Chthoniobacterales* (2)	0.0	0.0	0.0	0.0	0.0	0.0	0.0	0.0	0.0	0.0	0.0	0.0	0.0	0.0
DA101 soil group (28)	3.4	3.5	3.4	3.6	3.3	3.3	3.3	2.1	1.7	2.3	2.6	2.7	3.1	3.0
Xiphinematobacteraceae (9)	1.2	1.1	1.1	1.1	1.0	1.2	1.1	0.5	0.7	0.8	0.9	1.0	1.1	1.1
Unassigned *Spartobacteria* (7)	0.0	0.0	0.0	0.0	0.0	0.0	0.0	0.0	0.0	0.0	0.0	0.0	0.0	0.0
Verrucomicrobiae,														
Verrucomicrobiaceae (22)	0.1	0.0	0.0	0.0	0.1	0.0	0.0	0.0	0.0	0.0	0.0	0.0	0.1	0.1
Unassigned *Verrucomicrobia* (1)	0.0	0.0	0.0	0.0	0.0	0.0	0.0	0.0	0.0	0.0	0.0	0.0	0.0	0.0

Sampling Time:	22 h							30 h						
Treatment:	C	CAA	Glu	Asp	Thr	Ala/Gly	Val/Gly	C	CAA	Glu	Asp	Thr	Ala/Gly	Val/Gly
**Phyla, Class, Family**[b]	Relative Abundance (%)													
Acidobacteria,														
Acidobacteria,														
Acidobacteriaceae (16)	0.0	0.0	0.0	0.0	0.0	0.0	0.1	0.1	0.0	0.0	0.0	0.0	0.0	0.0
Blastocatellia,														
Blastocatellaceae (23)	0.1	0.1	0.1	0.1	0.1	0.1	0.1	0.1	0.1	0.1	0.1	0.1	0.1	0.1
Holophagae,														
Unassigned *Holophagae* (12)	0.1	0.0	0.0	0.0	0.1	0.1	0.1	0.1	0.1	0.1	0.1	0.1	0.1	0.1
Solibacteres,														
Solibacteraceae (34)	0.2	0.1	0.1	0.1	0.1	0.1	0.2	0.1	0.1	0.1	0.1	0.1	0.1	0.1

Sampling Time:	22 h							30 h						
Treatment:	C	CAA	Glu	Asp	Thr	Ala/Gly	Val/Gly	C	CAA	Glu	Asp	Thr	Ala/Gly	Val/Gly
**Phyla, Class, Family[b]**	**Relative Abundance (%)**													
Subgroup_11 (5)	0.0	0.0	0.0	0.0	0.0	0.0	0.0	0.0	0.0	0.0	0.0	0.0	0.0	0.0
Subgroup_17 (16)	0.1	0.0	0.0	0.0	0.1	0.1	0.1	0.1	0.0	0.0	0.0	0.1	0.1	0.1
Subgroup_22 (16)	0.0	0.0	0.0	0.0	0.0	0.0	0.0	0.0	0.0	0.0	0.0	0.0	0.0	0.0
Subgroup_25 (9)	0.0	0.0	0.0	0.0	0.0	0.0	0.0	0.0	0.0	0.0	0.0	0.0	0.0	0.0
Subgroup_5 (10)	0.1	0.1	0.0	0.0	0.1	0.1	0.1	0.1	0.1	0.0	0.1	0.1	0.1	0.1
Subgroup_6 (113)	1.7	0.8	0.8	0.9	1.0	1.8	1.6	1.5	0.9	0.9	0.9	1.0	1.5	1.2
Unassigned *Acidobacteria* (8)	0.0	0.0	0.0	0.0	0.0	0.0	0.0	0.0	0.0	0.0	0.0	0.0	0.0	0.0
Actinobacteria,														
Acidimicrobiia,														
Acidimicrobiaceae (30)	1.2	0.6	0.7	0.7	0.9	1.1	1.2	1.1	0.8	0.7	0.8	0.9	1.2	1.0
Iamiaceae (12)	0.2	0.1	0.1	0.1	0.1	0.2	0.2	0.1	0.1	0.1	0.1	0.1	0.2	0.1
Unassigned *Acidimicrobiales* (70)	1.6	1.0	0.9	1.0	1.3	1.8	1.6	1.5	1.0	1.0	1.0	1.3	1.5	1.4
Actinobacteria,														
Actinospicaceae (1)	0.0	0.0	0.0	0.0	0.0	0.0	0.0	0.0	0.0	0.0	0.0	0.0	0.0	0.0
Catenulisporaceae (1)	0.0	0.0	0.0	0.0	0.0	0.0	0.0	0.0	0.0	0.0	0.0	0.0	0.0	0.0
Mycobacteriaceae (12)	1.0	0.6	0.5	0.6	0.7	1.1	0.9	0.9	0.6	0.6	0.6	0.7	0.9	0.8
Nocardiaceae (12)	0.1	0.0	0.0	0.0	0.0	0.1	0.1	0.1	0.0	0.0	0.0	0.0	0.0	0.1
Acidothermaceae (10)	0.2	0.1	0.1	0.1	0.2	0.2	0.2	0.2	0.1	0.1	0.1	0.1	0.2	0.1
Cryptosporangiaceae (2)	0.0	0.0	0.0	0.0	0.0	0.0	0.0	0.0	0.0	0.0	0.0	0.0	0.0	0.0
Frankiaceae (5)	0.1	0.1	0.1	0.1	0.1	0.2	0.1	0.1	0.1	0.1	0.1	0.1	0.1	0.1
Geodermatophilaceae (3)	0.0	0.0	0.0	0.0	0.0	0.0	0.0	0.0	0.0	0.0	0.0	0.0	0.0	0.0
Nakamurellaceae (5)	0.2	0.1	0.1	0.1	0.1	0.2	0.2	0.2	0.1	0.1	0.1	0.1	0.2	0.1
Sporichthyaceae (6)	0.1	0.0	0.0	0.1	0.1	0.1	0.1	0.1	0.1	0.1	0.0	0.1	0.1	0.1
Glycomycetaceae (2)	0.0	0.0	0.0	0.0	0.0	0.0	0.0	0.0	0.0	0.0	0.0	0.0	0.0	0.0
Kineosporiaceae (5)	0.0	0.0	0.0	0.0	0.0	0.0	0.0	0.0	0.0	0.0	0.0	0.0	0.0	0.0
Beutenbergiaceae (1)	0.0	0.0	0.0	0.0	0.0	0.0	0.0	0.0	0.0	0.0	0.0	0.0	0.0	0.0
Brevibacteriaceae (1)	0.0	0.0	0.0	0.0	0.0	0.0	0.0	0.0	0.0	0.0	0.0	0.0	0.0	0.0
Cellulomonadaceae (1)	0.1	0.0	0.0	0.0	0.1	0.1	0.1	0.1	0.1	0.1	0.0	0.1	0.1	0.1
Demequinaceae (1)	0.0	0.0	0.0	0.0	0.0	0.0	0.0	0.0	0.0	0.0	0.0	0.0	0.0	0.0
Dermabacteraceae (1)	0.0	0.0	0.0	0.0	0.0	0.0	0.0	0.0	0.0	0.0	0.0	0.0	0.0	0.0
Dermacoccaceae (1)	0.0	0.0	0.0	0.0	0.0	0.0	0.0	0.0	0.0	0.0	0.0	0.0	0.0	0.0
Intrasporangiaceae (4)	0.4	0.2	0.2	0.2	0.2	0.4	0.3	0.3	0.2	0.2	0.2	0.2	0.3	0.3
Microbacteriaceae (15)	0.2	0.1	0.1	0.1	0.1	0.2	0.2	0.2	0.1	0.1	0.1	0.1	0.2	0.2
Micrococcaceae (4)	0.4	0.2	0.2	0.2	0.2	0.3	0.3	0.3	0.2	0.2	0.3	0.2	0.4	0.4
Promicromonosporaceae (1)	0.0	0.0	0.0	0.0	0.0	0.0	0.0	0.0	0.0	0.0	0.0	0.0	0.0	0.0
Sanguibacteraceae (1)	0.0	0.0	0.0	0.0	0.0	0.0	0.0	0.0	0.0	0.0	0.0	0.0	0.0	0.0
Micromonosporaceae (44)	0.9	0.5	0.5	0.6	0.7	1.0	0.9	0.9	0.6	0.6	0.5	0.7	0.8	0.7
Nocardioidaceae (41)	1.9	1.1	1.1	1.2	1.5	2.0	2.0	2.1	1.4	1.2	1.2	1.6	2.1	1.8
Propionibacteriaceae (11)	0.2	0.1	0.1	0.1	0.2	0.2	0.2	0.2	0.1	0.2	0.1	0.1	0.2	0.2
Pseudonocardiaceae (20)	0.5	0.3	0.3	0.3	0.4	0.7	0.6	0.5	0.3	0.3	0.3	0.4	0.4	0.4
Streptomycetaceae (5)	0.6	0.3	0.3	0.3	0.4	0.7	0.6	0.6	0.4	0.4	0.4	0.5	0.6	0.6
Nocardiopsaceae (1)	0.0	0.0	0.0	0.0	0.0	0.0	0.0	0.0	0.0	0.0	0.0	0.0	0.0	0.0
Streptosporangiaceae (4)	0.1	0.0	0.0	0.0	0.0	0.1	0.1	0.1	0.0	0.0	0.0	0.1	0.1	0.1
Thermomonosporaceae (15)	0.1	0.0	0.0	0.0	0.0	0.1	0.0	0.0	0.0	0.0	0.0	0.0	0.0	0.0
Unassigned *Frankiales* (2)	0.0	0.0	0.0	0.0	0.0	0.0	0.0	0.0	0.0	0.0	0.0	0.0	0.0	0.0
Unassigned *Actinobacteria* (2)	0.1	0.0	0.0	0.0	0.1	0.1	0.1	0.1	0.0	0.0	0.0	0.1	0.0	0.0
Coriobacteriia,														
Coriobacteriaceae (1)	0.0	0.0	0.0	0.0	0.0	0.0	0.0	0.0	0.0	0.0	0.0	0.0	0.0	0.0
Rubrobacteria,														
Rubrobacteriaceae (6)	0.1	0.1	0.0	0.1	0.1	0.2	0.1	0.1	0.1	0.1	0.1	0.1	0.1	0.1
Thermoleophilia,														

Sampling Time:	22 h							30 h						
Treatment:	C	CAA	Glu	Asp	Thr	Ala/Gly	Val/Gly	C	CAA	Glu	Asp	Thr	Ala/Gly	Val/Gly
**Phyla, Class, Family[b]**	Relative Abundance (%)													
Gaiellaceae (11)	2.1	1.1	1.2	1.2	1.5	2.3	2.0	1.9	1.3	1.3	1.3	1.7	2.0	1.7
Unassigned *Gaiellales* (72)	3.9	2.2	2.2	2.5	3.2	4.5	4.0	3.9	2.6	2.5	2.6	3.2	4.0	3.4
Conexibacteraceae (3)	0.0	0.0	0.0	0.0	0.0	0.0	0.0	0.0	0.0	0.0	0.0	0.0	0.0	0.0
Parviterribacteraceae (3)	0.0	0.0	0.0	0.0	0.0	0.0	0.0	0.0	0.0	0.0	0.0	0.0	0.0	0.0
Patulibacteraceae (15)	0.0	0.0	0.0	0.0	0.0	0.0	0.0	0.0	0.0	0.0	0.0	0.0	0.0	0.0
Solirubrobacteraceae (15)	0.7	0.4	0.4	0.5	0.6	0.9	0.7	0.8	0.5	0.5	0.4	0.5	0.7	0.5
Unassigned *Solirubrobacterales* (26)	0.7	0.4	0.4	0.4	0.6	0.9	0.7	0.7	0.5	0.4	0.3	0.5	0.6	0.5
Unassigned *Thermoleophilia* (67)	2.3	1.2	1.3	1.4	1.6	2.5	2.2	2.2	1.5	1.5	1.3	1.6	2.0	1.7
Unassigned *Actinobacteria* (34)	1.5	0.9	0.9	1.0	1.2	1.8	1.5	1.4	1.0	0.9	1.0	1.2	1.5	1.3
Armatimonadetes,														
Armatimonadia,														
Unassigned *Armatimonadales* (3)	0.0	0.0	0.0	0.0	0.0	0.0	0.0	0.0	0.0	0.0	0.0	0.0	0.0	0.0
Chthonomonadaceae (2)	0.0	0.0	0.0	0.0	0.0	0.0	0.0	0.0	0.0	0.0	0.0	0.0	0.0	0.0
Unassigned *Chthonomonadales* (2)	0.0	0.0	0.0	0.0	0.0	0.0	0.0	0.0	0.0	0.0	0.0	0.0	0.0	0.0
Unassigned *Armatimonadetes* (21)	0.0	0.0	0.0	0.0	0.0	0.0	0.0	0.0	0.0	0.0	0.0	0.0	0.0	0.0
Bacteroidetes,														
Bacteroidia,														
Prolixibacteraceae (1)	0.0	0.0	0.0	0.0	0.0	0.0	0.0	0.0	0.0	0.0	0.0	0.0	0.0	0.0
Cytophagia (2)	0.0	0.0	0.0	0.0	0.0	0.0	0.0	0.0	0.0	0.0	0.0	0.0	0.0	0.0
Cytophagaceae (33)	0.0	0.0	0.0	0.0	0.0	0.0	0.0	0.0	0.0	0.0	0.0	0.0	0.0	0.0
Flammeovirgaceae (1)	0.0	0.0	0.0	0.0	0.0	0.0	0.0	0.0	0.0	0.0	0.0	0.0	0.0	0.0
Flavobacteriia,														
Flavobacteriaceae (21)	0.7	0.3	0.2	0.6	0.6	0.1	0.2	0.8	0.3	0.2	0.4	0.5	0.1	0.1
Sphingobacteriia,														
Chitinophagaceae (48)	0.1	0.1	0.0	0.1	0.1	0.1	0.1	0.1	0.1	0.0	0.1	0.1	0.1	0.1
Lentimicrobiaceae (1)	0.0	0.0	0.0	0.0	0.0	0.0	0.0	0.0	0.0	0.0	0.0	0.0	0.0	0.0
Saprospiraceae (2)	0.0	0.0	0.0	0.0	0.0	0.0	0.0	0.0	0.0	0.0	0.0	0.0	0.0	0.0
Sphingobacteriaceae (3)	0.0	0.0	0.0	0.0	0.0	0.0	0.0	0.0	0.0	0.0	0.0	0.0	0.0	0.0
Unassigned *Sphingobacteriales* (14)	0.0	0.0	0.0	0.0	0.0	0.0	0.0	0.0	0.0	0.0	0.0	0.0	0.0	0.0
Unassigned *Sphingobacteriia* (10)	0.0	0.0	0.0	0.0	0.0	0.0	0.0	0.0	0.0	0.0	0.0	0.0	0.0	0.0
Unassigned *Bacteroidetes* (3)	0.0	0.0	0.0	0.0	0.0	0.0	0.0	0.0	0.0	0.0	0.0	0.0	0.0	0.0
BJ-169,														
Unassigned *BJ-169* (6)	0.0	0.0	0.0	0.0	0.0	0.0	0.0	0.0	0.0	0.0	0.0	0.0	0.0	0.0
BRC1,														
Unassigned *BCR1* (14)	0.0	0.0	0.0	0.0	0.0	0.0	0.0	0.0	0.0	0.0	0.0	0.0	0.0	0.0
Chlamydiae,														
Chlamydiae,														
Chlamydiaceae (4)	0.0	0.0	0.0	0.0	0.0	0.0	0.0	0.0	0.0	0.0	0.0	0.0	0.0	0.0
Parachlamydiaceae (154)	0.1	0.0	0.0	0.0	0.1	0.1	0.1	0.1	0.1	0.1	0.1	0.1	0.1	0.1
Simkaniaceae (11)	0.0	0.0	0.0	0.0	0.0	0.0	0.0	0.0	0.0	0.0	0.0	0.0	0.0	0.0
Waddliaceae (1)	0.0	0.0	0.0	0.0	0.0	0.0	0.0	0.0	0.0	0.0	0.0	0.0	0.0	0.0
Unassigned *Chlamydiales* (7)	0.0	0.0	0.0	0.0	0.0	0.0	0.0	0.0	0.0	0.0	0.0	0.0	0.0	0.0
Chlorobi,														
Chlorobia,														
Unassigned *Chlorobia* (4)	0.0	0.0	0.0	0.0	0.0	0.0	0.0	0.0	0.0	0.0	0.0	0.0	0.0	0.0
Chloroflexi,														
Anaerolineae,														
Anaerolineaceae (14)	0.0	0.0	0.0	0.0	0.0	0.0	0.0	0.0	0.0	0.0	0.0	0.0	0.0	0.0
Unassigned *Ardenticatenia* (3)	0.0	0.0	0.0	0.0	0.0	0.0	0.0	0.0	0.0	0.0	0.0	0.0	0.0	0.0

Sampling Time:	22 h							30 h						
Treatment:	C	CAA	Glu	Asp	Thr	Ala/Gly	Val/Gly	C	CAA	Glu	Asp	Thr	Ala/Gly	Val/Gly
**Phyla, Class, Family[b]**	**Relative Abundance (%)**													
Caldilineae,														
Caldilineaceae (15)	0.1	0.0	0.1	0.1	0.1	0.1	0.1	0.1	0.1	0.1	0.1	0.1	0.1	0.1
Chloroflexia,														
Roseiflexaceae (8)	0.2	0.1	0.1	0.1	0.1	0.2	0.2	0.2	0.1	0.1	0.1	0.1	0.2	0.1
Unassigned *Chloroflexia* (4)	0.0	0.0	0.0	0.0	0.0	0.0	0.0	0.0	0.0	0.0	0.0	0.0	0.0	0.0
Ktedonobacteria,														
Ktedonobacterales,														
Ktedonobacteraceae (9)	0.0	0.0	0.0	0.0	0.0	0.0	0.0	0.0	0.0	0.0	0.0	0.0	0.0	0.0
Thermosporotrichaceae (7)	0.0	0.0	0.0	0.0	0.0	0.0	0.0	0.0	0.0	0.0	0.0	0.0	0.0	0.0
Unassigned *Ktedonobacterales* (6)	0.0	0.0	0.0	0.0	0.0	0.0	0.0	0.0	0.0	0.0	0.0	0.0	0.0	0.0
Unassigned *Ktedonobacteria* (18)	0.1	0.1	0.1	0.1	0.1	0.1	0.1	0.1	0.1	0.1	0.1	0.1	0.1	0.1
Thermomicrobia,														
Thermomicrobiaceae (1)	0.0	0.0	0.0	0.0	0.0	0.0	0.0	0.0	0.0	0.0	0.0	0.0	0.0	0.0
Unassigned *Thermomicrobia* (56)	0.3	0.2	0.2	0.2	0.2	0.3	0.4	0.3	0.2	0.2	0.2	0.3	0.3	0.3
Unassigned *Chloroflexi* (111)	1.9	1.0	0.9	1.1	1.4	2.0	1.9	1.7	1.1	1.0	1.1	1.3	1.8	1.5
Cyanobacteria,														
Chloroplast,														
Unassigned *Chloroplast* (34)	0.0	0.0	0.0	0.0	0.0	0.0	0.0	0.0	0.0	0.0	0.0	0.0	0.0	0.0
Cyanobacteria,														
Unassigned *Cyanobacteria* (2)	0.0	0.0	0.0	0.0	0.0	0.0	0.0	0.0	0.0	0.0	0.0	0.0	0.0	0.0
Gastranaerophilales,														
Unassigned *Gastranaerophilales* (1)	0.0	0.0	0.0	0.0	0.0	0.0	0.0	0.0	0.0	0.0	0.0	0.0	0.0	0.0
Obscuribacterales,														
Unassigned *Obscuribacterales* (6)	0.0	0.0	0.0	0.0	0.0	0.0	0.0	0.0	0.0	0.0	0.0	0.0	0.0	0.0
Unassigned *Cyanobacteria* (11)	0.0	0.0	0.0	0.0	0.0	0.0	0.0	0.0	0.0	0.0	0.0	0.0	0.0	0.0
Deferribacteres,														
Unassigned *Deferribacteres* (1)	0.0	0.0	0.0	0.0	0.0	0.0	0.0	0.0	0.0	0.0	0.0	0.0	0.0	0.0
Deinococcus-Thermus,														
Deinococci,														
Unassigned *Deinococci* (4)	0.0	0.0	0.0	0.0	0.0	0.0	0.0	0.0	0.0	0.0	0.0	0.0	0.0	0.0
Elusimicrobia,														
Unassigned *Elusimicrobia,*	0.0	0.0	0.0	0.0	0.0	0.0	0.0	0.0	0.0	0.0	0.0	0.0	0.0	0.0
Euryarchaeota,														
Methanomicrobia,														
Methanosarcinaceae (1)	0.0	0.0	0.0	0.0	0.0	0.0	0.0	0.0	0.0	0.0	0.0	0.0	0.0	0.0
Thermoplasmata,														
Unassinged Thermoplasmatales (1)	0.0	0.0	0.0	0.0	0.0	0.0	0.0	0.0	0.0	0.0	0.0	0.0	0.0	0.0
Fibrobacteres,														
Fibrobacteria,														
Fibrobacteraceae (5)	0.0	0.0	0.0	0.0	0.0	0.0	0.0	0.0	0.0	0.0	0.0	0.0	0.0	0.0
Unassigned *Fibrobacterales* (3)	0.0	0.0	0.0	0.0	0.0	0.0	0.0	0.0	0.0	0.0	0.0	0.0	0.0	0.0
Firmicutes,														
Bacilli,														
Alicyclobacillaceae (5)	0.0	0.0	0.0	0.0	0.0	0.0	0.0	0.0	0.0	0.0	0.0	0.0	0.0	0.0
Bacillaceae (38)	0.6	0.5	0.4	0.4	0.7	0.8	0.7	0.9	0.9	0.7	0.5	1.0	1.0	1.1
Paenibacillaceae (88)	0.2	0.1	0.1	0.1	0.1	0.2	0.2	0.2	0.2	0.2	0.3	0.2	0.3	0.3
Pasteuriaceae (6)	0.0	0.0	0.0	0.0	0.0	0.0	0.0	0.0	0.0	0.0	0.0	0.0	0.0	0.0
Planococcaceae (7)	0.1	0.0	0.0	0.0	0.0	0.1	0.1	0.1	0.0	0.1	0.0	0.1	0.1	0.1
Sporolactobacillaceae (1)	0.0	0.0	0.0	0.0	0.0	0.0	0.0	0.0	0.0	0.0	0.0	0.0	0.0	0.0

Sampling Time:	22 h							30 h						
Treatment:	C	CAA	Glu	Asp	Thr	Ala/Gly	Val/Gly	C	CAA	Glu	Asp	Thr	Ala/Gly	Val/Gly
**Phyla, Class, Family[b]**	**Relative Abundance (%)**													
Staphylococcaceae (1)	0.0	0.0	0.0	0.0	0.0	0.0	0.0	0.0	0.0	0.0	0.0	0.0	0.0	0.0
Thermoactinomycetaceae (18)	0.0	0.0	0.0	0.0	0.0	0.0	0.0	0.0	0.0	0.0	0.0	0.0	0.0	0.0
Streptococcaceae (1)	0.0	0.0	0.0	0.0	0.0	0.0	0.0	0.0	0.0	0.0	0.0	0.0	0.0	0.0
Unassigned *Bacilli* (13)	0.2	0.2	0.1	0.2	0.2	0.2	0.3	0.2	0.2	0.1	0.2	0.2	0.2	0.2
Clostridia,														
Caldicoprobacteraceae (7)	0.0	0.0	0.0	0.0	0.0	0.0	0.0	0.0	0.0	0.0	0.0	0.0	0.0	0.0
Christensenellaceae (7)	0.0	0.0	0.0	0.0	0.0	0.0	0.0	0.0	0.0	0.0	0.0	0.0	0.0	0.0
Clostridiaceae (38) **[A14]**	0.7	0.6	0.9	0.6	0.7	1.1	0.8	2.0	1.5	2.6	2.0	2.2	2.3	1.7
Defluviitaleaceae (4)	0.0	0.0	0.0	0.0	0.0	0.0	0.0	0.0	0.0	0.0	0.0	0.0	0.0	0.0
Eubacteriaceae (4)	0.0	0.0	0.0	0.0	0.0	0.0	0.0	0.0	0.0	0.0	0.0	0.0	0.0	0.0
Gracilibacteraceae (7)	0.0	0.0	0.0	0.0	0.0	0.0	0.0	0.0	0.0	0.0	0.0	0.0	0.0	0.0
Heliobacteriaceae (22)	0.0	0.0	0.0	0.0	0.0	0.0	0.0	0.0	0.0	0.0	0.0	0.0	0.0	0.0
Lachnospiraceae (79)	0.2	0.2	0.1	0.3	0.2	0.6	0.4	1.2	1.2	0.6	1.5	1.1	3.2	3.0
Peptococcaceae (17)	1.8	1.1	1.0	1.0	1.0	1.3	1.5	1.8	1.4	1.4	1.4	1.3	2.0	1.8
Peptostreptococcaceae (11) **[GPT-4], [A8]**	0.6	1.0	0.3	0.2	1.2	1.5	1.1	0.8	2.0	0.5	0.2	2.6	5.5	2.7
Ruminococcaceae (96)	0.1	0.0	0.0	0.0	0.0	0.1	0.1	0.1	0.1	0.0	0.1	0.1	0.1	0.1
Syntrophomonadaceae (10)	0.0	0.0	0.0	0.0	0.0	0.0	0.0	0.0	0.0	0.0	0.0	0.0	0.0	0.0
Thermoanaerobacteraceae (8)	0.0	0.0	0.0	0.0	0.0	0.0	0.0	0.0	0.0	0.0	0.0	0.0	0.0	0.0
Unassigned *Clostridiales* (17)	0.0	0.0	0.0	0.0	0.0	0.0	0.0	0.1	0.1	0.0	0.1	0.0	0.1	0.1
Unassigned *Thermoanaerobacterales* (1)	0.0	0.0	0.0	0.0	0.0	0.0	0.0	0.0	0.0	0.0	0.0	0.0	0.0	0.0
Unassigned *Clostridia* (34)	0.0	0.0	0.0	0.0	0.0	0.0	0.0	0.0	0.0	0.0	0.0	0.0	0.0	0.0
Erysipelotrichia,														
Erysipelotrichaceae (18)	0.0	0.0	0.0	0.0	0.0	0.0	0.0	0.0	0.0	0.0	0.0	0.0	0.0	0.0
Limnochordia,														
Limnochordaceae (23)	0.0	0.0	0.0	0.0	0.0	0.0	0.0	0.0	0.0	0.0	0.0	0.0	0.0	0.0
Unassigned *Limnochordales* (3)	0.0	0.0	0.0	0.0	0.0	0.0	0.0	0.0	0.0	0.0	0.0	0.0	0.0	0.0
Negativicutes,														
Veillonellaceae (20)	0.0	0.0	0.0	0.0	0.0	0.0	0.0	0.0	0.0	0.0	0.0	0.0	0.0	0.0
Unassigned *Selenomonadales* (3)	0.0	0.0	0.0	0.0	0.0	0.0	0.0	0.0	0.0	0.0	0.0	0.0	0.0	0.0
Unassigned *Firmicutes* (1)	0.0	0.0	0.0	0.0	0.0	0.0	0.0	0.0	0.0	0.0	0.0	0.0	0.0	0.0
Fusobacteria,														
Fusobacteriia,														
Fusobacteriaceae (10) **[GPT-5]**	21	39	44	30	25	17	22	18	33	39	23	20	14	23
Leptotrichiaceae (1)	0.0	0.0	0.0	0.0	0.0	0.0	0.0	0.0	0.0	0.0	0.0	0.0	0.0	0.0
Gemmatimonadetes,														
Gemmatimonadetes,														
Gemmatimonadaceae (37)	0.5	0.2	0.2	0.3	0.3	0.5	0.4	0.4	0.2	0.3	0.3	0.4	0.5	0.3
Longimicrobiaceae (1)	0.0	0.0	0.0	0.0	0.0	0.0	0.0	0.0	0.0	0.0	0.0	0.0	0.0	0.0
Unassigned *Gemmatimonadetes* (6)	0.0	0.0	0.0	0.0	0.0	0.0	0.0	0.0	0.0	0.0	0.0	0.0	0.0	0.0
Hydrogenedentes,														
Unassigned *Hydrogenedentes* (2)	0.0	0.0	0.0	0.0	0.0	0.0	0.0	0.0	0.0	0.0	0.0	0.0	0.0	0.0
Latescibacteria,														
Unassigned *Latescibacteria* (30)	0.0	0.0	0.0	0.0	0.0	0.0	0.0	0.0	0.0	0.0	0.0	0.0	0.0	0.0
Nitrospirae,														
Nitrospira,														
Nitrospiraceae (8)	0.1	0.1	0.1	0.1	0.1	0.2	0.2	0.1	0.1	0.1	0.1	0.1	0.1	0.1
Unassigned *Nitrospira* (18)	0.5	0.3	0.3	0.4	0.4	0.5	0.6	0.4	0.3	0.3	0.3	0.4	0.4	0.4
Parcubacteria,														
Unassigned *Parcubacteria* (1)	0.0	0.0	0.0	0.0	0.0	0.0	0.0	0.0	0.0	0.0	0.0	0.0	0.0	0.0

Sampling Time:	22 h							30 h						
Treatment:	C	CAA	Glu	Asp	Thr	Ala/Gly	Val/Gly	C	CAA	Glu	Asp	Thr	Ala/Gly	Val/Gly
**Phyla, Class, Family[b]**	Relative Abundance (%)													
Planctomycetes,														
Phycisphaerae,														
Phycisphaeraceae (38)	0.0	0.0	0.0	0.0	0.0	0.0	0.0	0.0	0.0	0.0	0.0	0.0	0.0	0.0
Tepidisphaeraceae (82)	0.5	0.3	0.3	0.3	0.4	0.4	0.5	0.4	0.3	0.3	0.3	0.4	0.5	0.4
Unassigned *Phycisphaerales* (4)	0.0	0.0	0.0	0.0	0.0	0.0	0.0	0.0	0.0	0.0	0.0	0.0	0.0	0.0
Unassigned *Phycisphaerae* (7)	0.0	0.0	0.0	0.0	0.0	0.0	0.0	0.0	0.0	0.0	0.0	0.0	0.0	0.0
Planctomycetacia,														
Planctomycetaceae (733)	3.1	1.7	1.9	2.1	2.5	2.7	3.3	3.1	2.0	1.8	2.1	2.7	3.4	2.7
Unassigned *Planctomycetes* (82)	0.1	0.0	0.0	0.0	0.1	0.1	0.1	0.0	0.0	0.0	0.0	0.0	0.0	0.0
Proteobacteria,														
Alphaproteobacteria,														
Caulobacteraceae (5)	0.0	0.0	0.0	0.0	0.0	0.0	0.0	0.0	0.0	0.0	0.0	0.0	0.0	0.0
Beijerinckiaceae (2)	0.0	0.0	0.0	0.0	0.0	0.0	0.0	0.0	0.0	0.0	0.0	0.0	0.0	0.0
Bradyrhizobiaceae (5)	0.8	0.5	0.5	0.6	0.7	0.9	0.9	0.8	0.5	0.5	0.6	0.7	0.8	0.8
Hyphomicrobiaceae (8)	0.3	0.1	0.1	0.2	0.2	0.2	0.3	0.3	0.2	0.1	0.2	0.2	0.3	0.2
Methylobacteriaceae (4)	0.1	0.1	0.1	0.1	0.1	0.2	0.1	0.1	0.1	0.1	0.1	0.1	0.1	0.1
Methylocystaceae (1)	0.0	0.0	0.0	0.0	0.0	0.0	0.0	0.0	0.0	0.0	0.0	0.0	0.0	0.0
Phyllobacteriaceae (3)	0.2	0.1	0.1	0.1	0.1	0.2	0.2	0.2	0.1	0.1	0.1	0.1	0.2	0.2
Rhizobiaceae (3)	0.0	0.0	0.0	0.0	0.0	0.0	0.0	0.0	0.0	0.0	0.0	0.0	0.0	0.0
Rhodobiaceae (2)	1.1	0.5	0.5	0.5	0.6	1.0	1.0	0.9	0.6	0.6	0.7	0.8	1.1	0.9
Roseiarcaceae (1)	0.0	0.0	0.0	0.0	0.0	0.0	0.0	0.0	0.0	0.0	0.0	0.0	0.0	0.0
Xanthobacteraceae (9)	2.7	1.4	1.4	1.4	1.9	2.3	2.6	2.4	1.6	1.4	1.7	2.0	2.7	2.2
Unassigned *Rhizobiales* (19)	0.2	0.1	0.1	0.1	0.1	0.2	0.2	0.2	0.1	0.1	0.1	0.2	0.2	0.2
Rhodobacteraceae (6)	0.0	0.0	0.0	0.0	0.0	0.1	0.0	0.0	0.0	0.0	0.0	0.0	0.0	0.0
Acetobacteraceae (15)	0.1	0.1	0.0	0.1	0.1	0.1	0.1	0.1	0.1	0.1	0.1	0.1	0.1	0.1
Rhodospirillaceae (19)	0.2	0.1	0.1	0.2	0.2	0.3	0.2	0.2	0.1	0.1	0.1	0.2	0.2	0.2
Unassigned *Rhodospirillales* (25)	0.5	0.2	0.2	0.3	0.4	0.5	0.5	0.5	0.3	0.2	0.2	0.4	0.5	0.4
Anaplasmataceae (1)	0.0	0.0	0.0	0.0	0.0	0.0	0.0	0.0	0.0	0.0	0.0	0.0	0.0	0.0
Holosporaceae (2)	0.0	0.0	0.0	0.0	0.0	0.0	0.0	0.0	0.0	0.0	0.0	0.0	0.0	0.0
Mitochondria (2)	0.0	0.0	0.0	0.0	0.0	0.0	0.0	0.0	0.0	0.0	0.0	0.0	0.0	0.0
Rickettsiaceae (1)	0.0	0.0	0.0	0.0	0.0	0.0	0.0	0.0	0.0	0.0	0.0	0.0	0.0	0.0
Unassigned *Rickettsiales* (7)	0.0	0.0	0.0	0.0	0.0	0.0	0.0	0.0	0.0	0.0	0.0	0.0	0.0	0.0
Erythrobacteraceae (1)	0.0	0.0	0.0	0.0	0.0	0.0	0.0	0.0	0.0	0.0	0.0	0.0	0.0	0.0
Sphingomonadaceae (5)	0.0	0.0	0.0	0.0	0.0	0.0	0.0	0.0	0.0	0.0	0.0	0.0	0.0	0.0
Unassigned *Sphingomonadales* (2)	0.0	0.0	0.0	0.0	0.0	0.0	0.0	0.0	0.0	0.0	0.0	0.0	0.0	0.0
Unassigned *Alphaproteobacteria* (19)	0.1	0.1	0.1	0.1	0.1	0.1	0.1	0.1	0.1	0.0	0.1	0.1	0.1	0.1
Betaproteobacteria,														
Alcaligenaceae (2)	0.1	0.0	0.0	0.0	0.0	0.0	0.0	0.0	0.0	0.0	0.0	0.0	0.1	0.0
Burkholderiaceae (10)	0.0	0.0	0.0	0.0	0.0	0.0	0.0	0.0	0.0	0.0	0.0	0.0	0.0	0.0
Comamonadaceae (17)	0.1	0.1	0.0	0.1	0.1	0.1	0.1	0.1	0.1	0.1	0.1	0.1	0.1	0.1
Oxalobacteraceae (15)	0.0	0.0	0.0	0.0	0.0	0.0	0.0	0.0	0.0	0.0	0.0	0.0	0.0	0.0
Neisseriaceae (1)	0.0	0.0	0.0	0.0	0.0	0.0	0.0	0.0	0.0	0.0	0.0	0.0	0.0	0.0
Gallionellaceae (3)	0.0	0.0	0.0	0.0	0.0	0.0	0.0	0.0	0.0	0.0	0.0	0.0	0.0	0.0
Nitrosomonadaceae (30)	0.2	0.1	0.1	0.1	0.2	0.2	0.3	0.2	0.1	0.1	0.1	0.2	0.2	0.2
Rhodocyclaceae (9)	0.1	0.0	0.0	0.0	0.1	0.1	0.1	0.1	0.0	0.0	0.0	0.0	0.1	0.0
Unassigned *Betaproteobacteria* (41)	0.3	0.1	0.1	0.1	0.2	0.2	0.2	0.2	0.1	0.1	0.1	0.2	0.2	0.2
Deltaproteobacteria,														
Bacteriovoracaceae (12)	0.0	0.0	0.0	0.0	0.0	0.0	0.0	0.0	0.0	0.0	0.0	0.0	0.0	0.0
Bdellovibrionaceae (54)	0.0	0.0	0.0	0.0	0.0	0.0	0.0	0.0	0.0	0.0	0.0	0.0	0.0	0.0
Desulfarculaceae (1)	0.0	0.0	0.0	0.0	0.0	0.0	0.0	0.0	0.0	0.0	0.0	0.0	0.0	0.0
Desulfobulbaceae (3)	0.0	0.0	0.0	0.0	0.0	0.0	0.0	0.0	0.0	0.0	0.0	0.0	0.0	0.0

Sampling Time:	22 h							30 h						
Treatment:	C	CAA	Glu	Asp	Thr	Ala/Gly	Val/Gly	C	CAA	Glu	Asp	Thr	Ala/Gly	Val/Gly
**Phyla, Class, Family**[b]	**Relative Abundance (%)**													
Desulfurellaceae (41)	0.7	0.4	0.4	0.5	0.6	0.8	0.8	0.7	0.4	0.4	0.4	0.6	0.7	0.6
Desulfuromonadaceae (2)	0.0	0.0	0.0	0.0	0.0	0.0	0.0	0.0	0.0	0.0	0.0	0.0	0.0	0.0
Geobacteraceae (26)	0.1	0.1	0.1	0.1	0.1	0.2	0.1	0.1	0.1	0.1	0.1	0.1	0.1	0.1
Archangiaceae (20)	0.1	0.0	0.0	0.0	0.0	0.1	0.1	0.0	0.0	0.0	0.0	0.0	0.1	0.0
Haliangiaceae (74)	0.2	0.1	0.1	0.1	0.1	0.2	0.2	0.2	0.1	0.1	0.1	0.2	0.2	0.2
Myxococcaceae (3)	0.0	0.0	0.0	0.0	0.0	0.0	0.0	0.0	0.0	0.0	0.0	0.0	0.0	0.0
Nannocystaceae (6)	0.0	0.0	0.0	0.0	0.0	0.0	0.0	0.0	0.0	0.0	0.0	0.0	0.0	0.0
Phaselicystidaceae (14)	0.1	0.0	0.0	0.0	0.0	0.1	0.1	0.1	0.1	0.0	0.0	0.1	0.1	0.1
Polyangiaceae (48)	0.1	0.1	0.1	0.1	0.1	0.1	0.1	0.1	0.1	0.1	0.1	0.1	0.1	0.1
Sandaracinaceae (38)	0.1	0.0	0.0	0.0	0.0	0.1	0.1	0.1	0.0	0.0	0.0	0.1	0.1	0.1
Vulgatibacteraceae (3)	0.0	0.0	0.0	0.0	0.0	0.0	0.0	0.0	0.0	0.0	0.0	0.0	0.0	0.0
Unassigned *Myxococcales* (177)	0.2	0.1	0.1	0.1	0.1	0.2	0.1	0.2	0.1	0.1	0.1	0.1	0.2	0.1
Oligoflexaceae (29)	0.0	0.0	0.0	0.0	0.0	0.0	0.0	0.0	0.0	0.0	0.0	0.0	0.0	0.0
Unassigned *Oligoflexales* (86)	0.0	0.0	0.0	0.0	0.0	0.0	0.0	0.0	0.0	0.0	0.0	0.0	0.0	0.0
Syntrophaceae (1)	0.0	0.0	0.0	0.0	0.0	0.0	0.0	0.0	0.0	0.0	0.0	0.0	0.0	0.0
Unassigned *Deltaproteobacteria* (19)	0.0	0.0	0.0	0.0	0.0	0.0	0.0	0.0	0.0	0.0	0.0	0.0	0.0	0.0
Gammaproteobacteria,														
Acidiferrobacteraceae (3)	0.0	0.0	0.0	0.0	0.0	0.0	0.0	0.0	0.0	0.0	0.0	0.0	0.0	0.0
Aeromonadaceae (8) **[GPT-1]**	9.8	13	5.9	16	9.5	9.6	8.0	10	11	5.0	16	8.7	7.5	7.2
Shewanellaceae (3)	7.1	6.9	7.1	7.6	12	7.8	8.0	9.6	7.7	7.4	7.6	10	6.5	8.6
Cellvibrionaceae (2)	0.0	0.0	0.0	0.0	0.0	0.0	0.0	0.0	0.0	0.0	0.0	0.0	0.0	0.0
Halieaceae (4)	0.0	0.0	0.0	0.0	0.0	0.0	0.0	0.0	0.0	0.0	0.0	0.0	0.0	0.0
Enterobacteriaceae (12) **[GPT-2], [GPT-3]**	2.5	4.8	10	8.9	8.9	3.8	3.3	2.4	4.4	10	12	9.2	3.1	3.5
Coxiellaceae (35)	0.0	0.0	0.0	0.0	0.0	0.0	0.0	0.1	0.0	0.0	0.0	0.0	0.1	0.1
Legionellaceae (30)	0.0	0.0	0.0	0.0	0.0	0.0	0.0	0.0	0.0	0.0	0.0	0.0	0.0	0.0
Methylococcaceae (3)	0.0	0.0	0.0	0.0	0.0	0.0	0.0	0.0	0.0	0.0	0.0	0.0	0.0	0.0
Oleiphilaceae (1)	0.0	0.0	0.0	0.0	0.0	0.0	0.0	0.0	0.0	0.0	0.0	0.0	0.0	0.0
Moraxellaceae (2)	0.0	0.0	0.0	0.0	0.0	0.0	0.0	0.0	0.0	0.0	0.0	0.0	0.0	0.0
Pseudomonadaceae (4)	0.1	0.0	0.0	0.0	0.0	0.1	0.1	0.2	0.0	0.0	0.1	0.1	0.1	0.2
Unassigned Thiotrichales (1)	0.0	0.0	0.0	0.0	0.0	0.0	0.0	0.0	0.0	0.0	0.0	0.0	0.0	0.0
Xanthomonadaceae (12)	0.0	0.0	0.0	0.0	0.0	0.0	0.0	0.0	0.0	0.0	0.0	0.0	0.0	0.0
Unassigned *Xanthomonadales* (24)	0.2	0.1	0.1	0.1	0.1	0.2	0.2	0.2	0.1	0.1	0.1	0.2	0.2	0.2
Unassigned *Gammaproteobacteria* (30)	0.0	0.0	0.0	0.0	0.0	0.0	0.0	0.0	0.0	0.0	0.0	0.0	0.0	0.0
Unassigned *Proteobacteria* (2)	0.0	0.0	0.0	0.0	0.0	0.0	0.0	0.0	0.0	0.0	0.0	0.0	0.0	0.0
RsaHf231,														
Unassigned *RsaHf231* (1)	0.0	0.0	0.0	0.0	0.0	0.0	0.0	0.0	0.0	0.0	0.0	0.0	0.0	0.0
Saccharibacteria,														
Unassigned *Saccharibacteria* (78)	0.0	0.0	0.0	0.0	0.0	0.0	0.0	0.0	0.0	0.0	0.0	0.0	0.0	0.0
Spirochaetae,														
Spirochaetes,														
Spirochaetaceae (1)	0.0	0.0	0.0	0.0	0.0	0.0	0.0	0.0	0.0	0.0	0.0	0.0	0.0	0.0
Brevinemataceae (1)	0.0	0.0	0.0	0.0	0.0	0.0	0.0	0.0	0.0	0.0	0.0	0.0	0.0	0.0
Synergistetes,														
Synergistia,														
Synergistaceae (1)	0.0	0.0	0.0	0.0	0.0	0.0	0.0	0.0	0.0	0.0	0.0	0.0	0.0	0.0
Tectomicrobia,														
Unassigned *Tectomicrobia* (21)	0.1	0.1	0.1	0.1	0.1	0.2	0.2	0.1	0.1	0.1	0.1	0.1	0.1	0.1
Tenericutes,														
Mollicutes,														

Sampling Time:	22 h							30 h						
Treatment:	C	CAA	Glu	Asp	Thr	Ala/Gly	Val/Gly	C	CAA	Glu	Asp	Thr	Ala/Gly	Val/Gly
**Phyla, Class, Family[b]**	Relative Abundance (%)													
Unassigned *Entomoplasmatales* (3)	0.0	0.0	0.0	0.0	0.0	0.0	0.0	0.0	0.0	0.0	0.0	0.0	0.0	0.0
Haloplasmataceae (7)	0.0	0.0	0.0	0.0	0.0	0.0	0.0	0.0	0.0	0.0	0.0	0.0	0.0	0.0
Mycoplasmataceae (19)	9.7	7.3	5.4	5.9	6.5	8.4	8.0	8.6	7.6	5.9	7.2	7.2	10	9.5
Thaumarchaeota,														
Unassigned *Thaumarchaeota* (9)	0.0	0.0	0.0	0.0	0.0	0.0	0.0	0.0	0.0	0.0	0.0	0.0	0.0	0.0
TM6_Dependentiae,														
Unassigned *TM6_Dependentiae* (40)	0.0	0.0	0.0	0.0	0.0	0.0	0.0	0.0	0.0	0.0	0.0	0.0	0.0	0.0
Verrucomicrobia,														
Unassigned OPB35 soil group (87)	0.2	0.1	0.1	0.1	0.1	0.2	0.2	0.1	0.1	0.1	0.1	0.1	0.1	0.1
Opitutae,														
Opitutaceae (5)	0.0	0.0	0.0	0.0	0.0	0.0	0.0	0.0	0.0	0.0	0.0	0.0	0.0	0.0
Unassigned *Opitutae* (2)	0.0	0.0	0.0	0.0	0.0	0.0	0.0	0.0	0.0	0.0	0.0	0.0	0.0	0.0
Spartobacteria,														
Chthoniobacteraceae (46)	0.1	0.0	0.0	0.0	0.1	0.1	0.1	0.1	0.0	0.0	0.0	0.1	0.1	0.0
Unassigned *Chthoniobacterales* (2)	0.0	0.0	0.0	0.0	0.0	0.0	0.0	0.0	0.0	0.0	0.0	0.0	0.0	0.0
DA101 soil group (28)	2.8	1.5	1.5	1.7	2.0	2.6	2.7	2.6	1.7	1.7	1.6	2.1	2.5	2.1
Xiphinematobacteraceae (9)	0.9	0.6	0.6	0.7	0.9	0.9	1.0	1.0	0.7	0.6	0.7	0.9	1.0	0.9
Unassigned *Spartobacteria* (7)	0.0	0.0	0.0	0.0	0.0	0.0	0.0	0.0	0.0	0.0	0.0	0.0	0.0	0.0
Verrucomicrobiae,														
Verrucomicrobiaceae (22)	0.0	0.0	0.0	0.0	0.0	0.1	0.0	0.0	0.0	0.0	0.0	0.0	0.0	0.0
Unassigned *Verrucomicrobia* (1)	0.0	0.0	0.0	0.0	0.0	0.0	0.0	0.0	0.0	0.0	0.0	0.0	0.0	0.0

(B) 16S rRNA

Sampling Time:	0 h									10 h							22 h						
Treatment:	C1	C2	C3	CAA	Glu	Asp	Thr	Ala/ Gly	Val/ Gly	C	CAA	Glu	Asp	Thr	Ala/ Gly	Val/ Gly	C	CAA	Glu	Asp	Thr	Ala/ Gly	Val/ Gly
**Phyla, Class, Family[b]**	Relative Abundance (%)																						
Acidobacteria,																							
Acidobacteria,																							
Acidobacteriaceae (16)	0.1	0.0	0.0	0.1	0.1	0.0	0.1	0.1	0.1	0.0	0.0	0.0	0.1	0.1	0.0	0.1	0.1	0.0	0.0	0.1	0.0	0.1	0.0
Blastocatellia,																							
Blastocatellaceae (23)	0.1	0.1	0.1	0.1	0.1	0.1	0.1	0.1	0.1	0.1	0.1	0.1	0.1	0.1	0.1	0.1	0.1	0.0	0.1	0.0	0.1	0.1	0.1
Holophagae,																							
Unassigned *Holophagae* (12)	0.0	0.0	0.0	0.0	0.0	0.0	0.1	0.1	0.0	0.0	0.0	0.0	0.0	0.0	0.0	0.0	0.0	0.0	0.0	0.0	0.0	0.0	0.1
Solibacteres,																							
Solibacteraceae (34)	0.3	0.3	0.3	0.3	0.3	0.3	0.3	0.3	0.3	0.3	0.2	0.2	0.3	0.3	0.3	0.3	0.2	0.2	0.2	0.2	0.2	0.3	0.3
Subgroup_11 (5)	0.0	0.0	0.0	0.0	0.0	0.0	0.0	0.0	0.0	0.0	0.0	0.0	0.0	0.0	0.0	0.0	0.0	0.0	0.0	0.0	0.0	0.0	0.0
Subgroup_17 (16)	0.0	0.0	0.0	0.0	0.0	0.0	0.0	0.0	0.0	0.0	0.0	0.0	0.0	0.0	0.0	0.0	0.0	0.0	0.0	0.0	0.0	0.0	0.0
Subgroup_22 (16)	0.0	0.0	0.0	0.0	0.0	0.0	0.0	0.0	0.0	0.0	0.0	0.0	0.0	0.0	0.0	0.0	0.0	0.0	0.0	0.0	0.0	0.0	0.0
Subgroup_25 (9)	0.0	0.0	0.0	0.0	0.0	0.0	0.0	0.0	0.0	0.0	0.0	0.0	0.0	0.0	0.0	0.0	0.0	0.0	0.0	0.0	0.0	0.0	0.0
Subgroup_5 (10)	0.1	0.1	0.1	0.1	0.1	0.1	0.1	0.1	0.1	0.1	0.1	0.1	0.1	0.1	0.1	0.1	0.1	0.1	0.1	0.0	0.1	0.1	0.1
Subgroup_6 (113)	1.0	0.8	0.9	0.8	0.8	0.9	0.7	0.7	0.7	0.6	0.6	0.6	0.7	0.8	1.0	0.8	0.8	0.6	0.5	0.5	0.6	0.6	0.8
Unassigned *Acidobacteria* (8)	0.0	0.0	0.0	0.0	0.0	0.0	0.0	0.0	0.0	0.0	0.0	0.0	0.0	0.0	0.0	0.0	0.0	0.0	0.0	0.0	0.0	0.0	0.0
Actinobacteria,																							
Acidimicrobiia,																							

Sampling Time:	0 h									10 h							22 h						
Treatment:	C1	C2	C3	CAA	Glu	Asp	Thr	Ala/ Gly	Val/ Gly	C	CAA	Glu	Asp	Thr	Ala/ Gly	Val/ Gly	C	CAA	Glu	Asp	Thr	Ala/ Gly	Val/ Gly
**Phyla, Class, Family**[b]										**Relative Abundance (%)**													
Acidimicrobiaceae (30)	1.3	1.2	1.1	1.2	1.2	1.3	1.0	1.2	1.2	1.2	0.8	1.0	1.1	1.2	1.3	1.4	1.1	0.6	0.7	0.9	1.0	1.2	1.3
Iamiaceae (12)	0.1	0.1	0.1	0.2	0.1	0.2	0.2	0.2	0.2	0.2	0.1	0.2	0.2	0.1	0.1	0.2	0.1	0.1	0.1	0.1	0.1	0.2	0.2
Unassigned *Acidimicrobiales* (70)	1.2	1.1	1.1	1.3	1.3	1.3	1.4	1.5	1.5	1.4	0.9	1.2	1.3	1.2	1.3	1.5	1.2	0.8	0.8	1.1	1.1	1.5	1.5
Actinobacteria,																							
Actinospicaceae (1)	0.0	0.0	0.0	0.0	0.0	0.0	0.0	0.0	0.0	0.0	0.0	0.0	0.0	0.0	0.0	0.0	0.0	0.0	0.0	0.0	0.0	0.0	0.0
Catenulisporaceae (1)	0.0	0.0	0.0	0.0	0.0	0.0	0.0	0.0	0.0	0.0	0.0	0.0	0.0	0.0	0.0	0.0	0.0	0.0	0.0	0.0	0.0	0.0	0.0
Mycobacteriaceae (12)	0.5	0.4	0.5	0.5	0.5	0.7	0.7	0.8	0.7	0.7	0.4	0.5	0.6	0.5	0.5	0.6	0.4	0.3	0.3	0.3	0.4	0.6	0.6
Nocardiaceae (12)	0.1	0.0	0.0	0.1	0.1	0.1	0.1	0.1	0.1	0.1	0.0	0.0	0.1	0.1	0.0	0.1	0.1	0.0	0.0	0.0	0.0	0.1	0.1
Acidothermaceae (10)	0.1	0.1	0.1	0.1	0.1	0.1	0.1	0.1	0.1	0.1	0.1	0.1	0.1	0.1	0.1	0.1	0.1	0.1	0.0	0.1	0.1	0.1	0.1
Cryptosporangiaceae (2)	0.0	0.0	0.0	0.0	0.0	0.0	0.0	0.0	0.0	0.0	0.0	0.0	0.0	0.0	0.0	0.0	0.0	0.0	0.0	0.0	0.0	0.0	0.0
Frankiaceae (5)	0.1	0.1	0.1	0.1	0.1	0.1	0.1	0.1	0.1	0.1	0.1	0.1	0.1	0.1	0.1	0.1	0.1	0.1	0.0	0.1	0.1	0.1	0.1
Geodermatophilaceae (3)	0.0	0.0	0.0	0.0	0.0	0.0	0.0	0.0	0.0	0.0	0.0	0.0	0.0	0.0	0.0	0.0	0.0	0.0	0.0	0.0	0.0	0.0	0.0
Nakamurellaceae (5)	0.3	0.3	0.3	0.3	0.3	0.3	0.2	0.2	0.2	0.2	0.1	0.2	0.2	0.2	0.3	0.2	0.2	0.1	0.1	0.2	0.2	0.2	0.2
Sporichthyaceae (6)	0.1	0.1	0.1	0.1	0.1	0.1	0.1	0.1	0.1	0.1	0.1	0.1	0.1	0.1	0.1	0.1	0.1	0.0	0.1	0.1	0.1	0.1	0.1
Glycomycetaceae (2)	0.0	0.0	0.0	0.0	0.0	0.0	0.0	0.0	0.0	0.0	0.0	0.0	0.0	0.0	0.0	0.0	0.0	0.0	0.0	0.0	0.0	0.0	0.0
Kineosporiaceae (5)	0.0	0.0	0.0	0.0	0.0	0.1	0.0	0.1	0.0	0.0	0.0	0.0	0.0	0.0	0.1	0.1	0.1	0.0	0.0	0.0	0.0	0.0	0.0
Beutenbergiaceae (1)	0.0	0.0	0.0	0.0	0.0	0.0	0.0	0.0	0.0	0.0	0.0	0.0	0.0	0.0	0.0	0.0	0.0	0.0	0.0	0.0	0.0	0.0	0.0
Brevibacteriaceae (1)	0.0	0.0	0.0	0.0	0.0	0.0	0.0	0.0	0.0	0.0	0.0	0.0	0.0	0.0	0.0	0.0	0.0	0.0	0.0	0.0	0.0	0.0	0.0
Cellulomonadaceae (1)	0.2	0.2	0.2	0.2	0.2	0.2	0.1	0.2	0.2	0.2	0.1	0.2	0.2	0.2	0.2	0.2	0.2	0.1	0.1	0.1	0.1	0.2	0.2
Demequinaceae (1)	0.0	0.0	0.0	0.0	0.0	0.0	0.0	0.0	0.0	0.0	0.0	0.0	0.0	0.0	0.0	0.0	0.0	0.0	0.0	0.0	0.0	0.0	0.0
Dermabacteraceae (1)	0.0	0.0	0.0	0.0	0.0	0.0	0.0	0.0	0.0	0.0	0.0	0.0	0.0	0.0	0.0	0.0	0.0	0.0	0.0	0.0	0.0	0.0	0.0
Dermacoccaceae (1)	0.0	0.0	0.0	0.0	0.0	0.0	0.0	0.0	0.0	0.0	0.0	0.0	0.0	0.0	0.0	0.0	0.0	0.0	0.0	0.0	0.0	0.0	0.0
Intrasporangiaceae (4)	0.2	0.2	0.2	0.2	0.2	0.3	0.2	0.2	0.2	0.2	0.2	0.2	0.2	0.2	0.3	0.2	0.3	0.2	0.1	0.2	0.2	0.2	0.3
Microbacteriaceae (15)	0.2	0.2	0.1	0.2	0.2	0.2	0.1	0.1	0.2	0.1	0.1	0.1	0.2	0.1	0.1	0.2	0.1	0.1	0.1	0.1	0.1	0.1	0.1
Micrococcaceae (4)	0.3	0.2	0.2	0.3	0.3	0.3	0.2	0.3	0.3	0.2	0.2	0.2	0.2	0.2	0.2	0.2	0.2	0.1	0.1	0.1	0.1	0.2	0.2
Promicromonosporaceae (1)	0.0	0.0	0.0	0.0	0.0	0.0	0.0	0.0	0.0	0.0	0.0	0.0	0.0	0.0	0.0	0.0	0.0	0.0	0.0	0.0	0.0	0.0	0.0
Sanguibacteraceae (1)	0.0	0.0	0.0	0.0	0.0	0.0	0.0	0.0	0.0	0.0	0.0	0.0	0.0	0.0	0.0	0.0	0.0	0.0	0.0	0.0	0.0	0.0	0.0
Micromonosporaceae (44)	1.1	0.9	0.9	1.0	1.0	1.3	0.9	1.1	1.1	1.3	0.8	0.9	1.0	1.0	1.1	1.3	1.0	0.7	0.6	0.7	0.8	1.4	1.3
Nocardioidaceae (41)	1.3	1.1	1.1	1.3	1.2	1.4	1.1	1.3	1.3	1.2	0.8	1.1	1.2	1.1	1.3	1.4	1.0	0.7	0.6	0.9	0.9	1.2	1.3
Propionibacteriaceae (11)	0.2	0.2	0.2	0.2	0.2	0.2	0.1	0.2	0.2	0.2	0.1	0.2	0.2	0.2	0.2	0.2	0.2	0.1	0.1	0.2	0.2	0.2	0.2
Pseudonocardiaceae (20)	0.8	0.7	0.7	0.6	0.6	0.7	0.5	0.7	0.6	0.7	0.4	0.5	0.6	0.6	0.7	0.7	0.5	0.4	0.4	0.5	0.6	0.6	0.7
Streptomycetaceae (5)	0.5	0.4	0.4	0.5	0.4	0.5	0.4	0.4	0.5	0.5	0.3	0.4	0.4	0.4	0.5	0.5	0.5	0.4	0.3	0.3	0.3	0.5	0.5
Nocardiopsaceae (1)	0.0	0.0	0.0	0.0	0.0	0.0	0.0	0.0	0.0	0.0	0.0	0.0	0.0	0.0	0.0	0.0	0.0	0.0	0.0	0.0	0.0	0.0	0.0
Streptosporangiaceae (4)	0.0	0.0	0.0	0.0	0.0	0.0	0.0	0.0	0.0	0.0	0.0	0.0	0.0	0.0	0.0	0.0	0.0	0.0	0.0	0.0	0.0	0.1	0.0
Thermomonosporaceae (15)	0.0	0.0	0.0	0.0	0.0	0.0	0.0	0.0	0.0	0.0	0.0	0.0	0.0	0.0	0.0	0.0	0.0	0.0	0.0	0.0	0.0	0.0	0.0
Unassigned *Frankiales* (2)	0.0	0.0	0.0	0.0	0.0	0.0	0.0	0.0	0.0	0.0	0.0	0.0	0.0	0.0	0.0	0.0	0.0	0.0	0.0	0.0	0.0	0.0	0.0
Unassigned *Actinobacteria* (2)	0.2	0.1	0.1	0.2	0.2	0.2	0.1	0.2	0.2	0.1	0.1	0.1	0.2	0.2	0.2	0.2	0.2	0.1	0.1	0.2	0.2	0.2	0.2
Coriobacteriia,																							
Coriobacteriaceae (1)	0.0	0.0	0.0	0.0	0.0	0.0	0.0	0.0	0.0	0.0	0.0	0.0	0.0	0.0	0.0	0.0	0.0	0.0	0.0	0.0	0.0	0.0	0.0
Rubrobacteria,																							
Rubrobacteriaceae (6)	0.1	0.1	0.1	0.1	0.1	0.1	0.1	0.1	0.1	0.1	0.1	0.1	0.1	0.1	0.1	0.1	0.1	0.1	0.1	0.1	0.1	0.1	0.1
Thermoleophilia,																							
Gaiellaceae (11)	0.8	0.7	0.7	0.7	0.7	0.8	0.7	0.7	0.7	0.8	0.5	0.7	0.8	0.7	0.8	0.9	0.8	0.5	0.5	0.6	0.6	0.7	0.9
Unassigned *Gaiellales* (72)	1.4	1.3	1.3	1.3	1.3	1.4	1.4	1.4	1.4	1.5	0.9	1.2	1.4	1.2	1.5	1.6	1.3	0.9	0.9	1.1	1.1	1.4	1.5
Conexibacteraceae (3)	0.0	0.0	0.0	0.0	0.0	0.0	0.0	0.0	0.0	0.0	0.0	0.0	0.0	0.0	0.0	0.0	0.0	0.0	0.0	0.0	0.0	0.0	0.0
Parviterribacteraceae (3)	0.0	0.0	0.0	0.0	0.0	0.0	0.0	0.0	0.0	0.0	0.0	0.0	0.0	0.0	0.0	0.0	0.0	0.0	0.0	0.0	0.0	0.0	0.0
Patulibacteraceae (15)	0.0	0.0	0.0	0.0	0.0	0.0	0.0	0.0	0.0	0.0	0.0	0.0	0.0	0.0	0.0	0.0	0.0	0.0	0.0	0.0	0.0	0.0	0.0
Solirubrobacteraceae (15)	1.0	1.0	0.9	1.0	0.9	1.0	1.0	1.1	1.0	1.0	0.7	0.9	1.0	1.1	1.2	1.3	1.0	0.7	0.7	0.8	0.8	1.0	1.1
Unassigned *Solirubrobacterales* (26)	0.4	0.3	0.3	0.4	0.4	0.4	0.3	0.4	0.4	0.4	0.3	0.3	0.4	0.4	0.4	0.5	0.4	0.2	0.2	0.3	0.3	0.4	0.4
Unassigned *Thermoleophilia* (67)	0.5	0.4	0.4	0.4	0.5	0.6	0.3	0.4	0.5	0.5	0.3	0.5	0.5	0.5	0.6	0.6	0.6	0.3	0.3	0.3	0.4	0.6	0.5
Unassigned *Actinobacteria* (34)	0.4	0.4	0.4	0.4	0.4	0.5	0.5	0.5	0.5	0.5	0.3	0.4	0.5	0.4	0.5	0.5	0.4	0.3	0.2	0.3	0.4	0.5	0.5

Sampling Time:	0 h									10 h							22 h						
Treatment:	C1	C2	C3	CAA	Glu	Asp	Thr	Ala/ Gly	Val/ Gly	C	CAA	Glu	Asp	Thr	Ala/ Gly	Val/ Gly	C	CAA	Glu	Asp	Thr	Ala/ Gly	Val/ Gly
Phyla, Class, Family[b]	Relative Abundance (%)																						
Armatimonadetes,																							
Armatimonadia,																							
Unassigned *Armatimonadales* (3)	0.0	0.0	0.0	0.0	0.0	0.0	0.0	0.0	0.0	0.0	0.0	0.0	0.0	0.0	0.0	0.0	0.0	0.0	0.0	0.0	0.0	0.0	0.0
Chthonomonadaceae (2)	0.0	0.0	0.0	0.0	0.0	0.0	0.0	0.0	0.0	0.0	0.0	0.0	0.0	0.0	0.0	0.0	0.0	0.0	0.0	0.0	0.0	0.0	0.0
Unassigned *Chthonomonadales* (2)	0.0	0.0	0.0	0.0	0.0	0.0	0.0	0.0	0.0	0.0	0.0	0.0	0.0	0.0	0.0	0.0	0.0	0.0	0.0	0.0	0.0	0.0	0.0
Unassigned *Armatimonadetes* (21)	0.0	0.0	0.0	0.0	0.0	0.0	0.0	0.0	0.0	0.0	0.0	0.0	0.0	0.0	0.0	0.0	0.0	0.0	0.0	0.0	0.0	0.0	0.0
Bacteroidetes,																							
Bacteroidia,																							
Prolixibacteraceae (1)	0.0	0.0	0.0	0.0	0.0	0.0	0.0	0.0	0.0	0.0	0.0	0.0	0.0	0.0	0.0	0.0	0.0	0.0	0.0	0.0	0.0	0.0	0.0
Cytophagia (2)	0.0	0.0	0.0	0.0	0.0	0.0	0.0	0.0	0.0	0.0	0.0	0.0	0.0	0.0	0.0	0.0	0.0	0.0	0.0	0.0	0.0	0.0	0.0
Cytophagaceae (33)	0.1	0.1	0.1	0.1	0.1	0.1	0.1	0.1	0.1	0.1	0.0	0.1	0.1	0.1	0.1	0.1	0.1	0.0	0.0	0.1	0.1	0.1	0.1
Flammeovirgaceae (1)	0.0	0.0	0.0	0.0	0.0	0.0	0.0	0.0	0.0	0.0	0.0	0.0	0.0	0.0	0.0	0.0	0.0	0.0	0.0	0.0	0.0	0.0	0.0
Flavobacteriia,																							
Flavobacteriaceae (21)	0.9	0.9	0.9	0.9	0.9	0.8	0.6	0.5	0.7	1.2	0.5	0.7	0.8	0.8	0.3	0.4	1.1	0.5	0.5	0.9	1.0	0.1	0.2
Sphingobacteriia,																							
Chitinophagaceae (48)	0.2	0.1	0.1	0.1	0.1	0.2	0.1	0.1	0.1	0.1	0.1	0.1	0.1	0.1	0.1	0.1	0.1	0.0	0.0	0.1	0.1	0.1	0.1
Lentimicrobiaceae (1)	0.0	0.0	0.0	0.0	0.0	0.0	0.0	0.0	0.0	0.0	0.0	0.0	0.0	0.0	0.0	0.0	0.0	0.0	0.0	0.0	0.0	0.0	0.0
Saprospiraceae (2)	0.0	0.0	0.0	0.0	0.0	0.0	0.0	0.0	0.0	0.0	0.0	0.0	0.0	0.0	0.0	0.0	0.0	0.0	0.0	0.0	0.0	0.0	0.0
Sphingobacteriaceae (3)	0.0	0.0	0.0	0.0	0.0	0.0	0.0	0.0	0.0	0.0	0.0	0.0	0.0	0.0	0.0	0.0	0.0	0.0	0.0	0.0	0.0	0.0	0.0
Unassigned *Sphingobacteriales* (14)	0.0	0.0	0.0	0.0	0.0	0.0	0.0	0.0	0.0	0.0	0.0	0.0	0.0	0.0	0.0	0.0	0.0	0.0	0.0	0.0	0.0	0.0	0.0
Unassigned *Sphingobacteriia* (10)	0.0	0.0	0.0	0.0	0.0	0.0	0.0	0.0	0.0	0.0	0.0	0.0	0.0	0.0	0.0	0.0	0.0	0.0	0.0	0.0	0.0	0.0	0.0
Unassigned *Bacteroidetes* (3)	0.0	0.0	0.0	0.0	0.0	0.0	0.0	0.0	0.0	0.0	0.0	0.0	0.0	0.0	0.0	0.0	0.0	0.0	0.0	0.0	0.0	0.0	0.0
BJ-169,																							
Unassigned *BJ-169* (6)	0.0	0.0	0.0	0.0	0.0	0.0	0.0	0.0	0.0	0.0	0.0	0.0	0.0	0.0	0.0	0.0	0.0	0.0	0.0	0.0	0.0	0.0	0.0
BRC1,																							
Unassigned *BCR1* (14)	0.0	0.0	0.0	0.0	0.0	0.0	0.0	0.0	0.0	0.0	0.0	0.0	0.0	0.0	0.0	0.0	0.0	0.0	0.0	0.0	0.0	0.0	0.0
Chlamydiae,																							
Chlamydiae,																							
Chlamydiaceae (4)	0.0	0.0	0.0	0.0	0.0	0.0	0.0	0.0	0.0	0.0	0.0	0.0	0.0	0.0	0.0	0.0	0.0	0.0	0.0	0.0	0.0	0.0	0.0
Parachlamydiaceae (154)	0.1	0.1	0.0	0.1	0.1	0.1	0.1	0.0	0.1	0.0	0.0	0.0	0.0	0.1	0.1	0.1	0.1	0.0	0.0	0.0	0.0	0.1	0.1
Simkaniaceae (11)	0.0	0.0	0.0	0.0	0.0	0.0	0.0	0.0	0.0	0.0	0.0	0.0	0.0	0.0	0.0	0.0	0.0	0.0	0.0	0.0	0.0	0.0	0.0
Waddliaceae (1)	0.0	0.0	0.0	0.0	0.0	0.0	0.0	0.0	0.0	0.0	0.0	0.0	0.0	0.0	0.0	0.0	0.0	0.0	0.0	0.0	0.0	0.0	0.0
Unassigned *Chlamydiales* (7)	0.0	0.0	0.0	0.0	0.0	0.0	0.0	0.0	0.0	0.0	0.0	0.0	0.0	0.0	0.0	0.0	0.0	0.0	0.0	0.0	0.0	0.0	0.0
Chlorobi,																							
Chlorobia,																							
Unassigned *Chlorobia* (4)	0.0	0.0	0.0	0.0	0.0	0.0	0.0	0.0	0.0	0.0	0.0	0.0	0.0	0.0	0.0	0.0	0.0	0.0	0.0	0.0	0.0	0.0	0.0
Chloroflexi,																							
Anaerolineae,																							
Anaerolineaceae (14)	0.0	0.0	0.0	0.0	0.0	0.0	0.0	0.0	0.0	0.0	0.0	0.0	0.0	0.0	0.0	0.0	0.0	0.0	0.0	0.0	0.0	0.0	0.0
Unassigned *Ardenticatenia* (3)	0.0	0.0	0.0	0.0	0.0	0.0	0.0	0.0	0.0	0.0	0.0	0.0	0.0	0.0	0.0	0.0	0.0	0.0	0.0	0.0	0.0	0.0	0.0
Caldilineae,																							
Caldilineaceae (15)	0.1	0.1	0.1	0.1	0.1	0.1	0.1	0.1	0.1	0.1	0.1	0.1	0.1	0.1	0.1	0.1	0.1	0.1	0.1	0.0	0.1	0.1	0.1
Chloroflexia,																							
Roseiflexaceae (8)	0.1	0.1	0.1	0.1	0.1	0.1	0.1	0.2	0.1	0.1	0.1	0.1	0.1	0.1	0.2	0.2	0.2	0.1	0.1	0.1	0.1	0.2	0.2
Unassigned *Chloroflexia* (4)	0.0	0.0	0.0	0.0	0.0	0.0	0.0	0.0	0.0	0.0	0.0	0.0	0.0	0.0	0.0	0.0	0.0	0.0	0.0	0.0	0.0	0.0	0.0
Ktedonobacteria,																							
Ktedonobacterales,																							
Ktedonobacteraceae (9)	0.0	0.0	0.0	0.0	0.0	0.0	0.0	0.0	0.0	0.0	0.0	0.0	0.0	0.0	0.0	0.0	0.0	0.0	0.0	0.0	0.0	0.0	0.0

Sampling Time:	0 h									10 h							22 h						
Treatment:	C1	C2	C3	CAA	Glu	Asp	Thr	Ala/ Gly	Val/ Gly	C	CAA	Glu	Asp	Thr	Ala/ Gly	Val/ Gly	C	CAA	Glu	Asp	Thr	Ala/ Gly	Val/ Gly
**Phyla, Class, Family**[b]	Relative Abundance (%)																						
Thermosporotrichaceae (7)	0.0	0.0	0.0	0.0	0.0	0.0	0.0	0.0	0.0	0.0	0.0	0.0	0.0	0.0	0.0	0.0	0.0	0.0	0.0	0.0	0.0	0.0	0.0
Unassigned *Ktedonobacterales* (6)	0.0	0.0	0.0	0.0	0.0	0.0	0.0	0.0	0.0	0.0	0.0	0.0	0.0	0.0	0.0	0.0	0.0	0.0	0.0	0.0	0.0	0.0	0.0
Unassigned *Ktedonobacteria* (18)	0.1	0.1	0.1	0.1	0.1	0.1	0.1	0.1	0.1	0.1	0.1	0.1	0.1	0.1	0.1	0.1	0.1	0.1	0.1	0.1	0.1	0.1	0.1
Thermomicrobia,																							
Thermomicrobiaceae (1)	0.0	0.0	0.0	0.0	0.0	0.0	0.0	0.0	0.0	0.0	0.0	0.0	0.0	0.0	0.0	0.0	0.0	0.0	0.0	0.0	0.0	0.0	0.0
Unassigned *Thermomicrobia* (56)	0.2	0.2	0.2	0.2	0.3	0.3	0.2	0.2	0.2	0.3	0.1	0.2	0.2	0.2	0.2	0.3	0.2	0.1	0.1	0.2	0.2	0.3	0.2
Unassigned *Chloroflexi* (111)	1.3	1.2	1.1	1.3	1.1	1.2	1.2	1.3	1.3	1.4	0.8	1.0	1.2	1.1	1.2	1.4	1.2	0.7	0.8	0.9	0.9	1.1	1.2
Cyanobacteria,																							
Chloroplast,																							
Unassigned *Chloroplast* (34)	0.0	0.0	0.0	0.0	0.0	0.0	0.0	0.0	0.0	0.0	0.0	0.0	0.0	0.0	0.0	0.0	0.0	0.0	0.0	0.0	0.0	0.0	0.0
Cyanobacteria,																							
Unassigned *Cyanobacteria* (2)	0.0	0.0	0.0	0.0	0.0	0.0	0.0	0.0	0.0	0.0	0.0	0.0	0.0	0.0	0.0	0.0	0.0	0.0	0.0	0.0	0.0	0.0	0.0
Gastranaerophilales,																							
Unassigned *Gastranaerophilales* (1)	0.0	0.0	0.0	0.0	0.0	0.0	0.0	0.0	0.0	0.0	0.0	0.0	0.0	0.0	0.0	0.0	0.0	0.0	0.0	0.0	0.0	0.0	0.0
Obscuribacterales,																							
Unassigned *Obscuribacterales* (6)	0.0	0.0	0.0	0.0	0.0	0.0	0.0	0.0	0.0	0.0	0.0	0.0	0.0	0.0	0.0	0.0	0.0	0.0	0.0	0.0	0.0	0.0	0.0
Unassigned *Cyanobacteria* (11)	0.0	0.0	0.0	0.0	0.0	0.0	0.0	0.0	0.0	0.0	0.0	0.0	0.0	0.0	0.0	0.0	0.0	0.0	0.0	0.0	0.0	0.0	0.0
Deferribacteres,																							
Unassigned *Deferribacteres* (1)	0.0	0.0	0.0	0.0	0.0	0.0	0.0	0.0	0.0	0.0	0.0	0.0	0.0	0.0	0.0	0.0	0.0	0.0	0.0	0.0	0.0	0.0	0.0
Deinococcus-Thermus,																							
Deinococci,																							
Unassigned *Deinococci* (4)	0.0	0.0	0.0	0.0	0.0	0.0	0.0	0.0	0.0	0.0	0.0	0.0	0.0	0.0	0.0	0.0	0.0	0.0	0.0	0.0	0.0	0.0	0.0
Elusimicrobia,																							
Unassigned *Elusimicrobia,*	0.0	0.0	0.0	0.0	0.0	0.0	0.0	0.0	0.0	0.0	0.0	0.0	0.0	0.0	0.0	0.0	0.0	0.0	0.0	0.0	0.0	0.0	0.0
Euryarchaeota,																							
Methanomicrobia,																							
Methanosarcinaceae (1)	0.0	0.0	0.0	0.0	0.0	0.0	0.0	0.0	0.0	0.0	0.0	0.0	0.0	0.0	0.0	0.0	0.0	0.0	0.0	0.0	0.0	0.0	0.0
Thermoplasmata,																							
Unassinged Thermoplasmatales (1)	0.0	0.0	0.0	0.0	0.0	0.0	0.0	0.0	0.0	0.0	0.0	0.0	0.0	0.0	0.0	0.0	0.0	0.0	0.0	0.0	0.0	0.0	0.0
Fibrobacteres,																							
Fibrobacteria,																							
Fibrobacteraceae (5)	0.0	0.0	0.0	0.0	0.0	0.0	0.0	0.0	0.0	0.0	0.0	0.0	0.0	0.0	0.0	0.0	0.0	0.0	0.0	0.0	0.0	0.0	0.0
Unassigned *Fibrobacterales* (3)	0.0	0.0	0.0	0.0	0.0	0.0	0.0	0.0	0.0	0.0	0.0	0.0	0.0	0.0	0.0	0.0	0.0	0.0	0.0	0.0	0.0	0.0	0.0
Firmicutes,																							
Bacilli,																							
Alicyclobacillaceae (5)	0.0	0.0	0.0	0.0	0.0	0.0	0.0	0.0	0.0	0.0	0.0	0.0	0.0	0.0	0.0	0.0	0.0	0.0	0.0	0.0	0.0	0.0	0.0
Bacillaceae (38)	0.7	0.6	0.6	0.6	0.5	0.6	0.5	0.5	0.6	0.5	0.4	0.4	0.5	0.5	0.6	0.6	0.5	0.5	0.4	0.5	0.6	0.7	0.8
Paenibacillaceae (88)	0.2	0.1	0.1	0.2	0.2	0.2	0.2	0.1	0.2	0.1	0.1	0.1	0.2	0.1	0.1	0.2	0.2	0.1	0.1	0.2	0.1	0.3	0.2
Pasteuriaceae (6)	0.0	0.0	0.0	0.0	0.0	0.0	0.0	0.0	0.0	0.0	0.0	0.0	0.0	0.0	0.0	0.0	0.0	0.0	0.0	0.0	0.0	0.0	0.0
Planococcaceae (7)	0.1	0.1	0.0	0.0	0.0	0.1	0.0	0.0	0.0	0.0	0.0	0.0	0.1	0.0	0.0	0.1	0.0	0.0	0.0	0.0	0.0	0.0	0.1
Sporolactobacillaceae (1)	0.0	0.0	0.0	0.0	0.0	0.0	0.0	0.0	0.0	0.0	0.0	0.0	0.0	0.0	0.0	0.0	0.0	0.0	0.0	0.0	0.0	0.0	0.0
Staphylococcaceae (1)	0.0	0.0	0.0	0.0	0.0	0.0	0.0	0.0	0.0	0.0	0.0	0.0	0.0	0.0	0.0	0.0	0.0	0.0	0.0	0.0	0.0	0.0	0.0
Thermoactinomycetaceae (18)	0.0	0.0	0.0	0.0	0.0	0.0	0.0	0.0	0.0	0.0	0.0	0.0	0.0	0.0	0.0	0.0	0.0	0.0	0.0	0.0	0.0	0.0	0.0
Streptococcaceae (1)	0.0	0.0	0.0	0.0	0.0	0.0	0.0	0.0	0.0	0.0	0.0	0.0	0.0	0.0	0.0	0.0	0.0	0.0	0.0	0.0	0.0	0.0	0.0
Unassigned *Bacilli* (13)	0.8	0.8	0.7	0.8	0.7	0.8	0.7	0.7	0.7	0.5	0.4	0.4	0.5	0.4	0.5	0.6	0.4	0.3	0.2	0.3	0.2	0.4	0.4
Clostridia,																							
Caldicoprobacteraceae (7)	0.0	0.0	0.0	0.0	0.0	0.0	0.0	0.0	0.0	0.0	0.0	0.0	0.0	0.0	0.0	0.0	0.0	0.0	0.0	0.0	0.0	0.0	0.0
Christensenellaceae (7)	0.0	0.0	0.0	0.0	0.0	0.0	0.0	0.0	0.0	0.0	0.0	0.0	0.0	0.0	0.0	0.0	0.0	0.0	0.0	0.0	0.0	0.0	0.0

Sampling Time:	0 h									10 h							22 h						
Treatment:	C1	C2	C3	CAA	Glu	Asp	Thr	Ala/ Gly	Val/ Gly	C	CAA	Glu	Asp	Thr	Ala/ Gly	Val/ Gly	C	CAA	Glu	Asp	Thr	Ala/ Gly	Val/ Gly
Phyla, Class, Family[b]										Relative Abundance (%)													
Clostridiaceae (38) **[A14]**	0.2	0.2	0.1	0.2	0.2	0.2	0.1	0.2	0.2	0.3	0.4	0.4	0.3	0.5	0.4	0.4	1.3	1.3	4.7	1.2	1.4	1.7	1.3
Defluviitaleaceae (4)	0.0	0.0	0.0	0.0	0.0	0.0	0.0	0.0	0.0	0.0	0.0	0.0	0.0	0.0	0.0	0.0	0.0	0.0	0.0	0.0	0.0	0.0	0.0
Eubacteriaceae (4)	0.0	0.0	0.0	0.0	0.0	0.0	0.0	0.0	0.0	0.0	0.0	0.0	0.0	0.0	0.0	0.0	0.0	0.0	0.0	0.0	0.0	0.0	0.0
Gracilibacteraceae (7)	0.0	0.0	0.0	0.0	0.0	0.0	0.0	0.0	0.0	0.0	0.0	0.0	0.0	0.0	0.0	0.0	0.0	0.0	0.0	0.0	0.0	0.0	0.0
Heliobacteriaceae (22)	0.0	0.0	0.0	0.0	0.0	0.0	0.0	0.0	0.0	0.0	0.0	0.0	0.0	0.0	0.0	0.0	0.0	0.0	0.0	0.0	0.0	0.0	0.0
Lachnospiraceae (79)	0.1	0.1	0.0	0.1	0.1	0.1	0.1	0.1	0.1	0.1	0.1	0.1	0.1	0.1	0.1	0.1	0.5	0.3	0.2	0.4	0.3	1.0	0.9
Peptococcaceae (17)	2.0	1.9	1.7	1.9	1.9	2.0	1.9	1.5	1.7	1.3	1.0	1.3	1.3	1.5	2.0	1.7	2.0	1.0	1.0	1.1	1.1	1.6	1.7
Peptostreptococcaceae (11) **[GPT-4]**, **[A8]**	0.1	0.1	0.1	0.1	0.1	0.2	0.1	0.1	0.2	0.4	1.2	0.4	0.3	0.8	0.8	0.8	1.1	2.3	0.7	0.3	3.6	5.5	2.9
Ruminococcaceae (96)	0.0	0.0	0.0	0.0	0.0	0.0	0.0	0.0	0.0	0.0	0.0	0.0	0.0	0.0	0.0	0.0	0.1	0.0	0.0	0.0	0.0	0.0	0.1
Syntrophomonadaceae (10)	0.0	0.0	0.0	0.0	0.0	0.0	0.0	0.0	0.0	0.0	0.0	0.0	0.0	0.0	0.0	0.0	0.0	0.0	0.0	0.0	0.0	0.0	0.0
Thermoanaerobacteraceae (8)	0.0	0.0	0.0	0.0	0.0	0.0	0.0	0.0	0.0	0.0	0.0	0.0	0.0	0.0	0.0	0.0	0.0	0.0	0.0	0.0	0.0	0.0	0.0
Unassigned *Clostridiales* (17)	0.0	0.0	0.0	0.0	0.0	0.0	0.0	0.0	0.0	0.0	0.0	0.0	0.0	0.0	0.0	0.0	0.1	0.1	0.0	0.1	0.0	0.0	0.0
Unassigned *Thermoanaerobacterales* (1)	0.0	0.0	0.0	0.0	0.0	0.0	0.0	0.0	0.0	0.0	0.0	0.0	0.0	0.0	0.0	0.0	0.0	0.0	0.0	0.0	0.0	0.0	0.0
Unassigned *Clostridia* (34)	0.0	0.0	0.0	0.0	0.0	0.0	0.0	0.0	0.0	0.0	0.0	0.0	0.0	0.0	0.0	0.0	0.0	0.0	0.0	0.0	0.0	0.0	0.0
Erysipelotrichia,																							
Erysipelotrichaceae (18)	0.0	0.0	0.0	0.0	0.0	0.0	0.0	0.0	0.0	0.0	0.0	0.0	0.0	0.0	0.0	0.0	0.0	0.0	0.0	0.0	0.0	0.0	0.0
Limnochordia,																							
Limnochordaceae (23)	0.0	0.0	0.0	0.0	0.0	0.0	0.0	0.0	0.0	0.0	0.0	0.0	0.0	0.0	0.0	0.0	0.0	0.0	0.0	0.0	0.0	0.0	0.0
Unassigned *Limnochordales* (3)	0.0	0.0	0.0	0.0	0.0	0.0	0.0	0.0	0.0	0.0	0.0	0.0	0.0	0.0	0.0	0.0	0.0	0.0	0.0	0.0	0.0	0.0	0.0
Negativicutes,																							
Veillonellaceae (20)	0.0	0.0	0.0	0.0	0.0	0.0	0.0	0.0	0.0	0.0	0.0	0.0	0.0	0.0	0.0	0.0	0.0	0.0	0.0	0.0	0.0	0.0	0.0
Unassigned *Selenomonadales* (3)	0.0	0.0	0.0	0.0	0.0	0.0	0.0	0.0	0.0	0.0	0.0	0.0	0.0	0.0	0.0	0.0	0.0	0.0	0.0	0.0	0.0	0.0	0.0
Unassigned *Firmicutes* (1)	0.0	0.0	0.0	0.0	0.0	0.0	0.0	0.0	0.0	0.0	0.0	0.0	0.0	0.0	0.0	0.0	0.0	0.0	0.0	0.0	0.0	0.0	0.0
Fusobacteria,																							
Fusobacteriia,																							
Fusobacteriaceae (10) [**GPT-5**]	8.6	13	13	9.6	13	10	12	10	10	20	33	29	20	19	13	13	20	33	34	25	22	12	15
Leptotrichiaceae (1)	0.0	0.0	0.0	0.0	0.0	0.0	0.0	0.0	0.0	0.0	0.0	0.0	0.0	0.0	0.0	0.0	0.0	0.0	0.0	0.0	0.0	0.0	0.0
Gemmatimonadetes,																							
Gemmatimonadetes,																							
Gemmatimonadaceae (37)	0.2	0.2	0.2	0.2	0.2	0.2	0.2	0.2	0.2	0.2	0.1	0.2	0.2	0.2	0.3	0.3	0.2	0.2	0.1	0.2	0.2	0.3	0.3
Longimicrobiaceae (1)	0.0	0.0	0.0	0.0	0.0	0.0	0.0	0.0	0.0	0.0	0.0	0.0	0.0	0.0	0.0	0.0	0.0	0.0	0.0	0.0	0.0	0.0	0.0
Unassigned *Gemmatimonadetes* (6)	0.0	0.0	0.0	0.0	0.0	0.0	0.0	0.0	0.0	0.0	0.0	0.0	0.0	0.0	0.0	0.0	0.0	0.0	0.0	0.0	0.0	0.0	0.0
Hydrogenedentes,																							
Unassigned *Hydrogenedentes* (2)	0.0	0.0	0.0	0.0	0.0	0.0	0.0	0.0	0.0	0.0	0.0	0.0	0.0	0.0	0.0	0.0	0.0	0.0	0.0	0.0	0.0	0.0	0.0
Latescibacteria,																							
Unassigned *Latescibacteria* (30)	0.0	0.0	0.0	0.0	0.0	0.0	0.0	0.0	0.0	0.0	0.0	0.0	0.0	0.0	0.0	0.0	0.0	0.0	0.0	0.0	0.0	0.0	0.0
Nitrospirae,																							
Nitrospira,																							
Nitrospiraceae (8)	0.2	0.2	0.2	0.2	0.2	0.2	0.1	0.1	0.1	0.1	0.1	0.1	0.1	0.2	0.2	0.2	0.2	0.1	0.1	0.1	0.1	0.2	0.2
Unassigned *Nitrospira* (18)	0.5	0.4	0.4	0.4	0.4	0.5	0.5	0.6	0.6	0.5	0.3	0.4	0.4	0.4	0.5	0.6	0.5	0.3	0.4	0.3	0.4	0.6	0.5
Parcubacteria,																							
Unassigned *Parcubacteria* (1)	0.0	0.0	0.0	0.0	0.0	0.0	0.0	0.0	0.0	0.0	0.0	0.0	0.0	0.0	0.0	0.0	0.0	0.0	0.0	0.0	0.0	0.0	0.0
Planctomycetes,																							
Phycisphaerae,																							
Phycisphaeraceae (38)	0.0	0.0	0.0	0.0	0.0	0.0	0.0	0.0	0.0	0.0	0.0	0.0	0.0	0.0	0.0	0.0	0.0	0.0	0.0	0.0	0.0	0.0	0.0
Tepidisphaeraceae (82)	0.6	0.6	0.5	0.5	0.6	0.6	0.7	0.6	0.5	0.5	0.3	0.4	0.5	0.5	0.7	0.6	0.5	0.2	0.3	0.4	0.4	0.6	0.5
Unassigned *Phycisphaerales* (4)	0.0	0.0	0.0	0.0	0.0	0.0	0.0	0.0	0.0	0.0	0.0	0.0	0.0	0.0	0.0	0.0	0.0	0.0	0.0	0.0	0.0	0.0	0.0
Unassigned *Phycisphaerae* (7)	0.0	0.0	0.0	0.0	0.0	0.0	0.0	0.0	0.0	0.0	0.0	0.0	0.0	0.0	0.0	0.0	0.0	0.0	0.0	0.0	0.0	0.0	0.0

Sampling Time:	0 h									10 h							22 h						
Treatment:	C1	C2	C3	CAA	Glu	Asp	Thr	Ala/ Gly	Val/ Gly	C	CAA	Glu	Asp	Thr	Ala/ Gly	Val/ Gly	C	CAA	Glu	Asp	Thr	Ala/ Gly	Val/ Gly
**Phyla, Class, Family**[b]										Relative Abundance (%)													
Planctomycetacia,																							
Planctomycetaceae (733)	8.1	8.1	7.3	7.5	8.0	7.8	6.5	7.2	8.0	8.5	4.9	6.4	6.6	7.7	10	9.1	8.1	4.6	4.6	5.5	7.3	10	9.3
Unassigned *Planctomycetes* (82)	0.1	0.1	0.1	0.1	0.1	0.1	0.1	0.1	0.1	0.1	0.0	0.0	0.0	0.0	0.1	0.1	0.1	0.0	0.0	0.0	0.0	0.1	0.1
Proteobacteria,																							
Alphaproteobacteria,																							
Caulobacteraceae (5)	0.0	0.0	0.0	0.0	0.0	0.0	0.0	0.0	0.0	0.0	0.0	0.0	0.0	0.0	0.0	0.0	0.0	0.0	0.0	0.0	0.0	0.0	0.0
Beijerinckiaceae (2)	0.0	0.0	0.0	0.0	0.0	0.0	0.0	0.0	0.0	0.0	0.0	0.0	0.0	0.0	0.0	0.0	0.0	0.0	0.0	0.0	0.0	0.0	0.0
Bradyrhizobiaceae (5)	2.0	1.7	1.7	1.6	1.6	1.7	0.9	1.3	1.4	1.0	1.1	1.2	1.1	1.4	1.6	1.6	1.5	1.0	0.8	0.9	1.4	1.5	1.5
Hyphomicrobiaceae (8)	0.3	0.2	0.2	0.2	0.2	0.2	0.1	0.2	0.2	0.1	0.2	0.2	0.2	0.2	0.2	0.2	0.2	0.1	0.1	0.1	0.2	0.2	0.2
Methylobacteriaceae (4)	0.2	0.2	0.1	0.1	0.2	0.2	0.1	0.1	0.2	0.1	0.1	0.1	0.1	0.1	0.2	0.2	0.2	0.1	0.1	0.1	0.2	0.2	0.2
Methylocystaceae (1)	0.0	0.0	0.0	0.0	0.0	0.0	0.0	0.0	0.0	0.0	0.0	0.0	0.0	0.0	0.0	0.0	0.0	0.0	0.0	0.0	0.0	0.0	0.0
Phyllobacteriaceae (3)	0.3	0.3	0.3	0.3	0.2	0.3	0.2	0.2	0.3	0.2	0.2	0.2	0.2	0.2	0.3	0.3	0.2	0.2	0.1	0.2	0.2	0.2	0.2
Rhizobiaceae (3)	0.0	0.0	0.0	0.0	0.0	0.0	0.0	0.0	0.0	0.0	0.0	0.0	0.0	0.0	0.0	0.0	0.0	0.0	0.0	0.0	0.0	0.0	0.0
Rhodobiaceae (2)	0.3	0.3	0.2	0.3	0.3	0.4	0.3	0.3	0.3	0.3	0.2	0.2	0.3	0.2	0.3	0.3	0.3	0.2	0.1	0.2	0.2	0.4	0.3
Roseiarcaceae (1)	0.0	0.0	0.0	0.0	0.0	0.0	0.0	0.0	0.0	0.0	0.0	0.0	0.0	0.0	0.0	0.0	0.0	0.0	0.0	0.0	0.0	0.0	0.0
Xanthobacteraceae (9)	1.3	1.1	1.1	1.2	1.1	1.3	1.2	1.1	1.3	1.0	0.8	1.0	1.1	1.0	1.4	1.3	1.1	0.8	0.7	0.9	0.9	1.4	1.4
Unassigned *Rhizobiales* (19)	0.3	0.2	0.2	0.2	0.2	0.3	0.2	0.2	0.2	0.2	0.1	0.2	0.2	0.2	0.2	0.2	0.2	0.2	0.1	0.2	0.2	0.2	0.3
Rhodobacteraceae (6)	0.1	0.0	0.1	0.1	0.1	0.1	0.0	0.1	0.1	0.0	0.0	0.0	0.0	0.1	0.1	0.1	0.1	0.0	0.0	0.0	0.0	0.0	0.1
Acetobacteraceae (15)	0.2	0.2	0.2	0.2	0.2	0.2	0.2	0.2	0.2	0.2	0.1	0.2	0.2	0.2	0.2	0.2	0.2	0.1	0.1	0.2	0.2	0.2	0.2
Rhodospirillaceae (19)	0.6	0.5	0.5	0.5	0.5	0.5	0.4	0.5	0.6	0.4	0.3	0.4	0.4	0.5	0.6	0.6	0.4	0.3	0.3	0.4	0.6	0.5	0.6
Unassigned *Rhodospirillales* (25)	0.6	0.6	0.5	0.5	0.5	0.5	0.4	0.6	0.6	0.5	0.4	0.4	0.4	0.5	0.6	0.6	0.5	0.4	0.4	0.4	0.6	0.6	0.7
Anaplasmataceae (1)	0.0	0.0	0.0	0.0	0.0	0.0	0.0	0.0	0.0	0.0	0.0	0.0	0.0	0.0	0.0	0.0	0.0	0.0	0.0	0.0	0.0	0.0	0.0
Holosporaceae (2)	0.0	0.0	0.0	0.0	0.0	0.0	0.0	0.0	0.0	0.0	0.0	0.0	0.0	0.0	0.0	0.0	0.0	0.0	0.0	0.0	0.0	0.0	0.0
Mitochondria (2)	0.0	0.0	0.0	0.0	0.0	0.0	0.0	0.0	0.0	0.0	0.0	0.0	0.0	0.0	0.0	0.0	0.0	0.0	0.0	0.0	0.0	0.0	0.0
Rickettsiaceae (1)	0.0	0.0	0.0	0.0	0.0	0.0	0.0	0.0	0.0	0.0	0.0	0.0	0.0	0.0	0.0	0.0	0.0	0.0	0.0	0.0	0.0	0.0	0.0
Unassigned *Rickettsiales* (7)	0.0	0.0	0.0	0.0	0.0	0.0	0.0	0.0	0.0	0.0	0.0	0.0	0.0	0.0	0.0	0.0	0.0	0.0	0.0	0.0	0.0	0.0	0.0
Erythrobacteraceae (1)	0.0	0.0	0.0	0.0	0.0	0.0	0.0	0.0	0.0	0.0	0.0	0.0	0.0	0.0	0.0	0.0	0.0	0.0	0.0	0.0	0.0	0.0	0.0
Sphingomonadaceae (5)	0.0	0.0	0.0	0.0	0.0	0.0	0.0	0.0	0.0	0.0	0.0	0.0	0.0	0.0	0.0	0.0	0.0	0.0	0.0	0.0	0.0	0.0	0.0
Unassigned *Sphingomonadales* (2)	0.0	0.0	0.0	0.0	0.0	0.0	0.0	0.0	0.0	0.0	0.0	0.0	0.0	0.0	0.0	0.0	0.0	0.0	0.0	0.0	0.0	0.0	0.0
Unassigned *Alphaproteobacteria* (19)	0.2	0.2	0.2	0.2	0.2	0.2	0.1	0.2	0.2	0.1	0.1	0.1	0.1	0.1	0.2	0.2	0.2	0.1	0.1	0.1	0.2	0.2	0.2
Betaproteobacteria,																							
Alcaligenaceae (2)	0.1	0.1	0.1	0.1	0.1	0.1	0.1	0.1	0.1	0.1	0.0	0.1	0.1	0.1	0.1	0.1	0.1	0.0	0.0	0.1	0.1	0.1	0.1
Burkholderiaceae (10)	0.0	0.0	0.0	0.0	0.0	0.0	0.0	0.0	0.0	0.0	0.0	0.0	0.0	0.0	0.0	0.0	0.0	0.0	0.0	0.0	0.0	0.0	0.0
Comamonadaceae (17)	0.4	0.3	0.3	0.4	0.3	0.4	0.3	0.3	0.3	0.2	0.2	0.2	0.2	0.2	0.3	0.3	0.3	0.1	0.1	0.2	0.2	0.2	0.3
Oxalobacteraceae (15)	0.0	0.0	0.0	0.0	0.0	0.0	0.0	0.0	0.0	0.0	0.0	0.0	0.0	0.0	0.0	0.0	0.0	0.0	0.0	0.0	0.0	0.0	0.0
Neisseriaceae (1)	0.0	0.0	0.0	0.0	0.0	0.0	0.0	0.0	0.0	0.0	0.0	0.0	0.0	0.0	0.0	0.0	0.0	0.0	0.0	0.0	0.0	0.0	0.0
Gallionellaceae (3)	0.0	0.0	0.0	0.0	0.0	0.0	0.0	0.0	0.0	0.0	0.0	0.0	0.0	0.0	0.0	0.0	0.0	0.0	0.0	0.0	0.0	0.0	0.0
Nitrosomonadaceae (30)	0.2	0.2	0.2	0.2	0.1	0.2	0.1	0.2	0.2	0.1	0.1	0.1	0.1	0.2	0.2	0.2	0.2	0.1	0.1	0.1	0.1	0.2	0.2
Rhodocyclaceae (9)	0.1	0.0	0.1	0.0	0.1	0.1	0.0	0.0	0.1	0.0	0.0	0.0	0.0	0.0	0.1	0.1	0.0	0.0	0.0	0.0	0.0	0.0	0.0
Unassigned *Betaproteobacteria* (41)	0.2	0.2	0.2	0.2	0.2	0.2	0.2	0.2	0.2	0.2	0.1	0.2	0.2	0.2	0.2	0.2	0.2	0.1	0.1	0.1	0.1	0.2	0.2
Deltaproteobacteria,																							
Bacteriovoracaceae (12)	0.0	0.0	0.0	0.0	0.0	0.0	0.0	0.0	0.0	0.0	0.0	0.0	0.0	0.0	0.0	0.0	0.0	0.0	0.0	0.0	0.0	0.0	0.0
Bdellovibrionaceae (54)	0.1	0.1	0.1	0.1	0.1	0.1	0.1	0.1	0.1	0.1	0.0	0.1	0.1	0.1	0.1	0.1	0.1	0.0	0.0	0.0	0.1	0.1	0.1
Desulfarculaceae (1)	0.0	0.0	0.0	0.0	0.0	0.0	0.0	0.0	0.0	0.0	0.0	0.0	0.0	0.0	0.0	0.0	0.0	0.0	0.0	0.0	0.0	0.0	0.0
Desulfobulbaceae (3)	0.0	0.0	0.0	0.0	0.0	0.0	0.0	0.0	0.0	0.0	0.0	0.0	0.0	0.0	0.0	0.0	0.0	0.0	0.0	0.0	0.0	0.0	0.0
Desulfurellaceae (41)	0.6	0.5	0.5	0.6	0.5	0.6	0.4	0.6	0.6	0.5	0.4	0.4	0.5	0.5	0.6	0.6	0.5	0.4	0.3	0.4	0.5	0.6	0.7
Desulfuromonadaceae (2)	0.0	0.0	0.0	0.0	0.0	0.0	0.0	0.0	0.0	0.0	0.0	0.0	0.0	0.0	0.0	0.0	0.0	0.0	0.0	0.0	0.0	0.0	0.0
Geobacteraceae (26)	0.2	0.1	0.1	0.1	0.1	0.1	0.1	0.1	0.1	0.1	0.1	0.1	0.1	0.1	0.1	0.1	0.1	0.1	0.1	0.1	0.1	0.1	0.2
Archangiaceae (20)	0.1	0.1	0.1	0.1	0.1	0.1	0.1	0.1	0.1	0.1	0.1	0.1	0.1	0.1	0.1	0.1	0.1	0.1	0.0	0.1	0.1	0.1	0.1
Haliangiaceae (74)	0.4	0.3	0.3	0.3	0.3	0.3	0.2	0.2	0.3	0.3	0.2	0.3	0.3	0.3	0.4	0.3	0.4	0.3	0.2	0.2	0.3	0.4	0.3

Sampling Time:	0 h									10 h							22 h						
**Treatment:**	**C1**	**C2**	**C3**	**CAA**	**Glu**	**Asp**	**Thr**	**Ala/ Gly**	**Val/ Gly**	**C**	**CAA**	**Glu**	**Asp**	**Thr**	**Ala/ Gly**	**Val/ Gly**	**C**	**CAA**	**Glu**	**Asp**	**Thr**	**Ala/ Gly**	**Val/ Gly**
**Phyla, Class, Family**[b]										**Relative Abundance (%)**													
Myxococcaceae (3)	0.0	0.0	0.0	0.0	0.0	0.0	0.0	0.0	0.0	0.0	0.0	0.0	0.0	0.0	0.0	0.0	0.0	0.0	0.0	0.0	0.0	0.0	0.0
Nannocystaceae (6)	0.0	0.0	0.0	0.0	0.0	0.0	0.0	0.0	0.0	0.0	0.0	0.0	0.0	0.0	0.0	0.0	0.0	0.0	0.0	0.0	0.0	0.0	0.0
Phaselicystidaceae (14)	0.1	0.1	0.1	0.1	0.1	0.1	0.1	0.2	0.1	0.1	0.1	0.1	0.1	0.1	0.1	0.1	0.1	0.1	0.1	0.1	0.1	0.2	0.2
Polyangiaceae (48)	0.4	0.4	0.3	0.3	0.3	0.3	0.3	0.4	0.3	0.3	0.2	0.2	0.3	0.3	0.4	0.3	0.3	0.2	0.2	0.2	0.3	0.4	0.3
Sandaracinaceae (38)	0.2	0.1	0.1	0.1	0.1	0.1	0.1	0.1	0.1	0.1	0.1	0.1	0.1	0.1	0.2	0.1	0.1	0.1	0.1	0.1	0.1	0.1	0.2
Vulgatibacteraceae (3)	0.0	0.0	0.0	0.0	0.0	0.0	0.0	0.0	0.0	0.0	0.0	0.0	0.0	0.0	0.0	0.0	0.0	0.0	0.0	0.0	0.0	0.0	0.0
Unassigned *Myxococcales* (177)	0.4	0.3	0.3	0.3	0.2	0.3	0.2	0.3	0.2	0.2	0.2	0.2	0.2	0.2	0.4	0.3	0.3	0.2	0.1	0.2	0.3	0.4	0.4
Oligoflexaceae (29)	0.0	0.0	0.0	0.0	0.0	0.0	0.0	0.0	0.0	0.0	0.0	0.0	0.0	0.0	0.0	0.0	0.0	0.0	0.0	0.0	0.0	0.0	0.0
Unassigned *Oligoflexales* (86)	0.0	0.0	0.0	0.0	0.0	0.0	0.0	0.0	0.0	0.0	0.0	0.0	0.0	0.0	0.0	0.0	0.0	0.0	0.0	0.0	0.0	0.0	0.0
Syntrophaceae (1)	0.0	0.0	0.0	0.0	0.0	0.0	0.0	0.0	0.0	0.0	0.0	0.0	0.0	0.0	0.0	0.0	0.0	0.0	0.0	0.0	0.0	0.0	0.0
Unassigned *Deltaproteobacteria* (19)	0.0	0.0	0.0	0.0	0.0	0.0	0.0	0.0	0.0	0.0	0.0	0.0	0.0	0.0	0.0	0.0	0.0	0.0	0.0	0.0	0.0	0.0	0.0
Gammaproteobacteria,																							
Acidiferrobacteraceae (3)	0.0	0.0	0.0	0.0	0.0	0.0	0.0	0.0	0.0	0.0	0.0	0.0	0.0	0.0	0.0	0.0	0.0	0.0	0.0	0.0	0.0	0.0	0.0
Aeromonadaceae (8) **[GPT-1]**	15	19	20	16	17	13	19	17	16	18	16	14	17	15	11	12	12	15	10	18	14	12	8.9
Shewanellaceae (3)	2.0	2.4	2.4	2.8	2.9	2.6	3.1	3.1	2.9	2.6	2.3	3.3	2.4	2.9	2.4	2.6	2.1	3.2	3.2	3.0	4.2	2.4	2.5
Cellvibrionaceae (2)	0.0	0.0	0.0	0.0	0.0	0.0	0.0	0.0	0.0	0.0	0.0	0.0	0.0	0.0	0.0	0.0	0.0	0.0	0.0	0.0	0.0	0.0	0.0
Halieaceae (4)	0.0	0.0	0.0	0.0	0.0	0.0	0.0	0.0	0.0	0.0	0.0	0.0	0.0	0.0	0.0	0.0	0.0	0.0	0.0	0.0	0.0	0.0	0.0
Enterobacteriaceae (12) **[GPT-2], [GPT-3]**	5.6	4.9	5.3	6.0	5.1	5.1	5.0	5.9	5.8	3.4	6.2	5.2	5.3	4.5	4.5	5.3	3.9	6.5	13	9.1	4.5	5.4	5.3
Coxiellaceae (35)	0.0	0.1	0.1	0.0	0.0	0.0	0.0	0.0	0.0	0.0	0.0	0.0	0.0	0.0	0.0	0.0	0.0	0.0	0.0	0.0	0.0	0.0	0.0
Legionellaceae (30)	0.0	0.0	0.0	0.0	0.0	0.0	0.0	0.0	0.0	0.0	0.0	0.0	0.0	0.0	0.0	0.0	0.0	0.0	0.0	0.0	0.0	0.0	0.0
Methylococcaceae (3)	0.0	0.0	0.0	0.0	0.0	0.0	0.0	0.0	0.0	0.0	0.0	0.0	0.0	0.0	0.0	0.0	0.0	0.0	0.0	0.0	0.0	0.0	0.0
Oleiphilaceae (1)	0.0	0.0	0.0	0.0	0.0	0.0	0.0	0.0	0.0	0.0	0.0	0.0	0.0	0.0	0.0	0.0	0.0	0.0	0.0	0.0	0.0	0.0	0.0
Moraxellaceae (2)	0.0	0.0	0.0	0.0	0.0	0.0	0.0	0.0	0.0	0.0	0.0	0.0	0.0	0.0	0.0	0.0	0.0	0.0	0.0	0.0	0.0	0.0	0.0
Pseudomonadaceae (4)	0.1	0.1	0.1	0.1	0.1	0.1	0.1	0.1	0.1	0.1	0.1	0.1	0.1	0.1	0.1	0.1	0.2	0.1	0.1	0.1	0.1	0.2	0.1
Unassigned Thiotrichales (1)	0.0	0.0	0.0	0.0	0.0	0.0	0.0	0.0	0.0	0.0	0.0	0.0	0.0	0.0	0.0	0.0	0.0	0.0	0.0	0.0	0.0	0.0	0.0
Xanthomonadaceae (12)	0.0	0.0	0.0	0.0	0.0	0.0	0.0	0.0	0.0	0.0	0.0	0.0	0.0	0.0	0.0	0.0	0.0	0.0	0.0	0.0	0.0	0.0	0.0
Unassigned *Xanthomonadales* (24)	0.2	0.1	0.2	0.2	0.1	0.2	0.1	0.2	0.2	0.1	0.1	0.1	0.2	0.1	0.2	0.2	0.2	0.1	0.1	0.1	0.1	0.2	0.2
Unassigned *Gammaproteobacteria* (30)	0.0	0.0	0.0	0.0	0.0	0.0	0.0	0.0	0.0	0.0	0.0	0.0	0.0	0.0	0.0	0.0	0.0	0.0	0.0	0.0	0.0	0.0	0.0
Unassigned *Proteobacteria* (2)	0.0	0.0	0.0	0.0	0.0	0.0	0.0	0.0	0.0	0.0	0.0	0.0	0.0	0.0	0.0	0.0	0.0	0.0	0.0	0.0	0.0	0.0	0.0
RsaHf231,																							
Unassigned *RsaHf231* (1)	0.0	0.0	0.0	0.0	0.0	0.0	0.0	0.0	0.0	0.0	0.0	0.0	0.0	0.0	0.0	0.0	0.0	0.0	0.0	0.0	0.0	0.0	0.0
Saccharibacteria,																							
Unassigned *Saccharibacteria* (78)	0.0	0.0	0.0	0.0	0.0	0.0	0.0	0.0	0.0	0.0	0.0	0.0	0.0	0.0	0.0	0.0	0.0	0.0	0.0	0.0	0.0	0.0	0.0
Spirochaetae,																							
Spirochaetes,																							
Spirochaetaceae (1)	0.0	0.0	0.0	0.0	0.0	0.0	0.0	0.0	0.0	0.0	0.0	0.0	0.0	0.0	0.0	0.0	0.0	0.0	0.0	0.0	0.0	0.0	0.0
Brevinemataceae (1)	0.0	0.0	0.0	0.0	0.0	0.0	0.0	0.0	0.0	0.0	0.0	0.0	0.0	0.0	0.0	0.0	0.0	0.0	0.0	0.0	0.0	0.0	0.0
Synergistetes,																							
Synergistia,																							
Synergistaceae (1)	0.0	0.0	0.0	0.0	0.0	0.0	0.0	0.0	0.0	0.0	0.0	0.0	0.0	0.0	0.0	0.0	0.0	0.0	0.0	0.0	0.0	0.0	0.0
Tectomicrobia,																							
Unassigned *Tectomicrobia* (21)	0.9	0.8	0.8	0.8	0.7	0.7	0.5	0.8	0.8	0.7	0.5	0.5	0.6	0.7	0.9	0.8	0.7	0.6	0.5	0.7	0.9	0.7	1.0
Tenericutes,																							
Mollicutes,																							
Unassigned *Entomoplasmatales* (3)	0.0	0.0	0.0	0.0	0.0	0.0	0.0	0.0	0.0	0.0	0.0	0.0	0.0	0.0	0.0	0.0	0.0	0.0	0.0	0.0	0.0	0.0	0.0
Haloplasmataceae (7)	0.0	0.0	0.0	0.0	0.0	0.0	0.0	0.0	0.0	0.0	0.0	0.0	0.0	0.0	0.0	0.0	0.0	0.0	0.0	0.0	0.0	0.0	0.0
Mycoplasmataceae (19)	21	18	18	22	21	23	24	21	21	13	13	14	17	17	21	19	17	12	9.4	13	14	14	17
Thaumarchaeota,																							

Sampling Time:	0 h									10 h							22 h						
Treatment:	C1	C2	C3	CAA	Glu	Asp	Thr	Ala/ Gly	Val/ Gly	C	CAA	Glu	Asp	Thr	Ala/ Gly	Val/ Gly	C	CAA	Glu	Asp	Thr	Ala/ Gly	Val/ Gly
**Phyla, Class, Family[b]**	Relative Abundance (%)																						
Unassigned *Thaumarchaeota* (9)	0.0	0.0	0.0	0.0	0.0	0.0	0.0	0.0	0.0	0.0	0.0	0.0	0.0	0.0	0.0	0.0	0.0	0.0	0.0	0.0	0.0	0.0	0.0
TM6_Dependentiae,																							
Unassigned *TM6_Dependentiae* (40)	0.0	0.0	0.0	0.0	0.0	0.0	0.0	0.0	0.0	0.0	0.0	0.0	0.0	0.0	0.0	0.0	0.0	0.0	0.0	0.0	0.0	0.0	0.0
Verrucomicrobia,																							
Unassigned OPB35 soil group (87)	0.2	0.2	0.2	0.2	0.2	0.3	0.2	0.2	0.2	0.2	0.1	0.1	0.2	0.2	0.2	0.2	0.2	0.1	0.1	0.1	0.2	0.2	0.2
Opitutae,																							
Opitutaceae (5)	0.0	0.0	0.0	0.0	0.0	0.0	0.0	0.0	0.0	0.0	0.0	0.0	0.0	0.0	0.0	0.0	0.0	0.0	0.0	0.0	0.0	0.0	0.0
Unassigned *Opitutae* (2)	0.0	0.0	0.0	0.0	0.0	0.0	0.0	0.0	0.0	0.0	0.0	0.0	0.0	0.0	0.0	0.0	0.0	0.0	0.0	0.0	0.0	0.0	0.0
Spartobacteria,																							
Chthoniobacteraceae (46)	0.2	0.1	0.1	0.2	0.1	0.1	0.1	0.1	0.1	0.1	0.1	0.1	0.1	0.1	0.1	0.1	0.1	0.0	0.1	0.1	0.1	0.1	0.1
Unassigned *Chthoniobacterales* (2)	0.0	0.0	0.0	0.0	0.0	0.0	0.0	0.0	0.0	0.0	0.0	0.0	0.0	0.0	0.0	0.0	0.0	0.0	0.0	0.0	0.0	0.0	0.0
DA101 soil group (28)	1.0	0.9	0.8	1.0	0.8	1.0	0.9	1.0	1.0	1.0	0.6	0.8	0.9	0.8	0.8	0.9	1.0	0.4	0.6	0.6	0.5	1.0	0.7
Xiphinematobacteraceae (9)	2.3	2.0	1.9	2.0	1.8	2.0	0.8	1.6	1.8	1.5	1.1	1.3	1.2	1.8	1.4	1.8	1.7	1.0	0.9	1.0	1.9	1.9	1.9
Unassigned *Spartobacteria* (7)	0.0	0.0	0.0	0.0	0.0	0.0	0.0	0.0	0.0	0.0	0.0	0.0	0.0	0.0	0.0	0.0	0.0	0.0	0.0	0.0	0.0	0.0	0.0
Verrucomicrobiae,																							
Verrucomicrobiaceae (22)	0.0	0.0	0.0	0.0	0.0	0.0	0.0	0.0	0.0	0.0	0.0	0.0	0.0	0.0	0.0	0.0	0.0	0.0	0.0	0.0	0.0	0.0	0.0
Unassigned *Verrucomicrobia* (1)	0.0	0.0	0.0	0.0	0.0	0.0	0.0	0.0	0.0	0.0	0.0	0.0	0.0	0.0	0.0	0.0	0.0	0.0	0.0	0.0	0.0	0.0	0.0

Sampling Time:	30 h																				
Treatment:	C1	C2	C3	CAA 1	CAA 2	CAA 3	Glu 1	Glu 2	Glu 3	Asp 1	Asp 2	Asp 3	Thr 1	Thr 2	Thr 3	Ala/ Gly1	Ala/ Gly2	Ala/ Gly3	Val/ Gly1	Val/ Gly2	Val/ Gly3
**Phyla, Class, Family[b]**	Relative Abundance %																				
Acidobacteria,																					
Acidobacteria,																					
Acidobacteriaceae (16)	0.1	0.1	0.1	0.0	0.0	0.0	0.0	0.0	0.0	0.0	0.0	0.0	0.0	0.0	0.0	0.1	0.0	0.0	0.0	0.0	0.0
Blastocatellia,																					
Blastocatellaceae (23)	0.1	0.1	0.1	0.0	0.1	0.0	0.0	0.1	0.1	0.1	0.0	0.1	0.0	0.1	0.1	0.1	0.1	0.1	0.1	0.1	0.1
Holophagae,																					
Unassigned *Holophagae* (12)	0.0	0.0	0.0	0.0	0.0	0.0	0.0	0.0	0.0	0.0	0.0	0.0	0.0	0.0	0.0	0.0	0.0	0.0	0.0	0.0	0.0
Solibacteres,																					
Solibacteraceae (34)	0.3	0.3	0.2	0.2	0.2	0.2	0.2	0.2	0.1	0.2	0.2	0.2	0.2	0.2	0.2	0.3	0.2	0.2	0.3	0.2	0.2
Subgroup_11 (5)	0.0	0.0	0.0	0.0	0.0	0.0	0.0	0.0	0.0	0.0	0.0	0.0	0.0	0.0	0.0	0.0	0.0	0.0	0.0	0.0	0.0
Subgroup_17 (16)	0.0	0.0	0.0	0.0	0.0	0.0	0.0	0.0	0.0	0.0	0.0	0.0	0.0	0.0	0.0	0.0	0.0	0.0	0.0	0.0	0.0
Subgroup_22 (16)	0.0	0.0	0.0	0.0	0.0	0.0	0.0	0.0	0.0	0.0	0.0	0.0	0.0	0.0	0.0	0.0	0.0	0.0	0.0	0.0	0.0
Subgroup_25 (9)	0.0	0.0	0.0	0.0	0.0	0.0	0.0	0.0	0.0	0.0	0.0	0.0	0.0	0.0	0.0	0.0	0.0	0.0	0.0	0.0	0.0
Subgroup_5 (10)	0.1	0.1	0.1	0.1	0.1	0.1	0.1	0.1	0.1	0.1	0.1	0.1	0.0	0.1	0.1	0.1	0.1	0.1	0.1	0.1	0.1
Subgroup_6 (113)	0.7	0.6	0.6	0.5	0.5	0.4	0.4	0.5	0.4	0.6	0.4	0.6	0.5	0.5	0.5	0.6	0.6	0.5	0.5	0.5	0.5
Unassigned *Acidobacteria* (8)	0.0	0.0	0.0	0.0	0.0	0.0	0.0	0.0	0.0	0.0	0.0	0.0	0.0	0.0	0.0	0.0	0.0	0.0	0.0	0.0	0.0
Actinobacteria,																					
Acidimicrobiia,																					
Acidimicrobiaceae (30)	1.2	1.0	1.2	0.8	0.8	0.8	0.7	0.8	0.7	0.9	0.8	0.8	0.9	1.0	1.0	1.2	1.2	1.0	1.1	1.0	1.0
Iamiaceae (12)	0.1	0.1	0.2	0.1	0.1	0.1	0.1	0.1	0.1	0.1	0.1	0.1	0.1	0.1	0.1	0.2	0.1	0.1	0.2	0.1	0.1
Unassigned *Acidimicrobiales* (70)	1.2	1.1	1.2	0.9	0.8	0.8	0.7	0.8	0.7	0.9	0.9	0.8	1.0	1.0	1.0	1.1	1.1	1.0	1.3	0.9	1.0
Actinobacteria,																					
Actinospicaceae (1)	0.0	0.0	0.0	0.0	0.0	0.0	0.0	0.0	0.0	0.0	0.0	0.0	0.0	0.0	0.0	0.0	0.0	0.0	0.0	0.0	0.0
Catenulisporaceae (1)	0.0	0.0	0.0	0.0	0.0	0.0	0.0	0.0	0.0	0.0	0.0	0.0	0.0	0.0	0.0	0.0	0.0	0.0	0.0	0.0	0.0

Sampling Time:	30 h																				
Treatment:	C1	C2	C3	CAA 1	CAA 2	CAA 3	Glu 1	Glu 2	Glu 3	Asp 1	Asp 2	Asp 3	Thr 1	Thr 2	Thr 3	Ala/ Gly1	Ala/ Gly2	Ala/ Gly3	Val/ Gly1	Val/ Gly2	Val/ Gly3
**Phyla, Class, Family[b]**	**Relative Abundance %**																				
Mycobacteriaceae (12)	0.5	0.4	0.4	0.3	0.3	0.4	0.3	0.3	0.3	0.3	0.3	0.3	0.4	0.3	0.3	0.3	0.4	0.3	0.4	0.4	0.3
Nocardiaceae (12)	0.1	0.0	0.1	0.0	0.0	0.0	0.0	0.0	0.0	0.0	0.0	0.0	0.0	0.0	0.0	0.0	0.1	0.0	0.0	0.1	0.0
Acidothermaceae (10)	0.1	0.1	0.1	0.1	0.1	0.1	0.1	0.0	0.0	0.1	0.1	0.1	0.1	0.1	0.1	0.1	0.1	0.1	0.1	0.1	0.1
Cryptosporangiaceae (2)	0.0	0.0	0.0	0.0	0.0	0.0	0.0	0.0	0.0	0.0	0.0	0.0	0.0	0.0	0.0	0.0	0.0	0.0	0.0	0.0	0.0
Frankiaceae (5)	0.1	0.1	0.1	0.1	0.1	0.1	0.0	0.0	0.0	0.1	0.1	0.1	0.1	0.1	0.1	0.1	0.1	0.1	0.1	0.1	0.1
Geodermatophilaceae (3)	0.0	0.0	0.0	0.0	0.0	0.0	0.0	0.0	0.0	0.0	0.0	0.0	0.0	0.0	0.0	0.0	0.0	0.0	0.0	0.0	0.0
Nakamurellaceae (5)	0.2	0.2	0.2	0.2	0.2	0.2	0.1	0.1	0.1	0.2	0.1	0.2	0.2	0.1	0.1	0.2	0.2	0.2	0.2	0.2	0.2
Sporichthyaceae (6)	0.1	0.1	0.1	0.1	0.1	0.1	0.1	0.1	0.0	0.1	0.1	0.1	0.1	0.1	0.1	0.1	0.1	0.1	0.1	0.1	0.1
Glycomycetaceae (2)	0.0	0.0	0.0	0.0	0.0	0.0	0.0	0.0	0.0	0.0	0.0	0.0	0.0	0.0	0.0	0.0	0.0	0.0	0.0	0.0	0.0
Kineosporiaceae (5)	0.0	0.0	0.0	0.0	0.0	0.0	0.0	0.0	0.0	0.0	0.0	0.0	0.0	0.0	0.0	0.0	0.0	0.0	0.0	0.0	0.0
Beutenbergiaceae (1)	0.0	0.0	0.0	0.0	0.0	0.0	0.0	0.0	0.0	0.0	0.0	0.0	0.0	0.0	0.0	0.0	0.0	0.0	0.0	0.0	0.0
Brevibacteriaceae (1)	0.0	0.0	0.0	0.0	0.0	0.0	0.0	0.0	0.0	0.0	0.0	0.0	0.0	0.0	0.0	0.0	0.0	0.0	0.0	0.0	0.0
Cellulomonadaceae (1)	0.2	0.2	0.2	0.2	0.1	0.1	0.1	0.1	0.1	0.1	0.2	0.1	0.2	0.2	0.1	0.2	0.2	0.2	0.2	0.2	0.1
Demequinaceae (1)	0.0	0.0	0.0	0.0	0.0	0.0	0.0	0.0	0.0	0.0	0.0	0.0	0.0	0.0	0.0	0.0	0.0	0.0	0.0	0.0	0.0
Dermabacteraceae (1)	0.0	0.0	0.0	0.0	0.0	0.0	0.0	0.0	0.0	0.0	0.0	0.0	0.0	0.0	0.0	0.0	0.0	0.0	0.0	0.0	0.0
Dermacoccaceae (1)	0.0	0.0	0.0	0.0	0.0	0.0	0.0	0.0	0.0	0.0	0.0	0.0	0.0	0.0	0.0	0.0	0.0	0.0	0.0	0.0	0.0
Intrasporangiaceae (4)	0.3	0.2	0.2	0.2	0.2	0.1	0.1	0.2	0.2	0.2	0.2	0.2	0.2	0.2	0.2	0.2	0.2	0.2	0.2	0.2	0.2
Microbacteriaceae (15)	0.1	0.1	0.1	0.1	0.1	0.1	0.1	0.1	0.1	0.1	0.1	0.1	0.1	0.1	0.1	0.1	0.1	0.1	0.1	0.1	0.1
Micrococcaceae (4)	0.2	0.2	0.2	0.2	0.1	0.1	0.1	0.1	0.1	0.1	0.2	0.1	0.1	0.1	0.2	0.2	0.2	0.2	0.2	0.2	0.2
Promicromonosporaceae (1)	0.0	0.0	0.0	0.0	0.0	0.0	0.0	0.0	0.0	0.0	0.0	0.0	0.0	0.0	0.0	0.0	0.0	0.0	0.0	0.0	0.0
Sanguibacteraceae (1)	0.0	0.0	0.0	0.0	0.0	0.0	0.0	0.0	0.0	0.0	0.0	0.0	0.0	0.0	0.0	0.0	0.0	0.0	0.0	0.0	0.0
Micromonosporaceae (44)	1.1	1.0	1.0	0.8	0.7	1.0	0.7	0.7	0.6	0.8	0.7	0.7	0.9	0.9	0.8	0.9	1.0	0.8	0.9	0.9	0.9
Nocardioidaceae (41)	1.2	1.0	1.2	0.8	0.8	0.8	0.7	0.8	0.7	0.9	0.9	0.8	0.8	0.9	1.0	1.1	1.1	1.0	1.2	0.9	1.0
Propionibacteriaceae (11)	0.2	0.2	0.2	0.1	0.1	0.1	0.1	0.1	0.1	0.1	0.1	0.1	0.2	0.1	0.2	0.2	0.2	0.1	0.2	0.2	0.2
Pseudonocardiaceae (20)	0.6	0.5	0.5	0.5	0.4	0.5	0.4	0.3	0.4	0.4	0.4	0.4	0.6	0.4	0.4	0.5	0.5	0.4	0.5	0.5	0.6
Streptomycetaceae (5)	0.4	0.4	0.4	0.3	0.3	0.3	0.3	0.3	0.3	0.3	0.3	0.3	0.3	0.3	0.4	0.4	0.5	0.4	0.5	0.4	0.4
Nocardiopsaceae (1)	0.0	0.0	0.0	0.0	0.0	0.0	0.0	0.0	0.0	0.0	0.0	0.0	0.0	0.0	0.0	0.0	0.0	0.0	0.0	0.0	0.0
Streptosporangiaceae (4)	0.0	0.0	0.0	0.0	0.0	0.0	0.0	0.0	0.0	0.0	0.0	0.0	0.0	0.0	0.0	0.0	0.0	0.0	0.0	0.0	0.0
Thermomonosporaceae (15)	0.0	0.0	0.0	0.0	0.0	0.0	0.0	0.0	0.0	0.0	0.0	0.0	0.0	0.0	0.0	0.0	0.0	0.0	0.0	0.0	0.0
Unassigned *Frankiales* (2)	0.0	0.0	0.0	0.0	0.0	0.0	0.0	0.0	0.0	0.0	0.0	0.0	0.0	0.0	0.0	0.0	0.0	0.0	0.0	0.0	0.0
Unassigned *Actinobacteria* (2)	0.2	0.2	0.2	0.1	0.1	0.1	0.1	0.1	0.1	0.1	0.1	0.1	0.2	0.1	0.2	0.1	0.1	0.1	0.1	0.2	0.2
Coriobacteriia,																					
Coriobacteriaceae (1)	0.0	0.0	0.0	0.0	0.0	0.0	0.0	0.0	0.0	0.0	0.0	0.0	0.0	0.0	0.0	0.0	0.0	0.0	0.0	0.0	0.0
Rubrobacteria,																					
Rubrobacteriaceae (6)	0.1	0.1	0.1	0.1	0.1	0.1	0.1	0.1	0.0	0.1	0.1	0.1	0.1	0.1	0.1	0.1	0.1	0.1	0.1	0.1	0.1
Thermoleophilia,																					
Gaiellaceae (11)	0.7	0.6	0.7	0.5	0.5	0.5	0.5	0.5	0.5	0.7	0.6	0.6	0.6	0.6	0.6	0.7	0.8	0.7	0.8	0.7	0.6
Unassigned *Gaiellales* (72)	1.3	1.1	1.4	0.9	0.8	0.9	0.8	1.0	0.9	1.1	1.0	1.0	1.0	1.0	1.1	1.2	1.3	1.2	1.4	1.1	1.2
Conexibacteraceae (3)	0.0	0.0	0.0	0.0	0.0	0.0	0.0	0.0	0.0	0.0	0.0	0.0	0.0	0.0	0.0	0.0	0.0	0.0	0.0	0.0	0.0
Parviterribacteraceae (3)	0.0	0.0	0.0	0.0	0.0	0.0	0.0	0.0	0.0	0.0	0.0	0.0	0.0	0.0	0.0	0.0	0.0	0.0	0.0	0.0	0.0
Patulibacteraceae (15)	0.0	0.0	0.0	0.0	0.0	0.0	0.0	0.0	0.0	0.0	0.0	0.0	0.0	0.0	0.0	0.0	0.0	0.0	0.0	0.0	0.0
Solirubrobacteraceae (15)	1.0	1.0	1.0	0.7	0.8	0.6	0.6	0.6	0.6	0.8	0.8	0.7	0.7	0.8	0.9	1.0	1.0	0.9	1.1	0.9	0.9
Unassigned *Solirubrobacterales* (26)	0.3	0.3	0.4	0.3	0.2	0.2	0.2	0.3	0.2	0.3	0.3	0.3	0.3	0.3	0.3	0.3	0.3	0.3	0.4	0.3	0.3
Unassigned *Thermoleophilia* (67)	0.5	0.4	0.5	0.3	0.4	0.4	0.3	0.4	0.3	0.4	0.4	0.4	0.4	0.4	0.4	0.5	0.5	0.5	0.5	0.4	0.5
Unassigned *Actinobacteria* (34)	0.4	0.4	0.4	0.3	0.3	0.3	0.2	0.3	0.3	0.3	0.3	0.3	0.3	0.3	0.3	0.4	0.4	0.4	0.4	0.3	0.4
Armatimonadetes,																					
Armatimonadia,																					
Unassigned *Armatimonadales* (3)	0.0	0.0	0.0	0.0	0.0	0.0	0.0	0.0	0.0	0.0	0.0	0.0	0.0	0.0	0.0	0.0	0.0	0.0	0.0	0.0	0.0
Chthonomonadaceae (2)	0.0	0.0	0.0	0.0	0.0	0.0	0.0	0.0	0.0	0.0	0.0	0.0	0.0	0.0	0.0	0.0	0.0	0.0	0.0	0.0	0.0
Unassigned *Chthonomonadales* (2)	0.0	0.0	0.0	0.0	0.0	0.0	0.0	0.0	0.0	0.0	0.0	0.0	0.0	0.0	0.0	0.0	0.0	0.0	0.0	0.0	0.0

Sampling Time:	30 h																				
**Treatment:**	**C1**	**C2**	**C3**	**CAA 1**	**CAA 2**	**CAA 3**	**Glu 1**	**Glu 2**	**Glu 3**	**Asp 1**	**Asp 2**	**Asp 3**	**Thr 1**	**Thr 2**	**Thr 3**	**Ala/ Gly1**	**Ala/ Gly2**	**Ala/ Gly3**	**Val/ Gly1**	**Val/ Gly2**	**Val/ Gly3**
**Phyla, Class, Family[b]**	**Relative Abundance %**																				
Unassigned *Armatimonadetes* (21)	0.0	0.0	0.0	0.0	0.0	0.0	0.0	0.0	0.0	0.0	0.0	0.0	0.0	0.0	0.0	0.0	0.0	0.0	0.0	0.0	0.0
Bacteroidetes,																					
Bacteroidia,																					
Prolixibacteraceae (1)	0.0	0.0	0.0	0.0	0.0	0.0	0.0	0.0	0.0	0.0	0.0	0.0	0.0	0.0	0.0	0.0	0.0	0.0	0.0	0.0	0.0
Cytophagia (2)	0.0	0.0	0.0	0.0	0.0	0.0	0.0	0.0	0.0	0.0	0.0	0.0	0.0	0.0	0.0	0.0	0.0	0.0	0.0	0.0	0.0
Cytophagaceae (33)	0.1	0.1	0.1	0.0	0.0	0.1	0.0	0.0	0.0	0.1	0.1	0.0	0.1	0.1	0.1	0.1	0.1	0.1	0.0	0.1	0.1
Flammeovirgaceae (1)	0.0	0.0	0.0	0.0	0.0	0.0	0.0	0.0	0.0	0.0	0.0	0.0	0.0	0.0	0.0	0.0	0.0	0.0	0.0	0.0	0.0
Flavobacteriia,																					
Flavobacteriaceae (21)	1.5	1.3	1.3	0.7	0.7	0.5	0.6	0.4	0.3	0.5	0.6	0.6	0.6	0.7	0.8	0.1	0.1	0.1	0.2	0.1	0.1
Sphingobacteriia,																					
Chitinophagaceae (48)	0.1	0.1	0.1	0.1	0.1	0.1	0.1	0.1	0.1	0.1	0.1	0.1	0.1	0.1	0.1	0.1	0.1	0.1	0.1	0.1	0.1
Lentimicrobiaceae (1)	0.0	0.0	0.0	0.0	0.0	0.0	0.0	0.0	0.0	0.0	0.0	0.0	0.0	0.0	0.0	0.0	0.0	0.0	0.0	0.0	0.0
Saprospiraceae (2)	0.0	0.0	0.0	0.0	0.0	0.0	0.0	0.0	0.0	0.0	0.0	0.0	0.0	0.0	0.0	0.0	0.0	0.0	0.0	0.0	0.0
Sphingobacteriaceae (3)	0.0	0.0	0.0	0.0	0.0	0.0	0.0	0.0	0.0	0.0	0.0	0.0	0.0	0.0	0.0	0.0	0.0	0.0	0.0	0.0	0.0
Unassigned *Sphingobacteriales* (14)	0.0	0.0	0.0	0.0	0.0	0.0	0.0	0.0	0.0	0.0	0.0	0.0	0.0	0.0	0.0	0.0	0.0	0.0	0.0	0.0	0.0
Unassigned *Sphingobacteriia* (10)	0.0	0.0	0.0	0.0	0.0	0.0	0.0	0.0	0.0	0.0	0.0	0.0	0.0	0.0	0.0	0.0	0.0	0.0	0.0	0.0	0.0
Unassigned *Bacteroidetes* (3)	0.0	0.0	0.0	0.0	0.0	0.0	0.0	0.0	0.0	0.0	0.0	0.0	0.0	0.0	0.0	0.0	0.0	0.0	0.0	0.0	0.0
BJ-169,																					
Unassigned *BJ-169* (6)	0.0	0.0	0.0	0.0	0.0	0.0	0.0	0.0	0.0	0.0	0.0	0.0	0.0	0.0	0.0	0.0	0.0	0.0	0.0	0.0	0.0
BRC1,																					
Unassigned *BCR1* (14)	0.0	0.0	0.0	0.0	0.0	0.0	0.0	0.0	0.0	0.0	0.0	0.0	0.0	0.0	0.0	0.0	0.0	0.0	0.0	0.0	0.0
Chlamydiae,																					
Chlamydiae,																					
Chlamydiaceae (4)	0.0	0.0	0.0	0.0	0.0	0.0	0.0	0.0	0.0	0.0	0.0	0.0	0.0	0.0	0.0	0.0	0.0	0.0	0.0	0.0	0.0
Parachlamydiaceae (154)	0.1	0.0	0.0	0.0	0.0	0.0	0.0	0.0	0.1	0.1	0.0	0.0	0.0	0.1	0.1	0.1	0.1	0.1	0.1	0.1	0.1
Simkaniaceae (11)	0.0	0.0	0.0	0.0	0.0	0.0	0.0	0.0	0.0	0.0	0.0	0.0	0.0	0.0	0.0	0.0	0.0	0.0	0.0	0.0	0.0
Waddliaceae (1)	0.0	0.0	0.0	0.0	0.0	0.0	0.0	0.0	0.0	0.0	0.0	0.0	0.0	0.0	0.0	0.0	0.0	0.0	0.0	0.0	0.0
Unassigned *Chlamydiales* (7)	0.0	0.0	0.0	0.0	0.0	0.0	0.0	0.0	0.0	0.0	0.0	0.0	0.0	0.0	0.0	0.0	0.0	0.0	0.0	0.0	0.0
Chlorobi,																					
Chlorobia,																					
Unassigned *Chlorobia* (4)	0.0	0.0	0.0	0.0	0.0	0.0	0.0	0.0	0.0	0.0	0.0	0.0	0.0	0.0	0.0	0.0	0.0	0.0	0.0	0.0	0.0
Chloroflexi,																					
Anaerolineae,																					
Anaerolineaceae (14)	0.0	0.0	0.0	0.0	0.0	0.0	0.0	0.0	0.0	0.0	0.0	0.0	0.0	0.0	0.0	0.0	0.0	0.0	0.0	0.0	0.0
Unassigned *Ardenticatenia* (3)	0.0	0.0	0.0	0.0	0.0	0.0	0.0	0.0	0.0	0.0	0.0	0.0	0.0	0.0	0.0	0.0	0.0	0.0	0.0	0.0	0.0
Caldilineae,																					
Caldilineaceae (15)	0.1	0.1	0.1	0.1	0.1	0.1	0.0	0.1	0.1	0.1	0.0	0.1	0.1	0.1	0.1	0.1	0.1	0.1	0.1	0.1	0.1
Chloroflexia,																					
Roseiflexaceae (8)	0.2	0.1	0.1	0.1	0.1	0.1	0.1	0.1	0.1	0.1	0.1	0.1	0.1	0.1	0.1	0.1	0.1	0.1	0.1	0.1	0.1
Unassigned *Chloroflexia* (4)	0.0	0.0	0.0	0.0	0.0	0.0	0.0	0.0	0.0	0.0	0.0	0.0	0.0	0.0	0.0	0.0	0.0	0.0	0.0	0.0	0.0
Ktedonobacteria,																					
Ktedonobacterales,																					
Ktedonobacteraceae (9)	0.0	0.0	0.0	0.0	0.0	0.0	0.0	0.0	0.0	0.0	0.0	0.0	0.0	0.0	0.0	0.0	0.0	0.0	0.0	0.0	0.0
Thermosporotrichaceae (7)	0.0	0.0	0.0	0.0	0.0	0.0	0.0	0.0	0.0	0.0	0.0	0.0	0.0	0.0	0.0	0.0	0.0	0.0	0.0	0.0	0.0
Unassigned *Ktedonobacterales* (6)	0.0	0.0	0.0	0.0	0.0	0.0	0.0	0.0	0.0	0.0	0.0	0.0	0.0	0.0	0.0	0.0	0.0	0.0	0.0	0.0	0.0
Unassigned *Ktedonobacteria* (18)	0.1	0.1	0.1	0.1	0.1	0.0	0.0	0.1	0.0	0.1	0.1	0.1	0.1	0.1	0.1	0.1	0.1	0.0	0.1	0.1	0.1
Thermomicrobia,																					
Thermomicrobiaceae (1)	0.0	0.0	0.0	0.0	0.0	0.0	0.0	0.0	0.0	0.0	0.0	0.0	0.0	0.0	0.0	0.0	0.0	0.0	0.0	0.0	0.0

Sampling Time:	30 h																				
Treatment:	C1	C2	C3	CAA 1	CAA 2	CAA 3	Glu 1	Glu 2	Glu 3	Asp 1	Asp 2	Asp 3	Thr 1	Thr 2	Thr 3	Ala/ Gly1	Ala/ Gly2	Ala/ Gly3	Val/ Gly1	Val/ Gly2	Val/ Gly3
**Phyla, Class, Family[b]**	**Relative Abundance %**																				
Unassigned *Thermomicrobia* (56)	0.2	0.2	0.2	0.1	0.1	0.1	0.1	0.1	0.1	0.2	0.2	0.2	0.2	0.2	0.2	0.2	0.2	0.2	0.2	0.2	0.2
Unassigned *Chloroflexi* (111)	1.1	1.0	1.1	0.8	0.8	0.7	0.6	0.8	0.7	0.9	0.7	0.8	0.8	0.9	0.9	1.0	1.0	0.9	1.1	0.9	0.9
Cyanobacteria,																					
Chloroplast,																					
Unassigned *Chloroplast* (34)	0.0	0.0	0.0	0.0	0.0	0.0	0.0	0.0	0.0	0.0	0.0	0.0	0.0	0.0	0.0	0.0	0.0	0.0	0.0	0.0	0.0
Cyanobacteria,																					
Unassigned *Cyanobacteria* (2)	0.0	0.0	0.0	0.0	0.0	0.0	0.0	0.0	0.0	0.0	0.0	0.0	0.0	0.0	0.0	0.0	0.0	0.0	0.0	0.0	0.0
Gastranaerophilales,																					
Unassigned *Gastranaerophilales* (1)	0.0	0.0	0.0	0.0	0.0	0.0	0.0	0.0	0.0	0.0	0.0	0.0	0.0	0.0	0.0	0.0	0.0	0.0	0.0	0.0	0.0
Obscuribacterales,																					
Unassigned *Obscuribacterales* (6)	0.0	0.0	0.0	0.0	0.0	0.0	0.0	0.0	0.0	0.0	0.0	0.0	0.0	0.0	0.0	0.0	0.0	0.0	0.0	0.0	0.0
Unassigned *Cyanobacteria* (11)	0.0	0.0	0.0	0.0	0.0	0.0	0.0	0.0	0.0	0.0	0.0	0.0	0.0	0.0	0.0	0.0	0.0	0.0	0.0	0.0	0.0
Deferribacteres,																					
Unassigned *Deferribacteres* (1)	0.0	0.0	0.0	0.0	0.0	0.0	0.0	0.0	0.0	0.0	0.0	0.0	0.0	0.0	0.0	0.0	0.0	0.0	0.0	0.0	0.0
Deinococcus-Thermus,																					
Deinococci,																					
Unassigned *Deinococci* (4)	0.0	0.0	0.0	0.0	0.0	0.0	0.0	0.0	0.0	0.0	0.0	0.0	0.0	0.0	0.0	0.0	0.0	0.0	0.0	0.0	0.0
Elusimicrobia,																					
Unassigned *Elusimicrobia,*	0.0	0.0	0.0	0.0	0.0	0.0	0.0	0.0	0.0	0.0	0.0	0.0	0.0	0.0	0.0	0.0	0.0	0.0	0.0	0.0	0.0
Euryarchaeota,																					
Methanomicrobia,																					
Methanosarcinaceae (1)	0.0	0.0	0.0	0.0	0.0	0.0	0.0	0.0	0.0	0.0	0.0	0.0	0.0	0.0	0.0	0.0	0.0	0.0	0.0	0.0	0.0
Thermoplasmata,																					
Unassinged Thermoplasmatales (1)	0.0	0.0	0.0	0.0	0.0	0.0	0.0	0.0	0.0	0.0	0.0	0.0	0.0	0.0	0.0	0.0	0.0	0.0	0.0	0.0	0.0
Fibrobacteres,																					
Fibrobacteria,																					
Fibrobacteraceae (5)	0.0	0.0	0.0	0.0	0.0	0.0	0.0	0.0	0.0	0.0	0.0	0.0	0.0	0.0	0.0	0.0	0.0	0.0	0.0	0.0	0.0
Unassigned *Fibrobacterales* (3)	0.0	0.0	0.0	0.0	0.0	0.0	0.0	0.0	0.0	0.0	0.0	0.0	0.0	0.0	0.0	0.0	0.0	0.0	0.0	0.0	0.0
Firmicutes,																					
Bacilli,																					
Alicyclobacillaceae (5)	0.0	0.0	0.0	0.0	0.0	0.0	0.0	0.0	0.0	0.0	0.0	0.0	0.0	0.0	0.0	0.0	0.0	0.0	0.0	0.0	0.0
Bacillaceae (38)	0.6	0.6	0.6	0.6	0.6	0.6	0.4	0.5	0.5	0.5	0.4	0.5	1.0	0.8	1.0	0.7	0.9	0.9	1.0	0.9	0.9
Paenibacillaceae (88)	0.2	0.2	0.3	0.2	0.2	0.3	0.1	0.2	0.1	0.4	0.3	0.3	0.2	0.2	0.2	0.3	0.3	0.4	0.2	0.4	0.4
Pasteuriaceae (6)	0.0	0.0	0.0	0.0	0.0	0.0	0.0	0.0	0.0	0.0	0.0	0.0	0.0	0.0	0.0	0.0	0.0	0.0	0.0	0.0	0.0
Planococcaceae (7)	0.0	0.0	0.0	0.0	0.0	0.0	0.0	0.0	0.0	0.0	0.0	0.0	0.0	0.0	0.0	0.1	0.0	0.0	0.0	0.0	0.0
Sporolactobacillaceae (1)	0.0	0.0	0.0	0.0	0.0	0.0	0.0	0.0	0.0	0.0	0.0	0.0	0.0	0.0	0.0	0.0	0.0	0.0	0.0	0.0	0.0
Staphylococcaceae (1)	0.0	0.0	0.0	0.0	0.0	0.0	0.0	0.0	0.0	0.0	0.0	0.0	0.0	0.0	0.0	0.0	0.0	0.0	0.0	0.0	0.0
Thermoactinomycetaceae (18)	0.0	0.0	0.0	0.0	0.0	0.0	0.0	0.0	0.0	0.0	0.0	0.0	0.0	0.0	0.0	0.0	0.0	0.0	0.0	0.0	0.0
Streptococcaceae (1)	0.0	0.0	0.0	0.0	0.0	0.0	0.0	0.0	0.0	0.0	0.0	0.0	0.0	0.0	0.0	0.0	0.0	0.0	0.0	0.0	0.0
Unassigned *Bacilli* (13)	0.3	0.3	0.3	0.3	0.4	0.2	0.2	0.2	0.2	0.3	0.3	0.3	0.2	0.3	0.2	0.2	0.3	0.2	0.6	0.2	0.2
Clostridia,																					
Caldicoprobacteraceae (7)	0.0	0.0	0.0	0.0	0.0	0.0	0.0	0.0	0.0	0.0	0.0	0.0	0.0	0.0	0.0	0.0	0.0	0.0	0.0	0.0	0.0
Christensenellaceae (7)	0.0	0.0	0.0	0.0	0.0	0.0	0.0	0.0	0.0	0.0	0.0	0.0	0.0	0.0	0.0	0.0	0.0	0.0	0.0	0.0	0.0
Clostridiaceae (38) **[A14]**	2.6	3.2	2.9	2.7	2.2	2.9	8.7	6.8	7.8	3.2	3.4	3.2	3.4	3.6	3.7	2.8	2.4	3.5	1.9	3.0	2.3
Defluviitaleaceae (4)	0.0	0.0	0.0	0.0	0.0	0.0	0.0	0.0	0.0	0.0	0.0	0.0	0.0	0.0	0.0	0.0	0.0	0.0	0.0	0.0	0.0
Eubacteriaceae (4)	0.0	0.0	0.0	0.0	0.0	0.0	0.0	0.0	0.0	0.0	0.0	0.0	0.0	0.0	0.0	0.0	0.0	0.0	0.0	0.0	0.0
Gracilibacteraceae (7)	0.0	0.0	0.0	0.0	0.0	0.0	0.0	0.0	0.0	0.0	0.0	0.0	0.0	0.0	0.0	0.0	0.0	0.0	0.0	0.0	0.0
Heliobacteriaceae (22)	0.0	0.0	0.0	0.0	0.0	0.0	0.0	0.0	0.0	0.0	0.0	0.0	0.0	0.0	0.0	0.0	0.0	0.0	0.0	0.0	0.0

Sampling Time:	30 h																				
**Treatment:**	**C1**	**C2**	**C3**	**CAA 1**	**CAA 2**	**CAA 3**	**Glu 1**	**Glu 2**	**Glu 3**	**Asp 1**	**Asp 2**	**Asp 3**	**Thr 1**	**Thr 2**	**Thr 3**	**Ala/ Gly1**	**Ala/ Gly2**	**Ala/ Gly3**	**Val/ Gly1**	**Val/ Gly2**	**Val/ Gly3**
**Phyla, Class, Family[b]**	**Relative Abundance %**																				
Lachnospiraceae (79)	1.5	2.0	2.2	2.4	2.0	2.4	0.9	1.0	1.2	2.2	2.6	2.0	1.4	1.4	1.2	3.8	3.1	4.3	3.3	4.4	3.9
Peptococcaceae (17)	1.7	1.9	2.1	1.2	1.7	1.5	1.2	1.5	1.5	1.7	1.6	1.5	1.2	1.4	1.2	1.9	1.9	1.9	2.0	1.8	1.8
Peptostreptococcaceae (11) **[GPT-4], [A8]**	1.0	1.2	1.1	3.6	3.8	4.2	1.0	1.2	1.3	0.3	0.3	0.3	6.9	5.9	6.0	12.0	11.9	11.9	6.0	4.1	4.7
Ruminococcaceae (96)	0.2	0.1	0.2	0.1	0.1	0.1	0.0	0.0	0.0	0.1	0.1	0.1	0.1	0.1	0.1	0.2	0.1	0.2	0.1	0.1	0.1
Syntrophomonadaceae (10)	0.0	0.0	0.0	0.0	0.0	0.0	0.0	0.0	0.0	0.0	0.0	0.0	0.0	0.0	0.0	0.0	0.0	0.0	0.0	0.0	0.0
Thermoanaerobacteraceae (8)	0.0	0.0	0.0	0.0	0.0	0.0	0.0	0.0	0.0	0.0	0.0	0.0	0.0	0.0	0.0	0.0	0.0	0.0	0.0	0.0	0.0
Unassigned *Clostridiales* (17)	0.2	0.2	0.2	0.4	0.3	0.4	0.0	0.0	0.0	0.1	0.2	0.1	0.0	0.0	0.0	0.1	0.1	0.1	0.1	0.1	0.1
Unassigned *Thermoanaerobacterales* (1)	0.0	0.0	0.0	0.0	0.0	0.0	0.0	0.0	0.0	0.0	0.0	0.0	0.0	0.0	0.0	0.0	0.0	0.0	0.0	0.0	0.0
Unassigned *Clostridia* (34)	0.0	0.0	0.0	0.0	0.0	0.0	0.0	0.0	0.0	0.0	0.0	0.0	0.0	0.0	0.0	0.0	0.0	0.0	0.0	0.0	0.0
Erysipelotrichia,																					
Erysipelotrichaceae (18)	0.0	0.0	0.0	0.0	0.0	0.0	0.0	0.0	0.0	0.0	0.0	0.0	0.0	0.0	0.0	0.0	0.0	0.0	0.0	0.0	0.0
Limnochordia,																					
Limnochordaceae (23)	0.0	0.0	0.0	0.0	0.0	0.0	0.0	0.0	0.0	0.0	0.0	0.0	0.0	0.0	0.0	0.0	0.0	0.0	0.0	0.0	0.0
Unassigned *Limnochordales* (3)	0.0	0.0	0.0	0.0	0.0	0.0	0.0	0.0	0.0	0.0	0.0	0.0	0.0	0.0	0.0	0.0	0.0	0.0	0.0	0.0	0.0
Negativicutes,																					
Veillonellaceae (20)	0.0	0.0	0.0	0.0	0.0	0.0	0.0	0.0	0.0	0.0	0.0	0.0	0.0	0.0	0.0	0.0	0.0	0.0	0.0	0.0	0.0
Unassigned *Selenomonadales* (3)	0.0	0.0	0.0	0.0	0.0	0.0	0.0	0.0	0.0	0.0	0.0	0.0	0.0	0.0	0.0	0.0	0.0	0.0	0.0	0.0	0.0
Unassigned *Firmicutes* (1)	0.0	0.0	0.0	0.0	0.0	0.0	0.0	0.0	0.0	0.0	0.0	0.0	0.0	0.0	0.0	0.0	0.0	0.0	0.0	0.0	0.0
Fusobacteria,																					
Fusobacteriia,																					
Fusobacteriaceae (10) **[GPT-5]**	16	16	16	25	25	26	29	28	30	21	21	22	19	17	17	9.4	9.8	12	16	19	18
Leptotrichiaceae (1)	0.0	0.0	0.0	0.0	0.0	0.0	0.0	0.0	0.0	0.0	0.0	0.0	0.0	0.0	0.0	0.0	0.0	0.0	0.0	0.0	0.0
Gemmatimonadetes,																					
Gemmatimonadetes,																					
Gemmatimonadaceae (37)	0.2	0.2	0.2	0.1	0.2	0.1	0.1	0.2	0.1	0.2	0.1	0.1	0.2	0.2	0.2	0.2	0.2	0.2	0.2	0.2	0.1
Longimicrobiaceae (1)	0.0	0.0	0.0	0.0	0.0	0.0	0.0	0.0	0.0	0.0	0.0	0.0	0.0	0.0	0.0	0.0	0.0	0.0	0.0	0.0	0.0
Unassigned *Gemmatimonadetes* (6)	0.0	0.0	0.0	0.0	0.0	0.0	0.0	0.0	0.0	0.0	0.0	0.0	0.0	0.0	0.0	0.0	0.0	0.0	0.0	0.0	0.0
Hydrogenedentes,																					
Unassigned *Hydrogenedentes* (2)	0.0	0.0	0.0	0.0	0.0	0.0	0.0	0.0	0.0	0.0	0.0	0.0	0.0	0.0	0.0	0.0	0.0	0.0	0.0	0.0	0.0
Latescibacteria,																					
Unassigned *Latescibacteria* (30)	0.0	0.0	0.0	0.0	0.0	0.0	0.0	0.0	0.0	0.0	0.0	0.0	0.0	0.0	0.0	0.0	0.0	0.0	0.0	0.0	0.0
Nitrospirae,																					
Nitrospira,																					
Nitrospiraceae (8)	0.1	0.1	0.1	0.1	0.1	0.1	0.1	0.1	0.1	0.1	0.1	0.1	0.1	0.1	0.1	0.2	0.2	0.1	0.2	0.1	0.1
Unassigned *Nitrospira* (18)	0.4	0.4	0.5	0.4	0.3	0.3	0.3	0.3	0.2	0.3	0.3	0.3	0.4	0.4	0.4	0.4	0.4	0.4	0.4	0.5	0.4
Parcubacteria,																					
Unassigned *Parcubacteria* (1)	0.0	0.0	0.0	0.0	0.0	0.0	0.0	0.0	0.0	0.0	0.0	0.0	0.0	0.0	0.0	0.0	0.0	0.0	0.0	0.0	0.0
Planctomycetes,																					
Phycisphaerae,																					
Phycisphaeraceae (38)	0.0	0.0	0.0	0.0	0.0	0.0	0.0	0.0	0.0	0.0	0.0	0.0	0.0	0.0	0.0	0.0	0.0	0.0	0.0	0.0	0.0
Tepidisphaeraceae (82)	0.6	0.5	0.4	0.3	0.4	0.3	0.3	0.4	0.4	0.5	0.4	0.4	0.4	0.4	0.5	0.5	0.4	0.4	0.4	0.3	0.4
Unassigned *Phycisphaerales* (4)	0.0	0.0	0.0	0.0	0.0	0.0	0.0	0.0	0.0	0.0	0.0	0.0	0.0	0.0	0.0	0.0	0.0	0.0	0.0	0.0	0.0
Unassigned *Phycisphaerae* (7)	0.0	0.0	0.0	0.0	0.0	0.0	0.0	0.0	0.0	0.0	0.0	0.0	0.0	0.0	0.0	0.0	0.0	0.0	0.0	0.0	0.0
Planctomycetacia,																					
Planctomycetaceae (733)	8.9	7.7	7.8	6.1	6.9	5.7	5.6	6.7	6.2	7.2	6.2	6.3	7.7	8.1	8.6	8.9	8.3	8.2	8.1	6.9	8.6
Unassigned *Planctomycetes* (82)	0.1	0.0	0.0	0.0	0.0	0.0	0.0	0.0	0.0	0.1	0.0	0.0	0.0	0.0	0.0	0.1	0.0	0.1	0.0	0.0	0.0
Proteobacteria,																					

Sampling Time:	30 h																				
Treatment:	C1	C2	C3	CAA 1	CAA 2	CAA 3	Glu 1	Glu 2	Glu 3	Asp 1	Asp 2	Asp 3	Thr 1	Thr 2	Thr 3	Ala/ Gly1	Ala/ Gly2	Ala/ Gly3	Val/ Gly1	Val/ Gly2	Val/ Gly3
Phyla, Class, Family[b]	Relative Abundance %																				
Alphaproteobacteria,																					
Caulobacteraceae (5)	0.0	0.0	0.0	0.0	0.0	0.0	0.0	0.0	0.0	0.0	0.0	0.0	0.0	0.0	0.0	0.0	0.0	0.0	0.0	0.0	0.0
Beijerinckiaceae (2)	0.0	0.0	0.0	0.0	0.0	0.0	0.0	0.0	0.0	0.0	0.0	0.0	0.0	0.0	0.0	0.0	0.0	0.0	0.0	0.0	0.0
Bradyrhizobiaceae (5)	1.6	1.3	1.1	1.2	0.9	1.0	1.0	0.8	0.8	1.0	0.9	0.9	1.2	1.1	1.1	1.3	1.3	1.1	1.2	1.3	1.4
Hyphomicrobiaceae (8)	0.2	0.2	0.2	0.2	0.2	0.2	0.1	0.1	0.1	0.1	0.1	0.1	0.1	0.2	0.1	0.2	0.2	0.2	0.2	0.2	0.2
Methylobacteriaceae (4)	0.2	0.1	0.1	0.1	0.1	0.1	0.1	0.1	0.1	0.1	0.1	0.1	0.1	0.1	0.1	0.1	0.2	0.1	0.1	0.1	0.1
Methylocystaceae (1)	0.0	0.0	0.0	0.0	0.0	0.0	0.0	0.0	0.0	0.0	0.0	0.0	0.0	0.0	0.0	0.0	0.0	0.0	0.0	0.0	0.0
Phyllobacteriaceae (3)	0.2	0.2	0.2	0.2	0.2	0.2	0.1	0.1	0.1	0.2	0.1	0.1	0.1	0.2	0.1	0.2	0.2	0.2	0.2	0.2	0.2
Rhizobiaceae (3)	0.0	0.0	0.0	0.0	0.0	0.0	0.0	0.0	0.0	0.0	0.0	0.0	0.0	0.0	0.0	0.0	0.0	0.0	0.0	0.0	0.0
Rhodobiaceae (2)	0.3	0.3	0.3	0.2	0.2	0.2	0.2	0.2	0.2	0.2	0.2	0.2	0.2	0.2	0.2	0.3	0.3	0.3	0.3	0.3	0.2
Roseiarcaceae (1)	0.0	0.0	0.0	0.0	0.0	0.0	0.0	0.0	0.0	0.0	0.0	0.0	0.0	0.0	0.0	0.0	0.0	0.0	0.0	0.0	0.0
Xanthobacteraceae (9)	1.1	1.0	1.0	0.8	0.9	0.8	0.7	0.8	0.8	1.0	0.8	0.8	0.9	0.8	0.9	1.0	1.1	1.0	1.1	1.0	0.9
Unassigned *Rhizobiales* (19)	0.2	0.2	0.2	0.1	0.2	0.2	0.1	0.1	0.1	0.1	0.2	0.2	0.2	0.2	0.2	0.2	0.2	0.2	0.2	0.2	0.2
Rhodobacteraceae (6)	0.1	0.0	0.0	0.0	0.0	0.0	0.0	0.0	0.0	0.0	0.0	0.0	0.0	0.0	0.0	0.0	0.1	0.0	0.1	0.1	0.0
Acetobacteraceae (15)	0.2	0.2	0.2	0.2	0.1	0.2	0.1	0.1	0.1	0.1	0.1	0.1	0.2	0.1	0.2	0.2	0.2	0.2	0.2	0.2	0.2
Rhodospirillaceae (19)	0.5	0.4	0.4	0.4	0.3	0.3	0.3	0.3	0.3	0.3	0.3	0.3	0.5	0.3	0.3	0.4	0.4	0.4	0.5	0.4	0.5
Unassigned *Rhodospirillales* (25)	0.5	0.5	0.5	0.4	0.4	0.3	0.3	0.3	0.3	0.3	0.3	0.3	0.4	0.4	0.4	0.5	0.5	0.4	0.5	0.4	0.5
Anaplasmataceae (1)	0.0	0.0	0.0	0.0	0.0	0.0	0.0	0.0	0.0	0.0	0.0	0.0	0.0	0.0	0.0	0.0	0.0	0.0	0.0	0.0	0.0
Holosporaceae (2)	0.0	0.0	0.0	0.0	0.0	0.0	0.0	0.0	0.0	0.0	0.0	0.0	0.0	0.0	0.0	0.0	0.0	0.0	0.0	0.0	0.0
Mitochondria (2)	0.0	0.0	0.0	0.0	0.0	0.0	0.0	0.0	0.0	0.0	0.0	0.0	0.0	0.0	0.0	0.0	0.0	0.0	0.0	0.0	0.0
Rickettsiaceae (1)	0.0	0.0	0.0	0.0	0.0	0.0	0.0	0.0	0.0	0.0	0.0	0.0	0.0	0.0	0.0	0.0	0.0	0.0	0.0	0.0	0.0
Unassigned *Rickettsiales* (7)	0.0	0.0	0.0	0.0	0.0	0.0	0.0	0.0	0.0	0.0	0.0	0.0	0.0	0.0	0.0	0.0	0.0	0.0	0.0	0.0	0.0
Erythrobacteraceae (1)	0.0	0.0	0.0	0.0	0.0	0.0	0.0	0.0	0.0	0.0	0.0	0.0	0.0	0.0	0.0	0.0	0.0	0.0	0.0	0.0	0.0
Sphingomonadaceae (5)	0.0	0.0	0.0	0.0	0.0	0.0	0.0	0.0	0.0	0.0	0.0	0.0	0.0	0.0	0.0	0.0	0.0	0.0	0.0	0.0	0.0
Unassigned *Sphingomonadales* (2)	0.0	0.0	0.0	0.0	0.0	0.0	0.0	0.0	0.0	0.0	0.0	0.0	0.0	0.0	0.0	0.0	0.0	0.0	0.0	0.0	0.0
Unassigned *Alphaproteobacteria* (19)	0.2	0.1	0.1	0.1	0.1	0.1	0.1	0.1	0.1	0.1	0.1	0.1	0.1	0.1	0.1	0.1	0.2	0.1	0.1	0.1	0.1
Betaproteobacteria,																					
Alcaligenaceae (2)	0.1	0.1	0.1	0.0	0.0	0.0	0.0	0.0	0.0	0.0	0.0	0.0	0.1	0.0	0.0	0.1	0.1	0.1	0.1	0.0	0.0
Burkholderiaceae (10)	0.0	0.0	0.0	0.0	0.0	0.0	0.0	0.0	0.0	0.0	0.0	0.0	0.0	0.0	0.0	0.0	0.0	0.0	0.0	0.0	0.0
Comamonadaceae (17)	0.2	0.2	0.2	0.1	0.1	0.1	0.1	0.1	0.1	0.1	0.1	0.1	0.1	0.2	0.1	0.2	0.2	0.1	0.1	0.1	0.1
Oxalobacteraceae (15)	0.0	0.0	0.0	0.0	0.0	0.0	0.0	0.0	0.0	0.0	0.0	0.0	0.0	0.0	0.0	0.0	0.0	0.0	0.0	0.0	0.0
Neisseriaceae (1)	0.0	0.0	0.0	0.0	0.0	0.0	0.0	0.0	0.0	0.0	0.0	0.0	0.0	0.0	0.0	0.0	0.0	0.0	0.0	0.0	0.0
Gallionellaceae (3)	0.0	0.0	0.0	0.0	0.0	0.0	0.0	0.0	0.0	0.0	0.0	0.0	0.0	0.0	0.0	0.0	0.0	0.0	0.0	0.0	0.0
Nitrosomonadaceae (30)	0.2	0.1	0.1	0.1	0.1	0.1	0.1	0.1	0.1	0.1	0.1	0.1	0.1	0.1	0.1	0.1	0.1	0.1	0.2	0.1	0.1
Rhodocyclaceae (9)	0.0	0.0	0.0	0.0	0.0	0.0	0.0	0.0	0.0	0.0	0.0	0.0	0.0	0.0	0.0	0.0	0.0	0.0	0.0	0.0	0.0
Unassigned *Betaproteobacteria* (41)	0.2	0.1	0.1	0.1	0.1	0.1	0.1	0.1	0.1	0.1	0.1	0.1	0.1	0.1	0.1	0.2	0.2	0.1	0.1	0.1	0.1
Deltaproteobacteria,																					
Bacteriovoracaceae (12)	0.0	0.0	0.0	0.0	0.0	0.0	0.0	0.0	0.0	0.0	0.0	0.0	0.0	0.0	0.0	0.0	0.0	0.0	0.0	0.0	0.0
Bdellovibrionaceae (54)	0.1	0.1	0.1	0.1	0.1	0.1	0.0	0.1	0.0	0.1	0.0	0.0	0.1	0.1	0.1	0.1	0.1	0.1	0.1	0.1	0.1
Desulfarculaceae (1)	0.0	0.0	0.0	0.0	0.0	0.0	0.0	0.0	0.0	0.0	0.0	0.0	0.0	0.0	0.0	0.0	0.0	0.0	0.0	0.0	0.0
Desulfobulbaceae (3)	0.0	0.0	0.0	0.0	0.0	0.0	0.0	0.0	0.0	0.0	0.0	0.0	0.0	0.0	0.0	0.0	0.0	0.0	0.0	0.0	0.0
Desulfurellaceae (41)	0.5	0.5	0.5	0.4	0.4	0.4	0.4	0.3	0.3	0.4	0.3	0.4	0.4	0.4	0.4	0.5	0.5	0.5	0.5	0.5	0.5
Desulfuromonadaceae (2)	0.0	0.0	0.0	0.0	0.0	0.0	0.0	0.0	0.0	0.0	0.0	0.0	0.0	0.0	0.0	0.0	0.0	0.0	0.0	0.0	0.0
Geobacteraceae (26)	0.1	0.1	0.1	0.1	0.1	0.1	0.1	0.0	0.1	0.1	0.1	0.1	0.1	0.1	0.1	0.1	0.1	0.1	0.1	0.1	0.1
Archangiaceae (20)	0.1	0.1	0.1	0.0	0.1	0.0	0.0	0.1	0.0	0.1	0.1	0.1	0.1	0.0	0.1	0.1	0.1	0.1	0.1	0.1	0.1
Haliangiaceae (74)	0.4	0.3	0.3	0.2	0.3	0.3	0.2	0.3	0.3	0.4	0.2	0.3	0.2	0.2	0.2	0.4	0.4	0.4	0.4	0.3	0.3
Myxococcaceae (3)	0.0	0.0	0.0	0.0	0.0	0.0	0.0	0.0	0.0	0.0	0.0	0.0	0.0	0.0	0.0	0.0	0.0	0.0	0.0	0.0	0.0
Nannocystaceae (6)	0.0	0.0	0.0	0.0	0.0	0.0	0.0	0.0	0.0	0.0	0.0	0.0	0.0	0.0	0.0	0.0	0.0	0.0	0.0	0.0	0.0
Phaselicystidaceae (14)	0.1	0.1	0.1	0.1	0.1	0.1	0.1	0.1	0.1	0.1	0.1	0.1	0.1	0.1	0.1	0.1	0.1	0.1	0.1	0.1	0.1
Polyangiaceae (48)	0.3	0.3	0.3	0.2	0.2	0.2	0.2	0.2	0.2	0.2	0.2	0.2	0.3	0.3	0.2	0.3	0.3	0.3	0.3	0.2	0.3
Sandaracinaceae (38)	0.2	0.1	0.1	0.1	0.1	0.1	0.1	0.1	0.1	0.1	0.1	0.1	0.1	0.1	0.1	0.1	0.1	0.1	0.2	0.1	0.1

Sampling Time:	30 h																				
Treatment:	C1	C2	C3	CAA 1	CAA 2	CAA 3	Glu 1	Glu 2	Glu 3	Asp 1	Asp 2	Asp 3	Thr 1	Thr 2	Thr 3	Ala/ Gly1	Ala/ Gly2	Ala/ Gly3	Val/ Gly1	Val/ Gly2	Val/ Gly3
**Phyla, Class, Family[b]**	**Relative Abundance %**																				
Vulgatibacteraceae (3)	0.0	0.0	0.0	0.0	0.0	0.0	0.0	0.0	0.0	0.0	0.0	0.0	0.0	0.0	0.0	0.0	0.0	0.0	0.0	0.0	0.0
Unassigned *Myxococcales* (177)	0.3	0.3	0.2	0.2	0.2	0.2	0.2	0.2	0.2	0.3	0.2	0.2	0.2	0.2	0.2	0.3	0.3	0.3	0.3	0.2	0.3
Oligoflexaceae (29)	0.0	0.0	0.0	0.0	0.0	0.0	0.0	0.0	0.0	0.0	0.0	0.0	0.0	0.0	0.0	0.0	0.0	0.0	0.0	0.0	0.0
Unassigned *Oligoflexales* (86)	0.0	0.0	0.0	0.0	0.0	0.0	0.0	0.0	0.0	0.0	0.0	0.0	0.0	0.0	0.0	0.0	0.0	0.0	0.0	0.0	0.0
Syntrophaceae (1)	0.0	0.0	0.0	0.0	0.0	0.0	0.0	0.0	0.0	0.0	0.0	0.0	0.0	0.0	0.0	0.0	0.0	0.0	0.0	0.0	0.0
Unassigned *Deltaproteobacteria* (19)	0.0	0.0	0.0	0.0	0.0	0.0	0.0	0.0	0.0	0.0	0.0	0.0	0.0	0.0	0.0	0.0	0.0	0.0	0.0	0.0	0.0
Gammaproteobacteria,																					
Acidiferrobacteraceae (3)	0.0	0.0	0.0	0.0	0.0	0.0	0.0	0.0	0.0	0.0	0.0	0.0	0.0	0.0	0.0	0.0	0.0	0.0	0.0	0.0	0.0
Aeromonadaceae (8) **[GPT-1]**	13	16	14	12	10	11	8.1	7.3	7.9	14	16	15	14	11	12	7.1	7.0	8.1	7.1	9.2	8.1
Shewanellaceae (3)	3.0	3.5	3.4	3.3	3.8	3.0	3.4	3.4	3.3	2.6	3.1	3.4	4.5	4.2	4.3	2.0	2.2	2.7	3.4	3.4	3.4
Cellvibrionaceae (2)	0.0	0.0	0.0	0.0	0.0	0.0	0.0	0.0	0.0	0.0	0.0	0.0	0.0	0.0	0.0	0.0	0.0	0.0	0.0	0.0	0.0
Halieaceae (4)	0.0	0.0	0.0	0.0	0.0	0.0	0.0	0.0	0.0	0.0	0.0	0.0	0.0	0.0	0.0	0.0	0.0	0.0	0.0	0.0	0.0
Enterobacteriaceae (12) **[GPT-2], [GPT-3]**	2.8	3.2	3.1	5.4	4.9	6.0	12	10	9.1	7.6	8.5	8.1	4.3	4.4	4.1	3.8	4.3	4.6	4.6	4.9	5.2
Coxiellaceae (35)	0.0	0.0	0.0	0.0	0.0	0.0	0.0	0.0	0.0	0.0	0.0	0.0	0.0	0.0	0.0	0.0	0.0	0.0	0.0	0.0	0.0
Legionellaceae (30)	0.0	0.0	0.0	0.0	0.0	0.0	0.0	0.0	0.0	0.0	0.0	0.0	0.0	0.0	0.0	0.0	0.0	0.0	0.0	0.0	0.0
Methylococcaceae (3)	0.0	0.0	0.0	0.0	0.0	0.0	0.0	0.0	0.0	0.0	0.0	0.0	0.0	0.0	0.0	0.0	0.0	0.0	0.0	0.0	0.0
Oleiphilaceae (1)	0.0	0.0	0.0	0.0	0.0	0.0	0.0	0.0	0.0	0.0	0.0	0.0	0.0	0.0	0.0	0.0	0.0	0.0	0.0	0.0	0.0
Moraxellaceae (2)	0.0	0.0	0.0	0.0	0.0	0.0	0.0	0.0	0.0	0.0	0.0	0.0	0.0	0.0	0.0	0.0	0.0	0.0	0.0	0.0	0.0
Pseudomonadaceae (4)	0.9	0.3	0.7	0.1	0.1	0.1	0.1	0.1	0.1	0.1	0.2	0.3	0.2	0.2	0.1	0.2	0.7	0.5	0.4	0.7	0.2
Unassigned Thiotrichales (1)	0.0	0.0	0.0	0.0	0.0	0.0	0.0	0.0	0.0	0.0	0.0	0.0	0.0	0.0	0.0	0.0	0.0	0.0	0.0	0.0	0.0
Xanthomonadaceae (12)	0.0	0.0	0.0	0.0	0.0	0.0	0.0	0.0	0.0	0.0	0.0	0.0	0.0	0.0	0.0	0.0	0.0	0.0	0.0	0.0	0.0
Unassigned *Xanthomonadales* (24)	0.2	0.1	0.1	0.1	0.1	0.1	0.1	0.1	0.1	0.1	0.1	0.1	0.1	0.1	0.1	0.1	0.2	0.1	0.1	0.1	0.1
Unassigned *Gammaproteobacteria* (30)	0.0	0.0	0.0	0.0	0.0	0.0	0.0	0.0	0.0	0.0	0.0	0.0	0.0	0.0	0.0	0.0	0.0	0.0	0.0	0.0	0.0
Unassigned *Proteobacteria* (2)	0.0	0.0	0.0	0.0	0.0	0.0	0.0	0.0	0.0	0.0	0.0	0.0	0.0	0.0	0.0	0.0	0.0	0.0	0.0	0.0	0.0
RsaHf231,																					
Unassigned *RsaHf231* (1)	0.0	0.0	0.0	0.0	0.0	0.0	0.0	0.0	0.0	0.0	0.0	0.0	0.0	0.0	0.0	0.0	0.0	0.0	0.0	0.0	0.0
Saccharibacteria,																					
Unassigned *Saccharibacteria* (78)	0.0	0.0	0.0	0.0	0.0	0.0	0.0	0.0	0.0	0.0	0.0	0.0	0.0	0.0	0.0	0.0	0.0	0.0	0.0	0.0	0.0
Spirochaetae,																					
Spirochaetes,																					
Spirochaetaceae (1)	0.0	0.0	0.0	0.0	0.0	0.0	0.0	0.0	0.0	0.0	0.0	0.0	0.0	0.0	0.0	0.0	0.0	0.0	0.0	0.0	0.0
Brevinemataceae (1)	0.0	0.0	0.0	0.0	0.0	0.0	0.0	0.0	0.0	0.0	0.0	0.0	0.0	0.0	0.0	0.0	0.0	0.0	0.0	0.0	0.0
Synergistetes,																					
Synergistia,																					
Synergistaceae (1)	0.0	0.0	0.0	0.0	0.0	0.0	0.0	0.0	0.0	0.0	0.0	0.0	0.0	0.0	0.0	0.0	0.0	0.0	0.0	0.0	0.0
Tectomicrobia,																					
Unassigned *Tectomicrobia* (21)	0.8	0.7	0.7	0.6	0.5	0.5	0.5	0.5	0.5	0.6	0.5	0.6	0.7	0.5	0.7	0.6	0.7	0.5	0.7	0.7	0.8
Tenericutes,																					
Mollicutes,																					
Unassigned *Entomoplasmatales* (3)	0.0	0.0	0.0	0.0	0.0	0.0	0.0	0.0	0.0	0.0	0.0	0.0	0.0	0.0	0.0	0.0	0.0	0.0	0.0	0.0	0.0
Haloplasmataceae (7)	0.0	0.0	0.0	0.0	0.0	0.0	0.0	0.0	0.0	0.0	0.0	0.0	0.0	0.0	0.0	0.0	0.0	0.0	0.0	0.0	0.0
Mycoplasmataceae (19)	16	16	17	14	17	15	11	13	13	16	14	16	13	18	16	19	19	17	17	16	17
Thaumarchaeota,																					
Unassigned *Thaumarchaeota* (9)	0.0	0.0	0.0	0.0	0.0	0.0	0.0	0.0	0.0	0.0	0.0	0.0	0.0	0.0	0.0	0.0	0.0	0.0	0.0	0.0	0.0
TM6_Dependentiae,																					
Unassigned *TM6_Dependentiae* (40)	0.0	0.0	0.0	0.0	0.0	0.0	0.0	0.0	0.0	0.0	0.0	0.0	0.0	0.0	0.0	0.0	0.0	0.0	0.0	0.0	0.0
Verrucomicrobia,																					

Sampling Time:	30 h																				
Treatment:	C1	C2	C3	CAA 1	CAA 2	CAA 3	Glu 1	Glu 2	Glu 3	Asp 1	Asp 2	Asp 3	Thr 1	Thr 2	Thr 3	Ala/ Gly1	Ala/ Gly2	Ala/ Gly3	Val/ Gly1	Val/ Gly2	Val/ Gly3
**Phyla, Class, Family[b]**	**Relative Abundance %**																				
Unassigned OPB35 soil group (87)	0.2	0.2	0.1	0.1	0.1	0.1	0.1	0.1	0.1	0.1	0.1	0.1	0.1	0.1	0.1	0.1	0.1	0.1	0.1	0.1	0.1
Opitutae,																					
Opitutaceae (5)	0.0	0.0	0.0	0.0	0.0	0.0	0.0	0.0	0.0	0.0	0.0	0.0	0.0	0.0	0.0	0.0	0.0	0.0	0.0	0.0	0.0
Unassigned *Opitutae* (2)	0.0	0.0	0.0	0.0	0.0	0.0	0.0	0.0	0.0	0.0	0.0	0.0	0.0	0.0	0.0	0.0	0.0	0.0	0.0	0.0	0.0
Spartobacteria,																					
Chthoniobacteraceae (46)	0.1	0.1	0.1	0.1	0.0	0.1	0.1	0.1	0.1	0.1	0.1	0.1	0.1	0.1	0.1	0.1	0.1	0.1	0.1	0.1	0.1
Unassigned *Chthoniobacterales* (2)	0.0	0.0	0.0	0.0	0.0	0.0	0.0	0.0	0.0	0.0	0.0	0.0	0.0	0.0	0.0	0.0	0.0	0.0	0.0	0.0	0.0
DA101 soil group (28)	0.9	0.7	0.8	0.5	0.5	0.5	0.5	0.6	0.5	0.8	0.7	0.6	0.5	0.6	0.6	0.7	0.6	0.6	0.5	0.5	0.5
Xiphinematobacteraceae (9)	2.0	1.6	1.5	1.7	1.1	1.5	1.4	0.9	1.1	1.2	1.3	1.0	1.7	1.5	1.6	1.9	1.8	1.5	1.9	1.7	1.9
Unassigned *Spartobacteria* (7)	0.0	0.0	0.0	0.0	0.0	0.0	0.0	0.0	0.0	0.0	0.0	0.0	0.0	0.0	0.0	0.0	0.0	0.0	0.0	0.0	0.0
Verrucomicrobiae,																					
Verrucomicrobiaceae (22)	0.0	0.0	0.0	0.0	0.0	0.0	0.0	0.0	0.0	0.0	0.0	0.0	0.0	0.0	0.0	0.0	0.0	0.0	0.0	0.0	0.0
Unassigned *Verrucomicrobia* (1)	0.0	0.0	0.0	0.0	0.0	0.0	0.0	0.0	0.0	0.0	0.0	0.0	0.0	0.0	0.0	0.0	0.0	0.0	0.0	0.0	0.0

[a]Samples of the three replicates of the 16S rRNA control treatment at 0 h, and all 16S rRNA treatments at 30 h were analyzed separately. Samples of the three replicates were pooled for each of the other treatments at 0 h, 10 h, 22h, or 30 h. Identification numbers (e.g., C1) indicate the respective replicates. Abbreviations: C, unsupplemented control; CAA, casamino acids; Glu, glutamate; Asp, aspartate; Thr, threonine; Ala, alanine, alanine; Gly, glycine; Val, valine.

[b]The number of phylotypes are shown in parenthesis. Abundant responsive group phylotypes and phylotypes from Figure 6 are bold and in brackets.

Table 12. Summary of all detected families in control and ribose treatments based on 16S rRNA gene (A) and 16S rRNA (B) analysis.[a]

	16S rRNA Genes								16S rRNA									
**Sampling Time:**	**0 h**				**30 h**				**0 h**				**30 h**					
**Treatment:**	**C1**	**C2**	**C3**	**R**	**C1**	**C2**	**C3**	**R**	**C1**	**C2**	**C3**	**R**	**C1**	**C2**	**C3**	**R1**	**R2**	**R3**
**Phyla, Class, Family[b]**	**Relative Abundance (%)**																	
Acidobacteria,																		
Acidobacteria,																		
Acidobacteriaceae (3)	0.0	0.0	0.0	0.0	0.0	0.0	0.0	0.0	0.0	0.0	0.0	0.0	0.0	0.0	0.0	0.0	0.0	0.0
Blastocatellia,																		
Blastocatellaceae(4)	0.0	0.1	0.1	0.1	0.0	0.0	0.0	0.0	0.0	0.0	0.1	0.0	0.0	0.0	0.0	0.0	0.0	0.0
Holophagae,																		
Unassigned *Holophagae* (4)	0.1	0.1	0.1	0.1	0.0	0.1	0.1	0.0	0.0	0.0	0.1	0.0	0.0	0.0	0.0	0.0	0.0	0.0
Solibacteres,																		
Solibacteraceae (12)	0.1	0.1	0.1	0.1	0.1	0.0	0.1	0.0	0.1	0.1	0.2	0.1	0.0	0.1	0.1	0.0	0.1	0.1
Subgroup_5 (5)	0.1	0.1	0.1	0.1	0.1	0.1	0.1	0.0	0.0	0.1	0.0	0.1	0.0	0.0	0.0	0.0	0.0	0.0
Subgroup_6 (46)	1.4	1.2	1.2	1.1	0.7	0.7	0.9	0.4	0.5	0.3	0.7	0.8	0.2	0.3	0.3	0.2	0.1	0.3
Subgroup_11 (3)	0.0	0.0	0.0	0.0	0.0	0.0	0.0	0.0	0.0	0.0	0.0	0.0	0.0	0.0	0.0	0.0	0.0	0.0
Subgroup_17 (4)	0.0	0.0	0.1	0.0	0.0	0.0	0.0	0.0	0.0	0.0	0.0	0.0	0.0	0.0	0.0	0.0	0.0	0.0
Subgroup_18 (1)	0.0	0.0	0.0	0.0	0.0	0.0	0.0	0.0	0.0	0.1	0.0	0.0	0.0	0.0	0.0	0.0	0.0	0.0
Subgroup_22 (6)	0.0	0.0	0.0	0.0	0.0	0.0	0.0	0.0	0.0	0.0	0.0	0.0	0.0	0.0	0.0	0.0	0.0	0.0
Subgroup_25 (3)	0.0	0.0	0.0	0.0	0.0	0.1	0.0	0.0	0.0	0.0	0.0	0.0	0.0	0.0	0.0	0.0	0.0	0.0
Actinobacteria,																		
Acidimicrobiia,																		
Acidimicrobiaceae (13)	0.8	1.1	1.4	0.9	0.7	1.0	0.8	0.3	0.6	0.6	1.3	0.5	0.5	0.6	0.7	0.5	0.3	0.5
Unassigned *Acidimicrobiales* (40)	1.8	1.8	2.3	1.7	1.1	1.4	1.4	0.7	0.9	1.2	1.9	0.9	0.6	0.7	1.3	0.8	0.8	1.0
Iamiaceae (5)	0.1	0.2	0.2	0.1	0.2	0.2	0.1	0.0	0.2	0.1	0.1	0.1	0.0	0.2	0.1	0.1	0.1	0.1
Actinobacteria,																		
Acidothermaceae (4)	0.3	0.2	0.3	0.2	0.2	0.2	0.1	0.1	0.1	0.1	0.2	0.2	0.0	0.1	0.1	0.0	0.1	0.1
Catenulisporaceae (1)	0.0	0.0	0.0	0.0	0.0	0.0	0.0	0.0	0.0	0.0	0.0	0.0	0.0	0.0	0.0	0.0	0.0	0.0
Cellulomonadaceae (1)	0.1	0.0	0.0	0.0	0.0	0.1	0.0	0.0	0.0	0.1	0.2	0.1	0.0	0.1	0.2	0.1	0.1	0.0
Demequinaceae (1)	0.0	0.0	0.0	0.0	0.0	0.0	0.0	0.0	0.0	0.0	0.0	0.0	0.0	0.0	0.0	0.0	0.0	0.0
Dermacoccaceae (1)	0.0	0.0	0.0	0.0	0.0	0.0	0.0	0.0	0.0	0.0	0.0	0.0	0.0	0.0	0.0	0.0	0.0	0.0
Frankiaceae (2)	0.1	0.1	0.1	0.0	0.1	0.1	0.1	0.0	0.1	0.0	0.1	0.1	0.0	0.1	0.0	0.0	0.0	0.0
Geodermatophilaceae (1)	0.0	0.0	0.0	0.0	0.0	0.0	0.0	0.0	0.1	0.0	0.0	0.0	0.0	0.0	0.0	0.0	0.0	0.0
Glycomycetaceae (1)	0.0	0.0	0.0	0.0	0.0	0.0	0.0	0.0	0.0	0.0	0.0	0.0	0.0	0.0	0.0	0.0	0.0	0.0
Intrasporangiaceae (3)	0.2	0.2	0.3	0.1	0.1	0.2	0.2	0.1	0.1	0.1	0.3	0.2	0.1	0.3	0.1	0.1	0.1	0.1
Kineosporiaceae (2)	0.0	0.0	0.0	0.0	0.0	0.1	0.0	0.0	0.0	0.0	0.0	0.0	0.0	0.0	0.0	0.0	0.0	0.0
Microbacteriaceae (7)	0.1	0.2	0.2	0.1	0.1	0.1	0.2	0.1	0.1	0.0	0.1	0.1	0.1	0.2	0.1	0.0	0.1	0.0
Micrococcaceae (2)	0.2	0.2	0.1	0.2	0.1	0.1	0.2	0.1	0.0	0.1	0.1	0.1	0.2	0.1	0.0	0.0	0.0	0.0
Micromonosporaceae (18)	1.1	1.1	1.3	1.0	0.6	0.9	0.8	0.4	0.9	0.9	1.8	1.0	0.6	0.9	0.8	0.6	0.6	0.6
Mycobacteriaceae (5)	0.4	0.5	0.5	0.4	0.3	0.3	0.3	0.1	0.0	0.0	0.1	0.1	0.1	0.1	0.1	0.0	0.1	0.1
Nakamurellaceae (1)	0.1	0.0	0.1	0.1	0.0	0.0	0.0	0.0	0.1	0.0	0.0	0.0	0.0	0.0	0.1	0.0	0.0	0.0
Nocardiaceae (5)	0.0	0.0	0.1	0.0	0.0	0.0	0.0	0.0	0.0	0.1	0.0	0.0	0.0	0.0	0.0	0.0	0.0	0.0
Nocardioidaceae (18)	1.3	1.4	1.6	1.2	0.9	1.1	1.0	0.4	0.4	0.6	1.4	0.8	0.4	0.8	0.5	0.3	0.5	0.4
Promicromonosporaceae (1)	0.0	0.0	0.0	0.0	0.0	0.0	0.0	0.0	0.0	0.0	0.0	0.0	0.0	0.0	0.0	0.0	0.0	0.0
Propionibacteriaceae (6)	0.5	0.4	0.4	0.4	0.2	0.3	0.3	0.2	0.2	0.1	0.2	0.2	0.1	0.1	0.2	0.1	0.2	0.1
Pseudonocardiaceae (10)	0.5	0.5	0.5	0.4	0.3	0.5	0.4	0.1	0.2	0.3	0.9	0.6	0.2	0.3	0.2	0.3	0.3	0.3
Sporichthyaceae (3)	0.1	0.1	0.1	0.1	0.0	0.1	0.1	0.0	0.0	0.1	0.2	0.0	0.0	0.1	0.1	0.0	0.0	0.0
Streptomycetaceae (4)	0.8	0.7	0.7	0.6	0.5	0.5	0.6	0.3	0.2	0.2	0.8	0.5	0.3	0.3	0.3	0.2	0.1	0.2
Streptosporangiaceae (2)	0.0	0.1	0.0	0.1	0.0	0.0	0.1	0.0	0.0	0.0	0.0	0.0	0.0	0.0	0.0	0.0	0.0	0.0
Thermomonosporaceae (3)	0.1	0.1	0.1	0.0	0.0	0.0	0.0	0.0	0.0	0.0	0.0	0.0	0.0	0.0	0.0	0.0	0.0	0.0
Unassigned *Actinobacteria* (1)	0.0	0.0	0.0	0.0	0.0	0.0	0.0	0.0	0.0	0.1	0.1	0.1	0.0	0.0	0.0	0.0	0.0	0.0
Coriobacteriia,																		

	16S rRNA Genes								16S rRNA									
Sampling Time:	0 h				30 h				0 h				30 h					
Treatment:	C1	C2	C3	R	C1	C2	C3	R	C1	C2	C3	R	C1	C2	C3	R1	R2	R3
**Phyla, Class, Family**[b]	**Relative Abundance (%)**																	
Coriobacteriaceae (2)	0.0	0.0	0.0	0.0	0.0	0.0	0.0	0.0	0.0	0.0	0.0	0.0	0.0	0.0	0.0	0.0	0.0	0.0
Rubrobacteria,																		
Rubrobacteriaceae (3)	0.1	0.1	0.1	0.1	0.0	0.1	0.0	0.0	0.0	0.0	0.1	0.1	0.1	0.1	0.2	0.0	0.0	0.0
Thermoleophilia,																		
Conexibacteraceae (1)	0.0	0.0	0.0	0.0	0.0	0.0	0.0	0.0	0.0	0.0	0.0	0.0	0.0	0.0	0.0	0.0	0.0	0.0
Unassigned *Thermoleophilia* (28)	1.5	1.6	1.9	1.5	1.1	1.2	1.4	0.5	0.3	0.4	0.5	0.5	0.2	0.2	0.3	0.3	0.2	0.3
Gaiellaceae (5)	1.9	1.9	2.3	1.7	1.3	1.5	1.4	0.7	0.4	0.5	0.6	0.7	0.3	0.5	0.5	0.3	0.2	0.5
Unassigned *Gaiellales* (28)	3.5	4.2	4.3	3.0	2.0	2.6	2.6	1.1	0.8	0.8	1.4	1.0	0.4	1.0	0.8	0.5	0.5	1.0
Parviterribacteraceae (1)	0.0	0.0	0.0	0.0	0.0	0.0	0.0	0.0	0.0	0.0	0.0	0.0	0.0	0.0	0.0	0.0	0.0	0.0
Patulibacteraceae (3)	0.0	0.0	0.0	0.0	0.0	0.0	0.0	0.0	0.0	0.0	0.0	0.0	0.0	0.0	0.0	0.0	0.0	0.0
Solirubrobacteraceae (4)	0.5	0.6	0.7	0.4	0.4	0.6	0.6	0.2	0.7	0.7	1.3	0.9	0.4	0.7	0.7	0.5	0.5	0.8
Unassigned *Solirubrobacterales* (13)	0.9	0.8	1.1	0.7	0.4	0.7	0.8	0.3	0.3	0.3	0.4	0.4	0.1	0.3	0.1	0.2	0.2	0.2
Unassigned *Actinobacteria* (23)	2.2	2.8	3.2	2.0	1.6	1.6	1.8	0.7	0.3	0.6	0.9	0.5	0.2	0.6	0.4	0.3	0.2	0.3
Armatimonadetes,																		
Armatimonadia,																		
Unassigned *Armatimonadia* (1)	0.0	0.0	0.0	0.0	0.0	0.0	0.0	0.0	0.0	0.0	0.0	0.0	0.0	0.0	0.0	0.0	0.0	0.0
Bacteroidetes,																		
Bacteroidia,																		
Bacteroidaceae (5)	1.4	0.0	0.0	0.0	0.2	0.6	0.4	0.3	0.1	0.0	0.0	0.1	0.0	0.0	0.0	0.0	0.0	0.1
Porphyromonadaceae (1)	0.0	0.0	0.0	0.0	0.0	0.0	0.0	0.0	0.0	0.0	0.0	0.0	0.0	0.0	0.0	0.0	0.0	0.0
Rikenellaceae (1)	0.0	0.0	0.0	0.0	0.0	0.0	0.0	0.0	0.0	0.0	0.0	0.0	0.0	0.0	0.0	0.0	0.0	0.0
Cytophagia,																		
Cytophagaceae (6)	0.0	0.0	0.0	0.0	0.0	0.0	0.0	0.0	0.0	0.0	0.1	0.0	0.0	0.0	0.0	0.0	0.0	0.0
Flavobacteriia,																		
Flavobacteriaceae (9)	0.2	0.3	0.2	0.2	0.5	0.8	0.4	0.2	0.1	0.0	0.0	0.1	0.0	0.4	0.1	0.1	0.1	0.2
Sphingobacteriia,																		
Chitinophagaceae (5)	0.0	0.0	0.0	0.0	0.0	0.0	0.0	0.0	0.0	0.1	0.0	0.0	0.0	0.0	0.0	0.0	0.0	0.0
Sphingobacteriaceae (2)	0.0	0.0	0.0	0.0	0.0	0.0	0.0	0.0	0.0	0.0	0.0	0.0	0.0	0.0	0.0	0.0	0.0	0.0
Unassigned *Sphingobacteriales* (1)	0.0	0.0	0.0	0.0	0.0	0.0	0.0	0.0	0.0	0.0	0.0	0.0	0.0	0.0	0.0	0.0	0.0	0.0
BRC1,																		
Unassigned *BRC*1 (2)	0.0	0.0	0.0	0.0	0.0	0.0	0.0	0.0	0.0	0.0	0.0	0.0	0.0	0.0	0.0	0.0	0.0	0.0
Chlamydiae,																		
Chlamydiae,																		
Parachlamydiaceae (3)	0.0	0.0	0.0	0.0	0.0	0.0	0.0	0.0	0.0	0.0	0.0	0.0	0.0	0.0	0.0	0.0	0.0	0.0
Chloroflexi,																		
Anaerolineae,																		
Anaerolineaceae (6)	0.0	0.0	0.0	0.0	0.0	0.0	0.0	0.0	0.0	0.0	0.0	0.0	0.0	0.0	0.0	0.0	0.0	0.0
Ardenticatenia (1)	0.0	0.0	0.0	0.0	0.0	0.0	0.0	0.0	0.0	0.0	0.0	0.0	0.0	0.0	0.0	0.0	0.0	0.0
Caldilineae,																		
Caldilineaceae (10)	0.2	0.2	0.1	0.1	0.1	0.1	0.2	0.1	0.0	0.1	0.1	0.1	0.0	0.0	0.0	0.0	0.0	0.0
Chloroflexia,																		
Roseiflexaceae (4)	0.1	0.1	0.1	0.1	0.1	0.1	0.1	0.0	0.1	0.1	0.2	0.2	0.1	0.1	0.0	0.0	0.0	0.1
Unassigned *Kallotenuales* (1)	0.0	0.0	0.0	0.0	0.0	0.0	0.0	0.0	0.0	0.0	0.0	0.0	0.0	0.0	0.0	0.0	0.0	0.0
Ktedonobacteria,																		
Unassigned *Ktedonobacterales* (1)	0.0	0.0	0.0	0.0	0.0	0.0	0.0	0.0	0.0	0.0	0.0	0.0	0.0	0.0	0.0	0.0	0.0	0.0
Unassigned *Ktedonobacteria* (6)	0.1	0.1	0.1	0.1	0.1	0.1	0.1	0.0	0.0	0.0	0.1	0.1	0.1	0.0	0.0	0.0	0.0	0.0
Thermomicrobia,																		
Unassigned *Thermomicrobia* (27)	0.4	0.5	0.4	0.3	0.2	0.2	0.3	0.1	0.1	0.2	0.2	0.2	0.1	0.1	0.1	0.1	0.1	0.1

	16S rRNA Genes								16S rRNA											
**Sampling Time:**	**0 h**				**30 h**				**0 h**				**30 h**							
**Treatment:**	**C1**	**C2**	**C3**	**R**	**C1**	**C2**	**C3**	**R**	**C1**	**C2**	**C3**	**R**	**C1**	**C2**	**C3**	**R1**	**R2**	**R3**
**Phyla, Class, Family**[b]	**Relative Abundance (%)**																			
Unassigned *Chloroflexi* (51)	2.4	2.7	3.2	2.3	1.7	1.9	1.4	0.8	0.9	0.9	1.5	0.9	0.4	0.8	0.8	0.4	0.5	0.6
Cyanobacteria,																		
Chloroplast,																		
Unassigned *Chloroplast* (2)	0.0	0.0	0.0	0.0	0.0	0.0	0.0	0.0	0.0	0.0	0.0	0.0	0.0	0.0	0.0	0.0	0.0	0.0
Cyanobacteria,																		
Unassigned *Cyanobacteria* (2)	0.0	0.0	0.0	0.0	0.0	0.0	0.0	0.0	0.0	0.0	0.0	0.0	0.0	0.0	0.0	0.0	0.0	0.0
Euryarchaeota,																		
Methanomicrobia,																		
Methanocellaceae (1)	0.0	0.0	0.0	0.0	0.0	0.0	0.0	0.0	0.0	0.0	0.0	0.0	0.0	0.0	0.0	0.0	0.0	0.0
Firmicutes,																		
Bacilli,																		
Alicyclobacillaceae (2)	0.0	0.0	0.0	0.0	0.0	0.0	0.0	0.0	0.0	0.0	0.0	0.0	0.0	0.0	0.0	0.0	0.0	0.0
Bacillaceae (6)	0.6	0.5	0.4	0.5	0.5	0.5	0.6	0.3	0.4	0.2	0.6	0.7	0.1	0.4	0.3	0.4	0.3	0.4
Lactobacillaceae (1)	0.0	0.0	0.0	0.0	0.0	0.0	0.0	0.0	0.0	0.0	0.0	0.0	0.0	0.0	0.0	0.0	0.0	0.0
Unassigned *Lactobacillales* (1)	0.2	0.0	0.0	0.0	0.0	0.0	0.0	0.0	0.0	0.0	0.0	0.0	0.0	0.0	0.0	0.0	0.0	0.0
Paenibacillaceae (18)	0.1	0.1	0.2	0.1	0.1	0.2	0.2	1.0	0.0	0.1	0.1	0.1	0.1	0.1	0.0	1.4	1.7	1.4
Pasteuriaceae (1)	0.0	0.0	0.0	0.0	0.0	0.0	0.0	0.0	0.0	0.0	0.0	0.0	0.0	0.0	0.0	0.0	0.0	0.0
Planococcaceae (4)	0.1	0.1	0.1	0.0	0.0	0.0	0.1	0.1	0.1	0.0	0.0	0.0	0.0	0.0	0.0	0.0	0.0	0.0
Streptococcaceae (1)	0.0	0.0	0.0	0.0	0.0	0.0	0.0	0.0	0.0	0.0	0.0	0.0	0.0	0.0	0.0	0.0	0.0	0.0
Thermoactinomycetaceae (2)	0.0	0.0	0.0	0.0	0.0	0.0	0.0	0.0	0.0	0.0	0.0	0.0	0.0	0.0	0.0	0.0	0.0	0.0
Unassigned *Bacilli* (2)	0.6	0.7	0.5	0.8	0.3	0.3	0.3	0.1	0.4	0.5	0.7	0.6	0.3	0.2	0.3	0.2	0.2	0.2
Clostridia,																		
Christensenellaceae (1)	0.0	0.0	0.0	0.0	0.0	0.0	0.0	0.0	0.0	0.0	0.0	0.0	0.0	0.0	0.0	0.0	0.0	0.0
Clostridiaceae (16) **[A14]**	0.1	0.1	0.0	0.1	0.8	1.0	1.0	0.6	0.0	0.1	0.2	0.2	2.0	1.2	1.5	0.8	1.3	1.1
Unassigned *Clostridiales* (3)	0.0	0.0	0.0	0.0	0.0	0.0	0.2	0.0	0.0	0.0	0.0	0.0	0.2	0.1	0.1	0.0	0.0	0.0
Eubacteriaceae (1)	0.0	0.0	0.0	0.0	0.0	0.0	0.0	0.0	0.0	0.0	0.0	0.0	0.0	0.0	0.0	0.0	0.0	0.0
Gracilibacteraceae (2)	0.0	0.0	0.0	0.0	0.0	0.0	0.0	0.0	0.0	0.0	0.0	0.0	0.0	0.0	0.0	0.0	0.0	0.0
Heliobacteriaceae (3)	0.0	0.0	0.1	0.0	0.0	0.0	0.1	0.0	0.0	0.0	0.0	0.0	0.0	0.0	0.0	0.0	0.0	0.0
Lachnospiraceae (13)	0.0	0.0	0.0	0.0	0.5	0.7	0.8	0.8	0.0	0.0	0.0	0.1	1.5	0.8	0.9	0.6	0.7	0.6
Peptococcaceae (4)	1.4	1.5	1.3	1.8	1.0	1.1	0.9	1.0	1.2	1.3	2.3	0.8	1.2	0.9	0.9	1.3	1.2	0.9
Peptostreptococcaceae (4) **[GPT-4], [A8]**	0.0	0.0	0.0	0.0	0.1	0.1	0.2	0.1	0.0	0.0	0.1	0.0	0.1	0.2	0.1	0.1	0.0	0.1
Ruminococcaceae (11)	0.0	0.0	0.0	0.0	0.1	0.1	0.0	0.1	0.0	0.0	0.0	0.1	0.0	0.1	0.1	0.0	0.1	0.1
Unassigned *Clostridia* (2)	0.0	0.0	0.0	0.0	0.0	0.0	0.0	0.0	0.0	0.0	0.0	0.0	0.0	0.0	0.0	0.0	0.0	0.0
Erysipelotrichia,																		
Erysipelotrichaceae (3)	0.0	0.0	0.0	0.0	0.0	0.0	0.0	0.0	0.0	0.0	0.0	0.0	0.0	0.0	0.0	0.0	0.0	0.0
Negativicutes,																		
Veillonellaceae (2)	0.0	0.0	0.0	0.0	0.0	0.0	0.0	0.0	0.0	0.0	0.0	0.0	0.0	0.0	0.0	0.0	0.0	0.0
Fusobacteria,																		
Fusobacteriia,																		
Fusobacteriaceae (1) **[GPT-5]**	14	13	9.8	19	20	19	18	20	22	19	1.8	12	27	22	25	20	16	14
Gemmatimonadetes,																		
Gemmatimonadetes,																		
Gemmatimonadaceae (14)	0.2	0.2	0.3	0.3	0.2	0.2	0.3	0.1	0.1	0.1	0.2	0.1	0.0	0.2	0.2	0.1	0.0	0.1
Latescibacteria,																		
Unassigned *Latescrbacteria* (4)	0.1	0.0	0.0	0.0	0.0	0.0	0.0	0.0	0.0	0.0	0.0	0.0	0.0	0.0	0.0	0.0	0.0	0.0
Nitrospirae,																		
Nitrospira,																		
Nitrospiraceae (5)	0.0	0.0	0.1	0.1	0.0	0.1	0.1	0.0	0.0	0.1	0.1	0.1	0.1	0.0	0.0	0.0	0.0	0.0

	16S rRNA Genes								16S rRNA										
Sampling Time:	0 h				30 h				0 h				30 h						
Treatment:	C1	C2	C3	R	C1	C2	C3	R	C1	C2	C3	R	C1	C2	C3	R1	R2	R3
**Phyla, Class, Family**[b]	Relative Abundance (%)																		
Unassigned *Nitrospirales* (10)	0.6	0.7	0.7	0.4	0.4	0.5	0.4	0.2	0.1	0.2	0.5	0.4	0.2	0.5	0.2	0.3	0.3	0.3
Planctomycetes,																		
Phycisphaerae,																		
Phycisphaeraceae (1)	0.0	0.0	0.0	0.0	0.0	0.0	0.0	0.0	0.0	0.0	0.0	0.0	0.0	0.0	0.0	0.0	0.0	0.0
Unassigned *Phycisphaerae* (7)	0.0	0.1	0.1	0.0	0.0	0.0	0.0	0.0	0.0	0.0	0.0	0.0	0.0	0.0	0.1	0.0	0.0	0.0
Unassigned *Phycisphaerales* (1)	0.0	0.0	0.0	0.0	0.0	0.0	0.0	0.0	0.0	0.0	0.0	0.0	0.0	0.0	0.0	0.0	0.0	0.0
Tepidisphaeraceae (34)	0.4	0.6	0.6	0.5	0.4	0.4	0.4	0.2	0.4	0.5	0.8	0.4	0.2	0.2	0.4	0.2	0.2	0.3
Planctomycetacia,																		
Planctomycetaceae (331)	2.6	2.9	3.6	2.5	2.0	2.5	1.8	1.0	7.1	7.1	15	6.1	5.9	8.1	8.5	5.4	5.4	5.8
Unassigned *Planctomycetes* (17)	0.1	0.0	0.1	0.1	0.0	0.1	0.0	0.0	0.0	0.0	0.0	0.1	0.0	0.0	0.0	0.0	0.0	0.1
Proteobacteria,																		
Alphaproteobacteria,																		
Acetobacteraceae (4)	0.0	0.0	0.0	0.0	0.0	0.0	0.0	0.0	0.1	0.1	0.1	0.1	0.0	0.1	0.0	0.1	0.0	0.1
Beijerinckiaceae (1)	0.0	0.0	0.0	0.0	0.0	0.0	0.0	0.0	0.0	0.0	0.0	0.0	0.0	0.0	0.0	0.0	0.0	0.0
Bradyrhizobiaceae (3)	0.7	0.8	0.8	0.7	0.5	0.5	0.5	0.2	0.5	0.6	0.8	0.6	0.4	0.7	0.6	0.2	0.3	0.5
Caulobacteraceae (2)	0.0	0.0	0.0	0.0	0.0	0.0	0.0	0.0	0.0	0.0	0.0	0.0	0.0	0.0	0.0	0.0	0.0	0.0
Unassigned *Sphingomonadales* (1)	0.0	0.0	0.0	0.0	0.0	0.0	0.0	0.0	0.0	0.0	0.0	0.0	0.0	0.0	0.0	0.0	0.0	0.0
Hyphomicrobiaceae (4)	0.2	0.3	0.2	0.2	0.2	0.1	0.2	0.1	0.1	0.1	0.2	0.1	0.1	0.1	0.1	0.0	0.0	0.1
Methylobacteriaceae (3)	0.1	0.1	0.2	0.2	0.1	0.1	0.2	0.0	0.1	0.2	0.2	0.2	0.0	0.1	0.1	0.1	0.2	0.1
Phyllobacteriaceae (3)	0.2	0.2	0.2	0.1	0.1	0.2	0.2	0.0	0.2	0.0	0.4	0.2	0.1	0.0	0.3	0.0	0.0	0.1
Rhizobiaceae (1)	0.0	0.0	0.0	0.0	0.0	0.0	0.0	0.0	0.0	0.0	0.0	0.0	0.0	0.0	0.0	0.0	0.0	0.0
Rhodobacteraceae (3)	0.0	0.0	0.0	0.0	0.0	0.0	0.0	0.0	0.0	0.1	0.0	0.0	0.0	0.0	0.0	0.0	0.0	0.0
Rhodobiaceae (2)	1.0	1.0	0.9	0.6	0.7	0.7	0.7	0.2	0.2	0.1	0.4	0.2	0.0	0.2	0.1	0.1	0.1	0.1
Rhodospirillaceae (13)	0.4	0.5	0.5	0.5	0.4	0.4	0.4	0.1	0.2	0.3	1.0	0.5	0.5	0.5	0.4	0.3	0.2	0.4
Unassigned *Rhodospirillales* (23)	0.5	0.5	0.6	0.4	0.4	0.4	0.4	0.1	0.3	0.3	0.7	0.6	0.2	0.5	0.4	0.3	0.5	0.4
Sphingomonadaceae (2)	0.0	0.0	0.0	0.0	0.0	0.0	0.0	0.0	0.0	0.0	0.0	0.0	0.0	0.0	0.0	0.0	0.0	0.0
Xanthobacteraceae (3)	2.5	2.6	3.1	2.1	1.7	1.9	2.3	0.7	0.7	1.3	1.7	1.0	0.5	0.8	1.1	0.6	0.5	0.7
Unassigned *Rhizobiales* (12)	0.2	0.2	0.2	0.1	0.2	0.2	0.2	0.1	0.1	0.1	0.3	0.1	0.0	0.1	0.0	0.0	0.1	0.1
Betaproteobacteria,									0.0	0.0	0.0	0.0	0.0	0.0	0.0	0.0	0.0	0.0
Alcaligenaceae (1)	0.1	0.1	0.1	0.1	0.1	0.1	0.1	0.0	0.0	0.0	0.0	0.1	0.0	0.0	0.0	0.0	0.0	0.0
Burkholderiaceae (2)	0.0	0.0	0.0	0.0	0.0	0.0	0.0	0.0	0.0	0.0	0.0	0.0	0.0	0.0	0.0	0.0	0.0	0.0
Comamonadaceae (8)	0.1	0.0	0.1	0.0	0.0	0.1	0.0	0.0	0.0	0.1	0.2	0.1	0.0	0.0	0.0	0.0	0.0	0.0
Gallionellaceae (1)	0.0	0.0	0.0	0.0	0.0	0.0	0.0	0.0	0.0	0.0	0.0	0.0	0.0	0.0	0.0	0.0	0.0	0.0
Nitrosomonadaceae (16)	0.3	0.2	0.3	0.2	0.1	0.2	0.1	0.1	0.1	0.0	0.2	0.1	0.0	0.0	0.1	0.1	0.0	0.1
Oxalobacteraceae (2)	0.0	0.0	0.0	0.0	0.0	0.0	0.0	0.0	0.0	0.0	0.0	0.0	0.0	0.0	0.0	0.0	0.0	0.0
Rhodocyclaceae (3)	0.0	0.0	0.0	0.0	0.0	0.0	0.0	0.0	0.0	0.0	0.0	0.0	0.0	0.0	0.0	0.0	0.0	0.0
Unassigned *Betaproteobacteria* (11)	0.1	0.2	0.2	0.0	0.1	0.1	0.1	0.0	0.1	0.1	0.2	0.1	0.0	0.0	0.1	0.0	0.1	0.0
Deltaproteobacteria,																		
Unassigned *Oligoflexales* (5)	0.0	0.0	0.0	0.0	0.0	0.0	0.0	0.0	0.0	0.0	0.0	0.0	0.0	0.0	0.0	0.0	0.0	0.0
Archangiaceae (5)	0.1	0.1	0.1	0.1	0.1	0.1	0.0	0.0	0.0	0.1	0.2	0.3	0.1	0.1	0.1	0.1	0.0	0.1
Bdellovibrionaceae (6)	0.0	0.0	0.0	0.0	0.0	0.0	0.0	0.0	0.0	0.0	0.0	0.0	0.0	0.0	0.0	0.0	0.0	0.0
Desulfobulbaceae (1)	0.0	0.0	0.0	0.0	0.0	0.0	0.0	0.0	0.0	0.0	0.0	0.0	0.0	0.0	0.0	0.0	0.0	0.0
Desulfurellaceae (19)	0.8	0.7	0.9	0.6	0.4	0.4	0.7	0.2	0.2	0.1	0.6	0.5	0.1	0.1	0.2	0.1	0.1	0.2
Desulfuromonadaceae (2)	0.0	0.0	0.0	0.0	0.0	0.0	0.0	0.0	0.0	0.0	0.0	0.0	0.0	0.0	0.0	0.0	0.0	0.0
Geobacteraceae (9)	0.1	0.1	0.1	0.1	0.1	0.1	0.1	0.0	0.0	0.1	0.2	0.2	0.0	0.0	0.1	0.0	0.0	0.0
Haliangiaceae (26)	0.1	0.2	0.1	0.1	0.1	0.1	0.2	0.0	0.1	0.2	0.4	0.3	0.2	0.1	0.2	0.1	0.1	0.1
Myxococcaceae (1)	0.0	0.0	0.0	0.0	0.0	0.0	0.0	0.0	0.0	0.0	0.0	0.0	0.0	0.0	0.0	0.0	0.0	0.0
Nannocystaceae (2)	0.0	0.0	0.0	0.0	0.0	0.0	0.0	0.0	0.0	0.0	0.0	0.0	0.0	0.0	0.0	0.0	0.0	0.0
Phaselicystidaceae (1)	0.0	0.0	0.1	0.0	0.0	0.0	0.0	0.0	0.0	0.0	0.1	0.1	0.0	0.0	0.0	0.0	0.0	0.1

	16S rRNA Genes								16S rRNA									
**Sampling Time:**	**0 h**				**30 h**				**0 h**				**30 h**					
**Treatment:**	**C1**	**C2**	**C3**	**R**	**C1**	**C2**	**C3**	**R**	**C1**	**C2**	**C3**	**R**	**C1**	**C2**	**C3**	**R1**	**R2**	**R3**
**Phyla, Class, Family**[b]	**Relative Abundance (%)**																	
Polyangiaceae (17)	0.1	0.0	0.1	0.0	0.1	0.0	0.0	0.0	0.3	0.0	0.4	0.2	0.1	0.2	0.1	0.0	0.1	0.1
Sandaracinaceae (13)	0.0	0.1	0.1	0.1	0.0	0.0	0.1	0.0	0.1	0.1	0.2	0.2	0.0	0.0	0.0	0.0	0.0	0.0
Vulgatibacteraceae (1)	0.0	0.0	0.0	0.0	0.0	0.0	0.0	0.0	0.0	0.0	0.0	0.0	0.0	0.0	0.0	0.0	0.0	0.0
Unassigned *Myxococcales* (31)	0.1	0.2	0.1	0.1	0.2	0.1	0.2	0.1	0.1	0.1	0.3	0.2	0.0	0.2	0.2	0.1	0.1	0.2
Unassigned *Deltaproteobacteria* (4)	0.0	0.0	0.0	0.0	0.0	0.0	0.0	0.0	0.0	0.0	0.0	0.0	0.0	0.0	0.0	0.0	0.0	0.0
Gammaproteobacteria,																		
Aeromonadacea (3) **[GPT-1]**	22	22	23	22	24	20	19	42	27	21	5.0	34	21	25	21	39	41	44
Coxiellaceae (1)	0.1	0.1	0.1	0.1	0.1	0.1	0.1	0.0	0.0	0.0	0.0	0.0	0.0	0.0	0.0	0.0	0.0	0.0
Enterobacteriaceae (5) **[GPT-2], [GPT-3]**	1.4	2.4	1.5	1.6	1.7	2.0	2.2	5.0	2.5	3.0	0.4	3.5	1.9	2.0	1.9	4.1	5.0	4.1
Legionellaceae (1)	0.0	0.0	0.0	0.0	0.0	0.0	0.0	0.0	0.0	0.0	0.0	0.0	0.0	0.0	0.0	0.0	0.0	0.0
Oleiphilaceae (1)	0.0	0.0	0.0	0.0	0.0	0.0	0.0	0.0	0.0	0.0	0.0	0.0	0.0	0.0	0.0	0.0	0.0	0.0
Pseudomonadaceae (2)	0.0	0.0	0.1	0.0	0.1	0.0	0.1	0.0	0.1	0.0	0.0	0.1	0.1	0.1	0.0	0.0	0.1	0.0
Shewanellaceae (4)	8.4	8.6	8.8	7.9	15	15	16	10	3.1	2.3	0.7	3.9	2.4	5.1	4.3	4.5	3.6	4.9
Xanthomonadaceae (1)	0.0	0.0	0.0	0.0	0.0	0.0	0.0	0.0	0.0	0.0	0.0	0.0	0.0	0.0	0.0	0.0	0.0	0.0
Unassigned *Xanthomonadales* (12)	0.1	0.2	0.2	0.1	0.1	0.1	0.1	0.0	0.1	0.1	0.2	0.1	0.0	0.0	0.1	0.0	0.0	0.0
Unassigned *Gammaproteobacteria* (2)	0.0	0.0	0.0	0.0	0.0	0.0	0.0	0.0	0.0	0.0	0.0	0.0	0.0	0.0	0.0	0.0	0.0	0.0
Saccharibacteria,																		
Unassigned *Saccharibacteria* (1)	0.0	0.0	0.0	0.0	0.0	0.0	0.0	0.0	0.0	0.0	0.0	0.0	0.0	0.0	0.0	0.0	0.0	0.0
Spirochaetae,																		
Spirochaetes,																		
Spirochaetaceae (1)	0.0	0.0	0.0	0.0	0.0	0.0	0.0	0.0	0.0	0.0	0.0	0.0	0.0	0.0	0.0	0.0	0.0	0.0
Tectomicrobia,																		
Unassigned *Tectomicrobia* (10)	0.2	0.2	0.2	0.2	0.2	0.2	0.2	0.1	0.4	0.6	0.9	0.9	0.5	0.7	0.5	0.4	0.3	0.6
Tenericutes,																		
Mollicutes,																		
Unassigned *Entomoplasmatales* (2)	0.0	0.0	0.0	0.0	0.0	0.0	0.0	0.0	0.0	0.0	0.0	0.0	0.0	0.0	0.0	0.0	0.0	0.0
Mycoplasmataceae (5)	10	8.8	6.7	10	8.4	7.2	8.7	5.4	21	28	41	17	25	17	18	12	12	8.8
Thaumarchaeota,																		
Unassigned *Thaumarchaeota* (1)	0.0	0.0	0.0	0.0	0.0	0.0	0.0	0.0	0.0	0.0	0.0	0.0	0.0	0.0	0.0	0.0	0.0	0.0
Verrucomicrobia,																		
OPB35 soil group (29)	0.1	0.2	0.1	0.2	0.1	0.1	0.1	0.1	0.2	0.0	0.1	0.1	0.0	0.1	0.0	0.0	0.0	0.1
Opitutae,																		
Opitutaceae (1)	0.0	0.0	0.0	0.0	0.0	0.0	0.0	0.0	0.0	0.0	0.0	0.0	0.0	0.0	0.0	0.0	0.0	0.0
Spartobacteria,																		
Chthoniobacteraceae (10)	0.0	0.1	0.1	0.1	0.0	0.0	0.0	0.0	0.0	0.0	0.1	0.0	0.0	0.0	0.0	0.0	0.0	0.0
Unassigned *Chthoniobacterales* (17)	1.0	1.4	1.3	1.3	0.8	0.9	0.9	0.3	0.3	0.1	0.4	0.2	0.1	0.1	0.2	0.1	0.0	0.1
Xiphinematobacteraceae (3)	0.9	1.0	0.8	1.1	1.0	1.0	0.7	0.4	0.7	1.2	1.8	0.8	0.8	0.7	1.0	0.4	0.7	0.5
Verrucomicrobiae,																		
Verrucomicrobiaceae (4)	0.0	0.0	0.0	0.0	0.0	0.0	0.0	0.3	0.0	0.0	0.0	0.0	0.0	0.0	0.0	0.0	0.0	0.0

[a]Samples of the three replicates of the 16S rRNA gene control treatment at 0 h and 30 h, 16S rRNA control treatment at 0 h, and all 16S rRNA treatments at 30 h were analyzed separately. Samples of the three replicates were pooled for each of the other treatments at 0 h or 30 h. Identification numbers (e.g., C1) indicate the respective replicates. Abbreviations: C, unsupplemented control; R, ribose.

[b]The number of phylotypes are shown in parenthesis. Abundant responsive group phylotypes from Figure 6 are bold and in brackets.

Table S13. Summary of all detected families in control, glucose, formate, and succinate treatments based on 16S rRNA gene (A) and 16S rRNA (B) analysis.[a]

(A) 16S rRNA genes

Sampling Time:	0 h				10 h		22 h		30 h			
Treatment:	C	G	F	S	C	G	C	G	C	G	F	S
Phyla, Class, Family[b]	Relative Abundance (%)											
Acidobacteria,												
Acidobacteria,												
Acidobacteriaceae (3)	0.0	0.0	0.0	0.0	0.0	0.0	0.0	0.0	0.0	0.0	0.0	0.0
Blastocatellia,												
Blastocatellaceae (9)	0.2	0.1	0.1	0.1	0.1	0.0	0.1	0.0	0.1	0.1	0.1	0.1
Holophagae,												
Unassigned *Holophagae* (4)	0.1	0.1	0.1	0.1	0.1	0.0	0.1	0.0	0.0	0.0	0.1	0.1
Solibacteres,												
Solibacteraceae (10)	0.1	0.1	0.0	0.0	0.1	0.0	0.0	0.0	0.0	0.0	0.0	0.0
Subgroup_11 (3)	0.0	0.1	0.1	0.1	0.0	0.0	0.0	0.0	0.0	0.0	0.0	0.0
Subgroup_17 (9)	0.1	0.1	0.1	0.1	0.1	0.0	0.0	0.0	0.0	0.0	0.1	0.0
Subgroup_18 (1)	0.0	0.0	0.0	0.0	0.0	0.0	0.0	0.0	0.0	0.0	0.0	0.0
Subgroup_22 (5)	0.1	0.1	0.0	0.0	0.0	0.0	0.0	0.0	0.0	0.0	0.0	0.0
Subgroup_25 (3)	0.1	0.1	0.1	0.0	0.0	0.0	0.0	0.0	0.0	0.0	0.0	0.0
Subgroup_5 (6)	0.1	0.2	0.1	0.1	0.0	0.0	0.1	0.0	0.0	0.0	0.0	0.0
Subgroup_6 (49)	1.8	1.4	1.8	1.2	0.8	0.2	0.7	0.4	0.7	0.3	0.7	0.4
Subgroup_9 (1)	0.0	0.0	0.0	0.0	0.0	0.0	0.0	0.0	0.0	0.0	0.0	0.0
Actinobacteria,												
Acidimicrobiia,												
Acidimicrobiaceae (17)	1.6	1.7	1.6	1.2	1.0	0.4	0.9	0.4	0.9	0.7	1.1	0.7
Iamiaceae (8)	0.4	0.2	0.3	0.3	0.2	0.0	0.2	0.1	0.2	0.1	0.2	0.1
Unassigned *Acidimicrobiales* (39)	2.7	2.9	2.5	2.2	1.5	0.5	1.3	0.8	1.4	1.0	1.5	1.0
Actinobacteria,												
Acidothermaceae (4)	0.2	0.2	0.1	0.1	0.1	0.0	0.1	0.0	0.1	0.1	0.1	0.0
Actinomycetaceae (1)	0.0	0.0	0.0	0.0	0.0	0.0	0.0	0.0	0.0	0.0	0.0	0.0
Bifidobacteriaceae (3)	0.0	0.0	0.0	0.0	0.0	0.0	0.0	0.0	0.0	0.1	0.0	0.0
Bogoriellaceae (1)	0.0	0.0	0.0	0.0	0.0	0.0	0.0	0.0	0.0	0.0	0.0	0.0
Cellulomonadaceae (1)	0.0	0.0	0.1	0.0	0.0	0.0	0.0	0.0	0.0	0.1	0.1	0.0
Corynebacteriaceae (1)	0.0	0.0	0.0	0.0	0.0	0.0	0.0	0.0	0.0	0.0	0.0	0.0
Cryptosporangiaceae (2)	0.0	0.0	0.0	0.0	0.0	0.0	0.0	0.0	0.0	0.0	0.0	0.0
Demequinaceae (1)	0.0	0.0	0.0	0.0	0.0	0.0	0.0	0.0	0.0	0.0	0.0	0.0
Frankiaceae (3)	0.1	0.1	0.1	0.1	0.0	0.0	0.0	0.0	0.0	0.0	0.1	0.0
Geodermatophilaceae (1)	0.0	0.0	0.0	0.0	0.0	0.0	0.0	0.0	0.0	0.0	0.0	0.0
Intrasporangiaceae (3)	0.2	0.2	0.2	0.2	0.1	0.1	0.1	0.1	0.1	0.1	0.2	0.1
Kineosporiaceae (1)	0.0	0.0	0.0	0.0	0.0	0.0	0.0	0.0	0.0	0.0	0.0	0.0
Microbacteriaceae (6)	0.2	0.3	0.3	0.2	0.1	0.0	0.1	0.1	0.1	0.1	0.1	0.1
Micrococcaceae (1)	0.4	0.5	0.5	0.3	0.2	0.1	0.2	0.1	0.1	0.1	0.1	0.1

Sampling Time:	0 h				10 h		22 h		30 h			
**Treatment:**	**C**	**G**	**F**	**S**	**C**	**G**	**C**	**G**	**C**	**G**	**F**	**S**
**Phyla, Class, Family[b]**	**Relative Abundance (%)**											
Micromonosporaceae (18)	0.9	1.0	1.1	0.6	0.5	0.1	0.5	0.3	0.5	0.4	0.6	0.3
Mycobacteriaceae (5)	0.6	0.7	0.7	0.5	0.3	0.1	0.3	0.2	0.3	0.2	0.4	0.2
Nakamurellaceae (2)	0.0	0.1	0.0	0.0	0.0	0.0	0.0	0.0	0.0	0.0	0.0	0.0
Nocardiaceae (6)	0.0	0.0	0.0	0.0	0.0	0.0	0.0	0.0	0.0	0.0	0.0	0.0
Nocardioidaceae (25)	1.6	1.6	1.7	1.4	0.7	0.3	0.7	0.4	0.8	0.5	0.9	0.6
Promicromonosporaceae (2)	0.1	0.1	0.1	0.0	0.0	0.0	0.0	0.0	0.1	0.0	0.1	0.0
Propionibacteriaceae (6)	0.5	0.4	0.7	0.4	0.3	0.1	0.3	0.1	0.2	0.2	0.3	0.1
Pseudonocardiaceae (10)	0.5	0.6	0.6	0.5	0.4	0.1	0.3	0.2	0.3	0.3	0.4	0.2
Sporichthyaceae (3)	0.1	0.1	0.1	0.1	0.0	0.0	0.0	0.0	0.0	0.0	0.1	0.0
Streptomycetaceae (4)	0.7	0.6	0.6	0.4	0.4	0.1	0.3	0.1	0.3	0.2	0.4	0.2
Streptosporangiaceae (3)	0.0	0.0	0.0	0.1	0.0	0.0	0.1	0.0	0.0	0.0	0.0	0.1
Thermomonosporaceae (4)	0.0	0.1	0.0	0.0	0.0	0.0	0.0	0.0	0.0	0.0	0.0	0.0
Unassigned *Frankiales* (1)	0.0	0.0	0.0	0.0	0.0	0.0	0.0	0.0	0.0	0.0	0.0	0.0
Unassigned *Actinobacteria* (4)	0.1	0.1	0.0	0.0	0.0	0.0	0.0	0.0	0.0	0.0	0.1	0.0
Coriobacteriia,												
Coriobacteriaceae (1)	0.0	0.0	0.0	0.0	0.0	0.0	0.0	0.0	0.0	0.0	0.0	0.0
Rubrobacteria,												
Rubrobacteriaceae (4)	0.2	0.3	0.2	0.1	0.1	0.0	0.1	0.1	0.1	0.1	0.1	0.1
Thermoleophilia,												
Gaiellaceae (7)	2.6	2.1	1.9	1.9	1.2	0.5	1.1	0.6	1.2	0.8	1.4	0.8
Parviterribacteraceae (1)	0.0	0.0	0.0	0.0	0.0	0.0	0.0	0.0	0.0	0.0	0.0	0.0
Patulibacteraceae (4)	0.0	0.0	0.0	0.0	0.0	0.0	0.0	0.0	0.0	0.0	0.0	0.0
Solirubrobacteraceae (4)	0.4	0.5	0.5	0.4	0.4	0.1	0.3	0.2	0.3	0.3	0.5	0.3
Unassigned *Solirubrobacterales* (16)	0.9	0.8	0.8	0.7	0.6	0.2	0.3	0.3	0.5	0.3	0.7	0.4
Unassigned *Gaiellales* (27)	4.6	3.9	3.7	3.3	2.5	0.8	1.7	1.2	1.9	1.3	2.6	1.6
Unassigned *Thermoleophilia* (36)	2.2	1.8	1.6	1.3	1.2	0.5	0.9	0.5	1.0	0.7	1.2	0.7
Unassigned *Actinobacteria* (23)	2.5	2.7	2.6	1.9	1.4	0.4	1.3	0.5	1.1	0.8	1.2	0.9
Armatimonadetes,												
Unassigned *Armatimonadetes* (1)	0.0	0.0	0.0	0.0	0.0	0.0	0.0	0.0	0.0	0.0	0.0	0.0
Bacteroidetes,												
Bacteroidia,												
Bacteroidaceae (5)	0.0	0.0	0.0	0.0	0.0	0.0	0.0	0.0	0.0	0.2	0.0	0.6
Porphyromonadaceae (2)	0.0	0.0	0.0	0.0	0.0	0.0	0.0	0.0	0.0	0.0	0.0	0.0
Prevotellaceae (1)	0.0	0.0	0.0	0.0	0.0	0.0	0.0	0.0	0.0	0.0	0.0	0.0
Rikenellaceae (1)	0.0	0.0	0.0	0.0	0.0	0.0	0.0	0.0	0.0	0.0	0.0	0.0
Sphingobacteriia												
Chitinophagaceae (9)	0.0	0.0	0.1	0.1	0.0	0.0	0.0	0.0	0.0	0.0	0.0	0.0
Cytophagia,												
Cytophagaceae (11)	0.1	0.1	0.1	0.0	0.0	0.0	0.0	0.0	0.0	0.0	0.0	0.0
Flavobacteriia,												
Flavobacteriaceae (7)	0.7	0.9	0.7	0.6	0.4	0.1	0.3	0.1	0.2	0.0	0.1	0.1
Sphingobacteriia,												
Saprospiraceae (1)	0.0	0.0	0.0	0.0	0.0	0.0	0.0	0.0	0.0	0.0	0.0	0.0
Sphingobacteriaceae (1)	0.0	0.0	0.0	0.0	0.0	0.0	0.0	0.0	0.0	0.0	0.0	0.0

Sampling Time:	0 h				10 h		22 h		30 h			
Treatment:	C	G	F	S	C	G	C	G	C	G	F	S
Phyla, Class, Family[b]	Relative Abundance (%)											
Unassigned *Sphingobacteriales* (3)	0.0	0.0	0.0	0.0	0.0	0.0	0.0	0.0	0.0	0.0	0.0	0.0
BRC1,												
Unassigend BRC1 (1)	0.0	0.0	0.0	0.0	0.0	0.0	0.0	0.0	0.0	0.0	0.0	0.0
Chlamydiae,												
Chlamydiae,												
Chlamydiaceae (1)	0.0	0.0	0.0	0.0	0.0	0.0	0.0	0.0	0.0	0.0	0.0	0.0
Parachlamydiaceae (13)	0.0	0.0	0.0	0.0	0.0	0.0	0.0	0.0	0.0	0.0	0.0	0.0
Chlorobi,												
Chlorobiales,												
Unassigned *Chlorobiales* (1)	0.0	0.0	0.0	0.0	0.0	0.0	0.0	0.0	0.0	0.0	0.0	0.0
Chloroflexi,,												
Anaerolineae												
Anaerolineaceae (5)	0.0	0.0	0.0	0.0	0.0	0.0	0.0	0.0	0.0	0.0	0.0	0.0
Unassigned *Ardenticatenia* (2)	0.0	0.0	0.0	0.0	0.0	0.0	0.0	0.0	0.0	0.0	0.0	0.0
Caldilineae,												
Caldilineaceae (11)	0.3	0.2	0.2	0.2	0.1	0.0	0.1	0.1	0.1	0.1	0.1	0.1
Chloroflexia,												
Unassigned *Kallotenuales* (1)	0.0	0.0	0.0	0.0	0.0	0.0	0.0	0.0	0.0	0.0	0.0	0.0
Roseiflexaceae (6)	0.2	0.3	0.2	0.2	0.1	0.0	0.1	0.1	0.1	0.1	0.1	0.1
Ktedonobacteria,												
Ktedonobacteraceae (2)	0.0	0.0	0.0	0.0	0.0	0.0	0.0	0.0	0.0	0.0	0.0	0.0
Thermosporotrichaceae (1)	0.0	0.0	0.0	0.0	0.0	0.0	0.0	0.0	0.0	0.0	0.0	0.0
Unassigned *Ktedonobacterales* (1)	0.0	0.0	0.0	0.0	0.0	0.0	0.0	0.0	0.0	0.0	0.0	0.0
Unassigned *Ktedonobacteria* (5)	0.0	0.1	0.0	0.0	0.0	0.0	0.0	0.0	0.0	0.0	0.0	0.0
Unassigned *Thermomicrobia* (28)	0.5	0.6	0.5	0.3	0.2	0.1	0.3	0.1	0.3	0.2	0.3	0.2
Unassigned *Chloroflexi* (41)	2.9	2.3	2.8	2.1	1.4	0.5	1.4	0.5	1.4	0.8	1.6	1.1
Cyanobacteria,												
Chloroplast,												
Unassigned *Chloroplast* (5)	0.1	0.0	0.0	0.0	0.0	0.0	0.0	0.0	0.0	0.0	0.0	0.0
Cyanobacteria,												
Unassigned *Cyanobacteria* (2)	0.0	0.0	0.0	0.0	0.0	0.0	0.0	0.0	0.0	0.0	0.0	0.0
Unassigned *Cyanobacteria* (2)	0.0	0.0	0.0	0.0	0.0	0.0	0.0	0.0	0.0	0.0	0.0	0.0
Elusimicrobia,												
Unassigned *Elusimicrobia* (1)	0.0	0.0	0.0	0.0	0.0	0.0	0.0	0.0	0.0	0.0	0.0	0.0
Fibrobacteres,												
Fibrobacteria,												
Fibrobacteraceae (2)	0.0	0.0	0.0	0.0	0.0	0.0	0.0	0.0	0.0	0.0	0.0	0.0
Firmicutes,												
Bacilli,												
Aerococcaceae (1)	0.0	0.0	0.0	0.0	0.0	0.0	0.0	0.0	0.0	0.0	0.0	0.0
Alicyclobacillaceae (1)	0.0	0.0	0.0	0.0	0.0	0.0	0.0	0.0	0.0	0.0	0.0	0.0

Sampling Time:	0 h				10 h		22 h		30 h			
Treatment:	C	G	F	S	C	G	C	G	C	G	F	S
**Phyla, Class, Family[b]**	**Relative Abundance (%)**											
Bacillaceae (9)	1.3	1.3	0.8	0.9	0.8	0.4	1.1	0.8	1.8	1.5	2.0	1.1
Enterococcaceae (1)	0.0	0.0	0.0	0.4	0.0	0.0	0.0	0.0	0.0	0.0	0.0	0.0
Lactobacillaceae (2)	0.1	0.0	0.0	0.0	0.0	0.0	0.0	0.1	0.0	0.1	0.0	0.0
Paenibacillaceae (22)	0.2	0.1	0.1	0.1	0.0	0.0	0.2	0.1	0.3	0.2	0.2	0.2
Planococcaceae (7)	0.1	0.1	0.1	0.1	0.1	0.0	0.0	0.0	0.1	0.0	0.1	0.1
Staphylococcaceae (1)	0.0	0.0	0.0	0.0	0.0	0.0	0.0	0.0	0.0	0.0	0.0	0.0
Thermoactinomycetaceae (3)	0.0	0.0	0.0	0.0	0.0	0.0	0.0	0.0	0.0	0.0	0.0	0.0
Streptococcaceae (3)	0.0	0.0	0.0	0.0	0.0	0.1	0.0	0.0	0.1	0.0	0.0	0.0
Unassigned *Lactobacillales* (1)	0.0	0.0	0.0	0.0	0.1	0.0	0.0	0.0	0.0	0.1	0.0	0.1
Unassigned *Bacilli* (2)	0.1	0.1	0.1	0.2	0.1	0.0	0.0	0.0	0.0	0.0	0.0	0.0
Clostridia,												
Clostridiaceae (22) **[A14]**	0.4	0.4	0.4	0.2	0.5	0.2	1.7	1.0	2.5	2.5	2.5	2.1
Unassigned *Clostridiales* (7)	0.0	0.0	0.0	0.0	0.0	0.0	0.0	0.0	0.2	0.0	0.0	0.1
Eubacteriaceae (1)	0.0	0.0	0.0	0.0	0.0	0.0	0.0	0.0	0.0	0.0	0.0	0.0
Gracilibacteraceae (1)	0.0	0.0	0.0	0.0	0.0	0.0	0.0	0.0	0.0	0.0	0.0	0.0
Heliobacteriaceae (3)	0.0	0.0	0.0	0.0	0.0	0.0	0.0	0.0	0.0	0.0	0.0	0.0
Lachnospiraceae (24)	0.1	0.0	0.1	0.0	0.0	0.0	0.9	0.3	4.9	2.7	5.7	6.0
Peptococcaceae (5)	1.0	1.2	0.8	0.9	1.1	0.5	0.7	0.5	0.9	0.8	0.9	0.6
Peptostreptococcaceae (6) **[GPT-4], [A8]**	0.2	0.1	0.2	0.2	0.8	0.1	2.5	1.0	2.9	2.6	5.1	1.9
Ruminococcaceae (28)	0.1	0.1	0.0	0.0	0.0	0.0	0.0	0.0	0.1	0.2	0.0	1.6
Erysipelotrichia,												
Erysipelotrichaceae (6)	0.4	0.0	0.0	0.0	0.0	0.0	0.0	0.7	0.0	0.0	0.0	0.1
Limnochordia,												
Unassigned *Limnochordales* (1)	0.0	0.0	0.0	0.0	0.0	0.0	0.0	0.0	0.0	0.0	0.0	0.0
Negativicutes,												
Acidaminococcaceae (1)	0.0	0.0	0.0	0.0	0.0	0.0	0.0	0.0	0.0	0.0	0.0	0.0
Veillonellaceae (9)	0.0	0.0	0.0	0.0	0.0	0.0	0.0	0.0	0.0	0.0	0.0	0.0
Fusobacteria,												
Fusobacteriia,												
Fusobacteriaceae (1) [**GPT-5]**	1.8	2.8	3.0	4.2	20.3	11.8	29.4	22.7	21.6	21.1	16.7	35.3
Gemmatimonadetes,												
Gemmatimonadaceae (15)	0.3	0.3	0.4	0.2	0.2	0.1	0.1	0.1	0.1	0.1	0.2	0.1
Unassigned *Gemmatimonadetes* (4)	0.0	0.0	0.0	0.0	0.0	0.0	0.0	0.0	0.0	0.0	0.0	0.0
Latescibacteria,												
Unassigned *Latescibacteria* (8)	0.0	0.0	0.0	0.0	0.0	0.0	0.0	0.0	0.0	0.0	0.0	0.0
Nitrospirae,												
Nitrospira,												
Nitrospiraceae (5)	0.1	0.1	0.1	0.1	0.1	0.0	0.0	0.0	0.0	0.0	0.1	0.0
Unassigned *Nitrospirales* (1)	0.0	0.0	0.0	0.0	0.0	0.0	0.0	0.0	0.0	0.0	0.0	0.0
Unassigned *Nitrospira* (12)	0.7	0.9	0.8	0.7	0.5	0.1	0.4	0.1	0.4	0.3	0.4	0.3
Planctomycetes,												
Phycisphaerae,												
Phycisphaeraceae (9)	0.0	0.0	0.0	0.0	0.0	0.0	0.0	0.0	0.0	0.0	0.0	0.0

Sampling Time:	0 h				10 h		22 h		30 h			
Treatment:	C	G	F	S	C	G	C	G	C	G	F	S
**Phyla, Class, Family**[b]	Relative Abundance (%)											
Tepidisphaeraceae (40)	0.9	0.9	1.1	0.6	0.4	0.1	0.4	0.2	0.3	0.3	0.3	0.2
Unassigned *Phycisphaerae* (4)	0.0	0.0	0.0	0.0	0.0	0.0	0.0	0.0	0.0	0.0	0.0	0.0
Planctomycetacia,												
Planctomycetaceae (400)	3.8	3.9	3.7	2.7	2.4	0.7	2.0	1.0	2.1	1.6	1.9	1.3
Unassigned *Planctomycetes* (23)	0.1	0.1	0.1	0.1	0.0	0.0	0.0	0.0	0.0	0.0	0.1	0.0
Proteobacteria,												
Alphaproteobacteria												
Acetobacteraceae (6)	0.0	0.0	0.0	0.0	0.0	0.0	0.0	0.0	0.0	0.0	0.0	0.0
Beijerinckiaceae (1)	0.0	0.0	0.0	0.0	0.0	0.0	0.0	0.0	0.0	0.0	0.0	0.0
Bradyrhizobiaceae (2)	0.7	0.9	0.8	0.6	0.4	0.1	0.4	0.1	0.4	0.2	0.3	0.2
Caulobacteraceae (1)	0.0	0.0	0.0	0.0	0.0	0.0	0.0	0.0	0.0	0.0	0.0	0.0
Erythrobacteraceae (1)	0.0	0.0	0.0	0.0	0.0	0.0	0.0	0.0	0.0	0.0	0.0	0.0
Holosporaceae (1)	0.0	0.0	0.0	0.0	0.0	0.0	0.0	0.0	0.0	0.0	0.0	0.0
Hyphomicrobiaceae (6)	0.9	0.8	0.8	0.7	0.4	0.2	0.4	0.2	0.4	0.2	0.4	0.4
Methylobacteriaceae (3)	0.1	0.1	0.2	0.2	0.1	0.0	0.1	0.1	0.1	0.1	0.1	0.1
Phyllobacteriaceae (3)	0.4	0.3	0.4	0.3	0.2	0.0	0.1	0.1	0.2	0.1	0.1	0.2
Rhizobiaceae (2)	0.1	0.0	0.0	0.0	0.0	0.0	0.0	0.0	0.0	0.0	0.0	0.0
Unassigned *Rhizobiales* (9)	0.3	0.3	0.3	0.2	0.1	0.0	0.1	0.1	0.1	0.0	0.1	0.1
Rhodobacteraceae (5)	0.1	0.1	0.1	0.1	0.0	0.0	0.0	0.0	0.0	0.0	0.0	0.1
Rhodobiaceae (2)	1.9	1.6	1.7	1.1	0.8	0.3	0.7	0.2	0.7	0.4	0.9	0.6
Rhodospirillaceae (12)	0.6	1.0	1.0	0.5	0.4	0.1	0.4	0.2	0.3	0.2	0.4	0.3
Unassigned *Rhodospirillales* (13)	0.6	0.5	0.7	0.4	0.3	0.1	0.3	0.1	0.3	0.2	0.3	0.2
Sphingomonadaceae (1)	0.0	0.0	0.0	0.0	0.0	0.0	0.0	0.0	0.0	0.0	0.0	0.0
Unassigned *Sphingomonadales* (1)	0.0	0.0	0.0	0.0	0.0	0.0	0.0	0.0	0.0	0.0	0.0	0.0
Xanthobacteraceae (4)	2.8	2.7	2.5	2.0	1.3	0.4	1.2	0.6	1.0	0.6	1.1	1.0
Unassigned *Rickettsiales* (2)	0.0	0.0	0.0	0.0	0.0	0.0	0.0	0.0	0.0	0.0	0.0	0.0
Unassigned *Alphaproteobacteria* (12)	0.1	0.1	0.2	0.1	0.1	0.0	0.1	0.0	0.1	0.0	0.1	0.0
Betaproteobacteria,												
Alcaligenaceae (2)	0.2	0.1	0.1	0.1	0.1	0.0	0.1	0.0	0.1	0.0	0.1	0.0
Burkholderiaceae (4)	0.0	0.0	0.0	0.0	0.0	0.0	0.0	0.0	0.0	0.0	0.0	0.0
Comamonadaceae (9)	0.1	0.1	0.1	0.0	0.0	0.0	0.0	0.0	0.0	0.0	0.0	0.0
Oxalobacteraceae (1)	0.0	0.0	0.0	0.0	0.0	0.0	0.0	0.0	0.0	0.0	0.0	0.0
Neisseriaceae (1)	0.0	0.0	0.0	0.0	0.0	0.0	0.0	0.0	0.0	0.0	0.0	0.0
Nitrosomonadaceae (17)	0.3	0.4	0.4	0.3	0.2	0.1	0.2	0.1	0.1	0.1	0.1	0.2
Rhodocyclaceae (3)	0.0	0.0	0.0	0.0	0.0	0.0	0.0	0.0	0.0	0.0	0.0	0.0
Unassigned *Betaproteobacteria* (20)	0.3	0.3	0.2	0.2	0.1	0.1	0.1	0.0	0.1	0.0	0.1	0.1
Deltaproteobacteria,												
Bdellovibrionaceae (9)	0.0	0.0	0.0	0.0	0.0	0.0	0.0	0.0	0.0	0.0	0.0	0.0
Desulfovibrionaceae (1)	0.0	0.0	0.0	0.0	0.0	0.0	0.0	0.0	0.0	0.0	0.0	0.1
Desulfurellaceae (21)	1.3	1.1	1.3	1.0	0.6	0.2	0.6	0.3	0.5	0.4	0.6	0.3
Desulfuromonadaceae (2)	0.0	0.0	0.0	0.0	0.0	0.0	0.0	0.0	0.0	0.0	0.0	0.0
Geobacteraceae (8)	0.1	0.1	0.1	0.1	0.0	0.0	0.0	0.0	0.0	0.0	0.0	0.0
Archangiaceae (5)	0.1	0.1	0.1	0.1	0.0	0.0	0.0	0.0	0.0	0.0	0.1	0.0
Haliangiaceae (32)	0.2	0.2	0.2	0.1	0.2	0.0	0.1	0.0	0.1	0.1	0.1	0.1

Sampling Time:	0 h				10 h		22 h		30 h			
Treatment:	C	G	F	S	C	G	C	G	C	G	F	S
**Phyla, Class, Family[b]**	**Relative Abundance (%)**											
Myxococcaceae (2)	0.0	0.0	0.0	0.0	0.0	0.0	0.0	0.0	0.0	0.0	0.0	0.0
Unassigned *Myxococcales* (45)	0.2	0.2	0.2	0.1	0.1	0.0	0.1	0.0	0.1	0.0	0.1	0.1
Nannocystaceae (4)	0.0	0.0	0.0	0.0	0.0	0.0	0.0	0.0	0.0	0.0	0.0	0.0
Phaselicystidaceae (3)	0.1	0.0	0.0	0.0	0.0	0.0	0.0	0.0	0.0	0.0	0.0	0.0
Polyangiaceae (18)	0.1	0.1	0.1	0.1	0.1	0.0	0.0	0.0	0.0	0.0	0.1	0.0
Sandaracinaceae (19)	0.1	0.1	0.1	0.1	0.1	0.0	0.0	0.0	0.1	0.0	0.0	0.0
Vulgatibacteraceae (1)	0.0	0.0	0.0	0.0	0.0	0.0	0.0	0.0	0.0	0.0	0.0	0.0
Oligoflexaceae (1)	0.0	0.0	0.0	0.0	0.0	0.0	0.0	0.0	0.0	0.0	0.0	0.0
Unassigned *Oligoflexales* (6)	0.0	0.0	0.0	0.0	0.0	0.0	0.0	0.0	0.0	0.0	0.0	0.0
Unassigned *Deltaproteobacteria* (8)	0.0	0.1	0.1	0.0	0.0	0.0	0.0	0.0	0.0	0.0	0.0	0.0
Epsilonproteobacteria,												
Campylobacteraceae (1)	0.0	0.0	0.0	0.0	0.0	0.0	0.0	0.0	0.0	0.0	0.0	0.0
Gammaproteobacteria												
Aeromonadaceae (2) **[GPT-1]**	17	15	20	29	26	67	17	48	16	35	15	12
Coxiellaceae (2)	0.1	0.1	0.1	0.1	0.1	0.0	0.1	0.0	0.0	0.0	0.0	0.0
Enterobacteriaceae (6) **[GPT-2], [GPT-3]**	1.6	1.8	2.5	3.0	3.2	2.4	3.4	2.6	3.1	2.2	3.3	3.0
Halieaceae (1)	0.0	0.0	0.0	0.0	0.0	0.0	0.0	0.0	0.0	0.0	0.0	0.0
Legionellaceae (3)	0.0	0.0	0.0	0.0	0.0	0.0	0.0	0.0	0.0	0.0	0.0	0.0
Pseudomonadaceae (2)	0.0	0.0	0.0	0.0	0.0	0.0	0.0	0.0	0.0	0.0	0.0	0.0
Shewanellaceae (2)	3.1	3.8	3.6	4.2	6.9	2.3	8.6	3.6	9.2	4.6	8.9	6.7
Xanthomonadaceae (3)	0.0	0.0	0.0	0.0	0.0	0.0	0.0	0.0	0.0	0.0	0.0	0.0
Unassigned *Xanthomonadales* (14)	0.3	0.2	0.3	0.2	0.2	0.0	0.1	0.0	0.1	0.1	0.1	0.1
Unassigned *Gammaproteobacteria* (3)	0.0	0.0	0.0	0.0	0.0	0.0	0.0	0.0	0.0	0.0	0.0	0.0
Saccharibacteria,												
Unassigned *Saccharibacteria* (8)	0.0	0.0	0.0	0.0	0.0	0.0	0.0	0.0	0.0	0.0	0.0	0.0
Spirochaetae,												
Spirochaetes,,												
Spirochaetaceae (1)	0.0	0.0	0.0	0.0	0.0	0.0	0.0	0.0	0.0	0.0	0.0	0.0
Tectomicrobia												
Unassigned *Tectomicrobia* (16)	0.4	0.4	0.4	0.3	0.3	0.1	0.2	0.1	0.2	0.1	0.1	0.1
Tenericutes,												
Mollicutes,												
Mycoplasmataceae (6)	18	20	16	16	11	5.1	9.0	6.2	11	8.8	11	9.6
Unassigned *Entomoplasmatales* (3)	0.1	0.2	0.1	0.1	0.0	0.0	0.0	0.0	0.1	0.0	0.0	0.0
Thaumarchaeota,												
Unassigned *Thaumarchaeota* (5)	0.0	0.0	0.0	0.0	0.0	0.0	0.0	0.0	0.0	0.0	0.0	0.0
TM*6*_Dependentiae,												
Unassigned *TM*6*_Dependentiae* (1)	0.0	0.0	0.0	0.0	0.0	0.0	0.0	0.0	0.0	0.0	0.0	0.0
Verrucomicrobia,												
Unassigned *OPB*35 *soil group* (29)	0.2	0.2	0.2	0.2	0.1	0.0	0.1	0.0	0.0	0.0	0.0	0.0
Spartobacteria,												
Chthoniobacteraceae (16)	0.1	0.1	0.1	0.1	0.0	0.0	0.0	0.0	0.0	0.0	0.0	0.0

Sampling Time:	0 h				10 h		22 h		30 h			
Treatment:	C	G	F	S	C	G	C	G	C	G	F	S
**Phyla, Class, Family**[b]	**Relative Abundance (%)**											
Xiphinematobacteraceae (3)	1.8	2.0	2.1	1.6	1.0	0.3	1.3	0.5	1.1	0.9	0.9	0.7
Unassigned *Chthoniobacterales* (19)	1.7	1.4	1.2	1.1	0.8	0.3	0.7	0.3	0.8	0.5	0.6	0.5
Opitutae,												
Opitutaceae (2)	0.0	0.0	0.0	0.0	0.0	0.0	0.0	0.0	0.0	0.0	0.0	0.0
Unassigned *Opitutae* (1)	0.0	0.0	0.0	0.0	0.0	0.0	0.0	0.0	0.0	0.0	0.0	0.0
Verrucomicrobiae,												
Verrucomicrobiaceae (7)	0.0	0.1	0.0	0.0	0.0	0.0	0.0	0.0	0.0	0.1	0.0	0.1

(B) 16S rRNA

Sampling Time:	0 h						10 h		22 h		30 h											
Treatment:	C1	C2	C3	G	F	S	C	G	C	G	C1	C2	C3	G1	G2	G3	F1	F2	F3	S1	S2	S3
**Phyla, Class, Family**[b]	**Relative Abundance (%)**																					
Acidobacteria,																						
Acidobacteria,																						
Acidobacteriaceae (3)	0.0	0.0	0.0	0.0	0.0	0.0	0.0	0.0	0.0	0.0	0.0	0.0	0.0	0.0	0.0	0.0	0.0	0.0	0.0	0.0	0.0	0.0
Blastocatellia,																						
Blastocatellaceae (9)	0.1	0.1	0.1	0.1	0.1	0.1	0.1	0.0	0.1	0.1	0.0	0.1	0.0	0.0	0.0	0.0	0.0	0.0	0.1	0.1	0.1	0.1
Holophagae,																						
Unassigned *Holophagae* (4)	0.0	0.0	0.0	0.0	0.1	0.0	0.0	0.0	0.0	0.0	0.0	0.0	0.0	0.0	0.0	0.0	0.0	0.0	0.0	0.0	0.0	0.0
Solibacteres,																						
Solibacteraceae (10)	0.1	0.2	0.2	0.1	0.1	0.1	0.1	0.0	0.1	0.0	0.1	0.1	0.0	0.0	0.0	0.0	0.0	0.0	0.0	0.1	0.1	0.1
Subgroup_11 (3)	0.0	0.0	0.0	0.0	0.0	0.0	0.0	0.0	0.0	0.0	0.0	0.0	0.0	0.0	0.0	0.0	0.0	0.0	0.0	0.0	0.0	0.0
Subgroup_17 (9)	0.0	0.0	0.0	0.0	0.0	0.1	0.0	0.0	0.0	0.0	0.0	0.0	0.0	0.0	0.0	0.0	0.0	0.0	0.0	0.0	0.0	0.0
Subgroup_18 (1)	0.0	0.0	0.1	0.0	0.0	0.0	0.0	0.0	0.0	0.0	0.0	0.0	0.0	0.0	0.0	0.0	0.0	0.0	0.0	0.0	0.0	0.0
Subgroup_22 (5)	0.0	0.0	0.0	0.0	0.0	0.0	0.0	0.0	0.0	0.0	0.0	0.0	0.0	0.0	0.0	0.0	0.0	0.0	0.0	0.0	0.0	0.0
Subgroup_25 (3)	0.0	0.0	0.0	0.0	0.0	0.0	0.0	0.0	0.0	0.0	0.0	0.0	0.0	0.0	0.0	0.0	0.0	0.0	0.0	0.0	0.0	0.0
Subgroup_5 (6)	0.0	0.0	0.0	0.1	0.0	0.0	0.0	0.0	0.0	0.0	0.0	0.0	0.0	0.0	0.0	0.0	0.0	0.0	0.0	0.0	0.0	0.0
Subgroup_6 (49)	0.8	0.5	0.6	0.7	0.8	0.6	0.3	0.1	0.1	0.1	0.1	0.1	0.1	0.1	0.1	0.2	0.1	0.1	0.1	0.2	0.1	0.1
Subgroup_9 (1)	0.0	0.0	0.0	0.0	0.0	0.0	0.0	0.0	0.0	0.0	0.0	0.0	0.0	0.0	0.0	0.0	0.0	0.0	0.0	0.0	0.0	0.0
Actinobacteria,																						
Acidimicrobiia,																						
Acidimicrobiaceae (17)	1.3	1.5	1.2	1.2	1.2	1.1	0.7	0.3	0.7	0.3	0.8	0.7	0.6	0.3	0.4	0.4	0.9	0.8	0.7	0.6	0.6	0.7
Iamiaceae (8)	0.1	0.1	0.3	0.2	0.1	0.2	0.1	0.0	0.2	0.1	0.1	0.1	0.1	0.0	0.0	0.0	0.1	0.0	0.1	0.0	0.0	0.1
Unassigned *Acidimicrobiales* (39)	1.8	1.6	1.9	1.7	1.6	1.5	1.0	0.5	0.9	0.4	1.0	0.9	0.8	0.3	0.6	0.4	0.7	0.9	0.9	1.1	0.8	0.6
Actinobacteria,																						
Acidothermaceae (4)	0.0	0.0	0.0	0.1	0.1	0.0	0.0	0.0	0.0	0.0	0.0	0.0	0.0	0.0	0.0	0.0	0.0	0.0	0.0	0.0	0.1	0.1
Actinomycetaceae (1)	0.0	0.0	0.0	0.0	0.0	0.0	0.0	0.0	0.0	0.0	0.0	0.0	0.0	0.0	0.0	0.0	0.0	0.0	0.0	0.0	0.0	0.0
Bifidobacteriaceae (3)	0.0	0.0	0.0	0.0	0.0	0.1	0.0	0.0	0.0	0.0	0.0	0.0	0.0	0.0	0.0	0.0	0.0	0.0	0.0	0.0	0.0	0.0
Bogoriellaceae (1)	0.0	0.0	0.0	0.0	0.0	0.0	0.0	0.0	0.0	0.0	0.0	0.0	0.0	0.0	0.0	0.0	0.0	0.0	0.0	0.0	0.0	0.0

Sampling Time:	0 h						10 h		22 h		30 h											
Treatment:	C1	C2	C3	G	F	S	C	G	C	G	C1	C2	C3	G1	G2	G3	F1	F2	F3	S1	S2	S3
**Phyla, Class, Family[b]**	**Relative Abundance (%)**																					
Cellulomonadaceae (1)	0.2	0.2	0.1	0.2	0.2	0.1	0.1	0.0	0.0	0.0	0.1	0.1	0.1	0.0	0.0	0.0	0.1	0.1	0.1	0.1	0.1	0.1
Corynebacteriaceae (1)	0.0	0.0	0.0	0.0	0.0	0.0	0.0	0.0	0.0	0.0	0.0	0.0	0.0	0.0	0.0	0.0	0.0	0.0	0.0	0.0	0.0	0.0
Cryptosporangiaceae (2)	0.0	0.0	0.0	0.0	0.0	0.0	0.0	0.0	0.0	0.0	0.0	0.0	0.0	0.0	0.0	0.0	0.0	0.0	0.0	0.0	0.0	0.0
Demequinaceae (1)	0.0	0.0	0.0	0.0	0.0	0.0	0.0	0.0	0.0	0.0	0.0	0.0	0.0	0.0	0.0	0.0	0.0	0.0	0.0	0.0	0.0	0.0
Frankiaceae (3)	0.1	0.1	0.1	0.1	0.1	0.1	0.0	0.0	0.0	0.0	0.1	0.0	0.0	0.0	0.0	0.0	0.0	0.0	0.1	0.0	0.0	0.1
Geodermatophilaceae (1)	0.0	0.0	0.0	0.0	0.0	0.0	0.0	0.0	0.0	0.0	0.0	0.0	0.0	0.0	0.0	0.0	0.0	0.0	0.0	0.0	0.0	0.0
Intrasporangiaceae (3)	0.1	0.1	0.1	0.2	0.1	0.1	0.1	0.0	0.0	0.1	0.1	0.1	0.1	0.0	0.1	0.0	0.1	0.1	0.0	0.1	0.1	0.1
Kineosporiaceae (1)	0.0	0.0	0.0	0.0	0.0	0.0	0.0	0.0	0.0	0.0	0.0	0.0	0.0	0.0	0.0	0.0	0.0	0.0	0.0	0.0	0.0	0.0
Microbacteriaceae (6)	0.1	0.1	0.2	0.2	0.2	0.1	0.1	0.1	0.0	0.0	0.1	0.1	0.0	0.0	0.0	0.1	0.0	0.1	0.1	0.1	0.0	0.0
Micrococcaceae (1)	0.1	0.1	0.1	0.2	0.2	0.2	0.1	0.0	0.1	0.0	0.0	0.0	0.0	0.0	0.0	0.0	0.1	0.0	0.1	0.1	0.0	0.1
Micromonosporaceae (18)	0.7	0.9	0.9	1.2	0.8	0.8	0.6	0.2	0.4	0.2	0.5	0.4	0.5	0.2	0.3	0.3	0.8	0.4	0.5	0.5	0.4	0.5
Mycobacteriaceae (5)	0.2	0.1	0.1	0.2	0.2	0.2	0.1	0.0	0.1	0.0	0.1	0.1	0.1	0.0	0.0	0.0	0.1	0.1	0.1	0.1	0.1	0.1
Nakamurellaceae (2)	0.1	0.1	0.1	0.0	0.1	0.1	0.0	0.0	0.0	0.0	0.0	0.0	0.0	0.0	0.0	0.0	0.0	0.0	0.0	0.1	0.0	0.0
Nocardiaceae (6)	0.0	0.0	0.1	0.1	0.1	0.0	0.0	0.0	0.0	0.0	0.0	0.0	0.0	0.0	0.0	0.0	0.0	0.0	0.0	0.0	0.0	0.0
Nocardioidaceae (25)	1.1	0.8	1.0	0.9	0.8	0.7	0.4	0.2	0.4	0.2	0.4	0.5	0.4	0.2	0.2	0.2	0.7	0.4	0.5	0.5	0.4	0.5
Promicromonosporaceae (2)	0.1	0.1	0.1	0.1	0.2	0.1	0.1	0.0	0.1	0.1	0.1	0.1	0.1	0.0	0.1	0.0	0.0	0.1	0.1	0.2	0.1	0.1
Propionibacteriaceae (6)	0.2	0.2	0.1	0.3	0.2	0.3	0.2	0.1	0.1	0.1	0.2	0.1	0.2	0.0	0.0	0.0	0.1	0.1	0.1	0.2	0.1	0.1
Pseudonocardiaceae (10)	0.5	0.5	0.6	0.7	0.7	0.6	0.4	0.1	0.4	0.2	0.4	0.3	0.2	0.2	0.2	0.1	0.5	0.4	0.3	0.4	0.2	0.4
Sporichthyaceae (3)	0.0	0.0	0.0	0.0	0.1	0.0	0.0	0.0	0.0	0.0	0.0	0.0	0.0	0.0	0.0	0.0	0.0	0.0	0.1	0.0	0.0	0.0
Streptomycetaceae (4)	0.4	0.3	0.3	0.3	0.5	0.3	0.2	0.1	0.2	0.1	0.2	0.2	0.2	0.1	0.1	0.1	0.3	0.3	0.2	0.3	0.2	0.2
Streptosporangiaceae (3)	0.0	0.0	0.0	0.0	0.0	0.0	0.0	0.0	0.0	0.0	0.0	0.0	0.0	0.0	0.0	0.0	0.0	0.0	0.0	0.0	0.0	0.0
Thermomonosporaceae (4)	0.0	0.0	0.0	0.0	0.0	0.0	0.0	0.0	0.0	0.0	0.0	0.0	0.0	0.0	0.0	0.0	0.0	0.0	0.0	0.0	0.0	0.0
Unassigned *Frankiales* (1)	0.0	0.0	0.0	0.0	0.0	0.0	0.0	0.0	0.0	0.0	0.0	0.0	0.0	0.0	0.0	0.0	0.0	0.0	0.0	0.0	0.0	0.0
Unassigned *Actinobacteria* (4)	0.1	0.1	0.1	0.1	0.1	0.1	0.0	0.0	0.1	0.0	0.0	0.0	0.0	0.0	0.0	0.0	0.1	0.0	0.1	0.1	0.0	0.0
Coriobacteriia,																						
Coriobacteriaceae (1)	0.0	0.0	0.0	0.0	0.0	0.0	0.0	0.0	0.0	0.0	0.0	0.0	0.0	0.0	0.0	0.0	0.0	0.0	0.0	0.0	0.0	0.0
Rubrobacteria,																						
Rubrobacteriaceae (4)	0.1	0.2	0.3	0.2	0.3	0.2	0.1	0.1	0.2	0.0	0.2	0.1	0.1	0.1	0.1	0.1	0.1	0.1	0.1	0.1	0.1	0.1
Thermoleophilia,																						
Gaiellaceae (7)	0.7	0.7	0.7	0.8	0.7	0.7	0.5	0.2	0.5	0.2	0.4	0.3	0.4	0.1	0.2	0.2	0.3	0.2	0.3	0.5	0.3	0.3
Parviterribacteraceae (1)	0.0	0.0	0.0	0.0	0.0	0.0	0.0	0.0	0.0	0.0	0.0	0.0	0.0	0.0	0.0	0.0	0.0	0.0	0.0	0.0	0.0	0.0
Patulibacteraceae (4)	0.0	0.0	0.0	0.0	0.0	0.0	0.0	0.0	0.0	0.0	0.0	0.0	0.0	0.0	0.0	0.0	0.0	0.0	0.0	0.0	0.0	0.0
Solirubrobacteraceae (4)	0.9	0.8	1.0	1.0	0.8	0.8	0.5	0.2	0.5	0.2	0.5	0.4	0.4	0.2	0.3	0.3	0.4	0.3	0.4	0.7	0.5	0.4
Unassigned *Solirubrobacterales* (16)	0.3	0.4	0.4	0.4	0.3	0.3	0.3	0.1	0.2	0.1	0.2	0.1	0.1	0.1	0.0	0.1	0.1	0.2	0.2	0.4	0.2	0.3
Unassigned *Gaiellales* (27)	1.1	1.2	1.1	1.0	1.1	0.9	0.6	0.3	0.4	0.2	0.4	0.5	0.6	0.2	0.3	0.2	0.5	0.4	0.6	0.9	0.6	0.5
Unassigned *Thermoleophilia* (36)	0.6	0.3	0.4	0.3	0.3	0.3	0.2	0.1	0.2	0.1	0.2	0.2	0.2	0.1	0.1	0.2	0.2	0.1	0.3	0.4	0.1	0.2
Unassigned *Actinobacteria* (23)	0.5	0.4	0.4	0.5	0.5	0.4	0.2	0.1	0.2	0.1	0.4	0.3	0.2	0.1	0.1	0.1	0.3	0.2	0.4	0.3	0.2	0.2
Armatimonadetes,																						
Unassigned *Armatimonadetes* (1)	0.0	0.0	0.0	0.0	0.0	0.0	0.0	0.0	0.0	0.0	0.0	0.0	0.0	0.0	0.0	0.0	0.0	0.0	0.0	0.0	0.0	0.0
Bacteroidetes,																						
Bacteroidia,																						
Bacteroidaceae (5)	0.0	0.1	0.0	0.0	0.0	0.3	0.0	0.0	9.3	0.0	0.0	0.0	0.0	0.2	0.0	0.0	0.0	0.0	0.0	0.1	0.0	0.0
Porphyromonadaceae (2)	0.0	0.0	0.0	0.0	0.0	0.0	0.0	0.0	0.0	0.0	0.0	0.0	0.0	0.0	0.0	0.0	0.0	0.0	0.0	0.0	0.0	0.0
Prevotellaceae (1)	0.0	0.0	0.0	0.0	0.0	0.0	0.0	0.0	0.0	0.0	0.0	0.0	0.0	0.0	0.0	0.0	0.0	0.0	0.0	0.0	0.0	0.0

Sampling Time:	0 h						10 h		22 h		30 h											
Treatment:	C1	C2	C3	G	F	S	C	G	C	G	C1	C2	C3	G1	G2	G3	F1	F2	F3	S1	S2	S3
**Phyla, Class, Family**[b]	Relative Abundance (%)																					
Rikenellaceae (1)	0.0	0.0	0.0	0.0	0.0	0.0	0.0	0.0	0.0	0.0	0.0	0.0	0.0	0.0	0.0	0.0	0.0	0.0	0.0	0.0	0.0	0.0
Sphingobacteriia																						
Chitinophagaceae (9)	0.0	0.0	0.0	0.0	0.0	0.0	0.0	0.0	0.0	0.0	0.0	0.0	0.0	0.0	0.0	0.0	0.0	0.0	0.0	0.0	0.0	0.0
Cytophagia,																						
Cytophagaceae (11)	0.0	0.1	0.1	0.1	0.1	0.1	0.0	0.0	0.0	0.0	0.0	0.0	0.0	0.0	0.0	0.0	0.0	0.0	0.0	0.0	0.0	0.0
Flavobacteriia,																						
Flavobacteriaceae (7)	0.2	0.4	0.2	0.3	0.5	0.2	0.3	0.2	0.2	0.1	0.1	0.1	0.1	0.1	0.1	0.0	0.0	0.2	0.2	0.0	0.0	0.1
Sphingobacteriia,																						
Saprospiraceae (1)	0.0	0.0	0.0	0.0	0.0	0.0	0.0	0.0	0.0	0.0	0.0	0.0	0.0	0.0	0.0	0.0	0.0	0.0	0.0	0.0	0.0	0.0
Sphingobacteriaceae (1)	0.0	0.0	0.0	0.0	0.0	0.0	0.0	0.0	0.0	0.0	0.0	0.0	0.0	0.0	0.0	0.0	0.0	0.0	0.0	0.0	0.0	0.0
Unassigned *Sphingobacteriales* (3)	0.0	0.0	0.0	0.0	0.0	0.0	0.0	0.0	0.0	0.0	0.0	0.0	0.0	0.0	0.0	0.0	0.0	0.0	0.0	0.0	0.0	0.0
BRC1,																						
Unassigend BRC1 (1)	0.0	0.0	0.0	0.0	0.0	0.0	0.0	0.0	0.0	0.0	0.0	0.0	0.0	0.0	0.0	0.0	0.0	0.0	0.0	0.0	0.0	0.0
Chlamydiae,																						
Chlamydiae,																						
Chlamydiaceae (1)	0.0	0.0	0.0	0.0	0.0	0.0	0.0	0.0	0.0	0.0	0.0	0.0	0.0	0.0	0.0	0.0	0.0	0.0	0.0	0.0	0.0	0.0
Parachlamydiaceae (13)	0.0	0.0	0.0	0.0	0.0	0.0	0.0	0.0	0.0	0.0	0.0	0.0	0.0	0.0	0.0	0.0	0.0	0.0	0.0	0.0	0.0	0.0
Chlorobi,																						
Chlorobiales,																						
Unassigned *Chlorobiales* (1)	0.0	0.0	0.0	0.0	0.0	0.0	0.0	0.0	0.0	0.0	0.0	0.0	0.0	0.0	0.0	0.0	0.0	0.0	0.0	0.0	0.0	0.0
Chloroflexi,,																						
Anaerolineae																						
Anaerolineaceae (5)	0.0	0.0	0.0	0.0	0.0	0.0	0.0	0.0	0.0	0.0	0.0	0.0	0.0	0.0	0.0	0.0	0.0	0.0	0.0	0.0	0.0	0.0
Unassigned *Ardenticatenia* (2)	0.0	0.0	0.0	0.0	0.0	0.0	0.0	0.0	0.0	0.0	0.0	0.0	0.0	0.0	0.0	0.0	0.0	0.0	0.0	0.0	0.0	0.0
Caldilineae,																						
Caldilineaceae (11)	0.1	0.0	0.1	0.2	0.1	0.1	0.0	0.0	0.1	0.0	0.0	0.1	0.1	0.0	0.0	0.0	0.1	0.1	0.1	0.1	0.0	0.0
Chloroflexia,																						
Unassigned *Kallotenuales* (1)	0.0	0.0	0.0	0.0	0.0	0.0	0.0	0.0	0.0	0.0	0.0	0.0	0.0	0.0	0.0	0.0	0.0	0.0	0.0	0.0	0.0	0.0
Roseiflexaceae (6)	0.1	0.1	0.1	0.0	0.1	0.1	0.0	0.0	0.1	0.0	0.1	0.0	0.1	0.0	0.0	0.0	0.0	0.1	0.0	0.0	0.0	0.0
Ktedonobacteria,																						
Ktedonobacteraceae (2)	0.0	0.0	0.0	0.0	0.0	0.0	0.0	0.0	0.0	0.0	0.0	0.0	0.0	0.0	0.0	0.0	0.0	0.0	0.0	0.0	0.0	0.0
Thermosporotrichaceae (1)	0.0	0.0	0.0	0.0	0.0	0.0	0.0	0.0	0.0	0.0	0.0	0.0	0.0	0.0	0.0	0.0	0.0	0.0	0.0	0.0	0.0	0.0
Unassigned *Ktedonobacterales* (1)	0.0	0.0	0.0	0.0	0.0	0.0	0.0	0.0	0.0	0.0	0.0	0.0	0.0	0.0	0.0	0.0	0.0	0.0	0.0	0.0	0.0	0.0
Unassigned *Ktedonobacteria* (5)	0.0	0.0	0.1	0.0	0.0	0.0	0.0	0.0	0.0	0.0	0.0	0.0	0.0	0.0	0.0	0.0	0.0	0.0	0.0	0.0	0.0	0.0
Unassigned *Thermomicrobia* (28)	0.2	0.2	0.3	0.3	0.2	0.2	0.1	0.1	0.1	0.0	0.2	0.1	0.1	0.1	0.1	0.0	0.1	0.0	0.0	0.1	0.1	0.1
Unassigned *Chloroflexi* (41)	0.6	0.8	0.8	1.0	0.9	0.6	0.5	0.2	0.5	0.2	0.4	0.3	0.3	0.2	0.2	0.2	0.3	0.3	0.4	0.4	0.4	0.4
Cyanobacteria,																						
Chloroplast,																						
Unassigned *Chloroplast* (5)	0.0	0.0	0.0	0.0	0.0	0.0	0.0	0.0	0.0	0.0	0.0	0.0	0.0	0.0	0.0	0.0	0.0	0.0	0.0	0.0	0.0	0.0
Cyanobacteria,																						
Unassigned *Cyanobacteria* (2)	0.0	0.0	0.0	0.0	0.0	0.0	0.0	0.0	0.0	0.0	0.0	0.0	0.0	0.0	0.0	0.0	0.0	0.0	0.0	0.0	0.0	0.0
Unassigned *Cyanobacteria* (2)	0.0	0.0	0.0	0.0	0.0	0.0	0.0	0.0	0.0	0.0	0.0	0.0	0.0	0.0	0.0	0.0	0.0	0.0	0.0	0.0	0.0	0.0

Sampling Time:	0 h						10 h		22 h		30 h											
Treatment:	C1	C2	C3	G	F	S	C	G	C	G	C1	C2	C3	G1	G2	G3	F1	F2	F3	S1	S2	S3
**Phyla, Class, Family[b]**									**Relative Abundance (%)**													
Elusimicrobia,																						
Unassigned *Elusimicrobia* (1)	0.0	0.0	0.0	0.0	0.0	0.0	0.0	0.0	0.0	0.0	0.0	0.0	0.0	0.0	0.0	0.0	0.0	0.0	0.0	0.0	0.0	0.0
Fibrobacteres,																						
Fibrobacteria,																						
Fibrobacteraceae (2)	0.0	0.0	0.0	0.0	0.0	0.0	0.0	0.0	0.0	0.0	0.0	0.0	0.0	0.0	0.0	0.0	0.0	0.0	0.0	0.0	0.0	0.0
Firmicutes,																						
Bacilli,																						
Aerococcaceae (1)	0.0	0.0	0.0	0.0	0.0	0.0	0.0	0.0	0.0	0.0	0.0	0.0	0.0	0.0	0.0	0.0	0.0	0.0	0.0	0.0	0.0	0.0
Alicyclobacillaceae (1)	0.0	0.0	0.0	0.0	0.0	0.0	0.0	0.0	0.0	0.0	0.0	0.0	0.0	0.0	0.0	0.0	0.0	0.0	0.0	0.0	0.0	0.0
Bacillaceae (9)	1.3	1.2	1.5	1.1	1.3	1.2	0.8	0.5	0.7	0.6	1.1	1.1	1.2	1.0	1.0	0.7	1.6	1.3	1.4	1.2	0.6	0.9
Enterococcaceae (1)	0.0	1.3	0.0	0.0	0.0	0.0	0.0	0.8	1.0	0.0	0.0	0.0	0.0	0.0	0.0	0.7	0.0	0.0	0.0	0.7	0.0	0.0
Lactobacillaceae (2)	0.1	0.0	0.0	0.0	0.2	0.1	0.1	0.0	0.0	0.0	0.0	0.1	0.0	0.0	0.0	0.0	0.0	0.0	0.1	0.0	0.0	0.0
Paenibacillaceae (22)	0.1	0.1	0.0	0.0	0.1	0.1	0.1	0.0	0.1	0.1	0.1	0.2	0.1	0.2	0.2	0.1	0.1	0.1	0.1	0.3	0.2	0.1
Planococcaceae (7)	0.0	0.0	0.0	0.0	0.0	0.1	0.0	0.0	0.1	0.0	0.0	0.0	0.0	0.0	0.0	0.0	0.0	0.0	0.0	0.0	0.0	0.1
Staphylococcaceae (1)	0.0	0.0	0.0	0.0	0.0	0.0	0.0	0.0	0.0	0.0	0.0	0.0	0.0	0.0	0.0	0.0	0.0	0.0	0.0	0.0	0.0	0.0
Thermoactinomycetaceae (3)	0.0	0.0	0.0	0.0	0.0	0.0	0.0	0.0	0.0	0.0	0.0	0.0	0.0	0.0	0.0	0.0	0.0	0.0	0.0	0.0	0.0	0.0
Streptococcaceae (3)	0.1	0.0	0.2	0.1	0.0	0.0	0.1	0.1	0.0	0.2	0.0	0.0	0.1	0.0	0.0	0.0	0.1	0.0	0.0	0.0	0.0	0.3
Unassigned *Lactobacillales* (1)	0.0	0.0	0.1	0.0	0.0	0.2	0.0	0.0	0.3	0.0	0.0	0.0	0.0	0.0	0.0	0.0	0.0	0.0	0.0	0.0	0.2	0.0
Unassigned *Bacilli* (2)	0.1	0.1	0.1	0.1	0.1	0.1	0.0	0.0	0.0	0.0	0.0	0.0	0.0	0.0	0.0	0.0	0.0	0.0	0.0	0.0	0.0	0.0
Clostridia,																						
Clostridiaceae (22) **[A14]**	0.3	0.4	0.2	0.3	0.4	0.3	0.7	0.4	1.6	1.6	2.4	2.0	2.1	3.4	4.1	3.6	3.1	3.2	3.5	2.2	2.0	1.9
Unassigned *Clostridiales* (7)	0.0	0.0	0.0	0.0	0.0	0.0	0.0	0.0	0.1	0.0	0.3	0.2	0.3	0.0	0.0	0.0	0.0	0.0	0.0	0.2	0.1	0.2
Eubacteriaceae (1)	0.0	0.0	0.0	0.0	0.0	0.0	0.0	0.0	0.0	0.0	0.0	0.0	0.0	0.0	0.0	0.0	0.0	0.0	0.0	0.0	0.0	0.0
Gracilibacteraceae (1)	0.0	0.0	0.0	0.0	0.0	0.0	0.0	0.0	0.0	0.0	0.0	0.0	0.0	0.0	0.0	0.0	0.0	0.0	0.0	0.0	0.0	0.0
Heliobacteriaceae (3)	0.0	0.0	0.0	0.0	0.0	0.0	0.0	0.0	0.0	0.0	0.0	0.0	0.0	0.0	0.0	0.0	0.0	0.0	0.0	0.0	0.0	0.0
Lachnospiraceae (24)	0.0	0.0	0.0	0.0	0.1	0.2	0.1	0.0	1.1	0.5	3.1	4.1	4.3	3.0	3.2	4.0	2.5	3.5	5.6	5.2	4.7	4.8
Peptococcaceae (5)	0.7	0.7	0.9	0.6	0.8	0.8	0.7	0.3	0.5	0.5	0.7	0.4	0.7	0.4	0.6	0.5	0.7	0.8	1.0	0.7	0.8	0.8
Peptostreptococcaceae (6) **[GPT-4], [A8]**	0.1	0.1	0.2	0.0	0.2	0.2	1.8	0.5	3.1	2.4	3.2	3.1	3.5	3.6	4.3	4.2	7.9	7.6	9.4	3.1	2.6	2.2
Ruminococcaceae (28)	0.0	0.0	0.0	0.0	0.0	0.1	0.0	0.0	0.0	0.0	0.1	0.2	0.1	0.1	0.0	0.1	0.1	0.0	0.1	0.1	0.0	0.1
Erysipelotrichia,																						
Erysipelotrichaceae (6)	0.0	0.0	0.0	0.0	0.8	0.0	0.0	0.0	0.0	0.0	0.0	0.4	0.0	0.0	0.0	0.0	0.0	0.0	0.9	0.0	0.0	0.0
Limnochordia,																						
Unassigned *Limnochordales* (1)	0.0	0.0	0.0	0.0	0.0	0.0	0.0	0.0	0.0	0.0	0.0	0.0	0.0	0.0	0.0	0.0	0.0	0.0	0.0	0.0	0.0	0.0
Negativicutes,																						
Acidaminococcaceae (1)	0.0	0.0	0.0	0.0	0.0	0.0	0.0	0.0	0.0	0.0	0.0	0.0	0.0	0.0	0.0	0.0	0.0	0.0	0.0	0.0	0.0	0.0
Veillonellaceae (9)	0.0	0.0	0.0	0.0	0.0	0.1	0.0	0.0	0.0	0.0	0.0	0.0	0.0	0.1	0.0	0.0	0.0	0.0	0.0	0.0	0.0	0.0
Fusobacteria,																						
Fusobacteriia,																						
Fusobacteriaceae (1) **[GPT-5]**	1.3	1.5	0.6	1.3	1.2	1.4	13	10	20	22	23	18	17	16	15	15	15	16	11	22	29	30
Gemmatimonadetes,																						
Gemmatimonadaceae (15)	0.1	0.1	0.1	0.1	0.1	0.1	0.1	0.0	0.1	0.0	0.1	0.1	0.0	0.0	0.0	0.0	0.1	0.1	0.0	0.1	0.0	0.1
Unassigned *Gemmatimonadetes* (4)	0.0	0.0	0.0	0.0	0.0	0.0	0.0	0.0	0.0	0.0	0.0	0.0	0.0	0.0	0.0	0.0	0.0	0.0	0.0	0.0	0.0	0.0

Sampling Time:	0 h						10 h		22 h		30 h											
Treatment:	C1	C2	C3	G	F	S	C	G	C	G	C1	C2	C3	G1	G2	G3	F1	F2	F3	S1	S2	S3
Phyla, Class, Family[b]	Relative Abundance (%)																					
Latescibacteria,																						
Unassigned *Latescibacteria* (8)	0.0	0.0	0.0	0.0	0.0	0.0	0.0	0.0	0.0	0.0	0.0	0.0	0.0	0.0	0.0	0.0	0.0	0.0	0.0	0.0	0.0	0.0
Nitrospirae,																						
Nitrospira,																						
Nitrospiraceae (5)	0.1	0.1	0.1	0.1	0.1	0.1	0.0	0.0	0.0	0.0	0.0	0.1	0.0	0.0	0.1	0.0	0.1	0.1	0.0	0.1	0.0	0.0
Unassigned *Nitrospirales* (1)	0.0	0.0	0.0	0.0	0.0	0.0	0.0	0.0	0.0	0.0	0.0	0.0	0.0	0.0	0.0	0.0	0.0	0.0	0.0	0.0	0.0	0.0
Unassigned *Nitrospira* (12)	0.4	0.5	0.5	0.5	0.5	0.4	0.3	0.1	0.3	0.1	0.3	0.2	0.2	0.1	0.1	0.2	0.2	0.3	0.3	0.3	0.3	0.3
Planctomycetes,																						
Phycisphaerae,																						
Phycisphaeraceae (9)	0.0	0.0	0.0	0.1	0.0	0.0	0.0	0.0	0.0	0.0	0.0	0.0	0.0	0.0	0.0	0.0	0.0	0.0	0.0	0.0	0.0	0.0
Tepidisphaeraceae (40)	0.7	1.2	1.1	1.2	0.8	0.4	0.3	0.1	0.3	0.1	0.6	0.2	0.3	0.1	0.1	0.1	0.3	0.4	0.2	0.1	0.2	0.1
Unassigned *Phycisphaerae* (4)	0.0	0.0	0.0	0.0	0.0	0.0	0.0	0.0	0.0	0.0	0.0	0.0	0.0	0.0	0.0	0.0	0.0	0.0	0.0	0.0	0.0	0.0
Planctomycetacia,																						
Planctomycetaceae (400)	8.2	9.8	8.6	8.4	7.5	5.6	4.1	1.5	4.3	1.8	5.8	5.1	5.6	2.1	2.8	2.9	6.8	6.6	5.1	3.7	5.2	4.7
Unassigned *Planctomycetes* (23)	0.1	0.1	0.1	0.1	0.1	0.1	0.0	0.0	0.0	0.0	0.0	0.0	0.0	0.0	0.0	0.0	0.0	0.0	0.0	0.0	0.0	0.0
Proteobacteria,																						
Alphaproteobacteria																						
Acetobacteraceae (6)	0.0	0.1	0.0	0.1	0.0	0.0	0.1	0.0	0.0	0.0	0.0	0.0	0.0	0.0	0.0	0.0	0.1	0.0	0.0	0.1	0.0	0.0
Beijerinckiaceae (1)	0.0	0.0	0.0	0.0	0.0	0.0	0.0	0.0	0.0	0.0	0.0	0.0	0.0	0.0	0.0	0.0	0.0	0.0	0.0	0.0	0.0	0.0
Bradyrhizobiaceae (2)	0.7	0.8	0.9	1.0	1.1	0.9	0.5	0.2	0.5	0.3	0.5	0.4	0.4	0.2	0.2	0.2	0.4	0.4	0.3	0.4	0.2	0.5
Caulobacteraceae (1)	0.0	0.0	0.0	0.0	0.0	0.0	0.0	0.0	0.0	0.0	0.0	0.0	0.0	0.0	0.0	0.0	0.0	0.0	0.0	0.0	0.0	0.0
Erythrobacteraceae (1)	0.0	0.0	0.0	0.0	0.0	0.0	0.0	0.0	0.0	0.0	0.0	0.0	0.0	0.0	0.0	0.0	0.0	0.0	0.0	0.0	0.0	0.0
Holosporaceae (1)	0.0	0.0	0.0	0.0	0.0	0.0	0.0	0.0	0.0	0.0	0.0	0.0	0.0	0.0	0.0	0.0	0.0	0.0	0.0	0.0	0.0	0.0
Hyphomicrobiaceae (6)	0.3	0.4	0.4	0.4	0.3	0.3	0.3	0.1	0.2	0.1	0.1	0.3	0.2	0.0	0.1	0.0	0.2	0.1	0.2	0.3	0.2	0.1
Methylobacteriaceae (3)	0.1	0.1	0.2	0.2	0.2	0.2	0.1	0.1	0.1	0.0	0.1	0.1	0.1	0.1	0.0	0.1	0.1	0.0	0.1	0.2	0.1	0.2
Phyllobacteriaceae (3)	0.2	0.2	0.3	0.5	0.2	0.3	0.2	0.0	0.1	0.1	0.1	0.1	0.1	0.0	0.1	0.1	0.1	0.1	0.1	0.1	0.2	0.1
Rhizobiaceae (2)	0.0	0.0	0.0	0.0	0.0	0.0	0.0	0.0	0.0	0.0	0.0	0.0	0.0	0.0	0.0	0.0	0.0	0.0	0.0	0.0	0.0	0.0
Unassigned *Rhizobiales* (9)	0.1	0.1	0.2	0.1	0.2	0.1	0.1	0.0	0.1	0.0	0.1	0.1	0.1	0.0	0.0	0.0	0.1	0.0	0.1	0.0	0.1	0.0
Rhodobacteraceae (5)	0.1	0.1	0.1	0.1	0.1	0.1	0.0	0.0	0.1	0.0	0.0	0.0	0.0	0.0	0.0	0.0	0.0	0.0	0.0	0.1	0.1	0.0
Rhodobiaceae (2)	0.3	0.3	0.2	0.3	0.3	0.3	0.2	0.1	0.1	0.1	0.2	0.2	0.2	0.0	0.1	0.1	0.1	0.1	0.2	0.2	0.1	0.2
Rhodospirillaceae (12)	0.7	0.7	0.9	1.2	1.1	1.0	0.5	0.2	0.4	0.2	0.5	0.6	0.4	0.3	0.3	0.2	0.6	0.7	0.5	0.7	0.4	0.6
Unassigned *Rhodospirillales* (13)	0.4	0.4	0.4	0.7	0.6	0.6	0.3	0.1	0.3	0.1	0.2	0.4	0.3	0.1	0.1	0.1	0.3	0.3	0.2	0.4	0.2	0.3
Sphingomonadaceae (1)	0.0	0.0	0.0	0.0	0.0	0.0	0.0	0.0	0.0	0.0	0.0	0.0	0.0	0.0	0.0	0.0	0.0	0.0	0.0	0.0	0.0	0.0
Unassigned *Sphingomonadales* (1)	0.0	0.0	0.0	0.0	0.0	0.0	0.0	0.0	0.0	0.0	0.0	0.0	0.0	0.0	0.0	0.0	0.0	0.0	0.0	0.0	0.0	0.0
Xanthobacteraceae (4)	0.5	0.8	0.7	1.0	0.6	0.6	0.3	0.1	0.4	0.1	0.4	0.5	0.4	0.1	0.2	0.1	0.4	0.4	0.3	0.6	0.3	0.3
Unassigned *Rickettsiales* (2)	0.0	0.0	0.0	0.0	0.0	0.0	0.0	0.0	0.0	0.0	0.0	0.0	0.0	0.0	0.0	0.0	0.0	0.0	0.0	0.0	0.0	0.0
Unassigned *Alphaproteobacteria* (12)	0.1	0.1	0.1	0.1	0.2	0.1	0.1	0.0	0.1	0.0	0.1	0.1	0.0	0.0	0.0	0.0	0.0	0.0	0.0	0.1	0.0	0.1
Betaproteobacteria,																						
Alcaligenaceae (2)	0.1	0.1	0.1	0.1	0.1	0.1	0.0	0.1	0.0	0.0	0.0	0.0	0.0	0.0	0.0	0.0	0.0	0.0	0.0	0.1	0.0	0.0
Burkholderiaceae (4)	0.0	0.0	0.0	0.0	0.0	0.0	0.0	0.0	0.0	0.0	0.0	0.0	0.0	0.0	0.0	0.0	0.0	0.0	0.0	0.0	0.0	0.0
Comamonadaceae (9)	0.0	0.1	0.1	0.1	0.1	0.2	0.1	0.0	0.1	0.0	0.0	0.0	0.0	0.0	0.0	0.0	0.0	0.0	0.0	0.0	0.0	0.0
Oxalobacteraceae (1)	0.0	0.0	0.0	0.0	0.0	0.0	0.0	0.0	0.0	0.0	0.0	0.0	0.0	0.0	0.0	0.0	0.0	0.0	0.0	0.0	0.0	0.0
Neisseriaceae (1)	0.0	0.0	0.0	0.0	0.0	0.0	0.0	0.0	0.0	0.0	0.0	0.0	0.0	0.0	0.0	0.0	0.0	0.0	0.0	0.0	0.0	0.0

**Sampling Time:**	**0 h**						**10 h**		**22 h**		**30 h**											
**Treatment:**	**C1**	**C2**	**C3**	**G**	**F**	**S**	**C**	**G**	**C**	**G**	**C1**	**C2**	**C3**	**G1**	**G2**	**G3**	**F1**	**F2**	**F3**	**S1**	**S2**	**S3**
**Phyla, Class, Family[b]**	**Relative Abundance (%)**																					
Nitrosomonadaceae (17)	0.1	0.2	0.2	0.1	0.2	0.2	0.1	0.1	0.1	0.0	0.1	0.1	0.0	0.0	0.0	0.0	0.0	0.0	0.1	0.1	0.0	0.0
Rhodocyclaceae (3)	0.0	0.0	0.0	0.0	0.0	0.0	0.0	0.0	0.0	0.0	0.0	0.0	0.0	0.0	0.0	0.0	0.0	0.0	0.0	0.0	0.0	0.0
Unassigned *Betaproteobacteria* (20)	0.1	0.1	0.1	0.1	0.1	0.1	0.1	0.0	0.0	0.0	0.0	0.0	0.0	0.0	0.0	0.0	0.0	0.0	0.0	0.0	0.0	0.0
Deltaproteobacteria,																						
Bdellovibrionaceae (9)	0.1	0.1	0.0	0.1	0.1	0.1	0.0	0.0	0.0	0.0	0.0	0.0	0.0	0.0	0.0	0.0	0.0	0.0	0.1	0.1	0.1	0.0
Desulfovibrionaceae (1)	0.0	0.0	0.0	0.0	0.0	0.0	0.0	0.0	0.0	0.0	0.0	0.0	0.0	0.0	0.0	0.0	0.0	0.0	0.0	0.0	0.0	0.0
Desulfurellaceae (21)	0.4	0.4	0.5	0.6	0.7	0.6	0.4	0.1	0.3	0.1	0.3	0.2	0.3	0.1	0.1	0.1	0.2	0.1	0.3	0.4	0.2	0.4
Desulfuromonadaceae (2)	0.0	0.0	0.0	0.0	0.0	0.0	0.0	0.0	0.0	0.0	0.0	0.0	0.0	0.0	0.0	0.0	0.0	0.0	0.0	0.0	0.0	0.0
Geobacteraceae (8)	0.1	0.1	0.0	0.1	0.0	0.1	0.0	0.0	0.0	0.0	0.0	0.0	0.0	0.0	0.0	0.0	0.0	0.0	0.0	0.1	0.0	0.0
Archangiaceae (5)	0.1	0.1	0.2	0.2	0.1	0.1	0.1	0.0	0.1	0.0	0.1	0.0	0.0	0.0	0.0	0.0	0.1	0.1	0.1	0.1	0.0	0.1
Haliangiaceae (32)	0.2	0.2	0.2	0.2	0.2	0.1	0.2	0.0	0.1	0.1	0.2	0.1	0.2	0.1	0.1	0.0	0.1	0.1	0.1	0.3	0.2	0.2
Myxococcaceae (2)	0.0	0.0	0.0	0.0	0.0	0.0	0.0	0.0	0.0	0.0	0.0	0.0	0.0	0.0	0.0	0.0	0.0	0.0	0.0	0.0	0.0	0.0
Unassigned *Myxococcales* (45)	0.2	0.2	0.3	0.3	0.3	0.2	0.2	0.0	0.1	0.1	0.3	0.2	0.2	0.1	0.1	0.0	0.1	0.1	0.1	0.3	0.1	0.2
Nannocystaceae (4)	0.0	0.0	0.0	0.0	0.0	0.0	0.0	0.0	0.0	0.0	0.0	0.0	0.0	0.0	0.0	0.0	0.0	0.0	0.0	0.0	0.0	0.0
Phaselicystidaceae (3)	0.0	0.0	0.1	0.1	0.1	0.1	0.1	0.0	0.0	0.0	0.1	0.1	0.1	0.0	0.0	0.0	0.1	0.1	0.1	0.1	0.0	0.1
Polyangiaceae (18)	0.3	0.2	0.4	0.3	0.3	0.2	0.1	0.0	0.1	0.0	0.1	0.1	0.2	0.0	0.1	0.0	0.2	0.1	0.2	0.3	0.2	0.2
Sandaracinaceae (19)	0.1	0.1	0.1	0.1	0.2	0.2	0.1	0.0	0.1	0.0	0.0	0.2	0.2	0.1	0.1	0.0	0.1	0.1	0.1	0.2	0.1	0.1
Vulgatibacteraceae (1)	0.0	0.0	0.0	0.0	0.0	0.0	0.0	0.0	0.0	0.0	0.0	0.0	0.0	0.0	0.0	0.0	0.0	0.0	0.0	0.0	0.0	0.0
Oligoflexaceae (1)	0.0	0.0	0.0	0.0	0.0	0.0	0.0	0.0	0.0	0.0	0.0	0.0	0.0	0.0	0.0	0.0	0.0	0.0	0.0	0.0	0.0	0.0
Unassigned *Oligoflexales* (6)	0.0	0.0	0.0	0.0	0.0	0.0	0.0	0.0	0.0	0.0	0.0	0.0	0.0	0.0	0.0	0.0	0.0	0.0	0.0	0.0	0.0	0.0
Unassigned *Deltaproteobacteria* (8)	0.0	0.0	0.0	0.0	0.0	0.0	0.0	0.0	0.0	0.0	0.0	0.0	0.0	0.0	0.0	0.0	0.0	0.0	0.0	0.0	0.0	0.0
Epsilonproteobacteria,																						
Campylobacteraceae (1)	0.0	0.0	0.0	0.0	0.0	0.0	0.0	0.0	0.0	0.0	0.0	0.0	0.0	0.0	0.0	0.0	0.0	0.0	0.0	0.0	0.0	0.0
Gammaproteobacteria																						
Aeromonadaceae (2) **[GPT-1]**	23	21	15	16	17	22	31	62	23	46	20	23	20	46	41	39	24	18	15	16	13	13
Coxiellaceae (2)	0.0	0.0	0.0	0.0	0.0	0.0	0.0	0.0	0.0	0.0	0.0	0.0	0.0	0.0	0.0	0.0	0.0	0.0	0.0	0.0	0.0	0.0
Enterobacteriaceae (6) **[GPT-2], [GPT-3]**	3.3	3.1	7.0	3.1	5.8	7.2	6.0	4.0	4.1	4.1	3.0	3.5	3.5	4.6	4.0	4.6	4.0	4.2	4.7	4.6	3.2	4.2
Halieaceae (1)	0.0	0.0	0.0	0.0	0.0	0.0	0.0	0.0	0.0	0.0	0.0	0.0	0.0	0.0	0.0	0.0	0.0	0.0	0.0	0.0	0.0	0.0
Legionellaceae (3)	0.0	0.0	0.0	0.0	0.0	0.0	0.0	0.0	0.0	0.0	0.0	0.0	0.0	0.0	0.0	0.0	0.0	0.0	0.0	0.0	0.0	0.0
Pseudomonadaceae (2)	0.0	0.0	0.0	0.0	0.0	0.0	0.0	0.0	0.0	0.0	0.0	0.0	0.0	0.0	0.0	0.0	0.0	0.0	0.0	0.0	0.0	0.0
Shewanellaceae (2)	1.8	1.5	0.9	1.2	1.2	0.8	1.3	0.7	1.7	0.7	2.1	3.3	3.3	1.9	1.5	1.6	1.5	2.3	1.3	1.4	1.5	1.5
Xanthomonadaceae (3)	0.0	0.0	0.0	0.0	0.0	0.0	0.0	0.0	0.0	0.0	0.0	0.0	0.0	0.0	0.0	0.0	0.0	0.0	0.0	0.0	0.0	0.0
Unassigned *Xanthomonadales* (14)	0.1	0.1	0.1	0.1	0.1	0.0	0.1	0.0	0.0	0.0	0.0	0.1	0.1	0.0	0.0	0.0	0.0	0.0	0.0	0.1	0.0	0.0
Unassigned *Gammaproteobacteria* (3)	0.0	0.0	0.0	0.0	0.0	0.0	0.0	0.0	0.0	0.0	0.0	0.0	0.0	0.0	0.0	0.0	0.0	0.0	0.0	0.0	0.0	0.0
Saccharibacteria,																						
Unassigned *Saccharibacteria* (8)	0.0	0.0	0.0	0.0	0.0	0.0	0.0	0.0	0.0	0.0	0.0	0.0	0.0	0.0	0.0	0.0	0.0	0.0	0.0	0.0	0.0	0.0
Spirochaetae,																						
Spirochaetes,,																						
Spirochaetaceae (1)	0.0	0.0	0.0	0.0	0.0	0.0	0.0	0.0	0.0	0.0	0.0	0.0	0.0	0.0	0.0	0.0	0.0	0.0	0.0	0.0	0.0	0.0
Tectomicrobia																						
Unassigned *Tectomicrobia* (16)	0.8	0.8	1.2	1.2	1.4	1.4	0.8	0.3	0.8	0.3	0.6	0.8	0.8	0.5	0.5	0.4	1.0	0.8	0.6	1.0	0.7	0.8
Tenericutes,																						
Mollicutes,																						

Sampling Time:	0 h						10 h		22 h		30 h											
Treatment:	C1	C2	C3	G	F	S	C	G	C	G	C1	C2	C3	G1	G2	G3	F1	F2	F3	S1	S2	S3
**Phyla, Class, Family[b]**	**Relative Abundance (%)**																					
Mycoplasmataceae (6)	36	33	32	37	35	35	25	12	15	13	21	22	25	12	15	17	19	23	27	21	26	22
Unassigned *Entomoplasmatales* (3)	0.0	0.0	0.0	0.0	0.0	0.0	0.0	0.0	0.0	0.0	0.0	0.0	0.0	0.0	0.0	0.0	0.0	0.0	0.0	0.0	0.0	0.0
Thaumarchaeota,																						
Unassigned *Thaumarchaeota* (5)	0.0	0.0	0.0	0.0	0.0	0.0	0.0	0.0	0.0	0.0	0.0	0.0	0.0	0.0	0.0	0.0	0.0	0.0	0.0	0.0	0.0	0.0
TM*6_*Dependentiae,																						
Unassigned *TM*6_*Dependentiae* (1)	0.0	0.0	0.0	0.0	0.0	0.0	0.0	0.0	0.0	0.0	0.0	0.0	0.0	0.0	0.0	0.0	0.0	0.0	0.0	0.0	0.0	0.0
Verrucomicrobia,																						
Unassigned *OPB*35 *soil group* (29)	0.1	0.1	0.2	0.1	0.1	0.2	0.1	0.0	0.1	0.0	0.0	0.0	0.0	0.0	0.0	0.0	0.0	0.0	0.0	0.0	0.0	0.0
Spartobacteria,																						
Chthoniobacteraceae (16)	0.0	0.0	0.0	0.0	0.1	0.0	0.0	0.0	0.0	0.0	0.0	0.0	0.0	0.0	0.0	0.0	0.0	0.0	0.0	0.0	0.0	0.0
Xiphinematobacteraceae (3)	1.4	2.2	2.3	2.8	2.4	1.8	1.3	0.5	1.0	0.8	1.7	1.2	1.0	0.6	0.7	0.5	1.0	1.3	1.5	0.9	1.0	1.2
Unassigned *Chthoniobacterales* (19)	0.3	0.4	0.3	0.3	0.3	0.3	0.1	0.1	0.1	0.1	0.2	0.1	0.1	0.0	0.1	0.1	0.1	0.1	0.2	0.1	0.1	0.1
Opitutae,																						
Opitutaceae (2)	0.0	0.0	0.0	0.0	0.0	0.0	0.0	0.0	0.0	0.0	0.0	0.0	0.0	0.0	0.0	0.0	0.0	0.0	0.0	0.0	0.0	0.0
Unassigned *Opitutae* (1)	0.0	0.0	0.0	0.0	0.0	0.0	0.0	0.0	0.0	0.0	0.0	0.0	0.0	0.0	0.0	0.0	0.0	0.0	0.0	0.0	0.0	0.0
Verrucomicrobiae,																						
Verrucomicrobiaceae (7)	0.0	0.0	3.8	0.0	0.0	0.1	0.0	0.0	0.0	0.0	0.0	0.0	0.0	0.1	0.0	0.0	0.0	0.0	0.0	0.0	0.0	0.0

[a]Samples of the three replicates of the 16S rRNA control treatment at 0 h, and all 16S rRNA treatments at 30 h were analyzed separately. Samples of the three replicates were pooled for each of the other treatments at 0 h, 10 h, 22h, or 30 h. Identification numbers (e.g., C1) indicate the respective replicates. Abbreviations: C, unsupplemented control; S, succinate; F, formate; G, glucose.

[b]The number of phylotypes are shown in parenthesis. Abundant responsive group phylotypes from Figure 6 are bold and in brackets.
